# Comparative *de novo* transcriptome analysis of flower and root of *Oliveria decumbens* Vent. to identify putative genes in terpenes biosynthesis pathway

**DOI:** 10.3389/fgene.2022.916183

**Published:** 2022-08-04

**Authors:** Amir Khodavirdipour, Reza Safaralizadeh, Mehdi Haghi, Mohammad Ali Hosseinpourfeizi

**Affiliations:** Department of Biology, Faculty of Natural Sciences, University of Tabriz, Tabriz, Iran

**Keywords:** co-expression network, computational genetics, illumine, medicinal plant, terpenoid

## Abstract

The *Oliveria decumbens* Vent. is a wild, rare, annual medicinal plant and endemic plant of Iran that has metabolites (mostly terpenes) which make it a precious plant in Persian Traditional Medicine and also a potential chemotherapeutic agent. The lack of genetic resources has slowed the discovery of genes involved in the terpenes biosynthesis pathway. It is a wild relative of *Daucus carota*. In this research, we performed the transcriptomic differences between two samples, flower and root of *Oliveria decumbens,* and also analyze the expression value of the genes involved in terpenoid biosynthesis by RNA-seq and its essential oil’s phytochemicals analyzed by GC/MS. In total, 136,031,188 reads from two samples of flower and root have been produced. The result shows that the MEP pathway is mostly active in the flower and the MVA in the root. Three genes of *GPP*, *FPPS*, and *GGPP* that are the precursors in the synthesis of mono, di, and triterpenes are upregulated in root and 23 key genes were identified that are involved in the biosynthesis of terpenes. Three genes had the highest upregulation in the root including, and on the other hand, another three genes had the expression only in the flower. Meanwhile, 191 and 185 upregulated genes in the flower and root of the plant, respectively, were selected for the gene ontology analysis and reconstruction of co-expression networks. The current research is the first of its kind on *Oliveria decumbens* transcriptome and discussed 67 genes that have been deposited into the NCBI database. Collectively, the information obtained in this study unveils the new insights into characterizing the genetic blueprint of *Oliveria decumbens* Vent. which paved the way for medical/plant biotechnology and the pharmaceutical industry in the future.

## Introduction

The *Oliveria decumbens* Vent. is an aromatic annual herb from the Apiaceae family and endemic to Iran. The flower of the plant is prescribed in Persian Traditional Medicine (PTM) in the form of dry powder/infusion for dyspepsia, pyrexia, and diarrhea also as an anti-inflammatory and antibacterial agent (Alizadeh Behbahani et al., 2018; Eftekhari et al., 2021). The height of the plant varies from 20 to 45 cm. Mostly collected from the >2500 m heights from the skirts of central Zagros mountains in Fars, Chaharmahal & Bakhtiari, Kohgilouyeh & Boyerahmad, Boushehr, and Khuzestan provinces. The top chemical compounds have been reported in the plant including thymol, carvacrol, gamma-terpinene, and para-cymene (Khoshbakht et al., 2020). Each one of them has significant chemotherapeutic agent potential and also antibacterial properties (Ashraf, 2020; Ashraf et al., 2022). In this plant, they are available in high percentages which makes it a great candidate as a plant-based anti-cancer agent. In a very latest study, Khodavirdipour and his team elucidated the anti-cancer and anti-metastatic effect of this rare medicinal plant on HT-29 colorectal cell line (Khodavirdipour et al., 2021).

The core structure of terpenes consists of IPP (isopentenyl pyrophosphate) and its isomer, DMAPP (dimethylallyl pyrophosphate), where both of them synthesize via the cytosolic pathway, MVA (mevalonate pathway), and non-mevalonate plastid pathway, MEP (methylerythritol 4-phosphate). MVA pathway starts with 3 units of Acetyl-CoA and finally ends up in the production of FPP (Farnesyl pyrophosphate) (Bergman et al., 2019). The FPP has been formed by a condensation reaction of 2 units of IPP and a single unit of DMAPP and is an immediate precursor to all terpenes and terpenoids and sterols. Pyruvate and glyceraldehyde 3-phosphate are precursors in plastid-based MEP pathways. GPP (geranyl pyrophosphate) is a precursor to monoterpenes, diterpenes, and tetraterpenoids (carotenoids) and is produced by condensation of one unit DMAPP and one unit IPP. In later stages, the linear carbon backbone of GPP and FPP is converted into the main backbone of terpenes by TPS (terpene synthase) enzymes. Saponins are a terpenoid subclass and the biggest class of plant metabolites. They are active ingredients in more than 100 families of sea and soil endophytic fungi. The complex structure of Saponins is portrayed in physical, chemical, biological, and medicinal variations and resulted in Saponin’s importance in the health industry, cosmetics, and pharmaceutical sectors (Netala et al., 2014). Despite research conducted in gene profiling on genes involved in terpenes biosynthesis, still many mechanisms behind the secondary metabolite synthesis. Identification of key genes involving pathways is unknown and needed in-depth analysis (Sun et al., 2010; Hwang et al., 2015). There are numerous studies conducted to identify the genes involved in terpenes synthesis by the NGS method. In one of the studies using the NGS aiming to identify the involved genes in Saponins synthesis in *P. grandiflorum*, they produced more than 58 million reads, and by employing the *de novo* assembly genes, expression profiles in triterpenes synthesis have been determined (Ma et al., 2016). In another research on Siraitia grosvenorii to find the genes in the biosynthesis of triterpenes, more the 48 million reads have been analysed from the 2 samples from different time frames. The first one is 50 days and the second one 70 days after the flowering by the Illumina solexa method. The results suggest that there are seven gene families of Cytochromes P450 (CYP450) and five gene families of Uridine diphosphate glucose (UDPG) in magrosides biosynthesis (Tang et al., 2011). 18α-glycyrrhetinic acid and 18β-glycyrrhetinic acids are the two core and key structural medicinal compounds accumulated in *Glycyrrhiza* glabra. In RNA-seq research, the effects of three different level X-ray emissions were evaluated on the triterpenes biosynthesis pathway. Finally, 1376 genes, under influence of treatment, had the highest upregulation, and the genes involved in this pathway vigorously affected the synthesis above triterpenes (Gao et al., 2020). The comparative transcriptomic analysis of *Momordica charantia* was performed by RNA-seq. The findings were showing that despite fruits being the main accumulation sites of Cucurbitacins, but the highest upregulation and expression of genes happens in the leaves of the plants. This phenomenon displays that the biosynthesis of the Cucurbitacins occurs in the leaves and is transferred and amasses in the fruits (Takase et al., 2019). In another project, root, stem, and the leaves of *Entada phaseoloides* analyzed by NGS RNA-seq methods and yield more than 53 million reads have been produced. The results suggest that the highest upregulation of genes involved in triterpenes synthesis was observed in the stem of the plant (Liao et al., 2020). Other studies were also performed on mungbean, soybean, and Indian barnyard millet (Li et al., 2017; Jayakodi et al., 2019; Sudha et al., 2022). There is no study so far conducted to identify the key genes responsible for terpenes biosynthesis and, by and large, the reason behind the medicinal properties of the *Oliveria decumbens* Vent. by the latest sequencing techniques, and current research is the first of its kind in research on *de novo* assembly study on the rare endemic plant of Iran. This study designed and performed to identify the genes involved in the terpenoid biosynthesis pathway and build an expression profile of those genes in both root and flower of the *Oliveria decumbens* aiming to compare the intensity of metabolite synthesis in root and flower and also to find the exact site of production and accumulation of those secondary metabolites in both organs. Besides that, a complete metabolomic analysis of root and flower of the plant was performed for the first time to identify medicinally valued compound.

## Materials and methods

### Plant material

The study was carried out in compliance with all relevant guidelines and regulations. The plant samples include the root and flower of the wild plant *Oliveria decumbens* Vent. collected from the >2000m heights of the Zagros mountains of Kazeroun city-Fars province at the end of May 2020, identified by Dr. A.R. Khosravi, Division of Plant Biology, Department of Biology, Faculty of Sciences, Shiraz University-Fars province (herbarium #56024).

### Essential oil isolation and GC-MS analysis

The shadow-dried powder of the two samples (120 gm of root and 150 gm of flower) was exposed to hydro-distillation for 4 h by a Clevenger device. The obtained oil was again dried with anhydrous sodium sulfate and then later kept at 4°C.

GC-MS was carried out by Agilent GC (7890A)/MS (5975) with a capillary column, He gas carrier with a flow rate of 1.5 ml/min, flame ionization detector, and a split ratio of 1:25. The temperature was set at 50 °C for the column for 1 min and then for 265 °C for the heating purpose at the rate of 2.5°C/min, then stored at 265 °C for 20min. detector temp at 300 °C, injector temp at 265 °C flow of Helium at 35 ml/min with the airflow rate of 400 ml/min. The MS set off at 70 eV ionization energy. Retention times have been recorded and calculated by utilizing the n-alkanes retention times which have been injected in the same condition after the oil (www.agilent.com). The final found chemical compounds were reported by the “Agilent’s ChemStation Integrator” algorithm (www.agilent.com).

### RNA isolation, sequencing, and trimming

The total RNA extraction of two samples (flower and root) was performed on healthy and suitable samples by the Japelagi method (Japelaghi et al., 2011). The flower parts used for this study were the petal and last 10–12 cm of the root of the plant. Quality and quantity of the extracted RNA were analyzed and suitable samples have been sent to Macrogen (Seoul-South Korea) for RNA-seq by Illumina HiSeq 2000 platform with paired-end and read length of 101 bp at a sequencing depth of 10G. All the primary analyses include trimming, *de novo* assembly, mapping, nucleotide BLAST, and statistical analysis done by CLC Genomics Workbench 20.0.4 (http://www.qiagenbioinformatics.com). The software has been accessed for analyzing and statistical purposes from September to early November 2021.

### *De novo* transcriptome assembly, mapping, and annotation

After the *de novo* assembly of both the root and flower of the plant, they separately assembled on the genome for the mapping. The statistical analysis was done by the Z test method ((Kal et al., 1999). Unigenes BLAST was performed based on the NCBI nucleotide database and finally, genes were selected by highest e-value and identity percentage, for gene ontology and network analyses and performed Convert Gene ID (homology search) based on the *Arabidopsis thaliana* as a model organism. To convert gene identifications (GI) of identified genes to homolog GIs in *Arabidopsis thaliana*, the ensemble plant database and web-based Biomart software were used ((Kinsella et al., 2011; Hussain et al., 2021).

### Gene ontology and co-expression network analysis

Cytoscape 3.7.2 software (Shannon et al., 2003) and BinGo plug-in (Maere et al., 2005) were used for gene ontology purposes. Gene co-expression network for selected genes from both root and flower of *Oliveria decumbens* Vent. was performed and re-constructed by STRING web service (Szklarczyk et al., 2017) Cytoscape v.3.7.2. Network topology was evaluated and analyzed by the NetworkAnalyzer 2.7 plug-in of Cytoscape software (Assenov et al., 2008).

### Validation of RNA-seq results

To validate, based on the functional annotation importance, five genes related to plants are involved in terpenoid synthesis in *Oliveria decumbens* Vent. has been selected for validation through quantitative real-time PCR (qRT-PCR), and based on assembled sequence, the suitable primer for real-time-PCR has been designed by Primer3 plus web tools (Table 1) (Untergasser et al., 2007). The *Actin7* gene has been chosen as the reference gene. Consequently, the DNA-free RNA was used for first-strand cDNA synthesis by transcriptor First Strand cDNA Synthesis Kit (Cinnagen, Tehran, Iran) following the manufacturer’s instructions. PCR amplification was performed using 2× SYBR Green PCR Master Mix (Roche Applied Science, Penzberg, Germany) and Light-Cycler^®^ 480 (TaKaRa, Kusatsu, Shiga, Japan) following the standard protocol Relative expression of desired genes was obtained based on their Ct and analyzed by Pfaffl formula and calculated by 2^–ΔΔCT^ (Livak and Schmittgen, 2001).

**TABLE 1:** Properties of primers used in real-time PCR reaction.

Primers		Sequence	Melting temperature (°C)	Product length (bp)
*SQS*	Forward	5′-GAT​GGA​TCA​CGC​ATT​GTT​TG-3′	59.9	156
	Reverse	5′-TGC​AAG​GAT​TCA​GGA​GCT​TT-3′	60.0	
*SQE*	Forward	5′-ACC​AGG​AAC​ATC​AAC​CAA​GC-3′	60.0	202
	Reverse	5′-CTG​TGA​TGG​CTG​CTT​TTC​AA-3′	60.0	
*GPPS*	Forward	5′-ACG​CTG​TAA​TGG​GGA​ACA​AG-3′	60.0	161
	Reverse	5′-CGT​AGT​GGT​CAT​TTG​CAT​GG-3′	60.0	
*HMGR*	Forward	5′-AAC​TGT​CGG​AGG​TGG​AAC​AC-3′	60.0	168
	Reverse	5′-GCA​GCT​ATG​GCA​GAC​ATC​AA-3′	60.0	
*PMK*	Forward	5′-GGT​TGG​TGC​TGT​CAG​GAA​AT-3′	60.0	198
	Reverse	5′-GCT​TGC​TCC​ATC​CAC​TCT​TC-3′	60.0	
*MVD*	Forward	5′-TGC​GAT​GCT​ACT​CAA​GAA​CG-3′	60.2	166
	Reverse	5′-TTC​GCA​GAT​CAC​TGT​CAA​CC-3′	59.8	
*IPP*	Forward	5′-GTG​GGG​AGA​ACA​TGA​ATT​GG-3′	60.2	249
	Reverse	5′-TCC​ATA​TCG​GCA​ACT​TCC​TC-3′	60.0	
*Actin-7*	Forward	5′-TGT​GCC​TGC​CAT​GTA​TGT​TG -3′	59.6	160
	Reverse	5′-AGC​AAG​GTC​AAG​ACG​AAG​GA -3′	59.3	

## Results and discussion

### Metabolite components analysis by GC/MS in both flower and the root of *Oliveria decumbens*

GC/MS data which is analyzed by “Agilent’s Chemstation Integrator” showed that the compounds found in the flower include (in order of %, higher to lower): gamma-terpinene, p-cymene, limonene, thymol, beta-pinene, carvacrol, (23S)-ethylcholest-5-en-3beta-ol, clionasterol, beta-myrcene, myristicin, alpha-pinene, alpha-thujene, alpha-terpinene, 4,5-dimethyl-1,2-phenylenediamine, and 3-carene; while the chemical composition of *Oliveria decumbens* Vent. root deciphered for the very first time and shed light for future in detail *in vitro* and *in vivo* research. The top 15 compounds of the root include linolyl acetate, linalool, alpha-pinene, 1,8-cineol, alpha-terpinolene, camphor, dl-limonene, camphene, p-cymene, alpha-terpinene, gamma-terpinene, nonadecane, carvacrol, beta-Myrcene, and isoborneol (Tables 2, 3).

**TABLE 2:** Chemical composition of the *Oliveria decumbens* Vent. flower as shown from high to low in respect to their presence percentage in the sample. AC (anti-cancer), AI (anti-inflammatory), AB (antibacterial), PE (phlegm eliminator), AT (antitussive), AO (antioxidant), AA (anti-anxiety), BD (bronchodilator), AF (Antifungal), WH (wound healing), AD (antidiabetic), AM (antimutagenic), AN (analgesic), D (digestion), IB (immune system booster), AO (anti-osteoporosis).

Name	Area%	Quality	RT (min)	Function
Gamma-terpinene	42.11	96	8.402	AC, AI, AB, AO
Cymene	34.50	94	7.499	PE, AT, AB, AN, AI, AC, AA
Limonene	5.31	97	7.671	AI, AO, AA
Thymol	5.22	98	13.83	AI, AB, AC
β-Pinene	3.61	98	6.488	AA, BD, AI
Carvacrol	3.37	97	13.861	AB, AF, AI, WH
(23S)-ethylcholest-5-en-3beta-ol	1.92	94	43.778	AB
Clionasterol (gamma-sitosterol)	0.77	98	42.917	AD
Beta-myrcene	0.66	99	6.794	AB, AM, AI, AN
Myristicin	0.61	89	18.448	AI, AA, D, AB, AD, AC
Alpha-pinene	0.50	98	5.632	AA, BD, AI, IAA, E, H, AC
Alpha-thujene	0.47	97	5.481	AI, AB
Alpha-terpinene	0.41	95	7.38	AB, IB
4,5-Dimethyl-1,2-phenylenediamine	0.40	98	25.784	AB
3-Carene	0.15	99	7.261	AI, AO, AB

**TABLE 3:** Chemical composition of the *Oliveria decumbens* Vent. flower as shown from high to low in respect to their presence percentage in the sample. IAA (inhibiting acetylcholinesterase activity), E (euphoria), H (Hypervigilance), NP (neuroprotective), N (norgestimate-ethyl estradiol), AH (antihypertensive), AV (antiviral).

Name	Area%	Quality	RT (min)	Function
Linalyl acetate	21.78	96	13.031	AO, AI, AB
Linalool	14.94	94	9.383	AI, AB
Alpha-pinene	12.82	97	5.673	AA, BD, AI, IAA, E, H, AC
1,8-Cineole	9.76	98	7.629	AI, AO
Alpha-terpinolene	7.78	98	9.072	AC, AO
Camphor	5.30	97	10.058	AB, AF, AI
dl-Limonene	4.86	94	7.702	AI, AO, AA
Camphene	4.12	98	5.902	AN, AI, AF
Cymene	3.16	99	7.448	PE, AT, AB, AN, AI, AC, AA
Alpha-terpinene	2.23	89	7.375	AO, AC
Gamma-terpinene	1.89	98	8.314	AB, AI, AO, AC
Nonadecane	1.49	97	26.639	-
Carvacrol	1.39	95	13.861	AB, AF, AI, WH
Beta-myrcene	0.97	98	6.763	AB, AM, AI, AN
Isoborneol	0.97	99	10.494	AV, AB, NP
Heneicosane	0.54	91	28.778	AF
Z-5-nonadecene	0.53	95	25.141	-
Cyclohexene, 4-methyl-1-(1-methylethyl)-	0.53	92	7.733	-
Tricyclene	0.43	95	5.378	N
l-Phellandrene	0.39	93	7.064	AO, AI
4,5,6,7-Tetrahydro-1(3H)-isobenzofuranone	0.36	90	13.311	-
Trans beta-ocimene	0.36	96	8.065	AI, AF, AV, AB
Beta-pinene	0.36	98	6.457	AO, AI, AN
Borneol L	0.36	99	10.68	D, AI, AA
Neryl acetate	0.32	94	13.493	AO
Alpha-terpineol	0.22	90	11.298	AO, AC, AH
Geranyl acetate	0.21	93	15.537	-
Eicosane	0.20	93	27.242	AB, AI
Heptadecane	0.20	92	22.106	AO
m-Nitrophthalic acid	0.18	91	34.382	-
D-Fenchyl alcohol	0.16	97	9.575	-
(3Z)-Cembrene A	0.13	93	12.361	-
4-Octene, 2,6-dimethyl-, [S-(Z)]-	0.11	94	5.191	-
Hexadecane	0.08	93	31.648	-
Octadecane	0.07	97	30.247	-
Junipene	0.07	97	16.372	AF, AO
m-Mentha-1 (7),8-diene	0.05	92	10.198	-
Beta-phellandrene	0.03	96	6.363	AB, AF
E-14-Hexadecenal	0.02	96	21.634	-
1-Nonadecene (CAS)	0.02	95	28.327	-
l-Fenchone	0.02	92	8.719	AI, AF, AN
Germacrene-D	0.01	90	16.263	AB
Methyldiethylborane	0.01	92	24.181	-

Totally, 136,031,188 reads were produced, and after trimming and removing the adaptor sequences, 135,009,232 high-quality reads were used for *de novo* assembly (Table 4). After the *de novo* assembly, both sequenced samples of root and flower were separately mapped on the assembled genome. Out of 74,140,058 reads, 63,038,304 reads for the flower and out of 61,264,048 reads of root, 50,603,051 reads were successfully mapped (Table 5).

**TABLE 4:** Brief reports on quality and quantity of sequencing and *de novo* assembly.

	Parameter	Length
Unigene measurements (excluding scaffolded regions)	N75	750
	N50	1,259
	N25	2,094
	Minimum	53
	Maximum	20,380
	Average	978
	Count	32,734
	Total	32,011,608
Unigene measurements (including scaffolded regions)	N75	836
	N50	1,368
	N25	2,235
	Minimum	500
	Maximum	20,380
	Average	1,181
	Count	27,118
	Total	32,036,864

**TABLE 5:** Brief reports on mapping of flower and the root of the *Oliveria decumbens* Vent. with the *de novo* assembly transcriptome.

Mapping	Flower		Root	
	Number of fragments	Percentages of total	Number of fragments	Percentages of total
Counted fragments	59,642,553	80.74	48,020,831	78.55
unique fragments	58,572,566	79.29	46,814,066	76.57
non-specifically	1,069,987	1.45	1,206,765	1.97
Uncounted fragments	14,230,033	19.26	13,115,815	21.45
Total fragments	73,872,586	100.00	61,136,646	100.00

The mapping result showed that out of 27,118 unigenes, 23,219 unigenes have shown a significant differential expression at *p* < 0.01. To a great extent, 10,685 unigenes of root showed at least double upregulation of gene compared to the flower of *Oliveria decumbens* vent. and vice versa, 3,449 unigenes of flower displayed at least double upregulation in comparison to the root.

1,060 unigenes were specifically expressed in a single sample only (either root or flower). Based on the mapping results, upregulated unigenes were selected by thresholds such as *p* < 0.01, fold change >10, and < -10, and also a minimum of 5000 expression copies per unigenes in both flower and root samples. 191 and 189 unigenes from the flower and the root, respectively, selected for the BLAST (Supplementary Material S1). The selected unigenes primarily BLAST with all organisms. The BLAST findings determined that *Oliveria decumbens* Vent. has the most similarity to the *Daucus carota* with 78% similarity. In the next step, the GENE ID of identified genes is converted into GENE ID of homolog genes in *Arabidopsis thaliana* as a model organism (Supplementary Material S2).

### Gene ontology

**FIGURE 1:**
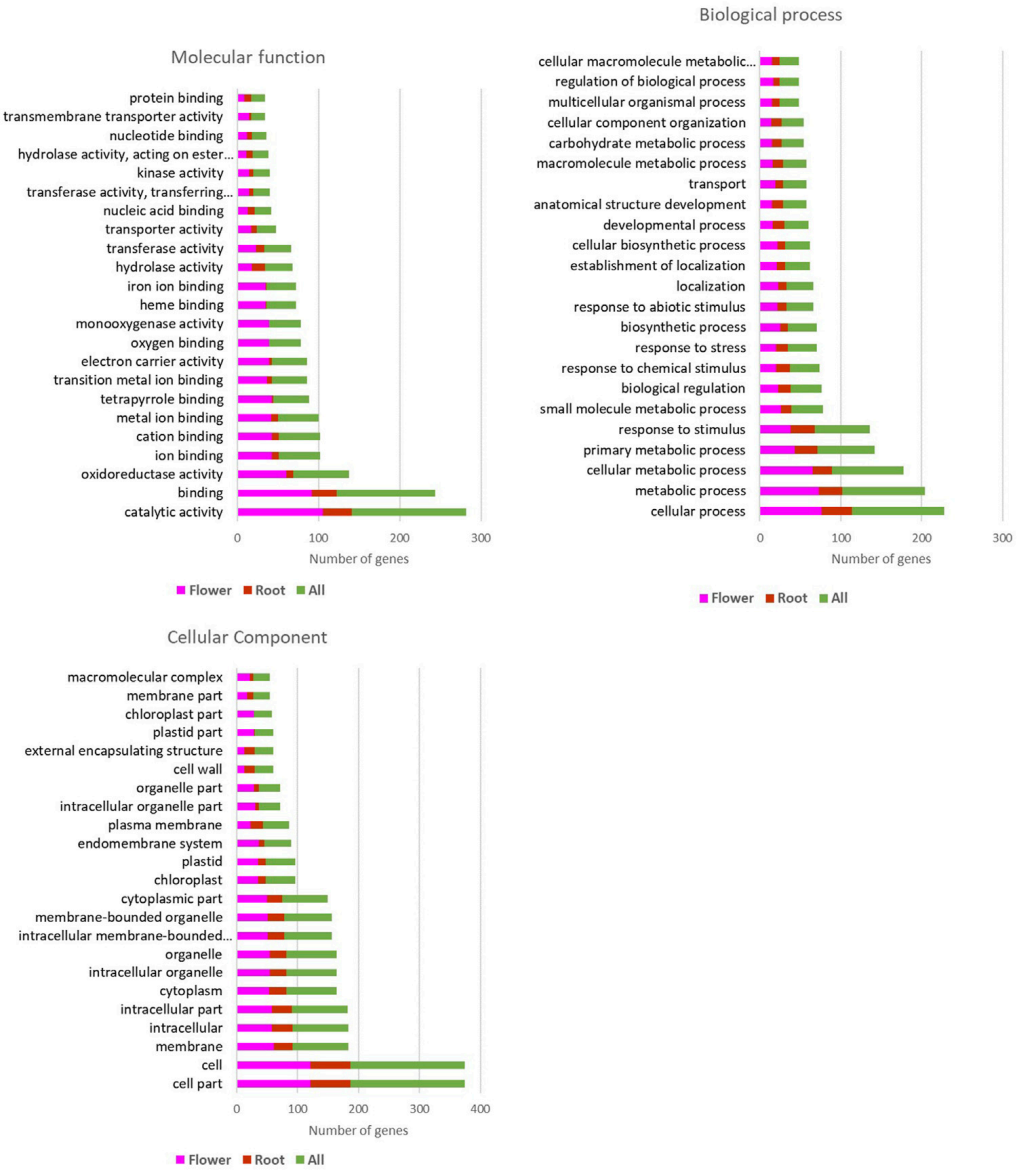
Gene ontology results of the upregulated genes in the flower and the root of the *Oliveria decumbens* Vent.

The result of upregulated genes in both flower and root of the *Oliveria decumbens* Vent. showed that 115 cellular components, 772 biological pathways, and 98 molecular functions at the significant level of 5% had at least one representative in the upregulated genes sector. As shown in Figure 1, the flower of the *Oliveria decumbens* Vent. is three times more active than root tissue. It has more active organelles and displays distinct and diverse biological pathways and molecular functions. Undoubtedly, one of the reasons shall be the diverse organelles of the flower with distinguished functions and sundry cellular tissues in comparison to the root structure (Supplementary Material S3).

### Analyzing the expression of genes involved in terpenes biosynthesis pathway in *Oliveria decumbens* Vent.

**FIGURE 2:**
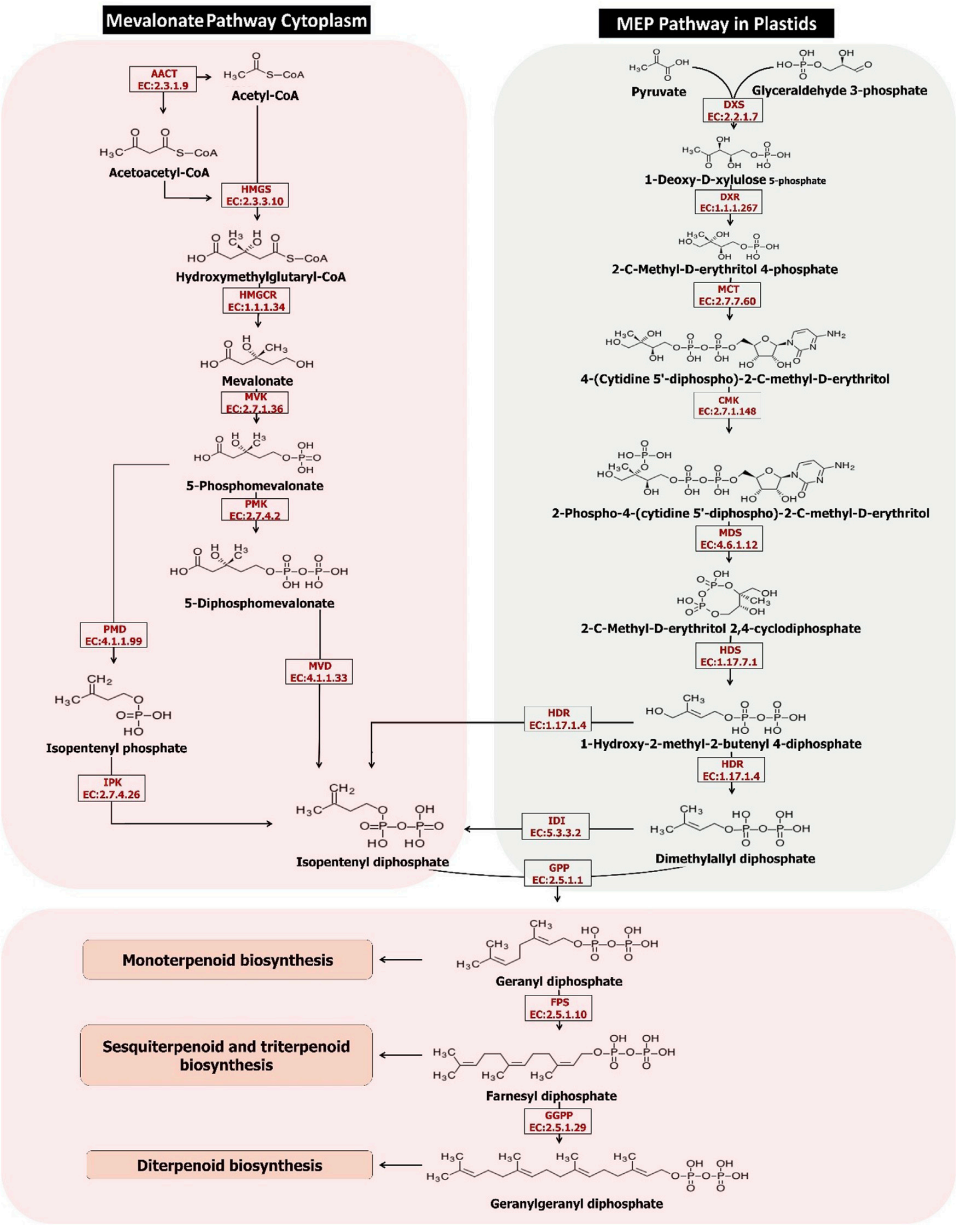
The terpenoids biosynthesis pathways in the *Oliveria decumbens* Vent.

The biosynthesis of isoprenoids is a vital process in all living organisms. Isoprenoids also have economic importance in agriculture and industries. Prenyl-PP (prenyl diphosphate) are precursors of all isoprenoids. Unlike the biosynthesis of isoprenoids in other living organisms, prenyl diphosphate is synthesized by two distinct pathways: MEP and MVA. The 2-C-methyl-D-erythritol 4-phosphate (MEP) pathway in plastids and the cytoplasmic pathway of the mevalonate (MVA) (Figure 2). However, all living organisms use only one of the above pathways, except plants (Yang et al., 2012). Plants optimize the regulation of isoprenoid biosynthesis according to ATP availability and fixed carbon and via the MEP pathway in the plastid or the MVA pathway of cytoplasm. Furthermore, survival pressure in different environments shall ease the various metabolites of specialized complex isoprenoid-derived biosynthesis through the use of both MEP and MVA pathways (Vranová et al., 2013). Understanding the expression of genes involved in both the above pathways in different tissues shall provide a clear perspective of the process. As it has shown in Table 4, genes involved in the MEP pathway show the highest upregulations in the flower tissue, and in the contrast, most genes involved in the MVA pathway are observed in the root tissue. With regards to that, flower tissue mostly consists of plastid and chloroplast compared to the root tissue; the high level of MEP activity in the flower was not unexpected. The gene ontology results also confirm the greater activity of the genes involved in the MEP pathway in the plastid and chloroplast of the flower than in the root.

### Analyzing the expression of genes involved in the MVA pathway in the flower and the root of the *Oliveria decumbens* Vent.

Engineering the MVA pathway in plants is challenging. The key endogenous enzyme overexpression sometimes fails to elevate the yield of the end products because of the rate-limiting steps or regulatory mechanisms, and negative feedback so needs to be specified and identified in greater detail (Pütter et al., 2017). The MVA pathway’s primary reaction is catalyzed by acetyl-CoA (acetyl-coenzyme A) C-acetyltransferase, furthermore named acetoacetyl-CoA thiolase (AACT; EC2.3.1.9). This enzyme is belonging to thiolase (class II) that converts two acetyl-CoA molecules into acetoacetyl-CoA in a reversible reaction. The studies elucidated that mutation in the *AACT2* gene in *A. thaliana* had a lethal embryonic phenotype, which shows that this gene has a key role in isoprenoid biosynthesis (Jin et al., 2012). The key role of *MsAACT1* in isoprenoid biosynthesis was demonstrated by overexpression analysis in *M. sativa* consequently resulting in higher thiolase activity and accumulation of squalene (Soto et al., 2011). Our findings showed that the expression of the *AACT* gene in *Oliveria decumbens* Vent. in the root tissue was higher compared to the flower tissue (3312 reads against 1868 reads). Acetyl-CoA is needed in numerous anabolic and catabolic pathways in plants and must be synthesized in every cell compartment. The study suggested that the expression of AACT in *T. brevicorniculatum* results in an increase in enzymatic activity and a higher level of squalene accumulation in the root (Pütter et al., 2017). Acetoacetyl-CoA is converted to HMG-CoA (β-hydroxy β-methylglutaryl-CoA) by HMG synthase (HMGS EC 2.3.3.10). The expression of the *HMGS* gene in the root is higher than in flowers, like the *AACT* gene (Figure 2). HMG-CoA in two reductive steps converted to MVA (mevalonic acid) by 3-hydroxy-3-methylglutaryl-CoA reductase (HMGR; EC 1.1.1.34). In the *Oliveria decumbens* Vent. too, expression of this gene in the root tissue was higher compared to the flower tissue. Entire plant proteins of HMGR binds to the ER (endoplasmic reticulum) with their catalytic site facing the cytosol (Darabi et al., 2012). In two back-to-back reactions catalyzed by phosphor-MVA kinase (PMK; EC2.7.4.2; At1g31910) and MVA kinase (MK; EC 2.7.1.36; At5g27450), MVA is later phosphorylated to MVA 5-diphosphate. PMKs and MKs are encoded by single genes in most plants. PMKs are mainly active in peroxisomes while cytosol is the chief activity site of MKs (Zheng et al., 2020). The expression of both genes was almost equal in the flower and the root of *Oliveria decumbens* Vent. MVK (mevalonate kinase) is considered a potential regulatory enzyme in the isoprenoid biosynthesis pathway (Lluch et al., 2000). Evaluation and analysis by the Northern blot technique confirmed that the mRNA of this gene prefers to accumulate in inflorescences and the root of the plant (Lluch et al., 2000). In the following, the diphosphomevalonate decarboxylase enzyme (MVD EC 4.1.1.33) catalyzes the decarboxylation of mevalonate 5-diphosphate. The expression comparison of this gene in the flower and the root of *Oliveria decumbens* Vent. shows significant upregulation in the root compared to the flower. As is shown in Table 5, out of eight genes involved in the MVA biosynthesis pathway, seven genes have the highest upregulation in the root tissue, and only the *MVK* gene shows slight upregulation in the flower of the plant (around 10%). The highest upregulation is going to the HMGR gene by 4568 reads. Also, this gene with 1906 reads had the highest level of expression out of all genes in the MVA biosynthesis pathway in the flower tissue. The four end genes of the MVA pathway including PMK, MVD, PMD, and IPK, in both flower and root tissues, had the lowest expressions, compared to the genes of the beginning of the pathway. Generally, the total expression of MVA pathway genes in the root was recorded as 11,689 reads and 6457 reads for the flower, which shows that expression difference is almost double in the root compared to the flower (Table 6).

**TABLE 6:** List of genes involved in terpenoids biosynthesis and their corresponding expression in the flower and the root tissue of the *Oliveria decumbens* Vent.

Pathway	Gene name	Protein name	Enzyme code	Number of unigenes	Expression value in flower	Expression value in root
**MVA**	*AACT*	acetyl-coenzyme A (CoA) C-acetyltransferase	2.3.1.9	2	1868	3312
	*HMGS*	Hydroxymethylglutaryl-CoA synthase	2.3.3.10	3	819	1570
	*HMGR*	hydroxy-3-methylglutaryl-coenzyme A reductase	1.1.1.34	3	1906	4568
	*MVK*	Mevalonate kinase	2.7.1.36	1	1065	955
	*PMK*	Phosphomevalonate kinase	2.7.4.2	1	253	266
	*MVD*	Diphosphomevalonate decarboxylase	4.1.1.33	1	138	352
	*PMD*	Phosphomevalonate decarboxylase	4.1.1.99	1	111	232
	*IPK*	Isopentenyl phosphate kinase	2.7.4.26	2	297	434
**MEP**	*DXS*	Deoxy-d-xylulose-5-phosphate synthase	2.2.1.7	3	8924	649
	*DXR*	Deoxy-d-xylulose 5-phosphate reductoisomerase	1.1.1.267	1	1278	611
	*MCT*	2-C-Methyl-D-erythritol 4-phosphate cytidylyltransferase	2.7.7.60	1	763	808
	*CMK*	4-(Cytidine 5-diphospho)-2-*C*-methyl-D-erythritol kinase	2.7.1.148	1	512	243
	*MDS*	2-C-Methyl-D-erythritol 2,4-cyclodiphosphate synthase	4.6.1.12	1	256	122
	*HDS*	Hydroxy-3-methylbut-2-en-1-yl diphosphate synthase	1.17.7.1	2	8860	2783
	*HDR*	4-Hydroxy-3-methylbut-2-enyl diphosphate reductase	1.17.1.4	3	6059	4885
	*IDI*	Isopentenyl-diphosphate Delta-isomerase	5.3.3.2	1	668	363
**MVA and MEP**	*GPP*	Geranyl pyrophosphate synthase	2.5.1.1	2	188	243
	*FPPS*	Farnesyl pyrophosphate synthase	2.5.1.10	3	110	267
	*GGPP*	Geranylgeranyl pyrophosphate synthase	2.5.1.29	3	306	346

### Analyzing the genes involved in the MEP pathway in the root and the flower of *Oliveria decumbens* Vent.

In the beginning, the MEP pathway is known as the Rohmer or non-mevalonate pathway (Banerjee and Sharkey, 2014). The MEP pathway initiated with the synthesis of 1-deoxy-d-xylulose 5-phosphate from d-glyceraldehyde 3-phosphate and pyruvate catalyzed by 1-deoxy-d-xylulose 5-phosphate synthase (DXS; EC 2.2.1.7). In most the species of the plant, DXS is encoded by several paralogs of the gene. The entire enzymes of the MEP pathway (except DXS) in *A. thaliana* and nearly all other plants are encoded by single-copy genes (Vranová et al., 2013). Expression of DXS gene in the flower of *Oliveria decumbens* Vent. was around 13 times its expression in the root. DXP reductoisomerase (DXR; EC 1.1.1.267) is the second enzyme in the MEP pathway which uses NADPH for reduction. The expression of the *DXR* gene in the flower of the *Oliveria decumbens* Vent. found to be double its expression in the root. 2-C-Methyl-D-erythritol 4-phosphate converted to CDP-ME by methylerythritol phosphate cytidylyltransferase (MCT; EC 2.7.7.60). The expression value of this gene was almost equal in both flower and the root of the *Oliveria decumbens* Vent. The position of the hydroxyl group in CDP-ME’s C2 position is phosphorylated by 4-(cytidine 5′-diphospho)-2-C-methyl-D-erythritol kinase (CMK; EC 2.7.1.148; at2g26930). The expression of the *CMK* gene in the flower was double of the root. 2-C-Methyl-d-erythritol-2,4-cyclopyrophosphate (MEcPP) enzyme (MDS EC 4.6.1.12) catalyzed 4-CDP-2-C-methyl-D-erythritol 2-phosphate (CDP-ME2P) to 2-C-Methyl- d-erythritol-2,4-cyclopyrophosphate (MEcPP). The MDS gene shows double expression in the flower compared to the root of the plant. Later, MEcPP converted to HMBPP by 4-hydroxy-3-methylbut-2-enyl-diphosphate synthase (ferredoxin) (HDS EC 1.17.7.1), and finally, HMBPP converted to a mixture of IPP and DMAPP by 4-hydroxy-3-methylbut-2-enyl diphosphate reductase (HDR; EC 1.17.1.2). The expression of both HDS and HDR genes in the flower of *Oliveria decumbens* is more than in the root. The synthesized IPP and DMAPP in the MVA pathway are used in isoprenoid synthesis in the cytosol and the mitochondria while the IPP and DMAPP-which are synthesized by the MEP pathway-used in isoprenoid synthesized in the plastids (Berthelot et al., 2012). Conversion of DMAPP from IPP and the balance between DMAPP and the IPP are controlled by IPP-isomerase (IPPI; EC 5.3.3.2) in a reversible reaction (Berthelot et al., 2012). The RNA-seq results of the *Oliveria decumbens* Vent. showed the double difference in expression of *IPP* gene in the lower compared to the root. DMAPP is further used for the synthesis of shorter chains prenyl-PPs such as GPP (geranyl diphosphate), FPP, and GGPP by adding the IPP. GPP, FPP, and GGPP are the main synthesis core of all isoprenoid’s final products. GPP synthase enzyme (GPPS EC 2.5.1.1) produces the GPP as the monoterpenoid precursor. FPP synthesizes by the farnesyl pyrophosphate synthase (FPPS EC 2.5.1.10) as the main precursor for triterpenoids, sterols, sesquiterpenoids, brassinosteroids, oligoprenols, polyprenols, and dolichols for prenylation of proteins. FPPS shall bind IPP to both GPP and DMAPP as substrates. GGPP pool in plants is used for the synthesis of gibberellins, chlorophylls, diterpenoids, plastoquinone, phylloquinone, carotenoids, tocopherols, oligoprenols, abscisic acid, polyphenols, and dolichols for protein geranylgeranylation. GGPP is synthesized by the GGPP synthase (GGPPS; EC2.5.1.29), which is performed as a homodimer and utilizes all three allylic prenyl-PPs (GPP, FPP, and DMAPP) as substrate. The analysis of expression profile of involved genes in the MEP pathway of the *Oliveria decumbens* Vent. shows that the average expression of key genes in the root were 10,464 reads vs. flower’s 27,329 reads. It is absolutely clear now that unlike the root tissue, MEP is the main pathway for the synthesis of terpenes in the flower tissue of *Oliveria decumbens* Vent. of the eight genes in this pathway, seven genes had higher expression in the flower tissue, except the MCT gene which is with a very little margin expressed higher in the root. The total expression of MEP and MVA genes were 22,153 reads in the root vs. 33,777 reads of the flower. It can be concluded that generally more terpenes are synthesized in flower of the *Oliveria decumbens* Vent. than the root tissue. The three genes of GPP, GGPP, and FPPS which at the end of the MEP and MVA pathways catalyze the production of precursors for the synthesis of mono, di, and triterpenoids, respectively, had higher expression levels than flowers at the root. The mean expression of these three genes was 856 reads in the root vs. 604 reads of the flower (Table 6).

### Synthesis of monoterpenoids, diterpenoids, and triterpenoids in *Oliveria decumbens* Vent.

**FIGURE 3:**
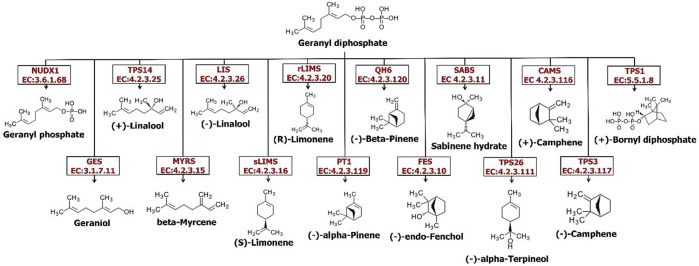
The monoterpenoids biosynthesis pathway in the *Oliveria decumbens* Vent.

The largest class of secondary metabolites of plants belong to terpenoids (terpenes). More than 36,000 distinct structures of this class have been reported so far (Dong et al., 2016). The terpene’s structure is extremely changeable and displays hundreds of different carbon skeletons. Nevertheless, this extensive structural diversity has a regular biosynthesis feature: entire terpenes are procured from the basic assembly process of 5-carbon atom isoprene units. The terpene compounds categories consist of one (C5 hemiterpenes), two isoprene units (C10 monoterpenes), three isoprene units (C15 sesquiterpenes), four isoprene units (C20 diterpenes), six isoprene units (C30 triterpenes), eight isoprene units (C40 tetraterpenes) or even more units namely called polyterpenes (>C40) (Dong et al., 2016). Limonene and its active serum-oxygenated metabolite derivatives, dihydroperillic acid and perillic acid, have been shown antiproliferative properties through their chemopreventive actions during the promotion stage of liver and breast carcinogenesis (Cox-Georgian et al., 2019). This is because of suppression of tumor cell proliferation, increase in tumor cell death rate, and/or induction of tumor cell differentiation (Cox-Georgian et al., 2019). As it is shown in Figure 3, a wide range of monoterpenes are formed by numerous enzymes in the GDP substrate in plants. Till now, a great number of genes encoding the catalyzing enzymes in monoterpenes biosynthesis in plants have been cloned; for example, linalool synthase (Jia et al., 1999), (-)-limonene synthase (Bohlmann et al., 1997), (+)-limonene synthase (Maruyama et al., 2002), myrcene synthase (Fischbach et al., 2001), (-)-camphene synthase, (-)-β-phellandrene synthase, (-)-limonene/(-)-α-pinene synthase, and terpinolene synthase (Bohlmann et al., 1999). It seems that enzymes leading to the production of primary monoterpenes skeletons appear to be active in plastids because all of the genes in this pathway that have been cloned to date have plastid target signals located in parenchymal cell chloroplasts and secretory cell leucoplasts (Aharoni et al., 2005). Comparison of the expression of the main genes involved in monoterpenoid synthesis in the root and the flower of the *Oliveria decumbens* Vent. shows a higher expression of all the genes except one gene in the flower tissue compared to the root tissue. The *geranyl diphosphate phosphohydrolase* gene is present in free monoterpenes alcohols biosynthesis in the cytosolic pathway that promotes the fragrance of the aromatic plants. This process lacks the activity of terpene synthase but shows the diphosphohydrolase function with farnesyl diphosphate and geranyl diphosphate as substrates (Magnard et al., 2015). Table 7 shows the expression of several genes for the synthesis of mono, di, and triterpenoids. Of the 23 genes identified in *Oliveria decumbens* Vent. transcriptome, 19 genes showed higher expression in the flower tissue. In contrast, three genes in the root sample showed the higher expression, namely geranyl diphosphate phosphohydrolase, geranyllinalool synthase, and ent-kaurene synthase. The expression value of the *Vetispiradiene synthase* gene was equal in both root and flower tissues of the *Oliveria decumbens*. Most of the genes showed only one homolog in the transcriptome of *Oliveria decumbens*. But the *Germacrene D synthase* gene had seven homologs, which could be due to the high similarity of the sequence of other genes in the same family, such as *Germacrene-A synthase* (EC: 4.2.3.23), *Germacrene-B synthase* (EC: 4.2.3.71), *Germacrene-C synthase* (EC: 4.2.3.60), *Germacrene A oxidase* (EC: 1.14.14.95) and *germacradienol/geosmin synthase* (EC: 4.2.3.22). As there was no sequence in the NCBI database for any of these genes in the Apiaceae, it was not possible to differentiate them by sequencing. The total expression of the main genes in the synthesis of mono, di, and triterpenoids in the flower tissue was 25,511 reads vs. just 3753 reads for the root tissue of the *Oliveria decumbens*. These findings clearly show that the main site for the synthesis of these compounds is the flower with a huge gap compared to the root. Meanwhile, three genes of (*R*)*-limonene synthase, (S)-limonene synthase,* and *beta-bisabolene synthase* are expressed only in the flower sample and had zero expression in the root tissue. Monoterpenes are important aromatic molecules that are widely distributed in nature (more than 400 structures) that shall be extracted by the perfume industry from the leaves, flowers, and fruits of many plants (Chen et al., 2003). Volatile compounds as a mixture extracted from diverse metabolite classes of plants such as terpenes, fatty acid derivatives, sulfur or nitrogen-containing compounds, and phenylpropanoids have been shown to attract different insects (even some mammals) which are pollen vectors (Dudareva and Pichersky, 2000). In a study to analyse the volatile metabolites secreted from the vegetative parts and flowers of Arabidopsis by headspace sampling method. The result showed that a mixture of volatile terpenes is emitted particularly from inflorescences and distinguished the majority of plentiful monoterpenes including β-myrcene, linalool, and limonene involved in the biosynthesis. It is discovered that most of the sesquiterpenes such as (+)-thujopsene, (-)-(E)-β-caryophyllene, (E)-β-farnesene, α-humulene, (-)-cuparene, and (+)-β-chamigrene were solely emitted from the flowers (Chen et al., 2003). Also, a comparison of *limonene synthase* gene expression showed that this gene is expressed exclusively in the flower of *Oliveria decumbens* Vent. Diterpenoids are defined as molecules deriving from the condensation of four isoprenyl units and containing 20 carbon atoms. Like terpenoids, they are ubiquitous throughout entire plants and many of them biosynthesized from geranylgeranyl diphosphate that consequently creates phytanes (acyclic), halimanes, clerodanes and labdanes (bicyclic), abietanes, rosanes, podocarpanes, pimaranes, cassanes, vouacapanes (tricyclic), kauranes, stemodanes, atisanes, trachylobanes, aphidicolanes, stemaranes, gibberallanes (tetracyclic), and taxanes, daphnanes, cembranes, ingenanes and tiglianes (macrocyclic diterpenes) based on their cyclization (Malla et al., 2019). While biosynthesis of diterpenes has been substantially studied in fungi and plants as well, bacteria recently identified for their unique production of diterpenoids and are probably to keep an underexplored reservoir of new DTSs (Smanski et al., 2012). Diterpenoids belong to the abietane and pimarane series. Along with aromatized nor-abietane diterpenoids, they have been determined for showing anti-tubercular activity (Ulubelen et al., 1997). Triterpenoid saponins are synthesized through the isoprenoid pathway via 2,3-oxidosqualene cyclization to offer dammarane triterpenoid or oleananethe (beta-amyrin) skeletons. The backbone of triterpenoid later undergoes numerous modifications such as glycosylation, substitution, and oxidation, detected by infrared with glycosyltransferases, cytochrome P450-dependent monooxygenases, and other enzymes (Haralampidis et al., 2002). Simple triterpenes are parts of specialized membranes and surface waxes and shall feasibly function as signaling molecules, where saponins (complex glycosylated triterpenes) furnish protection against pests and pathogens. Conjugated triterpenes or even simple ones have a wide range of uses in the health, biotechnology, and food sectors (Thimmappa et al., 2014). With regards to that the *Oliveria decumbens* plant has not been sequenced so far and the present study is the first transcriptome sequencing of this kind, finding the sequence of the main genes for the terpenoid synthesis requires doing the BLAST with genes at NCBI database on transcriptome of *Oliveria decumbens* plant. Alignment of already deposited genes for taxonomically remote families had no results. Therefore, inevitably the terpene synthetase genes sequence BLAST against Apiaceae family.

**TABLE 7:** List of key genes involved in mono, di and triterpenoids biosynthesis pathway and their expression value in the flower and the root of the *Oliveria decumbens* Vent.

Gene name	Protein name	Enzyme code	Number of unigenes	Expression value in flower	Expression value in root
*NUDX1*	Geranyl diphosphate phosphohydrolase	3.6.1.68	3	256	548
*GES*	Geraniol synthase	3.1.7.11	1	502	132
*TPS14*	(S)-linalool synthase	4.2.3.25	2	164	22
*MYRS*	Myrcene/ocimene synthase	4.2.3.15	1	12,206	31
*LIS*	(R)-linalool synthase	4.2.3.26	1	127	116
*sLIMS*	(S)-limonene synthase	4.2.3.16	1	344	0
*rLIMS*	(R)-limonene synthase	4.2.3.20	1	605	0
*PTS*	(-)-Alpha-pinene/(-)-camphene synthase	4.2.3.119	1	375	16
*QH6*	(-)-Beta-pinene synthase	4.2.3.120	1	3609	39
*FES*	(-)-Endo-fenchol synthase	4.2.3.10	1	256	122
*SABS*	Sabinene-hydrate synthase	4.2.3.11	1	3006	78
*TPS26*	(-)-Alpha-terpineol synthase	4.2.3.111	1	137	4
*CAMS*	(+)-Camphene synthase	4.2.3.116	1	138	23
*TPS3*	(-)-Camphene synthase	4.2.3.117	1	668	363
*TPS1*	(+)-Bornyl diphosphate synthase	5.5.1.8	1	188	43
*CPS1*	Ent-copalyl diphosphate synthase	5.5.1.13	3	319	157
*KS1*	Ent-kaurene synthase	4.2.3.19	1	75	125
*GLS*	Geranyllinalool synthase	4.2.3.144	1	340	389
*GERDS*	Germacrene D synthase	4.2.3.75	7	348	304
*PVS1*	Vetispiradiene synthase	4.2.3.21	1	93	93
*TPS1*	Beta-bisabolene synthase	4.2.3.55	1	285	0
*CAS1*	Cycloartenol synthase	5.4.99.8	1	507	450
*DDS*	Dammarenediol II synthase	4.2.1.125	1	963	698

Table 7 shows the expression of some terpene synthetase genes that can identify the relevant genes on the transcriptome of *Oliveria decumbens* Vent. by alignment. As can be seen, the expression of these genes in the flower of the *Oliveria decumbens* is much higher than in the root of the plant. In Persian traditional medicine, more emphasis has been placed on the medicinal effects of the *Oliveria decumbens* flower (Eftekhari et al., 2019; Khodavirdipour et al., 2021). Undoubtedly, however, a significant portion of terpenoids is transported between plant tissues in various ways after synthesis. The terpenoid transportation shall happen in at least three ways: transport in host cells, transport across the cell membrane, and intracellular transport (Fang and Xiao, 2021). Terpenoids are usually extracted from different parts of plants such as roots, leaves, or fruits. It must be considered that many of them were accumulated in the cuticular wax of different tissues of plants (Szakiel et al., 2012; Pensec et al., 2016; Pina et al., 2016). The cuticular wax consists of an epicuticular layer on the surface and an intracuticular layer implanted in the cutin matrix. The triterpenoid contents in the intracuticular layer are sometimes higher than in the epicuticular layer. A realizable elucidation for the triterpenoid’s uneven distribution in the cuticular wax tissue is the separation of the liquid-liquid phase since the long-chain compounds such as cyclic triterpenoids exhibit different conformation and molecular polarities (Buschhaus and Jetter, 2011). The following 67 genes (A/c numbers) including genes involved in terpenoids biosynthesis which are so far deposited to the NCBI are: MK392541, MK392542, MK392543, MK392544, MK392545, MN398148, MN398149, MN398150, MN398152, MN398153, MT002142, MT002143, MT002144, MT002145, MN954564, MN954565, MN937350, MN937351, MN937352, MN907431, MN900853, MN900854, MN900855, MN894141, MN894142, MN894143, MN894144, MN893226, MN891826, MN882159, MN882160, MN882161, MN864743, MN861110, MN861111, MN848166, MN818813, MW435622, MW435623, MW447134, MW447135, MW447136, MW447137, MW447138, MW447139, MW447140, MW447141, MW447142, MW447143, MW447144, MW447145, MW447146, MT920432, MZ620662, MZ620663, MZ620664, MZ620665, MZ620666, MZ620667, MZ620668, MZ620669, MZ620670, MZ620671, MZ620672, MZ620673, MZ620674, MZ620675 (Table 8).

**TABLE 8:** List of sequences identified in *Oliveria decumbens* in this research so far and deposited to the NCBI database.

Accession number	Gene name
MK392541	Gamma-terpinene synthase mRNA, partial cds
MK392542	Terpene synthase mRNA, partial cds
MK392543	1-Deoxy-d-xylulose 5-phosphate reductoisomerase mRNA, partial cds
MK392544	Cytochrome P450 mRNA, partial cds
MK392545	(R)-limonene synthase mRNA, partial cds
MN398148	E3 ubiquitin-protein ligase UPL 2-like mRNA, complete cds
MN398149	Midasin mRNA, complete cds
MN398150	Putative magnesium transporter NIPAB mRNA, complete cds
MN398152	Protein decapping 5 mRNA, complete cds
MN398153	RRP12-like protein mRNA, complete cds
MT002142	Protein cathepsin B mRNA, complete cds
MT002143	Protein indole-3-acetic acid-amido synthetase GH3.6 mRNA, partial cds
MT002144	Protein DNA-directed RNA polymerase II subunit 1 mRNA, partial cds
MT002145	Hypothetical protein mRNA, complete cds
MN954564	Hypothetical protein mRNA, complete cds
MN954565	Protein guanylate kinase 2 (GK-2) mRNA, partial cds
MN937350	NADH-plastoquinone oxidoreductase subunit 5 protein (ndhF) mRNA, complete cds
MN937351	Curvature thylakoid 1A protein (CURT1A) mRNA, complete cds
MN937352	PIN-LIKES 3-like protein (PILS3) mRNA, partial cds
MN907431	Cytochrome P450 71D9-like protein mRNA, partial cds
MN900853	Strictosidine synthase-like 10-like protein mRNA, complete cds
MN900854	ATP synthase CF1 alpha subunit protein mRNA, complete cds
MN900855	photosystem II 47 kDa protein mRNA, complete cds
MN894141	Two-component response regulator-like APRR7 protein mRNA, complete cds
MN894142	CRIB domain-containing protein RIC6-like protein mRNA, partial cds
MN894143	Exportin 1A protein mRNA, complete cds
MN894144	maturase protein mRNA, partial cds
MN893226	Cytochrome P450 78A6-like protein mRNA, partial cds
MN891826	WRKY transcription factor 7 protein mRNA, complete cds
MN882159	Non-specific lipid-transfer protein mRNA, complete cds
MN882160	Hypothetical protein mRNA, complete cds
MN882161	Hypothetical protein mRNA, complete cds
MN864743	Non-functional NADPH-dependent codeinone reductase 2-like protein mRNA, complete cds
MN861110	ATP-dependent helicase BRM-like protein mRNA, complete cds
MN861111	Heat shock cognate 70 kDa protein 2 mRNA, complete cds
MN848166	Chloroplast cytochrome P450 monooxygenase 97A3 mRNA, complete cds; nuclear gene for chloroplast product
MN818813	BTB/POZ domain-containing protein mRNA, complete cds
MW435622	Acetyl-CoA acetyltransferase cytosolic 1 (AAT1) mRNA, partial cds
MW435623	Dammarenediol II synthase protein (DDS) mRNA, partial cds
MW447134	Cycloartenol synthase (CAS1) mRNA, partial cds
MW447135	Dammarenediol II synthase protein transcript variant X2 (DDS) mRNA, partial cds
MW447136	Dammarenediol II synthase protein transcript variant X3 (DDS) mRNA, partial cds
MW447137	Farnesyl pyrophosphate synthase 1 (FPS1) mRNA, partial cds
MW447138	3-Hydroxy-3-methylglutaryl coenzyme A reductase (HMGCR) mRNA, partial cds
MW447139	Hydroxymethylglutaryl-CoA synthase (HMGCS1) mRNA, partial cds
MW447140	Diphosphomevalonate decarboxylase (MVD2) mRNA, complete cds
MW447141	Mevalonate kinase (MVK) mRNA, partial cds
MW447142	Phosphomevalonate kinase (PMVK) mRNA, partial cds
MW447143	Squalene synthase (FDFT1) mRNA, partial cds
MW447144	Squalene monooxygenase transcript variant x1 (SQLE) mRNA, partial cds
MW447145	Squalene monooxygenase transcript variant x2 (SQLE) mRNA, partial cds
MW447146	Squalene monooxygenase transcript variant x3 (SQLE) mRNA, partial cds
MT920432	ATP-dependent zinc metalloprotease FTSH 7 mRNA, partial cds
MZ620662	*Oliveria decumbens* 1-deoxy-d-xylulose-5-phosphate synthase (DXS) mRNA, partial cds
MZ620663	*Oliveria decumbens* enolase 1 (ENO1) mRNA, partial cds
MZ620664	*Oliveria decumbens* fructose-bisphosphate aldolase 1 (FBA1) mRNA, partial cds
MZ620665	*Oliveria decumbens* alcohol dehydrogenase 1 (ADH1) mRNA, complete cds
MZ620666	*Oliveria decumbens* solanesyl diphosphate synthase 1 (SPS1) mRNA, complete cds
MZ620667	*Oliveria decumbens* 4-hydroxy-3-methylbut-2-en-1-yl diphosphate synthase (ISPG) mRNA, complete cds
MZ620668	*Oliveria decumbens* 3-hydroxy-3-methylglutaryl-coenzyme A reductase (HMGCR) mRNA, complete cds
MZ620669	*Oliveria decumbens* hydroxymethylglutaryl-CoA synthase (HMGCS1) mRNA, partial cds
MZ620670	*Oliveria decumbens* isopentenyl-diphosphate Delta-isomerase I (IPP1) mRNA, complete cds
MZ620671	*Oliveria decumbens* 4-hydroxy-3-methylbut-2-enyl diphosphate reductase (ispH) mRNA, partial cds
MZ620672	*Oliveria decumbens* phosphoglycerate kinase 3 (PGK3) mRNA, complete cds
MZ620673	*Oliveria decumbens* pyruvate kinase (At4g26390) mRNA, complete cds
MZ620674	*Oliveria decumbens* phosphomevalonate kinase (PMVK) mRNA, partial cds
MZ620675	*Oliveria decumbens* gamma-terpinene synthase mRNA, partial cds

### Comparison of the expression network of upregulated genes in the roots and flowers of *Oliveria decumbens*

**FIGURE 4:**
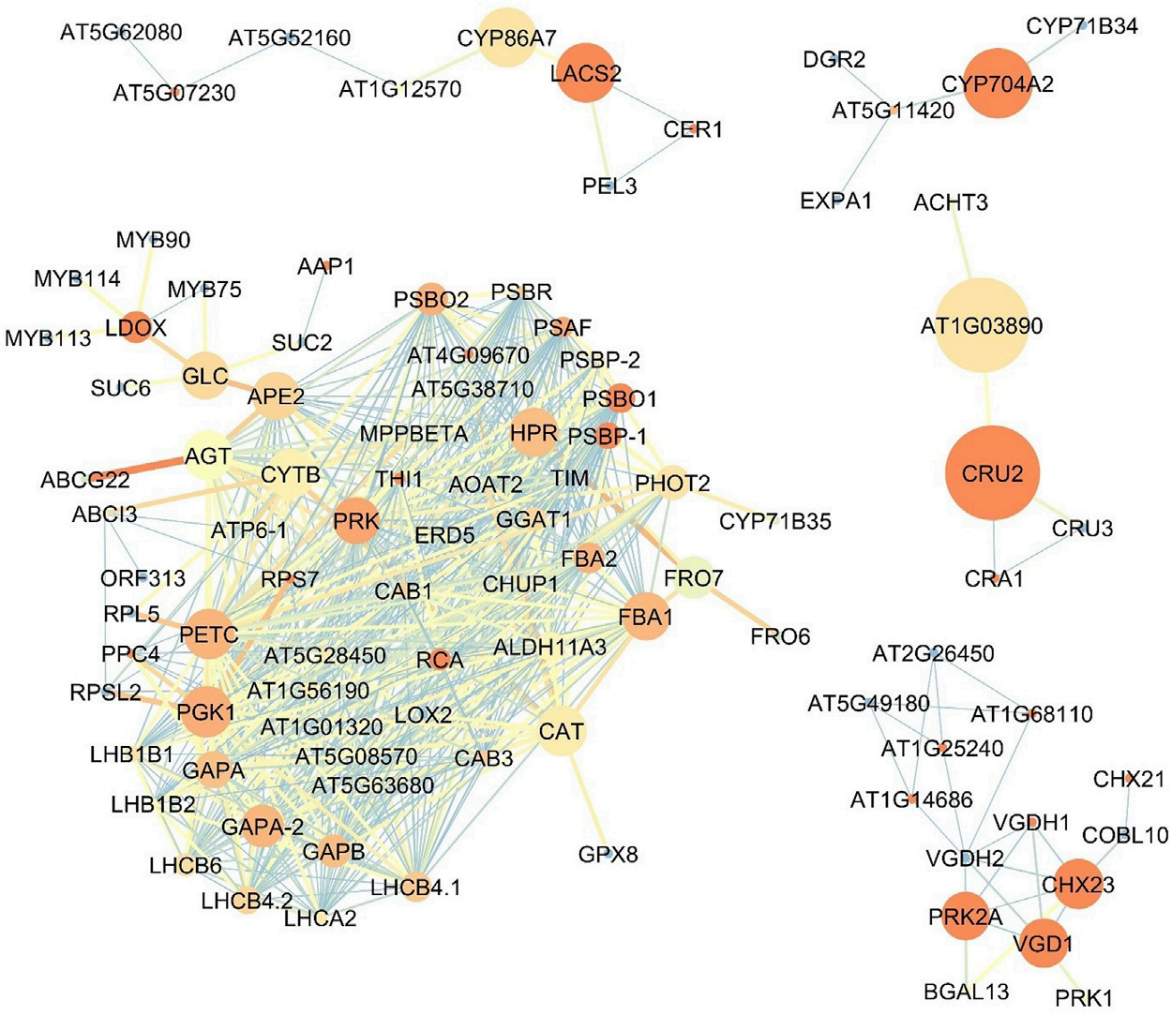
The co-expression network of upregulated genes in the flower of *Oliveria decumbens* Vent. The bigger size of the node is representative of higher betweenness centrality and the darker the red color is representative the higher the Closeness centrality and blueish color showing the lower closeness centrality in the network. Also, the thicker and more reddish connective line, represents the higher Edge Betweenness; the thinner and tending to light blue color showing the lower edge centrality.

**FIGURE 5:**
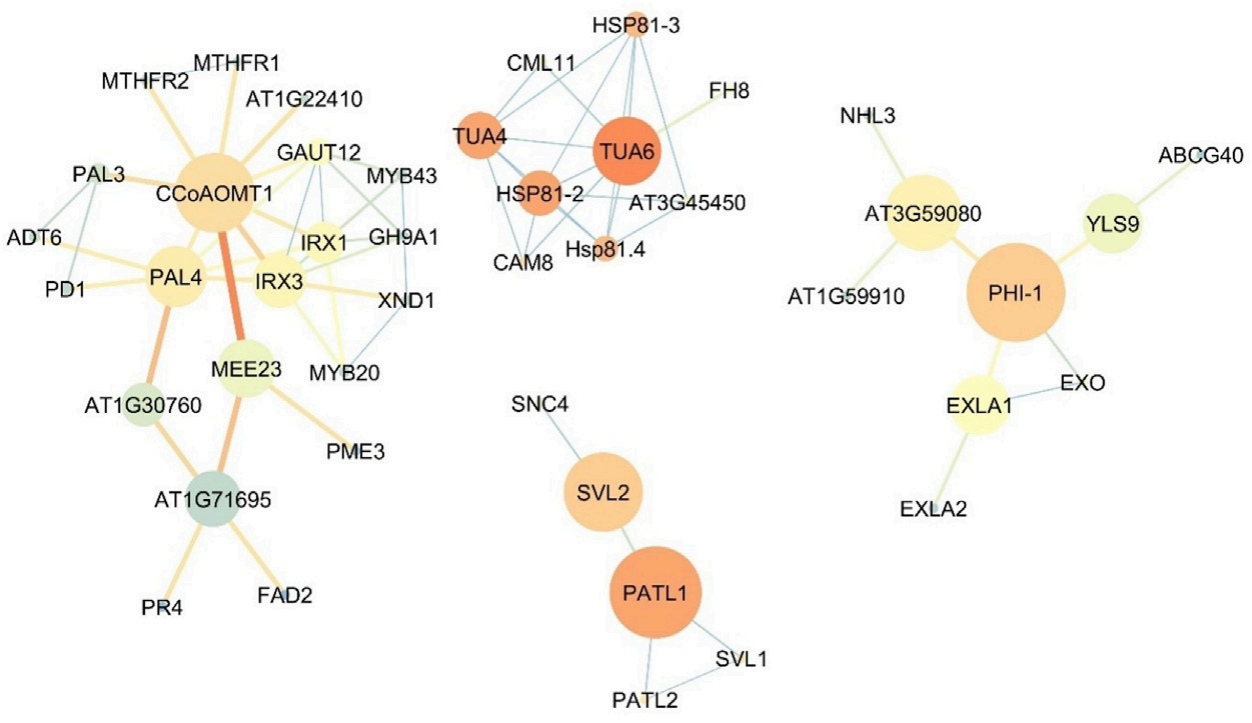
The co-expression network of upregulated genes in the root of *Oliveria decumbens* Vent. The bigger size of the node is representative of higher betweenness centrality and the darker the red color is representative the higher the Closeness centrality and blueish color showing the lower closeness centrality in the network. Also, the thicker connective line and more reddish represents the higher Edge Betweenness and thinner and tending to light blue color showing the lower edge centrality.

The bigger size of the node is representative of higher betweenness centrality and its the exact term in bioinformatics analysis the Closeness centrality and blueish color showing the lower closeness centrality in the network. Also, the thicker and more reddish connective line, represents the higher Edge Betweenness; the thinner and tending to light blue color showing the lower edge centrality. Figure 4 shows the expression network of upregulated genes in the flowers of the *Oliveria decumbens*. Topology analysis of the network showed that *GAPA-2, PRK,* and *PETC* genes each with 35 connections, and *FBA1* and *GAPA* genes each with 34 connections had the highest number of connections with other genes in the network. The two elements of closeness centrality and betweenness centrality are very useful in analyzing network topology. The centrality of a node within a complex network is called betweenness centrality, which is calculated based on the shortest communication lines between the nodes. In contrast, the term closeness centrality is the shortest distance from one node to other nodes (Shiri et al., 2018; 2020). In other words, the closer the amount of these two components are to each other, the more important that gene is in the network. Based on the closeness centrality component, 23 genes recorded the highest value, which is the same as score one, some of which are: *PSBP-1, RCA, PSBO1,* and *THI1*. Also, the highest betweenness centrality values for *CRU2*, *AT1G03890*, *CYP704A2*, *LACS2,* and *CYP86A7* genes with the following values 0.166667, 0.166667, 0.083333, 0.047619, and 0.047619 recorded, respectively (Supplementary Material S4). For genes expressed in the flowers, one main network and four sub-networks were observed. GAPA-2 genes are involved in the Calvin-Benson cycle (photosynthetic reductive pentose phosphate pathway). This gene by NADPH catalyzes the reduction of 1,3-diphophoglycerate (Li et al., 2019). The studies show that this gene has a key role in responding to abiotic stress in wheat, and this stress resistance process shall be complemented by the H2O2-mediated ABA signaling pathway (Li et al., 2019). PRK regulates the germination of pollen and growth of pollen tube polar (Chang et al., 2013). The product of the *PETC* gene is a vital and key protein for phototrophy with the help of heat dissipation of light energy and luminal acidification (increase of PH gradient) resistance to photo-oxidative damage. This product belongs to the cytochrome b6-f complex—which facilitates the electron transfer between photosystem I (PSI) and photosystem II (PSII)—and is responsible for state transfer and circular electron flow around the PSI (Munekage et al., 2001; Jahns et al., 2002; Maiwald et al., 2003). Fructose-1,6-biphosphate aldolase (FBA) in chloroplast catalyzes the development of both sedoheptulose 1,7-bisphosphate and fructose 1,6-bisphosphate within the Calvin-Benson cycle (Carrera et al., 2021). While the expression of the *FBA1* gene is most important in the aerial part of the plant, *FBA3*, its homolog, is expressed in the root (Carrera et al., 2021). PSBO1 and PSBP1 genes play a role in the photosystem II regulatory processes (Yi et al., 2005). One of the essential enzymes for the photosynthesis is *RCA* gene product named Rubisco activase. This enzyme removes the inhibitory sugar phosphates from the Rubisco’s active site, a necessary process for carbon fixation and activation of Rubisco (Nagarajan and Gill, 2018). *THI1* gene has a key role in thiamine precursor thiazole biosynthesis. Catalyzes the conversion glycine and NAD to ADT (adenosine diphosphate 5-(2-hydroxyethyl)-4-methyl thiazole-2-carboxylic acid, an adenylated thiazole intermediate (Li et al., 2016). Analyzing the role of the other genes in the upregulated gene expression in the flower demonstrated that the most of genes are involved in the photosynthesis process, responses to biological and non-biological stresses, and also specific genes for the development of the flower tissue and synthesis of the fatty acids. Figure 5 shows the upregulated gene expression network in the root. 44 genes construct the four-gene network of the root. Analyzing the network topology elucidated that *CCoAOMT1, IRX3, PAL4, TUA6,* and *IRX1* had the highest number of connections, respectively, with 9, 8, 7, 7, and 7 with other genes in the network. Based on the closeness centrality factor, *TUA6, HSP81-2, TUA4, PATL1,* and *Hsp81.4* genes, respectively, expressed with 0.888889, 0.8, 0.8, 0.8 and 0.727273 values. Also in terms of the betweenness centrality component, *PHI-1, PATL1, CCOAOMT1,* and *AT3G59080* genes had the highest values with 0.75, 0.666667, 0.50614, 0.5, and 0.46286 respectively (Supplementary Material S5). CCoAOMT1 (caffeoyl coenzyme A-dependent O-methyltransferase 1) is known to be one of the main OMTs (methyltransferases) in the lignin monomers biosynthesis, sinapoylalcohol. The CCoAOMT1 is exclusively and strongly expressed in the roots and stems of vascular tissues (Do et al., 2007; Kai et al., 2008; Fellenberg et al., 2012). *IRX1* and *3* genes are involved in cellulose biosynthesis. In higher plants, cellulose is synthesized by a multi-enzyme complex comprising multiple catalytic subunits and other proteins at the plasma membrane. In IRX3, synthesis of cellulose is deficient in the thickened secondary cell walls of interfascicular tissues and xylem in the stem (Ha et al., 2002). The product of the *PAL4* gene is a crucial and essential enzyme in plant metabolism catalyzing the preliminary reaction in the biosynthesis of a wide variety of natural products based on the phenylpropane skeleton from l-phenylalanine (Cochrane et al., 2004). *TUA4* and *TUA6* are key genes involved in tubulin synthesis in plants. Tubulin is the microtubule’s fundamental constituent, and it is bound to two GTP moles, one at a non-exchangeable site on the alpha chain and one at an exchangeable site on the beta chain. The mutant studies of these genes in Arabidopsis demonstrated that despite the detection of no obvious abnormalities in aerial parts of plants, root growth was seriously affected. The root tip cells contained abnormal microtubule structure, atypical proliferation, and the impracticality of normal cell division. These cellular defects may cause significant radial expansion of the root tip and also prevent elongation of the root (Bao et al., 2001; Matsumoto et al., 2010). *Hsp81.4* and *HSP81-2* are genes of molecular chaperones that boost structural maintenance, maturation, and specific target protein’s proper regulation that is involved in signal transduction and cell cycle control. It undergoes a functional cycle that is linked to its ATPase activity. The said cycle is likely to induce client proteins conformational changes, by that means promoting their activation. This functional cycle interconnect interactively with different co-chaperones that regulate its recognition of substrate, chaperone function, and ATPase cycle (Schenck et al., 2013). It seems that PHI-1 has a key function in a brassinosteroid-dependent regulatory pathway that manages growth and development under low energy and carbon availability (Schröder et al., 2011). The PATL1 gene binds to specific phosphoinositides that are important regulators of membrane transport. This gene usually binds to phosphoinositides (5)P, phosphoinositides (4,5)P_2_, and phosphoinositides P_3_. Studies show the role of PATL1in the membrane transfer events associated with cell plate proliferation or maturation, hinting at the involvement of phosphoinositides in cell plate biogenesis (Peterman et al., 2004). In general, it can be concluded that the genes of expression network of *Oliveria decumbens* root are mostly active in the processes of root growth and development, responses to environmental stresses, and biosynthesis of compounds such as l-phenylalanine.

### Validation

**FIGURE 6:**
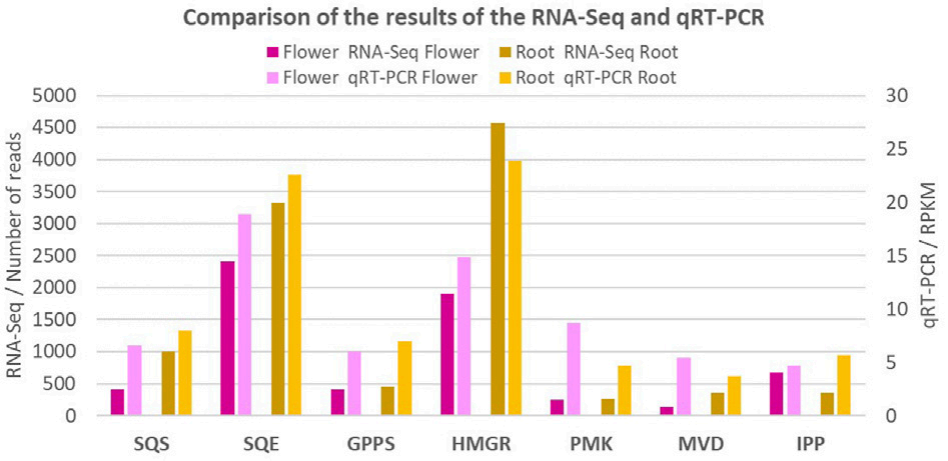
The comparison of expression value of the seven key genes in terpenoids biosynthesis by qRT-PCR. The comparison showing that the expression value of all seven genes are in harmony with all other genes of the study and their corresponding expressions.

The qRT-PCR method was used to validate the expression of seven genes involved in the terpenoid synthesis in the *Oliveria decumbens*. As it is shown in Figure 6, the expression of all seven genes is highly consistent with the expression of the other genes and their expression from the RNA-Seq results. It is important to consider that the slight difference in obtained expression values from the qRT-PCR method with the RNA-Seq results is negligible due to different parameters such as the primers efficiency, quality of the PCR kit, and the accuracy of the thermocycler.

## Conclusion

In this study, by producing more than 136 million reads of both samples of root and flower of the medicinal plant *Oliveria decumbens* Vent., transcriptomic differences between the tissue samples were investigated. The phytochemical analysis also confirmed the presence of the above gene products such as medicinally valuable compounds. The results showed that the MEP pathway is the most active in the flowers and the MVA pathway is the most active pathway in the root. These findings confirm that the synthesis of these compounds in the flowers of *Oliveria decumbens* has a great advantage over the root. The gene ontology analysis results showed that in general, the flower of the *Oliveria decumbens* is almost three times more active than the root tissue and has more active organs and more diverse biological pathways and molecular functions. Genes of the root are often active in the processes of root growth and development, response to environmental stresses, and the biosynthesis of compounds such as l-phenylalanine; while the genes of flower sample demonstrated that most of them are involved in the photosynthesis process, response to biological and abiotic stresses, specific genes for the growth, and development of flower organelles and synthesis of fatty acids.

## Data Availability

The datasets presented in this study can be found in online repositories. The names of the repository/repositories and accession number(s) can be found in the article/Supplementary Material.

## References

[B1] Aharoni A. Jongsma M. A. Bouwmeester H. J. (2005). Volatile science? Metabolic engineering of terpenoids in plants. Trends Plant Sci. 10, 594–602. 10.1016/j.tplants.2005.10.005 16290212

[B2] Alizadeh Behbahani B. Tabatabaei Yazdi F. Vasiee A. Mortazavi S. A. (2018). Oliveria decumbens essential oil: Chemical compositions and antimicrobial activity against the growth of some clinical and standard strains causing infection. Microb. Pathog. 114, 449–452. 10.1016/j.micpath.2017.12.033 29241765

[B3] Ashraf M. A. (20202020). Phytochemicals as potential anticancer drugs: Time to ponder nature’s bounty. Biomed. Res. Int. 2020, 8602879. 10.1155/2020/8602879 PMC7013350 32076618

[B4] Ashraf M. A. Sayed S. Bello M. Hussain N. Chando R. K. Alam S. (2022). CDK4 as a phytochemical based anticancer drug target. Inf. Med. Unlocked 28, 100826. 10.1016/j.imu.2021.100826

[B5] Assenov Y. Ramirez F. Schelhorn S. E. Lengauer T. Albrecht M. (2008). Computing topological parameters of biological networks. Bioinformatics 24, 282–284. 10.1093/bioinformatics/btm554 18006545

[B6] Banerjee A. Sharkey T. D. (2014). Methylerythritol 4-phosphate (MEP) pathway metabolic regulation. Nat. Prod. Rep. 31, 1043–1055. 10.1039/c3np70124g 24921065

[B7] Bao Y. Kost B. Chua N. H. (2001). Reduced expression of alpha-tubulin genes in *Arabidopsis thaliana* specifically affects root growth and morphology, root hair development and root gravitropism. Plant J. 28, 145–157. 10.1046/j.1365-313x.2001.01142.x 11722758

[B8] Bergman M. E. Davis B. Phillips M. A. (2019). Medically useful plant terpenoids: Biosynthesis, occurrence, and mechanism of action. Molecules 24, E3961. 10.3390/molecules24213961 31683764 PMC6864776

[B9] Berthelot K. Estevez Y. Deffieux A. Peruch F. (2012). Isopentenyl diphosphate isomerase: A checkpoint to isoprenoid biosynthesis. Biochimie 94, 1621–1634. 10.1016/j.biochi.2012.03.021 22503704

[B10] Bohlmann J. Phillips M. Ramachandiran V. Katoh S. Croteau R. (1999). cDNA cloning, characterization, and functional expression of four new monoterpene synthase members of the tpsd gene family from grand fir (abies grandis). Arch. Biochem. Biophys. 368, 232–243. 10.1006/abbi.1999.1332 10441373

[B11] Bohlmann J. Steele C. L. Croteau R. (1997). Monoterpene synthases from grand fir (abies grandis): cDNA isolation, characterization, and functional expression of myrcene synthase, (−)-(4S)-limonene synthase, and (−)-(1S, 5S)-pinene synthase. J. Biol. Chem. 272, 21784–21792. 10.1074/jbc.272.35.21784 9268308

[B12] Buschhaus C. Jetter R. (2011). Composition differences between epicuticular and intracuticular wax substructures: How do plants seal their epidermal surfaces? J. Exp. Bot. 62, 841–853. 10.1093/jxb/erq366 21193581

[B13] Carrera D. Á. George G. M. Fischer-Stettler M. Galbier F. Eicke S. Truernit E. (2021). Distinct plastid fructose bisphosphate aldolases function in photosynthetic and non-photosynthetic metabolism in Arabidopsis. J. Exp. Bot. 72, 3739–3755. 10.1093/jxb/erab099 33684221 PMC8628874

[B14] Chang F. Gu Y. Ma H. Yang Z. (2013). AtPRK2 promotes ROP1 activation via RopGEFs in the control of polarized pollen tube growth. Mol. Plant 6, 1187–1201. 10.1093/mp/sss103 23024212 PMC3888354

[B15] Chen F. Tholl D. D'auria J. C. Farooq A. Pichersky E. Gershenzon J. (2003). Biosynthesis and emission of terpenoid volatiles from Arabidopsis flowers. Plant Cell 15, 481–494. 10.1105/tpc.007989 12566586 PMC141215

[B16] Cochrane F. C. Davin L. B. Lewis N. G. (2004). The Arabidopsis phenylalanine ammonia lyase gene family: Kinetic characterization of the four PAL isoforms. Phytochemistry 65, 1557–1564. 10.1016/j.phytochem.2004.05.006 15276452

[B17] Cox-Georgian D. Ramadoss N. Dona C. Basu C. (2019). Therapeutic and medicinal uses of terpenes. Med. Plants Farm Pharm. 1, 333–359. 10.1007/978-3-030-31269-5_15

[B18] Darabi M. Izadi-Darbandi A. Masoudi-Nejad A. Naghavi M. R. Nemat-Zadeh G. (2012). Bioinformatics study of the 3-hydroxy-3-methylglotaryl-coenzyme A reductase (HMGR) gene in Gramineae. Mol. Biol. Rep. 39, 8925–8935. 10.1007/s11033-012-1761-2 22722993

[B19] Do C. T. Pollet B. Thévenin J. Sibout R. Denoue D. Barrière Y. (2007). Both caffeoyl Coenzyme A 3-O-methyltransferase 1 and caffeic acid O-methyltransferase 1 are involved in redundant functions for lignin, flavonoids and sinapoyl malate biosynthesis in Arabidopsis. Planta 226, 1117–1129. 10.1007/s00425-007-0558-3 17594112

[B20] Dong L. Jongedijk E. Bouwmeester H. Van Der Krol A. (2016). Monoterpene biosynthesis potential of plant subcellular compartments. New Phytol. 209, 679–690. 10.1111/nph.13629 26356766

[B21] Dudareva N. Pichersky E. (2000). Biochemical and molecular genetic aspects of floral scents. Plant Physiol. 122, 627–633. 10.1104/pp.122.3.627 10712525 PMC1539243

[B22] Eftekhari M. Shams Ardekani M. R. Amin M. Attar F. Akbarzadeh T. Safavi M. (2019). Oliveria decumbens, a bioactive essential oil: Chemical composition and biological activities. Iran. J. Pharm. Res. 18, 412–421. 31089376 PMC6487399

[B23] Eftekhari M. Shams Ardekani M. R. Amin M. Mansourian M. Saeedi M. Akbarzadeh T. (2021). Anti-Helicobacter pylori compounds from Oliveria decumbens vent. Through urease inhibitory *in-vitro* and in-silico studies. Iran. J. Pharm. Res. 20, 476–489. 10.22037/ijpr.2021.114485.14876 34904002 PMC8653682

[B24] Fang Y. Xiao H. (2021). The transport of triterpenoids. Biotechnol. Notes 2, 11–17. 10.1016/j.biotno.2021.03.001

[B25] Fellenberg C. Van Ohlen M. Handrick V. Vogt T. (2012). The role of CCoAOMT1 and COMT1 in Arabidopsis anthers. Planta 236, 51–61. 10.1007/s00425-011-1586-6 22258746

[B26] Fischbach R. J. Zimmer W. Schnitzler J.-P. (2001). Isolation and functional analysis of a cDNA encoding a myrcene synthase from holm oak (Quercus ilex L.). Eur. J. Biochem. 268, 5633–5638. 10.1046/j.1432-1033.2001.02519.x 11683887

[B27] Gao Z. Tian S. Hou J. Zhang Z. Yang L. Hu T. (2020). RNA-Seq based transcriptome analysis reveals the molecular mechanism of triterpenoid biosynthesis in Glycyrrhiza glabra. Bioorg. Med. Chem. Lett. 30, 127102. 10.1016/j.bmcl.2020.127102 32220349

[B28] Ha M.-A. Mackinnon I. M. Šturcová A. Apperley D. C. Mccann M. C. Turner S. R. (2002). Structure of cellulose-deficient secondary cell walls from the irx3 mutant of *Arabidopsis thaliana* . Phytochemistry 61, 7–14. 10.1016/s0031-9422(02)00199-1 12165296

[B29] Haralampidis K. Trojanowska M. Osbourn A. E. (2002). Biosynthesis of triterpenoid saponins in plants. Adv. Biochem. Eng. Biotechnol. 75, 31–49. 10.1007/3-540-44604-4_2 11783842

[B30] Hussain N. Chanda R. Abir R. A. Mou M. A. Hasan M. Ashraf M. A. (2021). Mpdb 2.0: A large scale and integrated medicinal plant database of Bangladesh. BMC Res. Notes 14, 301. 10.1186/s13104-021-05721-6 34362451 PMC8344187

[B31] Hwang H.-S. Lee H. Choi Y. E. (2015). Transcriptomic analysis of Siberian ginseng (Eleutherococcus senticosus) to discover genes involved in saponin biosynthesis. BMC Genomics 16, 180. 10.1186/s12864-015-1357-z 25888223 PMC4369101

[B32] Jahns P. Graf M. Munekage Y. Shikanai T. (2002). Single point mutation in the Rieske iron-sulfur subunit of cytochrome b6/f leads to an altered pH dependence of plastoquinol oxidation in Arabidopsis. FEBS Lett. 519, 99–102. 10.1016/s0014-5793(02)02719-9 12023025

[B33] Japelaghi R. H. Haddad R. Garoosi G. A. (2011). Rapid and efficient isolation of high quality nucleic acids from plant tissues rich in polyphenols and polysaccharides. Mol. Biotechnol. 49, 129–137. 10.1007/s12033-011-9384-8 21302150

[B34] Jayakodi M. Madheswaran M. Adhimoolam K. Perumal S. Manickam D. Kandasamy T. (2019). Transcriptomes of Indian barnyard millet and barnyardgrass reveal putative genes involved in drought adaptation and micronutrient accumulation. Acta Physiol. Plant. 41, 66. 10.1007/s11738-019-2855-4

[B35] Jia J.-W. Crock J. Lu S. Croteau R. Chen X.-Y. (1999). (3R)-Linalool synthase from artemisia annua L.: cDNA isolation, characterization, and wound induction. Arch. Biochem. Biophys. 372, 143–149. 10.1006/abbi.1999.1466 10562427

[B36] Jin H. Song Z. Nikolau B. J. (2012). Reverse genetic characterization of two paralogous acetoacetyl CoA thiolase genes in Arabidopsis reveals their importance in plant growth and development. Plant J. 70, 1015–1032. 10.1111/j.1365-313X.2012.04942.x 22332816

[B37] Kai K. Mizutani M. Kawamura N. Yamamoto R. Tamai M. Yamaguchi H. (2008). Scopoletin is biosynthesized via ortho-hydroxylation of feruloyl CoA by a 2-oxoglutarate-dependent dioxygenase in *Arabidopsis thaliana* . Plant J. 55, 989–999. 10.1111/j.1365-313X.2008.03568.x 18547395

[B38] Kal A. J. Van Zonneveld A. J. Benes V. Van Den Berg M. Koerkamp M. G. Albermann K. (1999). Dynamics of gene expression revealed by comparison of serial analysis of gene expression transcript profiles from yeast grown on two different carbon sources. Mol. Biol. Cell 10, 1859–1872. 10.1091/mbc.10.6.1859 10359602 PMC25383

[B39] Khodavirdipour A. Haddadi F. Nejad H. R. Shiri Y. Tilak V. P. (2021). *In vitro* anti-cancer activity of Oliveria decumbens vent. Extract, an endemic Persian medicinal plant, on HT-29 colorectal cancer cell line. New York: Cold Spring Harbor Laboratory.

[B40] Khoshbakht T. Karami A. Tahmasebi A. Maggi F. (2020). The variability of thymol and carvacrol contents reveals the level of antibacterial activity of the essential oils from different accessions of Oliveria decumbens. Antibiot. (Basel) 9, E409. 10.3390/antibiotics9070409 PMC7400187 32674440

[B41] Kinsella R. J. Kähäri A. Haider S. Zamora J. Proctor G. Spudich G. (2011). Ensembl BioMarts: A hub for data retrieval across taxonomic space. Database 2011, bar030. 10.1093/database/bar030 21785142 PMC3170168

[B42] Li C. Adhimoolam K. Yuan Y. Yin J. Ren R. Yang Y. (2017). Identification of candidate genes for resistance to Soybean mosaic virus strain SC3 by using fine mapping and transcriptome analyses. Crop Pasture Sci. 68, 156. 10.1071/cp16353

[B43] Li C. L. Wang M. Wu X. M. Chen D. H. Lv H. J. Shen J. L. (2016). THI1, a thiamine thiazole synthase, interacts with Ca2+-dependent protein kinase CPK33 and modulates the S-type Anion channels and stomatal closure in Arabidopsis. Plant Physiol. 170, 1090–1104. 10.1104/pp.15.01649 26662273 PMC4734576

[B44] Li X. Wei W. Li F. Zhang L. Deng X. Liu Y. (2019). The plastidial glyceraldehyde-3-phosphate dehydrogenase is critical for abiotic stress response in wheat. Int. J. Mol. Sci. 20, 1104. 10.3390/ijms20051104 PMC6429432 30836662

[B45] Liao W. Mei Z. Miao L. Liu P. Gao R. (2020). Comparative transcriptome analysis of root, stem, and leaf tissues of Entada phaseoloides reveals potential genes involved in triterpenoid saponin biosynthesis. BMC Genomics 21, 639. 10.1186/s12864-020-07056-1 32933468 PMC7493163

[B46] Livak K. J. Schmittgen T. D. (2001). Analysis of relative gene expression data using real-time quantitative PCR and the 2(-Delta Delta C(T)) Method. Methods 25, 402–408. 10.1006/meth.2001.1262 11846609

[B47] Lluch M. A. Masferrer A. Arró M. Boronat A. Ferrer A. (2000). Molecular cloning and expression analysis of the mevalonate kinase gene from *Arabidopsis thaliana* . Plant Mol. Biol. 42, 365–376. 10.1023/a:1006325630792 10794536

[B48] Ma C.-H. Gao Z.-J. Zhang J.-J. Zhang W. Shao J.-H. Hai M.-R. (2016). Candidate genes involved in the biosynthesis of triterpenoid saponins in platycodon grandiflorum identified by transcriptome analysis. Front. Plant Sci. 7, 673. 10.3389/fpls.2016.00673 27242873 PMC4871891

[B49] Maere S. Heymans K. Kuiper M. (2005). BiNGO: A Cytoscape plugin to assess overrepresentation of gene ontology categories in biological networks. Bioinformatics 21, 3448–3449. 10.1093/bioinformatics/bti551 15972284

[B50] Magnard J. L. Roccia A. Caissard J. C. Vergne P. Sun P. Hecquet R. (2015). PLANT VOLATILES. Biosynthesis of monoterpene scent compounds in roses. Science 349, 81–83. 10.1126/science.aab0696 26138978

[B51] Maiwald D. Dietzmann A. Jahns P. Pesaresi P. Joliot P. Joliot A. (2003). Knock-out of the genes coding for the Rieske protein and the ATP-synthase delta-subunit of Arabidopsis. Effects on photosynthesis, thylakoid protein composition, and nuclear chloroplast gene expression. Plant Physiol. 133, 191–202. 10.1104/pp.103.024190 12970486 PMC196597

[B52] Malla R. R. Kumari S. Deepak K. G. K. Gavara M. M. Guganavath S. Rokkam P. (2019). “Chapter 7 - terpenoids as potential targeted therapeutics of pancreatic cancer: Current advances and future directions,” in Breaking tolerance to pancreatic cancer unresponsiveness to chemotherapy. Editor Nagaraju G. P. (Academic Press), 111–116.

[B53] Maruyama T. Saeki D. Ito M. Honda G. (2002). Molecular cloning, functional expression and characterization of d-limonene synthase from Agastache rugosa. Biol. Pharm. Bull. 25, 661–665. 10.1248/bpb.25.661 12033511

[B54] Matsumoto S. Kumasaki S. Soga K. Wakabayashi K. Hashimoto T. Hoson T. (2010). Gravity-induced modifications to development in hypocotyls of Arabidopsis tubulin mutants. Plant Physiol. 152, 918–926. 10.1104/pp.109.147330 20018592 PMC2815900

[B55] Munekage Y. Takeda S. Endo T. Jahns P. Hashimoto T. Shikanai T. (2001). Cytochrome b(6)f mutation specifically affects thermal dissipation of absorbed light energy in Arabidopsis. Plant J. 28, 351–359. 10.1046/j.1365-313x.2001.01178.x 11722777

[B56] Nagarajan R. Gill K. S. (2018). Evolution of Rubisco activase gene in plants. Plant Mol. Biol. 96, 69–87. 10.1007/s11103-017-0680-y 29139059

[B57] Netala V. R. Ghosh S. B. Bobbu P. Anitha D. Tartte V. (2014). Triterpenoid saponins: A review on biosynthesis, applications and mechanism of their action. Int. J. Pharm. Pharm. Sci. 7, 24–28.

[B58] Pensec F. Szakiel A. Pączkowski C. Woźniak A. Grabarczyk M. Bertsch C. (2016). Characterization of triterpenoid profiles and triterpene synthase expression in the leaves of eight Vitis vinifera cultivars grown in the Upper Rhine Valley. J. Plant Res. 129, 499–512. 10.1007/s10265-016-0797-0 26879930

[B59] Peterman T. K. Ohol Y. M. Mcreynolds L. J. Luna E. J. (2004). Patellin1, a novel Sec14-like protein, localizes to the cell plate and binds phosphoinositides. Plant Physiol. 136, 3080–3094. 10.1104/pp.104.045369 15466235 PMC523369

[B60] Pina E. S. Silva D. B. Teixeira S. P. Coppede J. S. Furlan M. França S. C. (2016). Mevalonate-derived quinonemethide triterpenoid from *in vitro* roots of Peritassa laevigata and their localization in root tissue by MALDI imaging. Sci. Rep. 6, 22627. 10.1038/srep22627 26943243 PMC4778575

[B61] Pütter K. M. Van Deenen N. Unland K. Prüfer D. Schulze Gronover C. (2017). Isoprenoid biosynthesis in dandelion latex is enhanced by the overexpression of three key enzymes involved in the mevalonate pathway. BMC Plant Biol. 17, 88. 10.1186/s12870-017-1036-0 28532507 PMC5441070

[B62] Schenck C. A. Nadella V. Clay S. L. Lindner J. Abrams Z. Wyatt S. E. (2013). A proteomics approach identifies novel proteins involved in gravitropic signal transduction. Am. J. Bot. 100, 194–202. 10.3732/ajb.1200339 23281391

[B63] Schröder F. Lisso J. Müssig C. (2011). EXORDIUM-LIKE1 promotes growth during low carbon availability in Arabidopsis. Plant Physiol. 156, 1620–1630. 10.1104/pp.111.177204 21543728 PMC3135934

[B64] Shannon P. Markiel A. Ozier O. Baliga N. S. Wang J. T. Ramage D. (2003). Cytoscape: A software environment for integrated models of biomolecular interaction networks. Genome Res. 13, 2498–2504. 10.1101/gr.1239303 14597658 PMC403769

[B65] Shiri Y. Solouki M. Ebrahimie E. Emamjomeh A. Zahiri J. (2020). Gibberellin causes wide transcriptional modifications in the early stage of grape cluster development. Genomics 112, 820–830. 10.1016/j.ygeno.2019.05.022 31136791

[B66] Shiri Y. Solouki M. Ebrahimie E. Emamjomeh A. Zahiri J. (2018). Unraveling the transcriptional complexity of compactness in sistan grape cluster. Plant Sci. 270, 198–208. 10.1016/j.plantsci.2018.02.011 29576073

[B67] Smanski M. J. Peterson R. M. Huang S.-X. Shen B. (2012). Bacterial diterpene synthases: New opportunities for mechanistic enzymology and engineered biosynthesis. Curr. Opin. Chem. Biol. 16, 132–141. 10.1016/j.cbpa.2012.03.002 22445175 PMC3328645

[B68] Soto G. Stritzler M. Lisi C. Alleva K. Pagano M. E. Ardila F. (2011). Acetoacetyl-CoA thiolase regulates the mevalonate pathway during abiotic stress adaptation. J. Exp. Bot. 62, 5699–5711. 10.1093/jxb/err287 21908473

[B69] Sudha M. Karthikeyan A. Madhumitha B. Veera Ranjani R. Kanimoli Mathivathana M. Dhasarathan M. (2022). Dynamic transcriptome profiling of mungbean genotypes unveil the genes respond to the infection of mungbean yellow mosaic virus. Pathogens 11, 190. 10.3390/pathogens11020190 35215133 PMC8874377

[B70] Sun C. Li Y. Wu Q. Luo H. Sun Y. Song J. (2010). De novo sequencing and analysis of the American ginseng root transcriptome using a GS FLX Titanium platform to discover putative genes involved in ginsenoside biosynthesis. BMC Genomics 11, 262. 10.1186/1471-2164-11-262 20416102 PMC2873478

[B71] Szakiel A. Pączkowski C. Pensec F. Bertsch C. (2012). Fruit cuticular waxes as a source of biologically active triterpenoids. Phytochem. Rev. 11, 263–284. 10.1007/s11101-012-9241-9 23519009 PMC3601259

[B72] Szklarczyk D. Morris J. H. Cook H. Kuhn M. Wyder S. Simonovic M. (2017). The STRING database in 2017: Quality-controlled protein–protein association networks, made broadly accessible. Nucleic Acids Res. 45, D362–D368. 10.1093/nar/gkw937 27924014 PMC5210637

[B73] Takase S. Kera K. Hirao Y. Hosouchi T. Kotake Y. Nagashima Y. (2019). Identification of triterpene biosynthetic genes from Momordica charantia using RNA-seq analysis. Biosci. Biotechnol. Biochem. 83, 251–261. 10.1080/09168451.2018.1530096 30317922

[B74] Tang Q. Ma X. Mo C. Wilson I. W. Song C. Zhao H. (2011). An efficient approach to finding Siraitia grosvenorii triterpene biosynthetic genes by RNA-seq and digital gene expression analysis. BMC Genomics 12, 343. 10.1186/1471-2164-12-343 21729270 PMC3161973

[B75] Thimmappa R. Geisler K. Louveau T. O'maille P. Osbourn A. (2014). Triterpene biosynthesis in plants. Annu. Rev. Plant Biol. 65, 225–257. 10.1146/annurev-arplant-050312-120229 24498976

[B76] Ulubelen A. Topcu G. Johansson C. B. (1997). Norditerpenoids and diterpenoids from Salvia multicaulis with antituberculous activity. J. Nat. Prod. 60, 1275–1280. 10.1021/np9700681 9428161

[B77] Untergasser A. Nijveen H. Rao X. Bisseling T. Geurts R. Leunissen J. a. M. (2007). Primer3Plus, an enhanced web interface to Primer3. Nucleic Acids Res. 35, W71–W74. 10.1093/nar/gkm306 17485472 PMC1933133

[B78] Vranová E. Coman D. Gruissem W. (2013). Network analysis of the MVA and MEP pathways for isoprenoid synthesis. Annu. Rev. Plant Biol. 64, 665–700. 10.1146/annurev-arplant-050312-120116 23451776

[B79] Yang D. Du X. Liang X. Han R. Liang Z. Liu Y. (2012). Different roles of the mevalonate and methylerythritol phosphate pathways in cell growth and tanshinone production of salvia miltiorrhiza hairy roots. PLOS ONE 7, e46797. 10.1371/journal.pone.0046797 23209548 PMC3510226

[B80] Yi X. Mcchargue M. Laborde S. Frankel L. K. Bricker T. M. (2005). The manganese-stabilizing protein is required for photosystem II assembly/stability and photoautotrophy in higher plants. J. Biol. Chem. 280, 16170–16174. 10.1074/jbc.M501550200 15722336

[B81] Zheng H. Yu M. Y. Pu C. J. Chen M. L. Li F. Q. Shen Y. (2020). [Cloning and expression analysis of 5-phosphomevalonate kinase gene (CcPMK) in Cinnamomum camphora]. Zhongguo Zhong Yao Za Zhi 45, 78–84. 10.19540/j.cnki.cjcmm.20191104.104 32237414

