# Supplementary material for: Comparative de novo transcriptome analysis of flower and root of Oliveria decumbens Vent. to identify putative genes in terpenes biosynthesis pathway
Source: Front Genet. 2022 Aug 4;13:916183. doi: 10.3389/fgene.2022.916183 (PMC9386285; doi:10.3389/fgene.2022.916183)
Supplement: Supplementary file 1 [file DataSheet2.PDF]

Library Search Report

Data Path : G:\ÌÒÇÑÔ 1399\99-11-18\
Data File : 4955-osareh.D
Acq On : 9 Feb 2021 22:25
Operator :
Sample : 4955-osareh
Misc :
ALS Vial : 96 Sample Multiplier: 1

Search Libraries: E:\Database\wiley7n.l Minimum Quality: 0

Unknown Spectrum: Apex
Integration Events: ChemStation Integrator - events.e

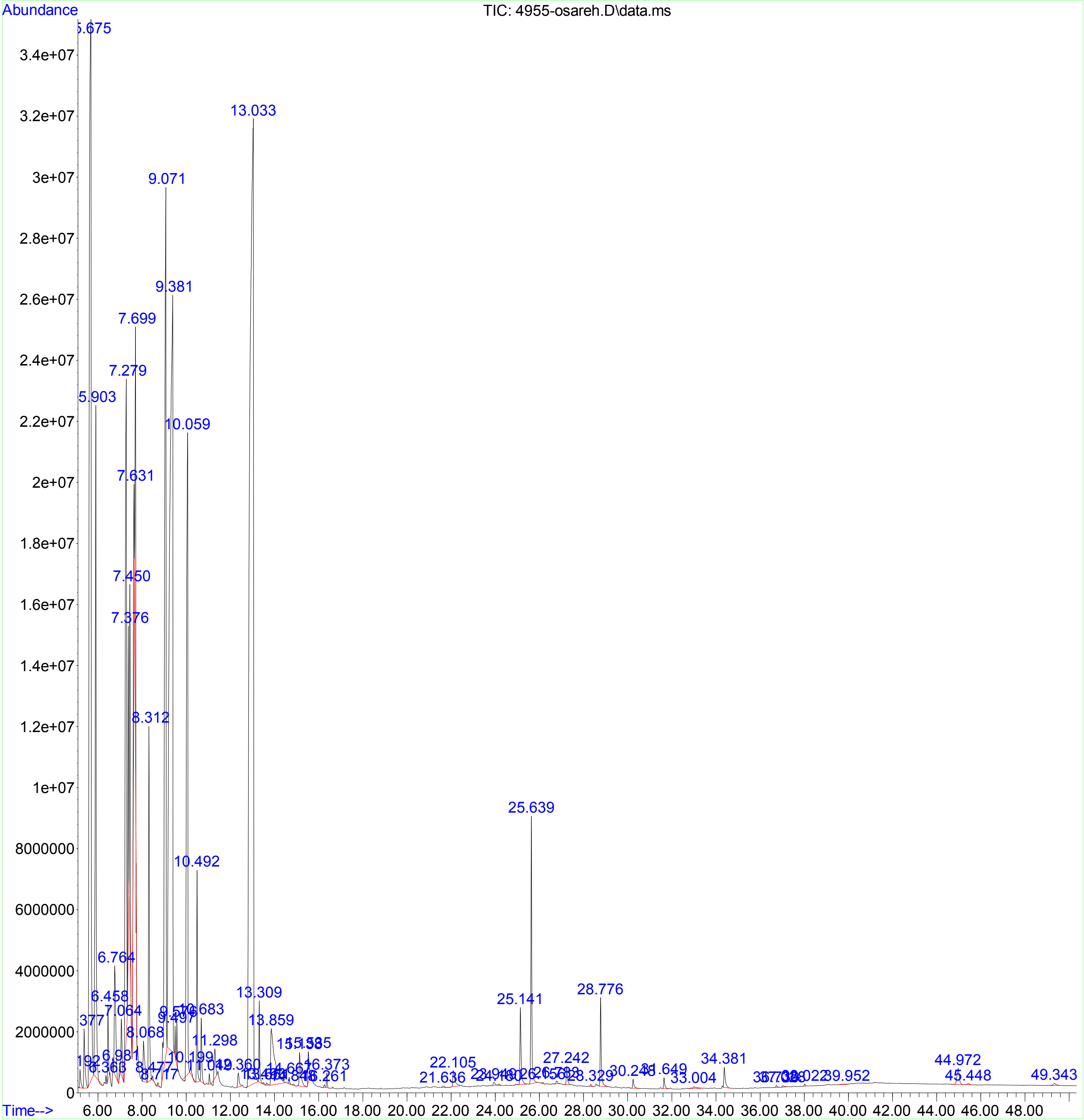

Unknown Spectrum based on Apex

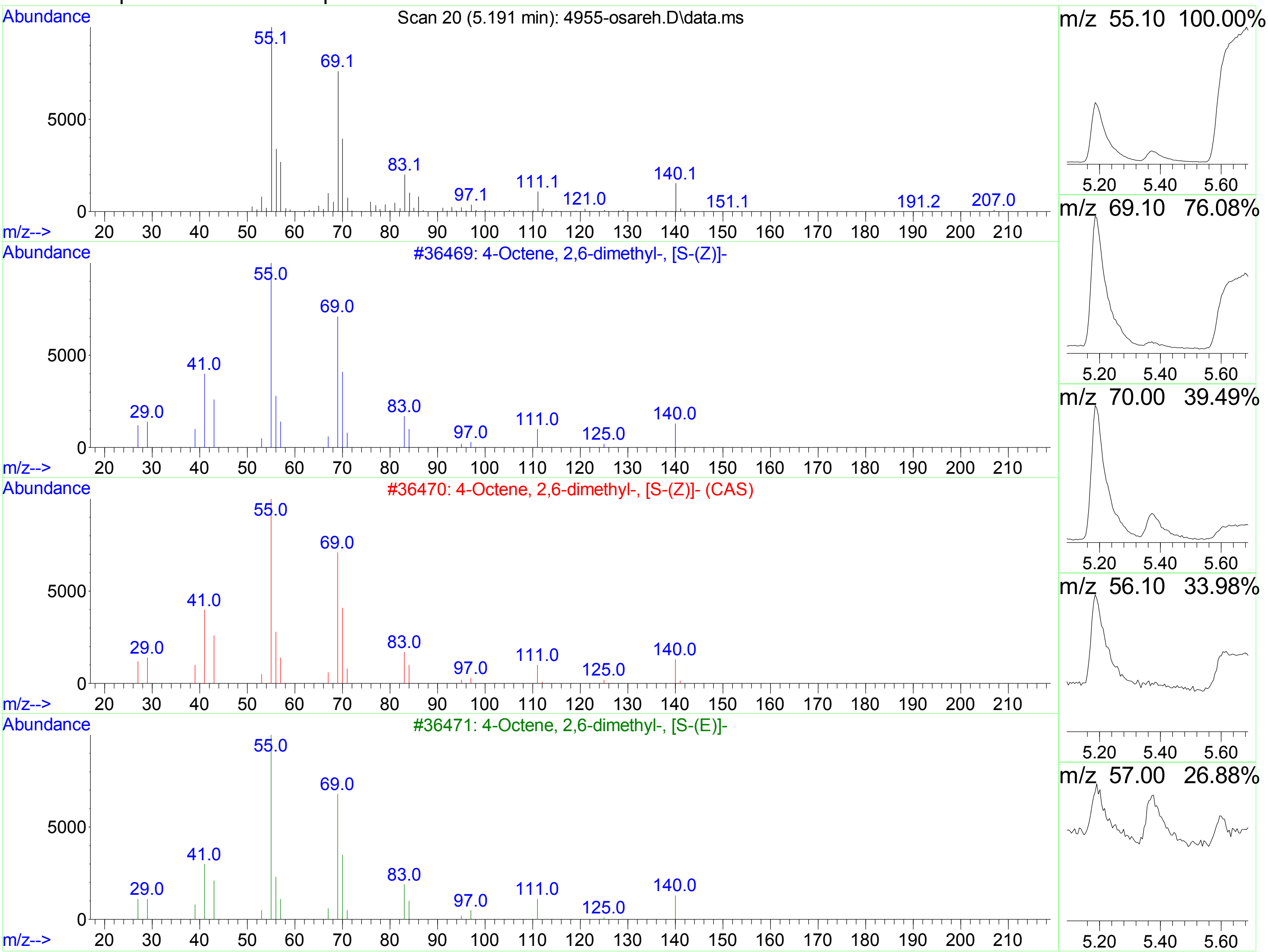

Data File: G:\ÌÒÇÑÔ 1399\99-11-18\4955-osareh.D
Sample : 4955-osareh

Peak Number: 1 at 5.191 min Area: 19449861 Area % 0.13

The 3 best hits from each library.	Ref#	CAS#	Qual
E:\Database\wiley7n.l			
1 4-Octene, 2,6-dimethyl-, [S-(Z)]-	36469	062960-77-4	94
2 4-Octene, 2,6-dimethyl-, [S-(Z)]...	36470	062960-77-4	94
3 4-Octene, 2,6-dimethyl-, [S-(E)]-	36471	062960-76-3	94

Unknown Spectrum based on Apex

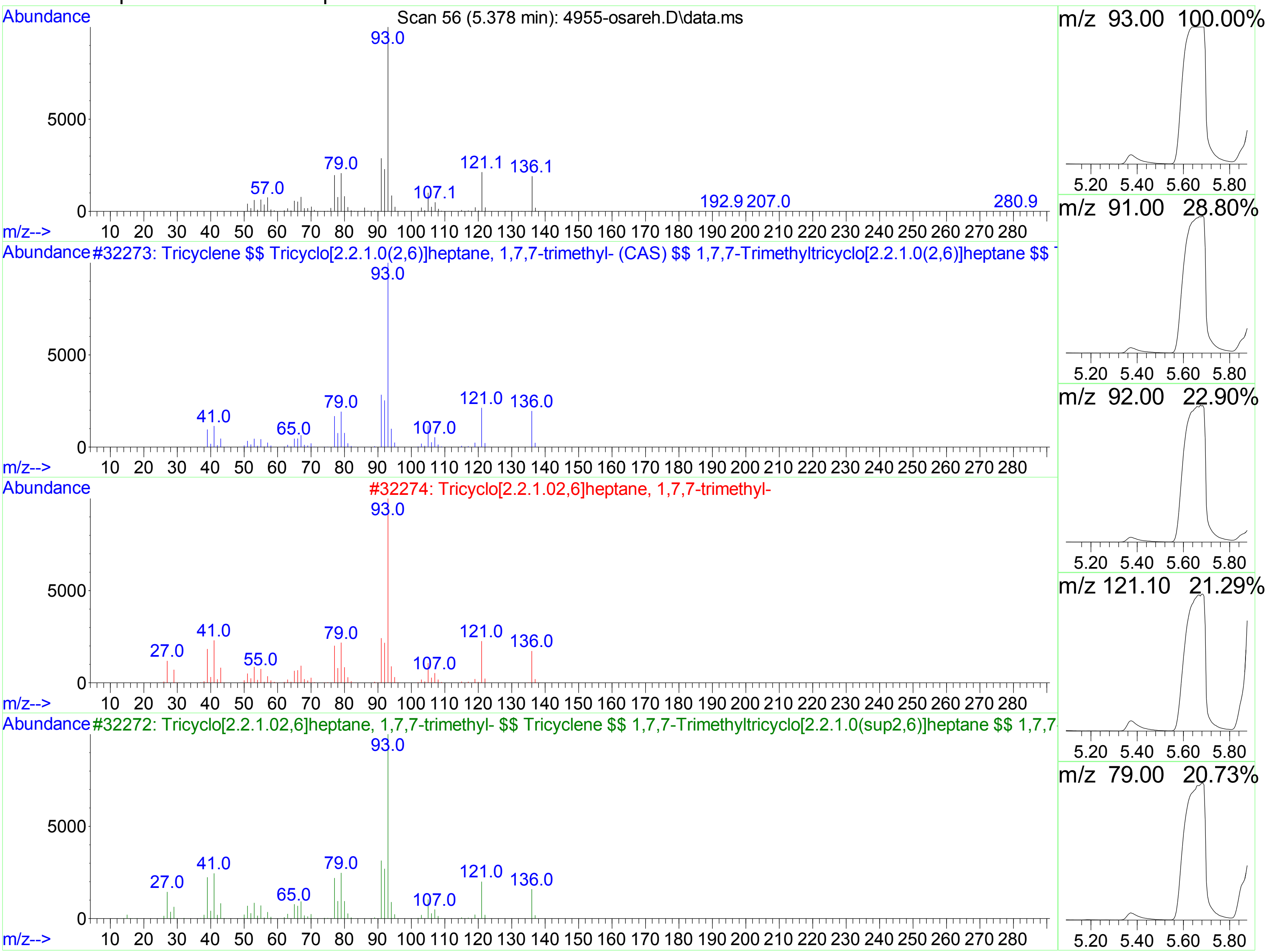

Data File: G:\ÌÒÇÑÔ 1399\99-11-18\4955-osareh.D
Sample : 4955-osareh

Peak Number: 2 at 5.378 min Area: 73323253 Area % 0.48

The 3 best hits from each library. Ref\# CAS\# Qual

E:\Database\wiley7n.l

1	Tricyclene $$ Tricyclo[2.2.1.0(2...	32273 000508-32-7	96
2	Tricyclo[2.2.1.02,6]heptane, 1,7...	32274 000508-32-7	96
3	Tricyclo[2.2.1.02,6]heptane, 1,7...	32272 000508-32-7	95

Unknown Spectrum based on Apex

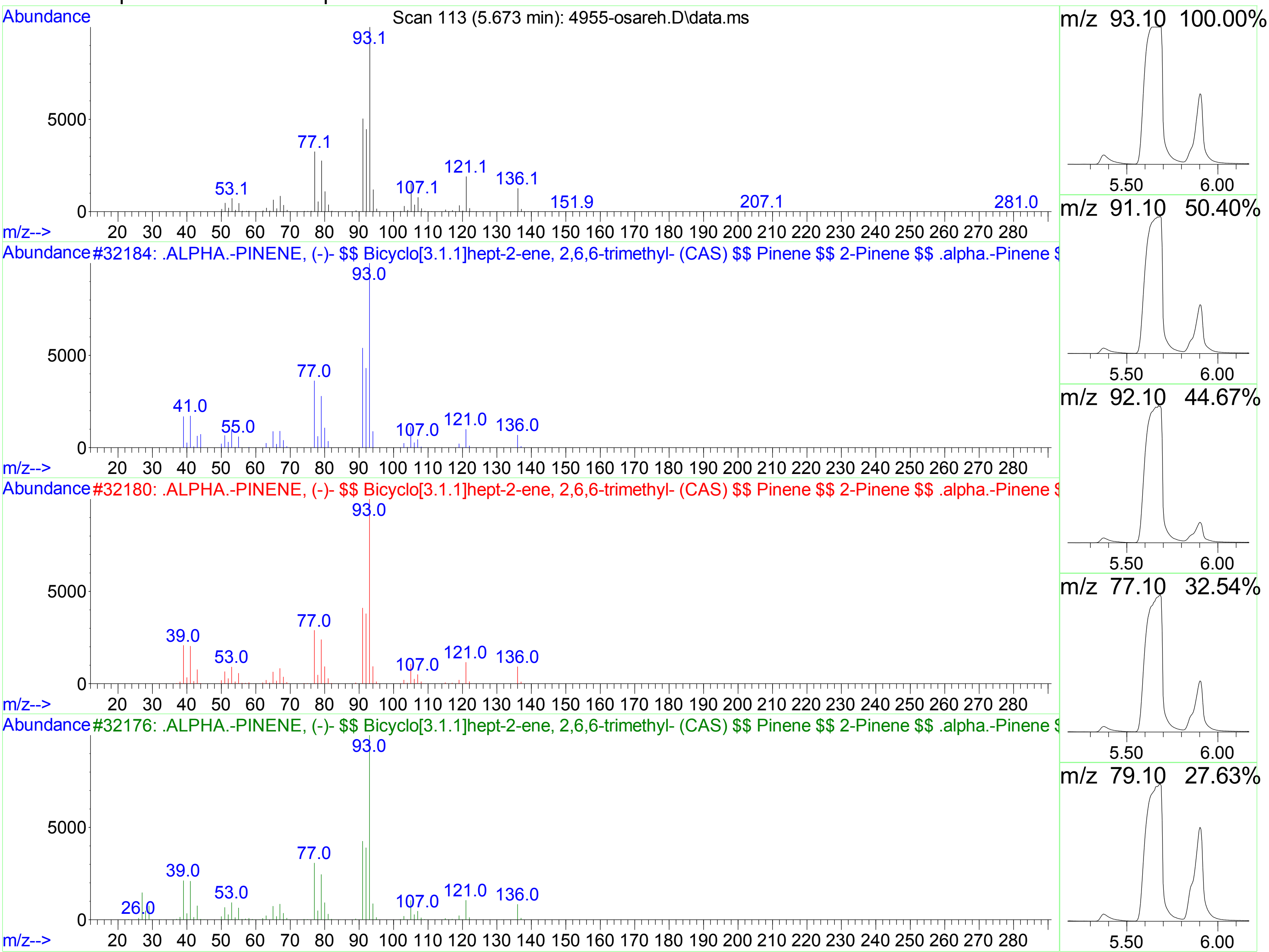

Data File: G:\ÌÒÇÑÔ 1399\99-11-18\4955-osareh.D
Sample : 4955-osareh

Peak Number: 3 at 5.673 min Area: 2175773565 Area % 14.18

The 3 best hits from each library.	Ref#	CAS#	Qual
E:\Database\wiley7n.l			
1 .ALPHA.-PINENE, (-)- $$ Bicyclo[...	32184	000080-56-8	96
2 .ALPHA.-PINENE, (-)- $$ Bicyclo[...	32180	000080-56-8	95
3 .ALPHA.-PINENE, (-)- $$ Bicyclo[...	32176	000080-56-8	94

Unknown Spectrum based on Apex

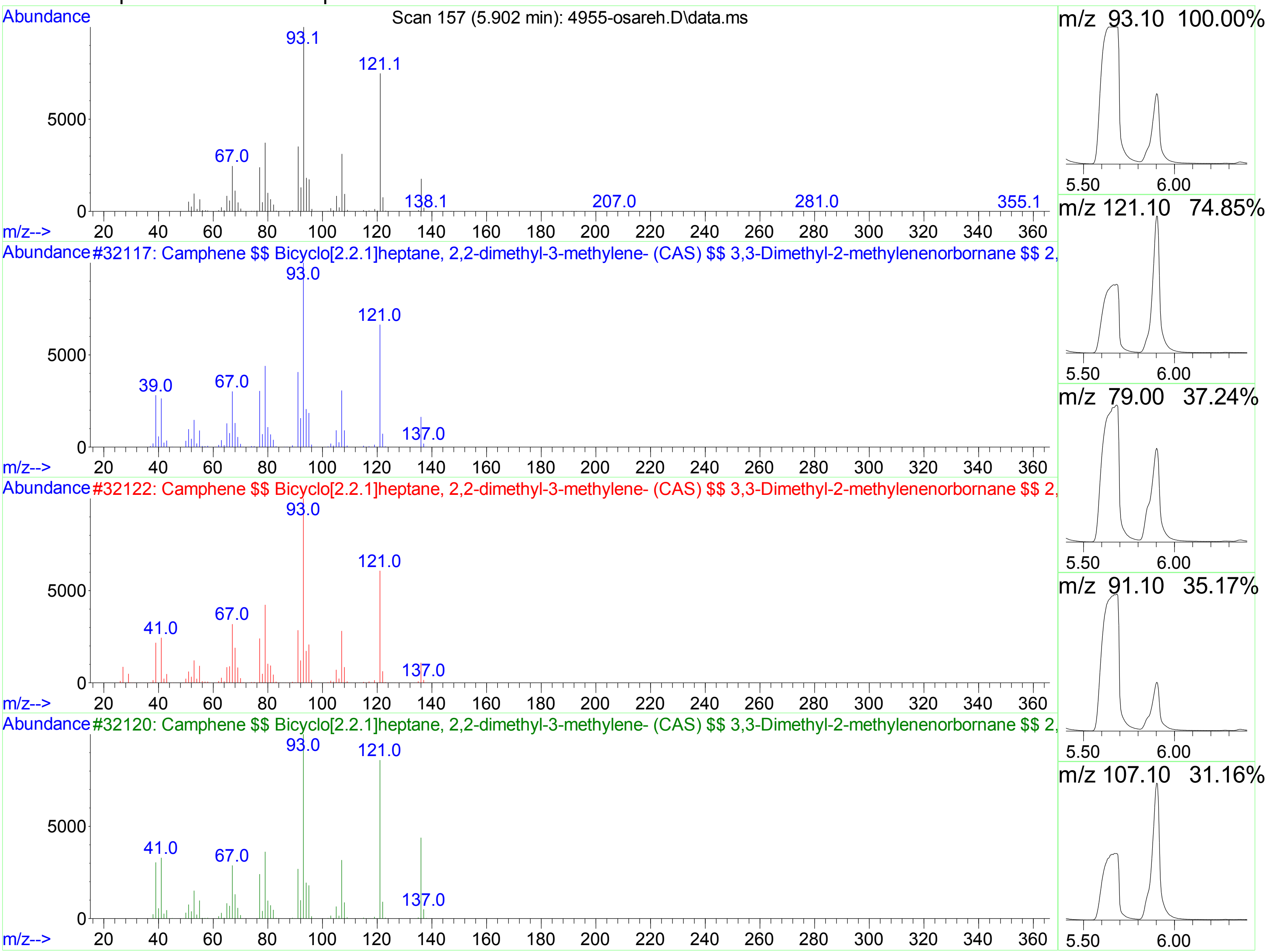

Data File: G:\ÌÒÇÑÔ 1399\99-11-18\4955-osareh.D
Sample : 4955-osareh

Peak Number: 4 at 5.902 min Area: 698787643 Area % 4.55

The 3 best hits from each library. Ref\# CAS\# Qual

E:\Database\wiley7n.l

1 Camphene $$ Bicyclo[2.2.1]heptan...	32117	000079-92-5	98
2 Camphene $$ Bicyclo[2.2.1]heptan...	32122	000079-92-5	97
3 Camphene $$ Bicyclo[2.2.1]heptan...	32120	000079-92-5	97

Unknown Spectrum based on Apex

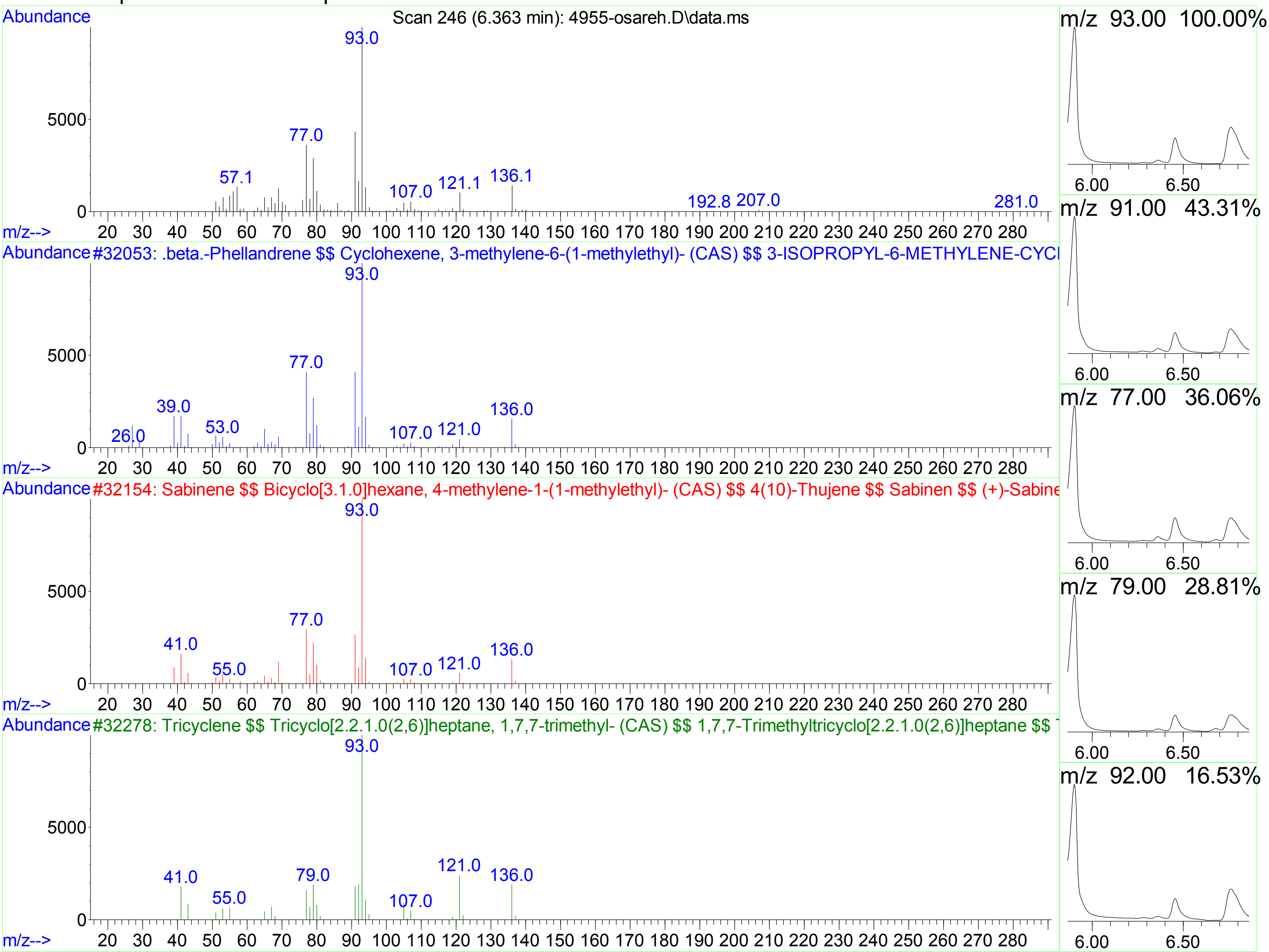

Data File: G:\ÌÒÇÑÔ 1399\99-11-18\4955-osareh.D
Sample : 4955-osareh

Peak Number: 5 at 6.363 min Area: 5728752 Area % 0.04

The 3 best hits from each library.	Ref\#	CAS\#	Qual
E:\Database\wiley7n.l			
1 .beta.-Phellandrene $$ Cyclohexe...	32053	000555-10-2	95
2 Sabinene $$ Bicyclo[3.1.0]hexane...	32154	003387-41-5	95
3 Tricyclene $$ Tricyclo[2.2.1.0(2...	32278	000508-32-7	95

Unknown Spectrum based on Apex

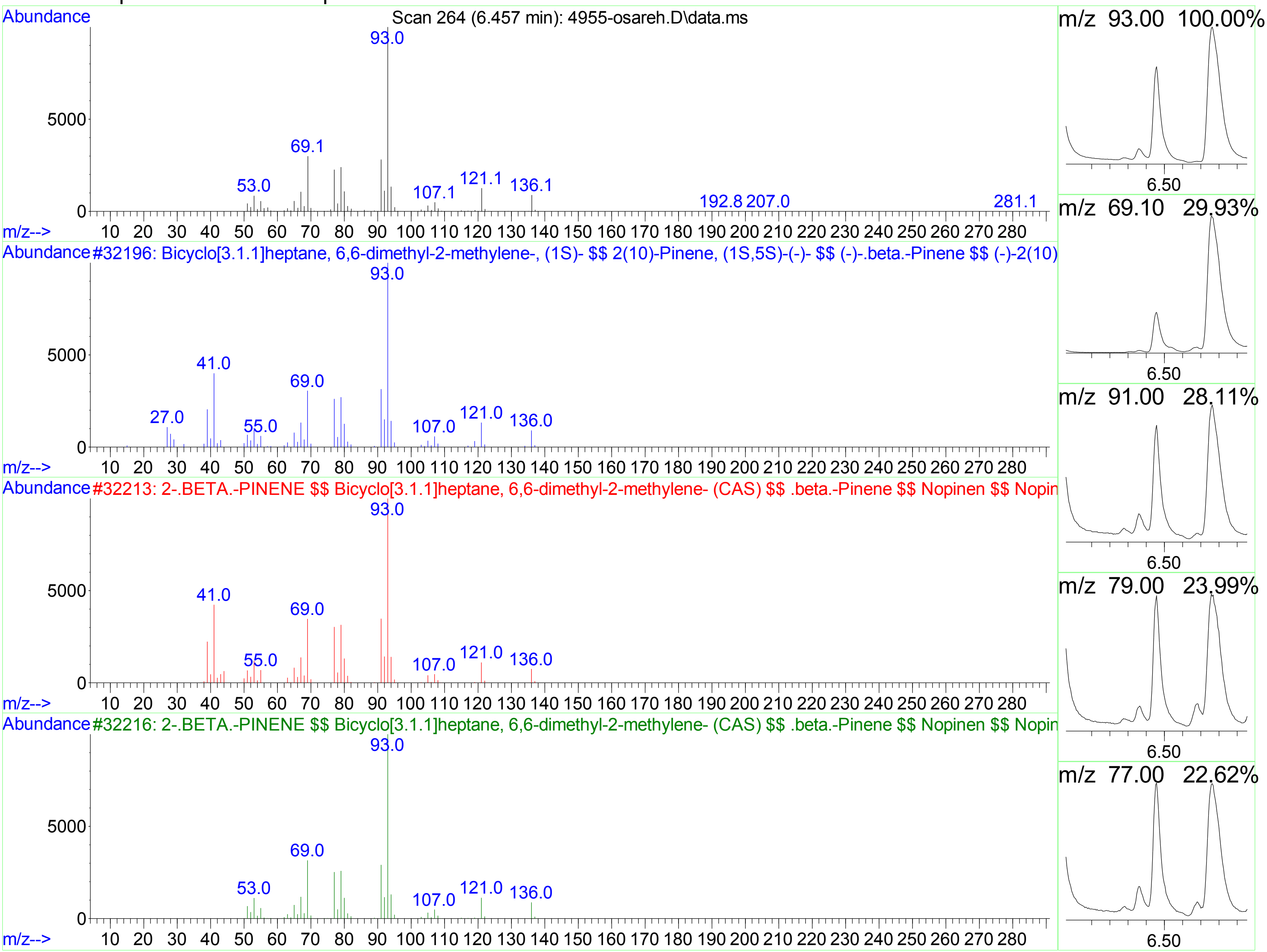

Data File: G:\ÌÒÇÑÔ 1399\99-11-18\4955-osareh.D
Sample : 4955-osareh

Peak Number: 6 at 6.457 min Area: 60729451 Area % 0.40

The 3 best hits from each library.	Ref#	CAS#	Qual
E:\Database\wiley7n.l			
1 Bicyclo[3.1.1]heptane, 6,6-dimet...	32196	018172-67-3	97
2 2-.BETA.-PINENE $$ Bicyclo[3.1.1...	32213	000127-91-3	96
3 2-.BETA.-PINENE $$ Bicyclo[3.1.1...	32216	000127-91-3	96

Library Search Report - ChemStation Integrator

Unknown Spectrum based on Apex

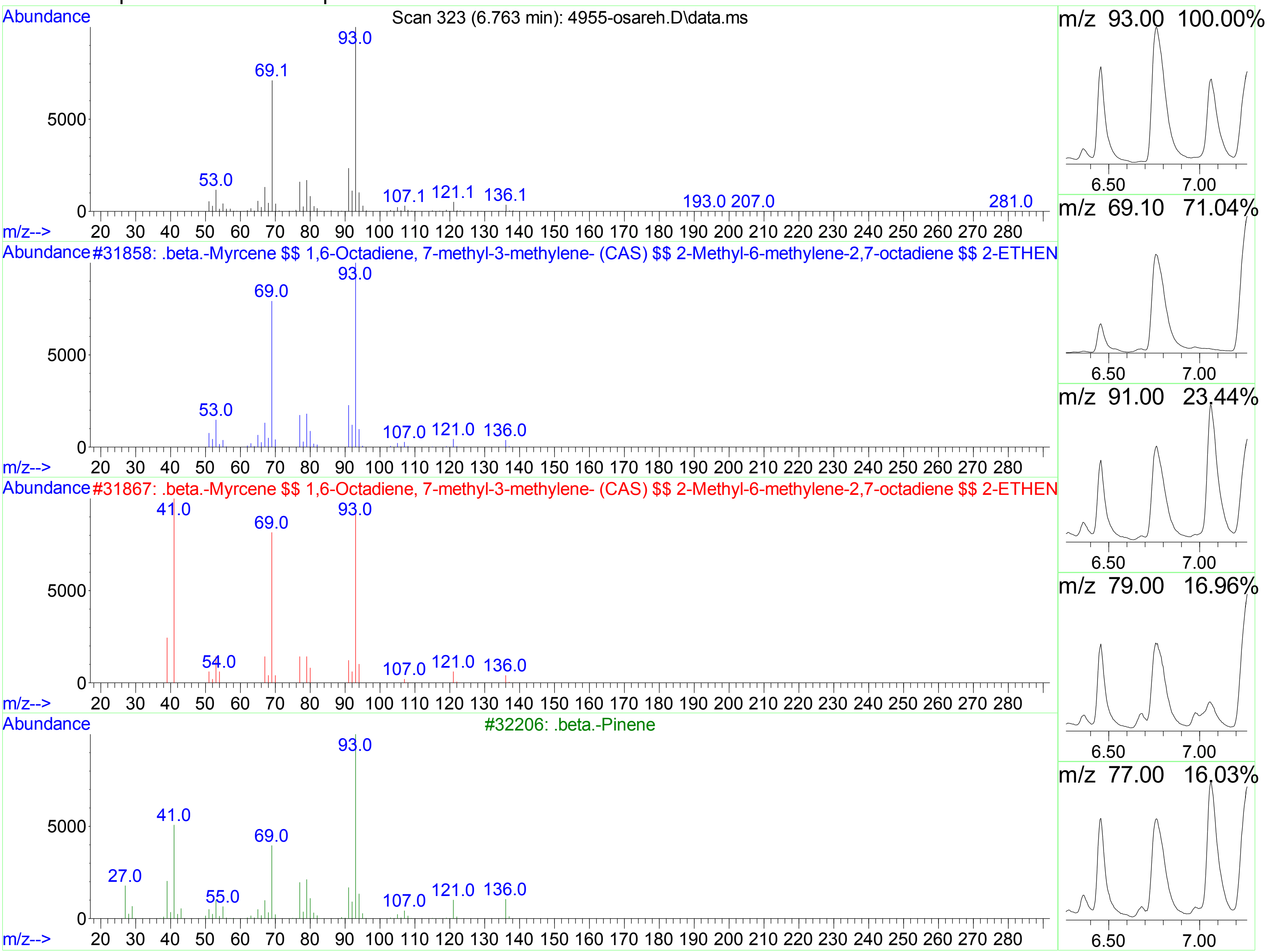

Data File: G:\ÌÒÇÑÔ 1399\99-11-18\4955-osareh.D
Sample : 4955-osareh

Peak Number: 7 at 6.763 min Area: 164667773 Area % 1.07

The 3 best hits from each library.	Ref#	CAS#	Qual
E:\Database\wiley7n.l			
1 .beta.-Myrcene $$ 1,6-Octadiene,...	31858	000123-35-3	96
2 .beta.-Myrcene $$ 1,6-Octadiene,...	31867	000123-35-3	94
3 .beta.-Pinene	32206	000127-91-3	91

Library Search Report - ChemStation Integrator

Unknown Spectrum based on Apex

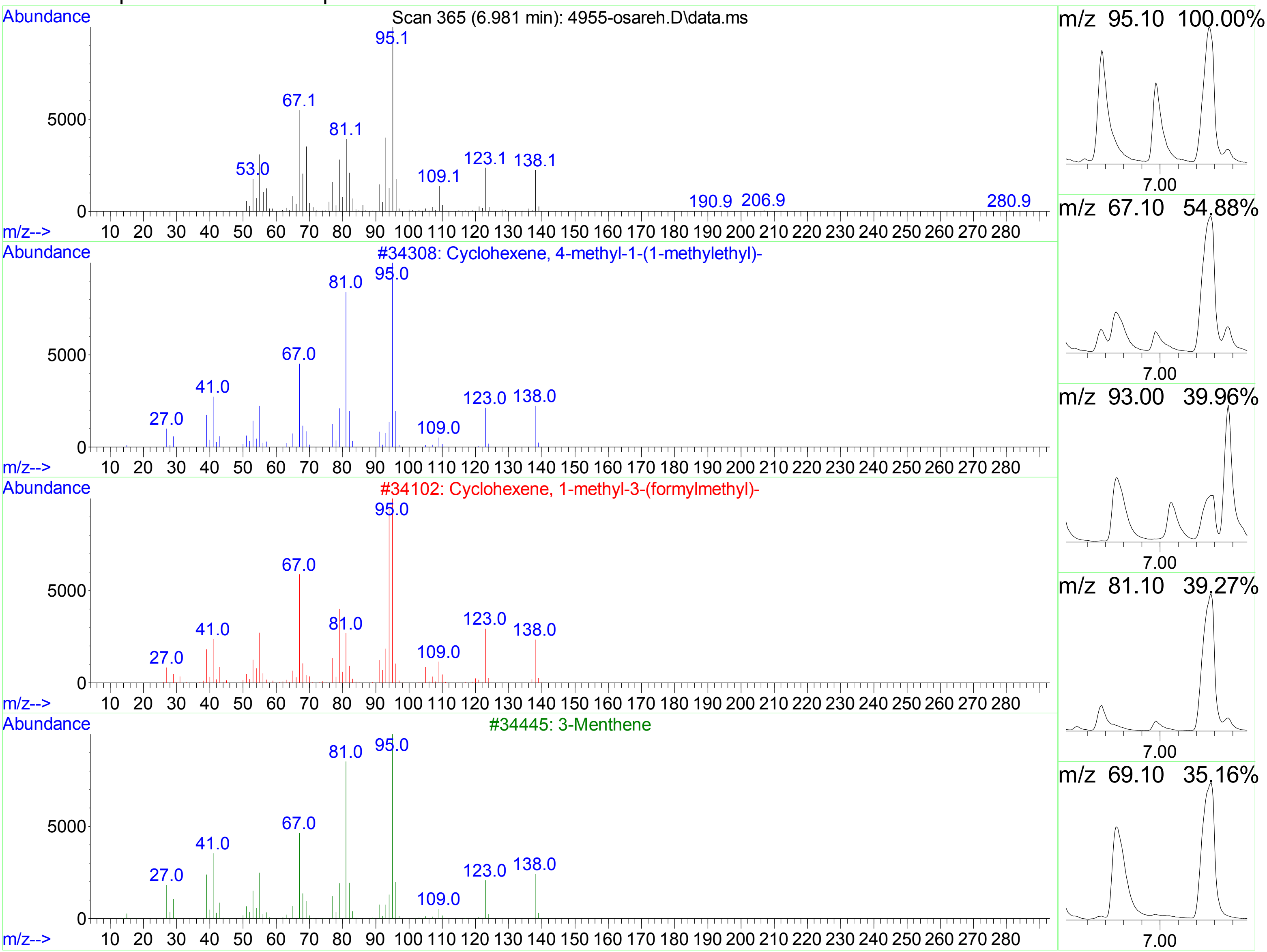

Data File: G:\ÌÒÇÑÔ 1399\99-11-18\4955-osareh.D
Sample : 4955-osareh

Peak Number: 8 at 6.981 min Area: 7373155 Area % 0.05

The 3 best hits from each library. Ref\# CAS\# Qual

E:\Database\wiley7n.l

		Ref#	CAS#	Qual
1	Cyclohexene, 4-methyl-1-(1-methy...	34308	000500-00-5	90
2	Cyclohexene, 1-methyl-3-(formylm...	34102	129993-40-4	89
3	3-Menthene	34445	000000-00-0	81

Unknown Spectrum based on Apex

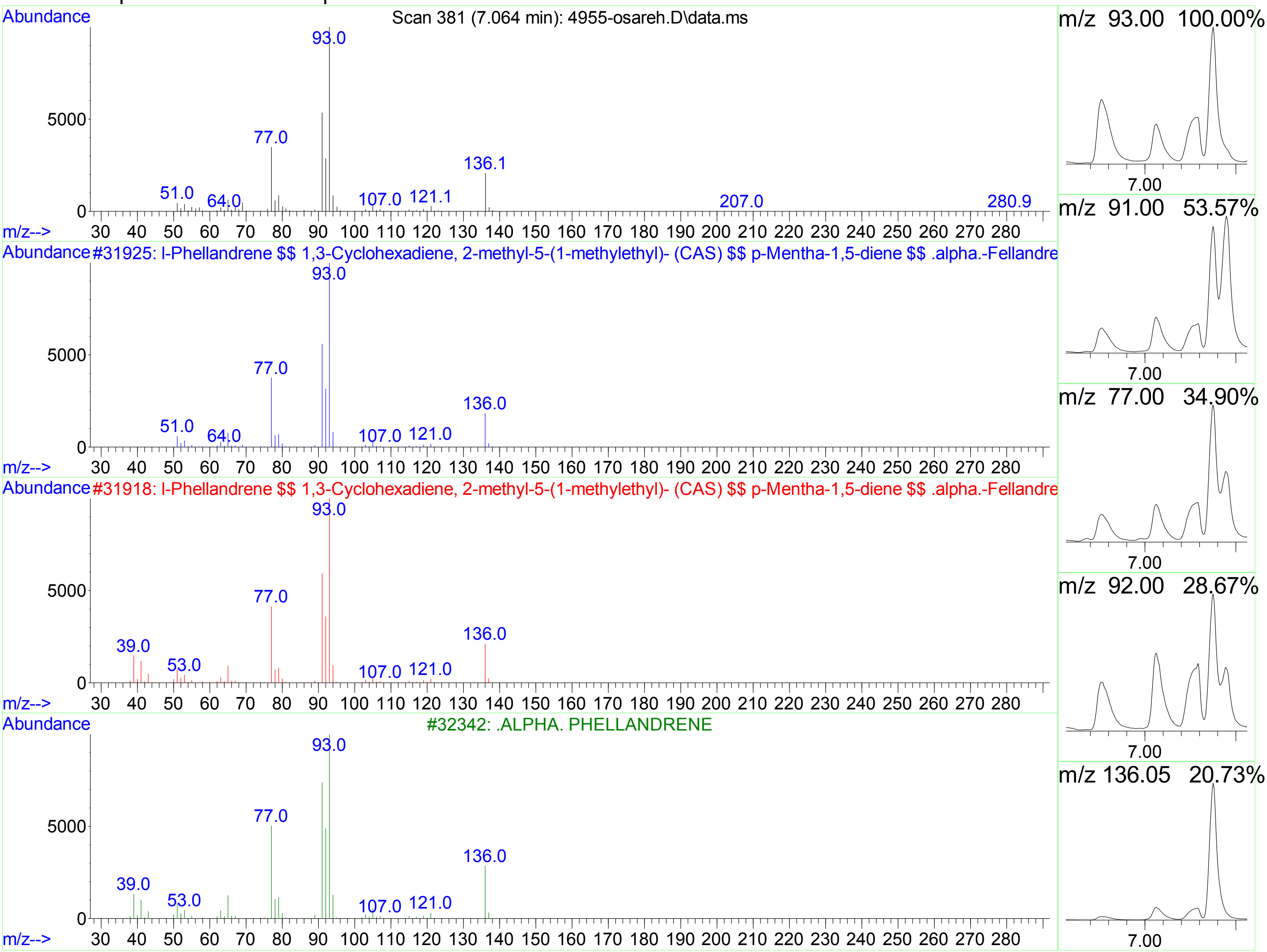

Data File: G:\lÒÇÑÔ 1399\99-11-18\4955-osareh.D
Sample : 4955-osareh

Peak Number: 9 at 7.064 min Area: 65494029 Area % 0.43

The 3 best hits from each library.	Ref#	CAS#	Qual
E:\Database\wiley7n.l			
1 l-Phellandrene $$ 1,3-Cyclohexad...	31925	000099-83-2	97
2 l-Phellandrene $$ 1,3-Cyclohexad...	31918	000099-83-2	96
3 .ALPHA. PHELLANDRENE	32342	001529-99-3	95

Library Search Report - ChemStation Integrator

Unknown Spectrum based on Apex

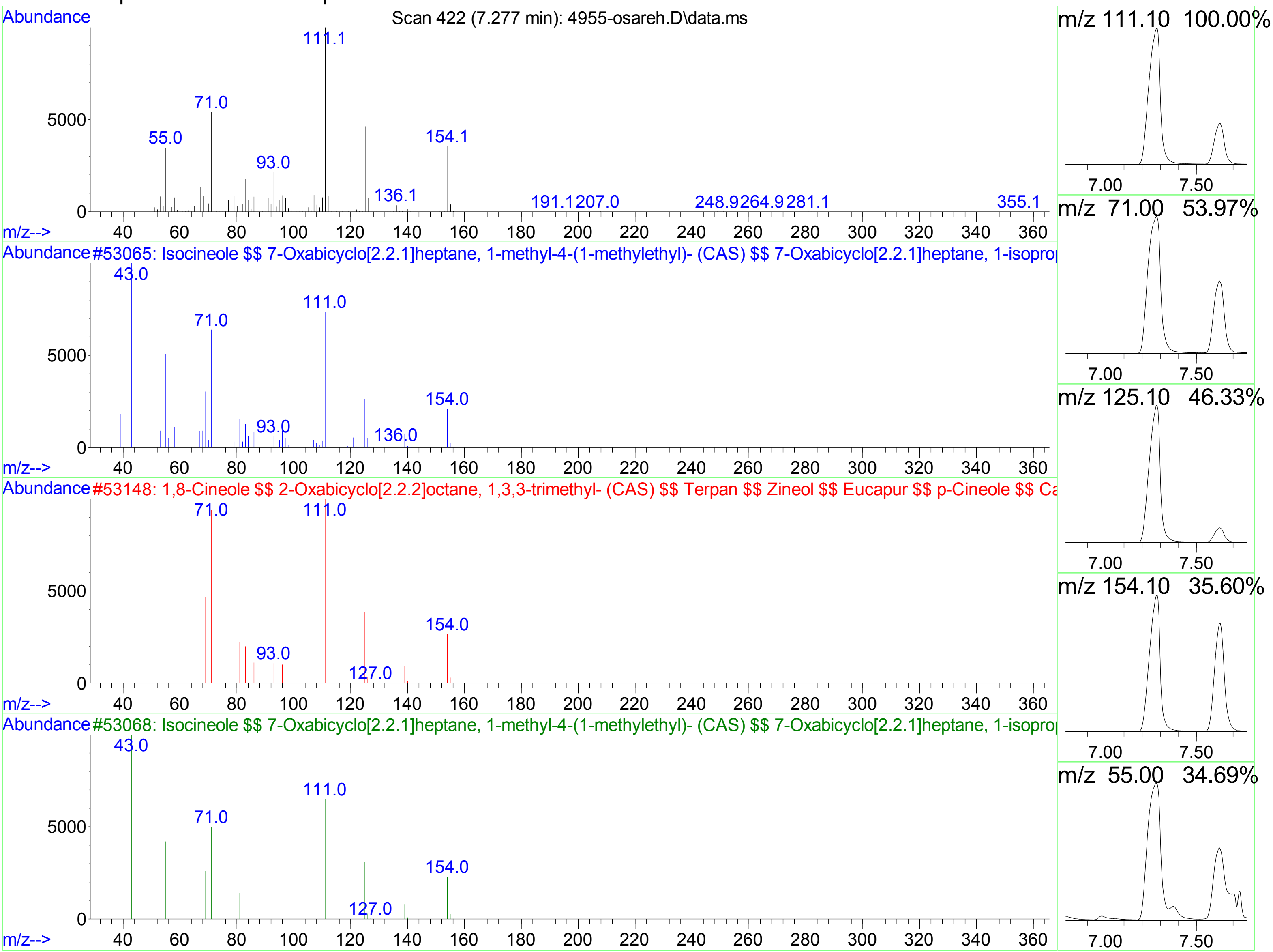

Data File: G:\ÌÒÇÑÔ 1399\99-11-18\4955-osareh.D
Sample : 4955-osareh

Peak Number: 10 at 7.277 min Area: 908752495 Area % 5.92

The 3 best hits from each library.	Ref#	CAS#	Qual
E:\Database\wiley7n.l			
1 Isocineole $$ 7-Oxabicyclo[2.2.1...	53065	000470-67-7	93
2 1,8-Cineole $$ 2-Oxabicyclo[2.2....	53148	000470-82-6	93
3 Isocineole $$ 7-Oxabicyclo[2.2.1...	53068	000470-67-7	87

Library Search Report - ChemStation Integrator

Unknown Spectrum based on Apex

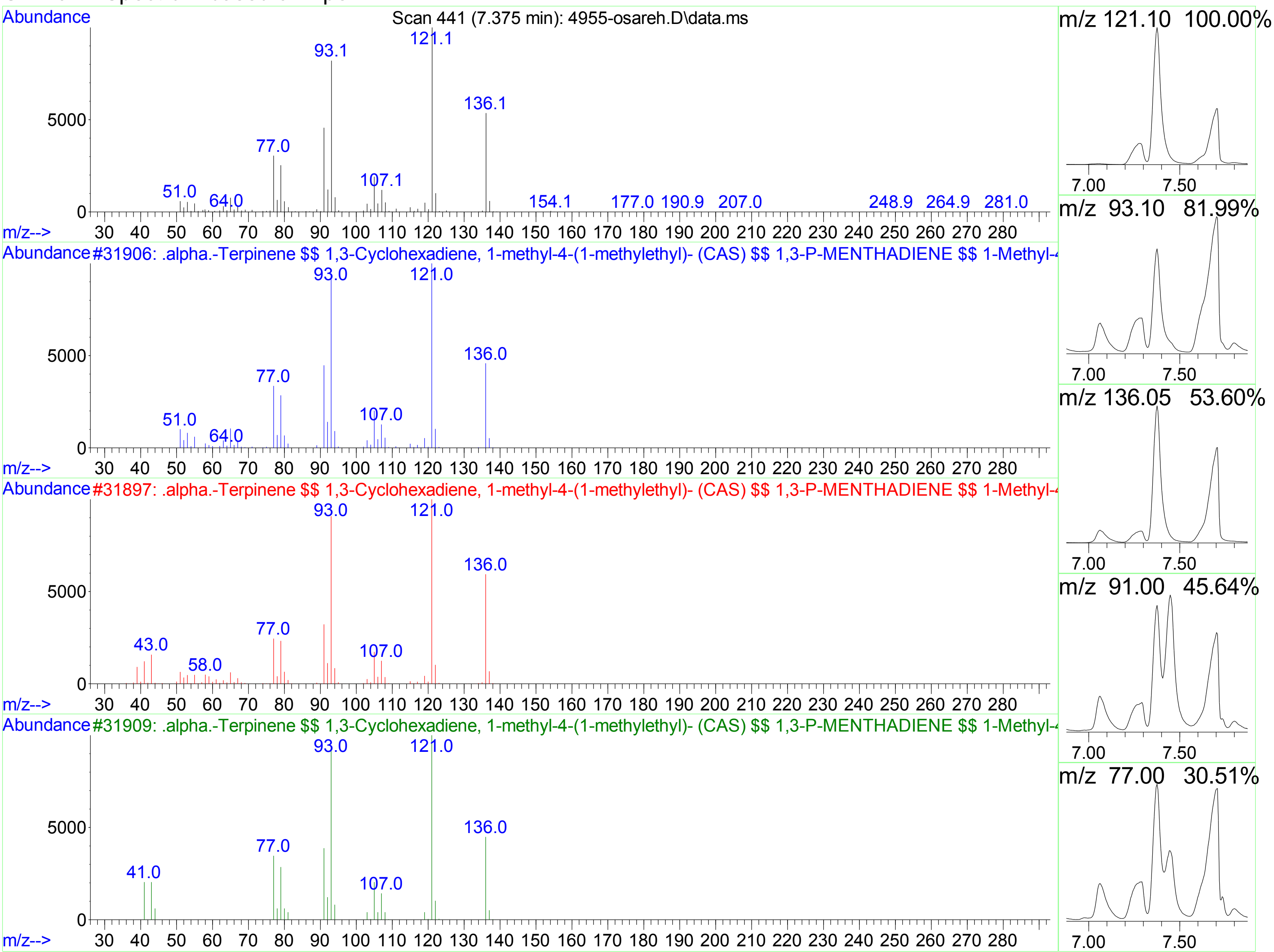

Data File: G:\[illegible]ÒÇÑÔ 1399\99-11-18\4955-osareh.D
Sample : 4955-osareh

Peak Number: 11 at 7.375 min Area: 223238949 Area % 1.45

The 3 best hits from each library.	Ref#	CAS#	Qual
E:\Database\wiley7n.l			
1 .alpha.-Terpinene $$ 1,3-Cyclohe...	31906	000099-86-5	98
2 .alpha.-Terpinene $$ 1,3-Cyclohe...	31897	000099-86-5	98
3 .alpha.-Terpinene $$ 1,3-Cyclohe...	31909	000099-86-5	98

Library Search Report - ChemStation Integrator

Unknown Spectrum based on Apex

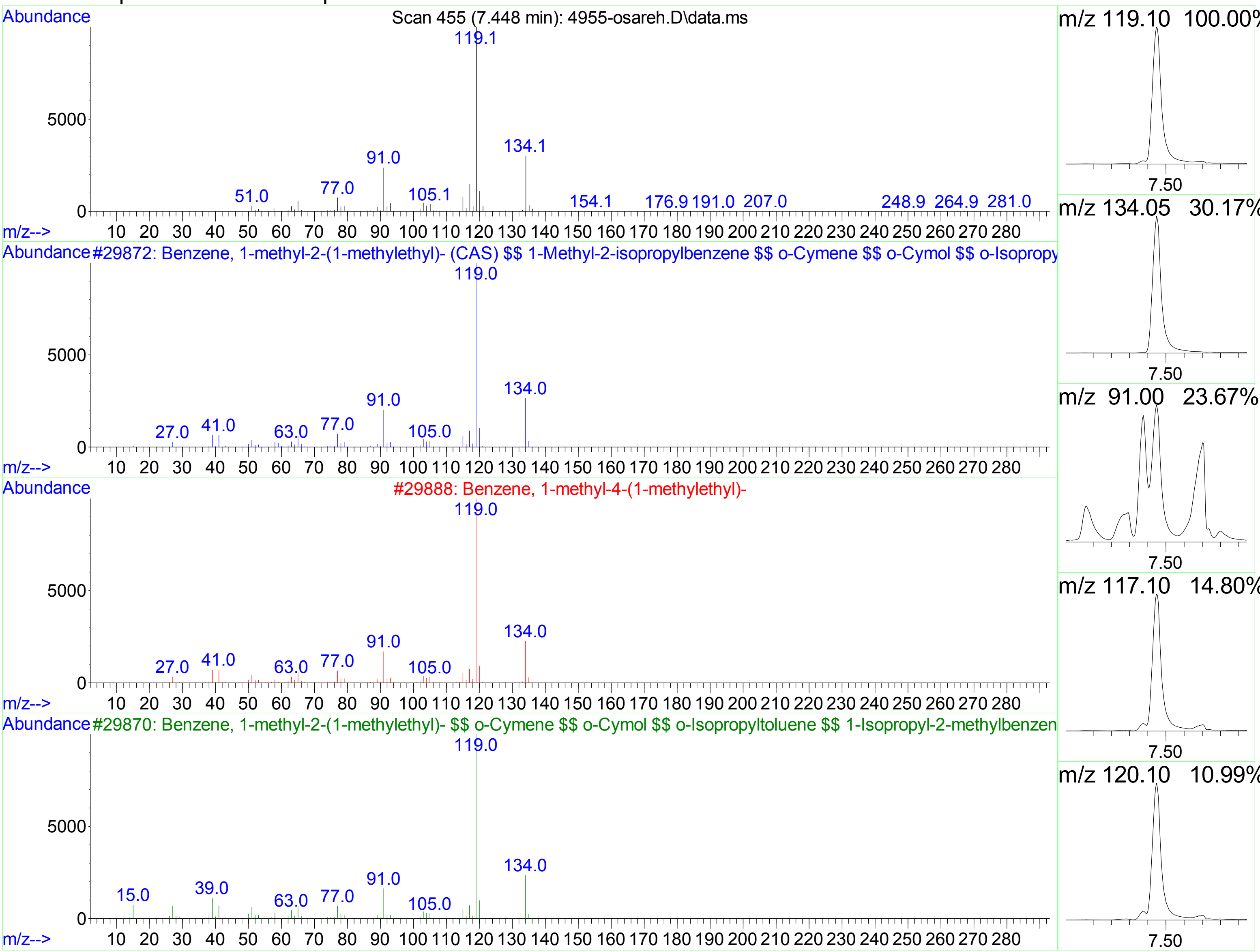

Data File: G:\ÌÒÇÑÔ 1399\99-11-18\4955-osareh.D
Sample : 4955-osareh

Peak Number: 12 at 7.448 min Area: 289364608 Area % 1.89

The 3 best hits from each library.	Ref#	CAS#	Qual
E:\Database\wiley7n.l			
1 Benzene, 1-methyl-2-(1-methyleth...	29872	000527-84-4	97
2 Benzene, 1-methyl-4-(1-methyleth...	29888	000099-87-6	97
3 Benzene, 1-methyl-2-(1-methyleth...	29870	000527-84-4	97

Library Search Report - ChemStation Integrator

Unknown Spectrum based on Apex

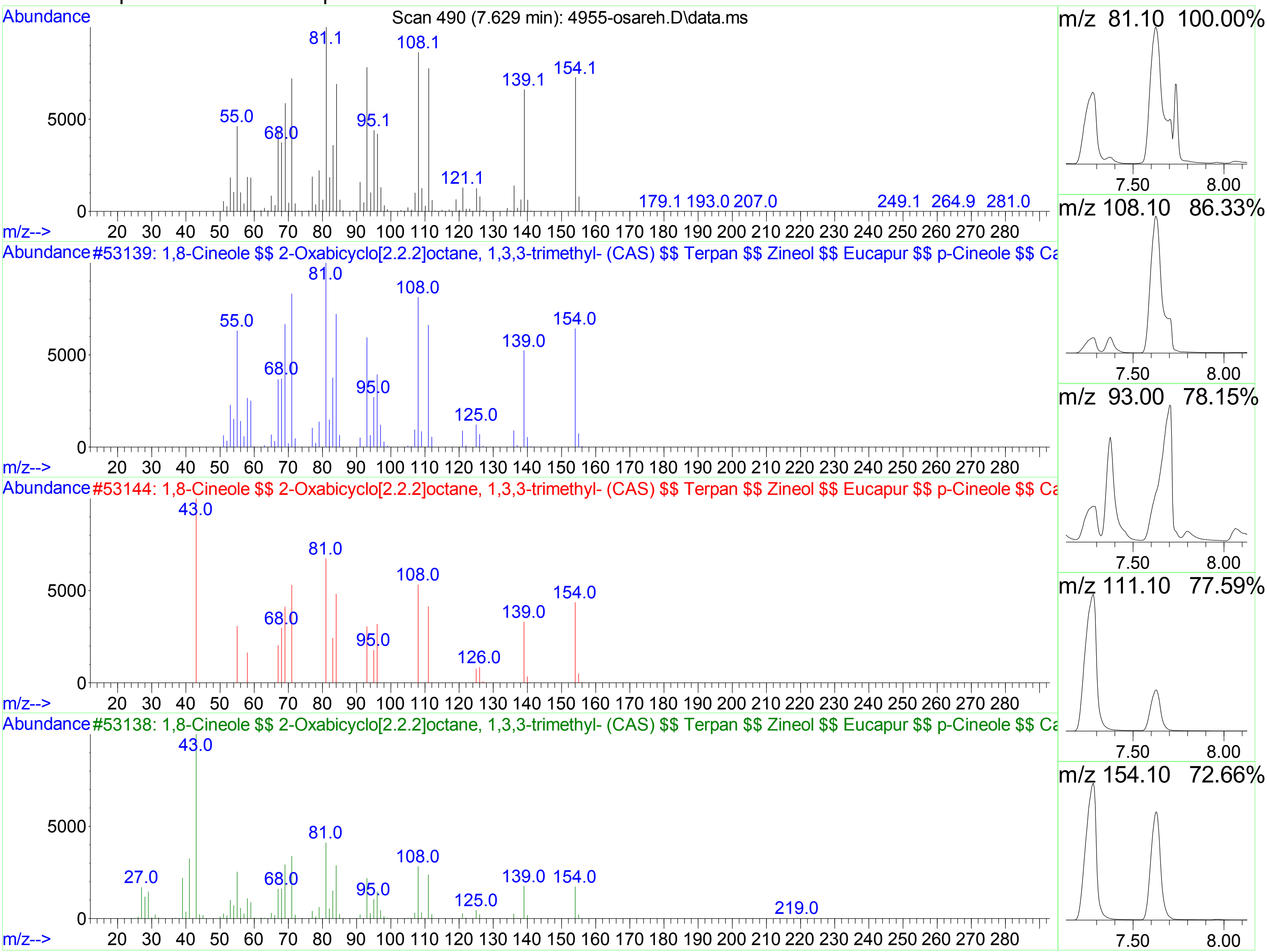

Data File: G:\ÌÒÇÑÔ 1399\99-11-18\4955-osareh.D
Sample : 4955-osareh

Peak Number: 13 at 7.629 min Area: 153027338 Area % 1.00

The 3 best hits from each library. Ref\# CAS\# Qual

E:\Database\wiley7n.l

1	1,8-Cineole $$ 2-Oxabicyclo[2.2....	53139	000470-82-6	98
2	1,8-Cineole $$ 2-Oxabicyclo[2.2....	53144	000470-82-6	94
3	1,8-Cineole $$ 2-Oxabicyclo[2.2....	53138	000470-82-6	94

Unknown Spectrum based on Apex

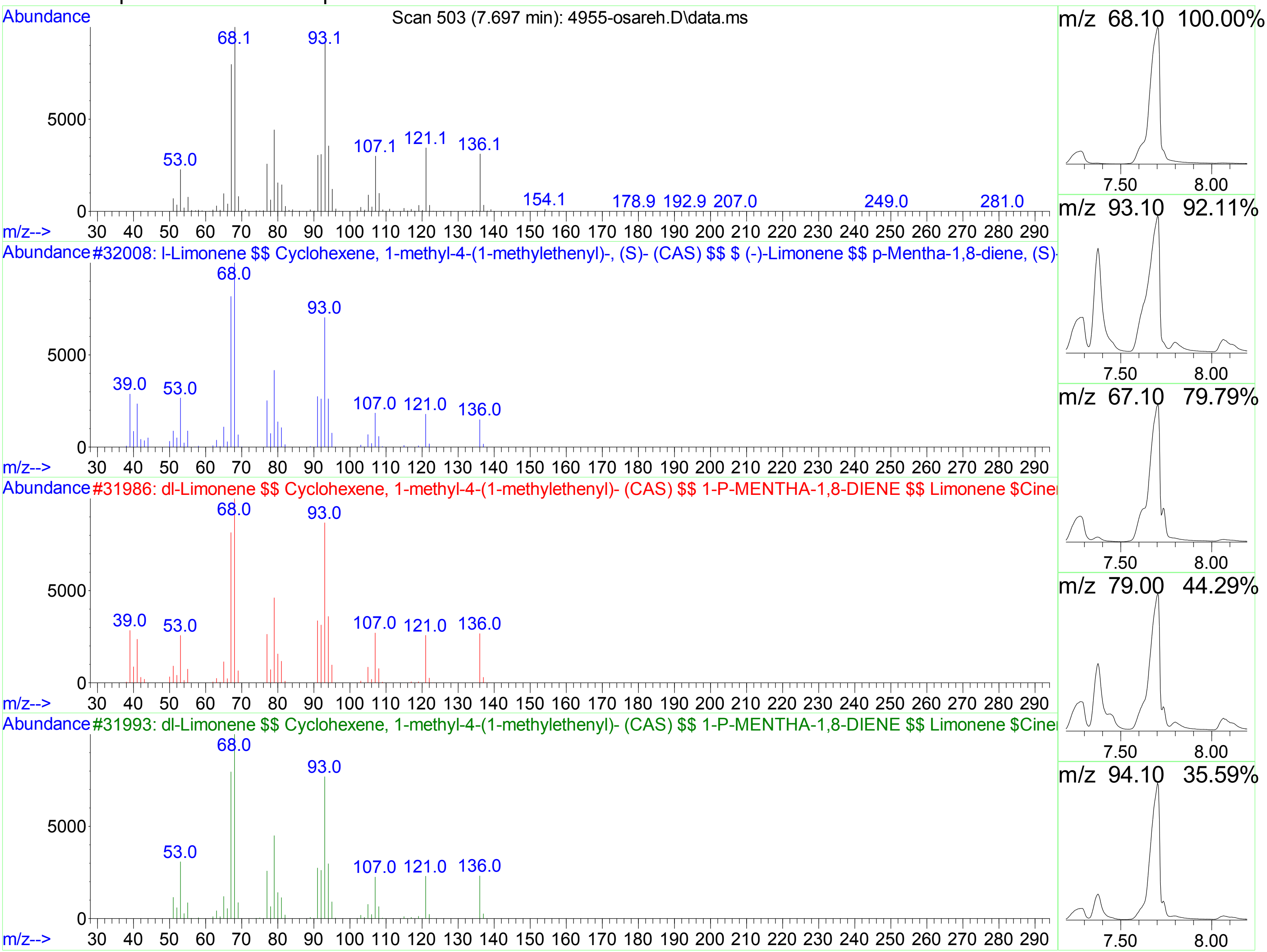

Data File: G:\ÌÒÇÑÔ 1399\99-11-18\4955-osareh.D
Sample : 4955-osareh

Peak Number: 14 at 7.697 min Area: 267380958 Area % 1.74

The 3 best hits from each library.	Ref#	CAS#	Qual
E:\Database\wiley7n.l			
1 l-Limonene $$ Cyclohexene, 1-met...	32008	005989-54-8	98
2 dl-Limonene $$ Cyclohexene, 1-me...	31986	000138-86-3	98
3 dl-Limonene $$ Cyclohexene, 1-me...	31993	000138-86-3	97

Library Search Report - ChemStation Integrator

Unknown Spectrum based on Apex

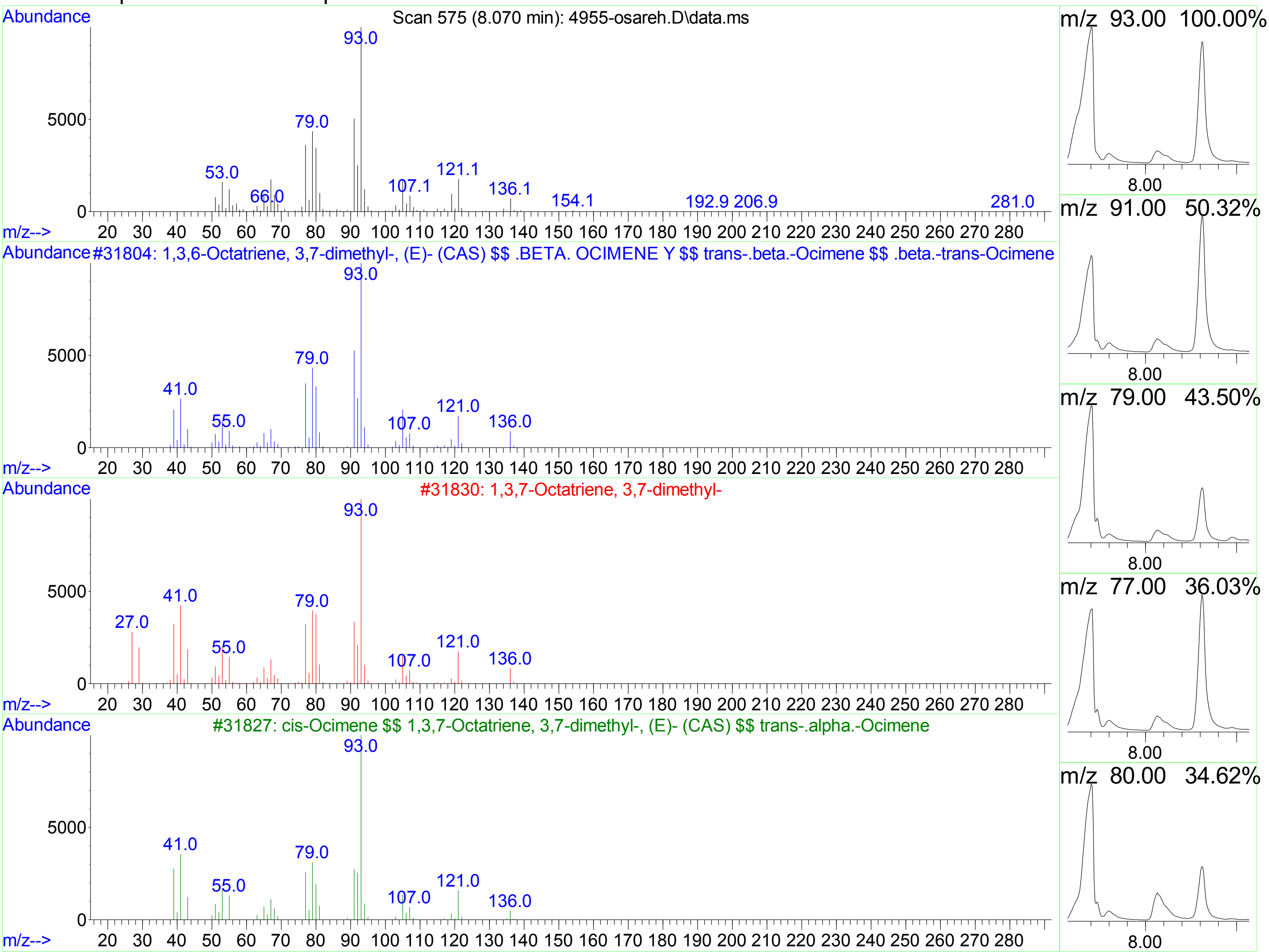

Data File: G:\\ÌÒÇÑÔ 1399\99-11-18\4955-osareh.D
Sample : 4955-osareh

Peak Number: 15 at 8.070 min Area: 63098290 Area % 0.41

The 3 best hits from each library.	Ref#	CAS#	Qual
E:\Database\wiley7n.l			
1 1,3,6-Octatriene, 3,7-dimethyl-,...	31804	003779-61-1	96
2 1,3,7-Octatriene, 3,7-dimethyl-	31830	000502-99-8	96
3 cis-Ocimene $$ 1,3,7-Octatriene,...	31827	006874-10-8	94

Unknown Spectrum based on Apex

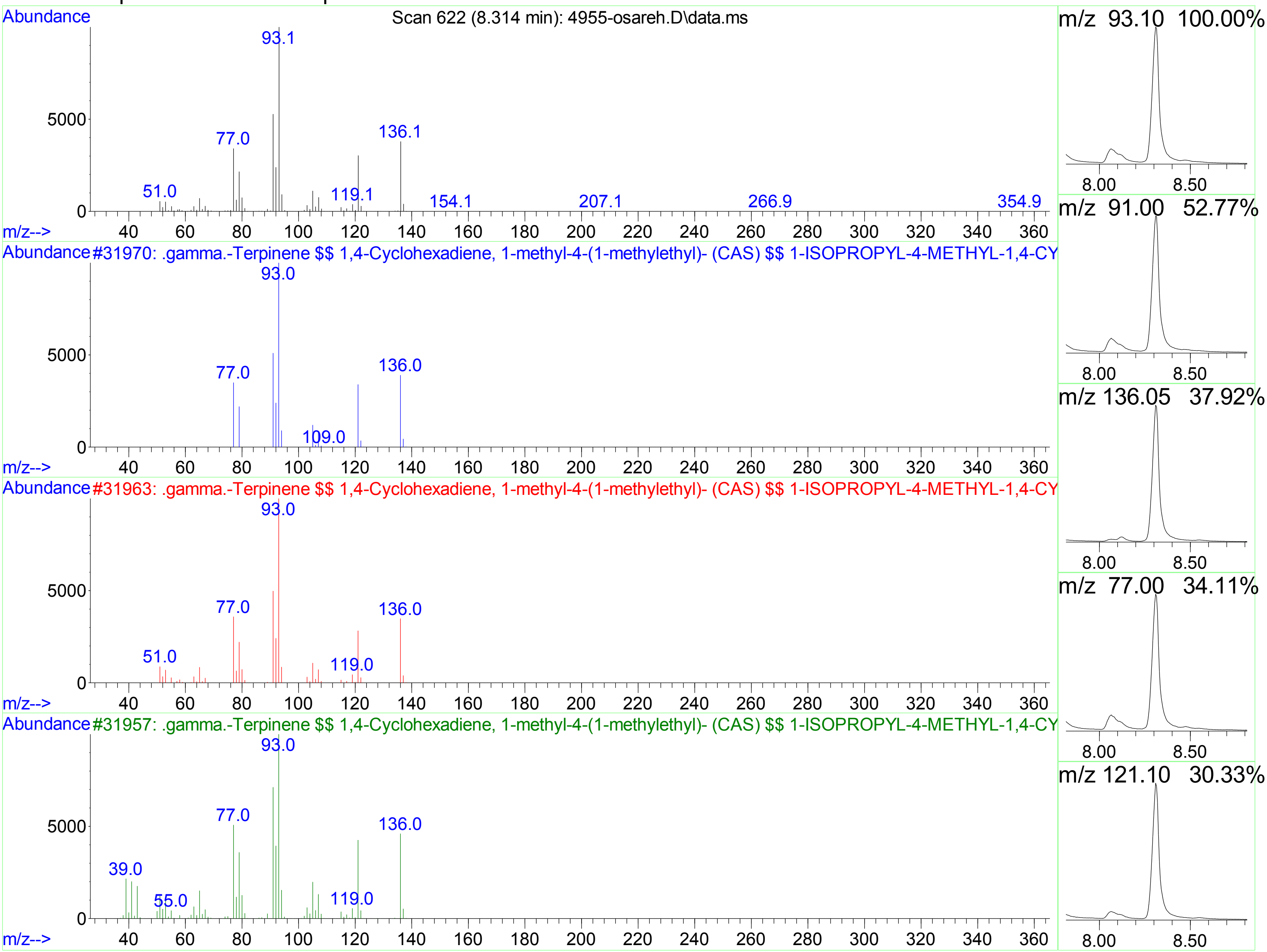

Data File: G:\ÌÒÇÑÔ 1399\99-11-18\4955-osareh.D
Sample : 4955-osareh

Peak Number: 16 at 8.314 min Area: 320454155 Area % 2.09

The 3 best hits from each library. Ref\# CAS\# Qual

E:\Database\wiley7n.l

		Ref#	CAS#	Qual
1	.gamma.-Terpinene $$ 1,4-Cyclohe...	31970	000099-85-4	97
2	.gamma.-Terpinene $$ 1,4-Cyclohe...	31963	000099-85-4	96
3	.gamma.-Terpinene $$ 1,4-Cyclohe...	31957	000099-85-4	96

Unknown Spectrum based on Apex

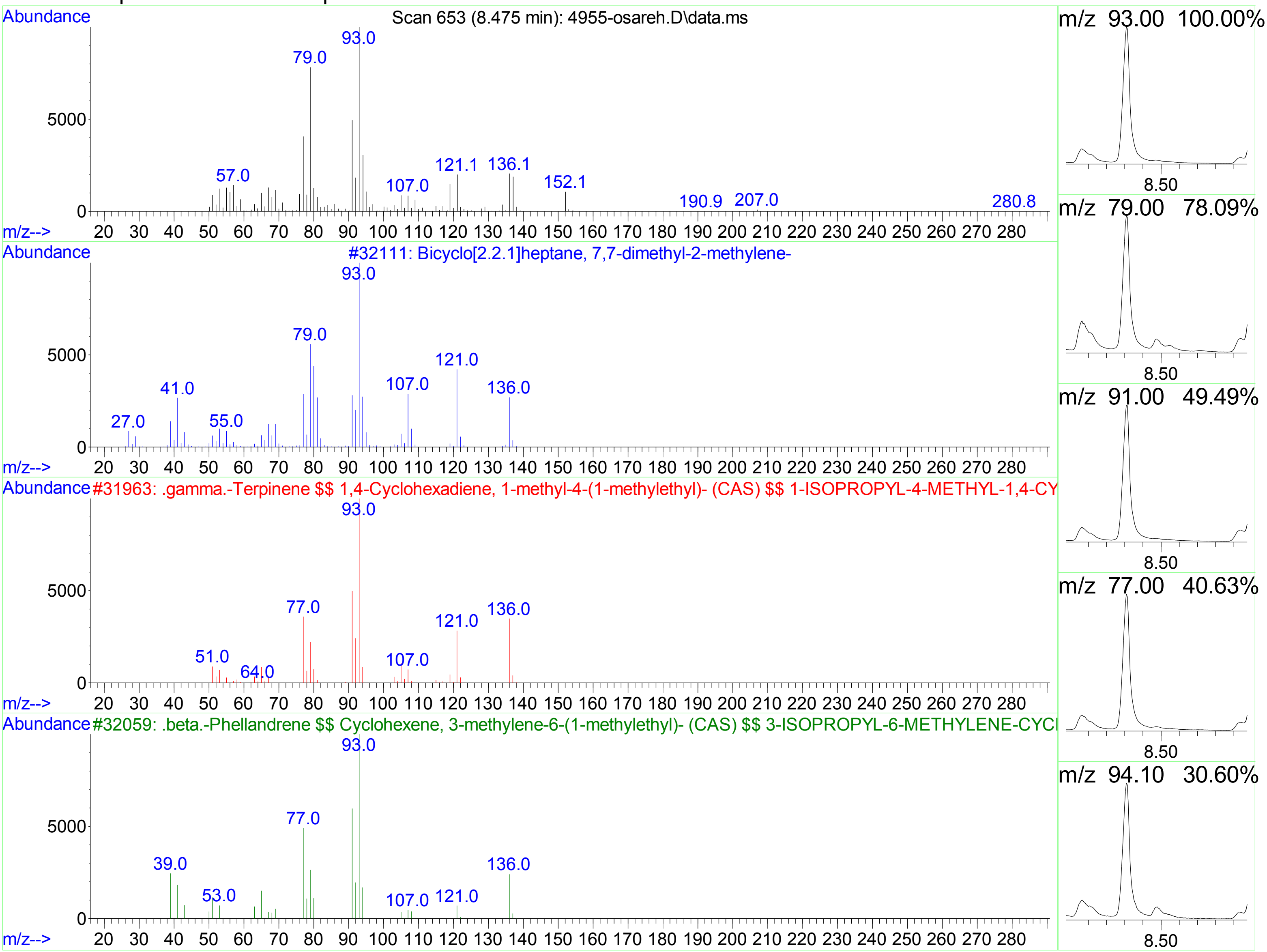

Data File: G:\ÌÒÇÑÔ 1399\99-11-18\4955-osareh.D
Sample : 4955-osareh

Peak Number: 17 at 8.475 min Area: 3254477 Area % 0.02

The 3 best hits from each library. Ref\# CAS\# Qual

E:\Database\wiley7n.l

1 Bicyclo[2.2.1]heptane, 7,7-dimet... 32111 000471-84-1 58
2 .gamma.-Terpinene $$ 1,4-Cyclohe... 31963 000099-85-4 55
3 .beta.-Phellandrene $$ Cyclohexe... 32059 000555-10-2 53

Unknown Spectrum based on Apex

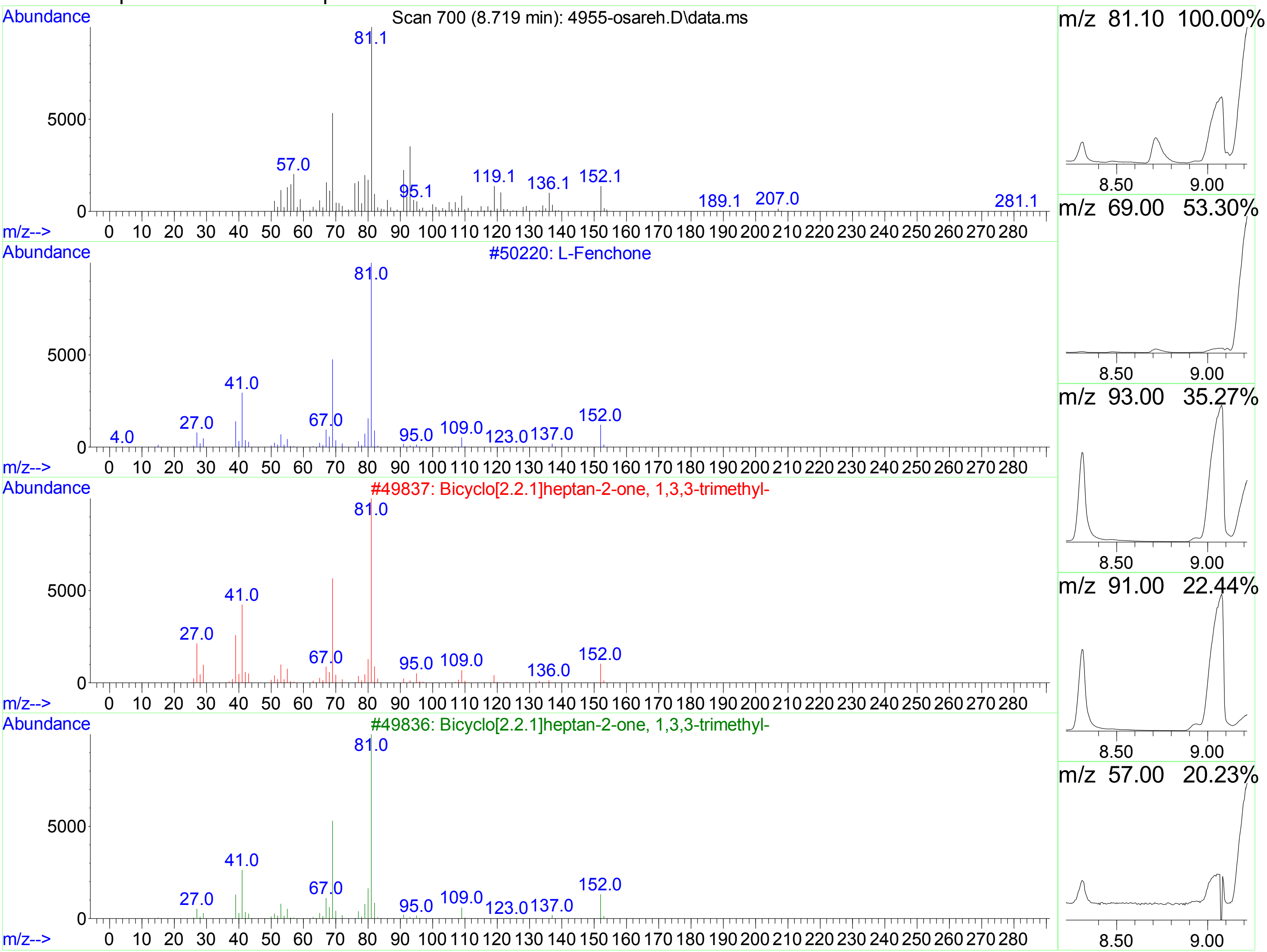

Data File: G:\ÌÒÇÑÔ 1399\99-11-18\4955-osareh.D
Sample : 4955-osareh

Peak Number: 18 at 8.719 min Area: 2929391 Area % 0.02

The 3 best hits from each library. Ref\# CAS\# Qual

E:\Database\wiley7n.l

1	L-Fenchone	50220	000126-21-6	60
2	Bicyclo[2.2.1]heptan-2-one, 1,3,...	49837	001195-79-5	60
3	Bicyclo[2.2.1]heptan-2-one, 1,3,...	49836	001195-79-5	60

Unknown Spectrum based on Apex

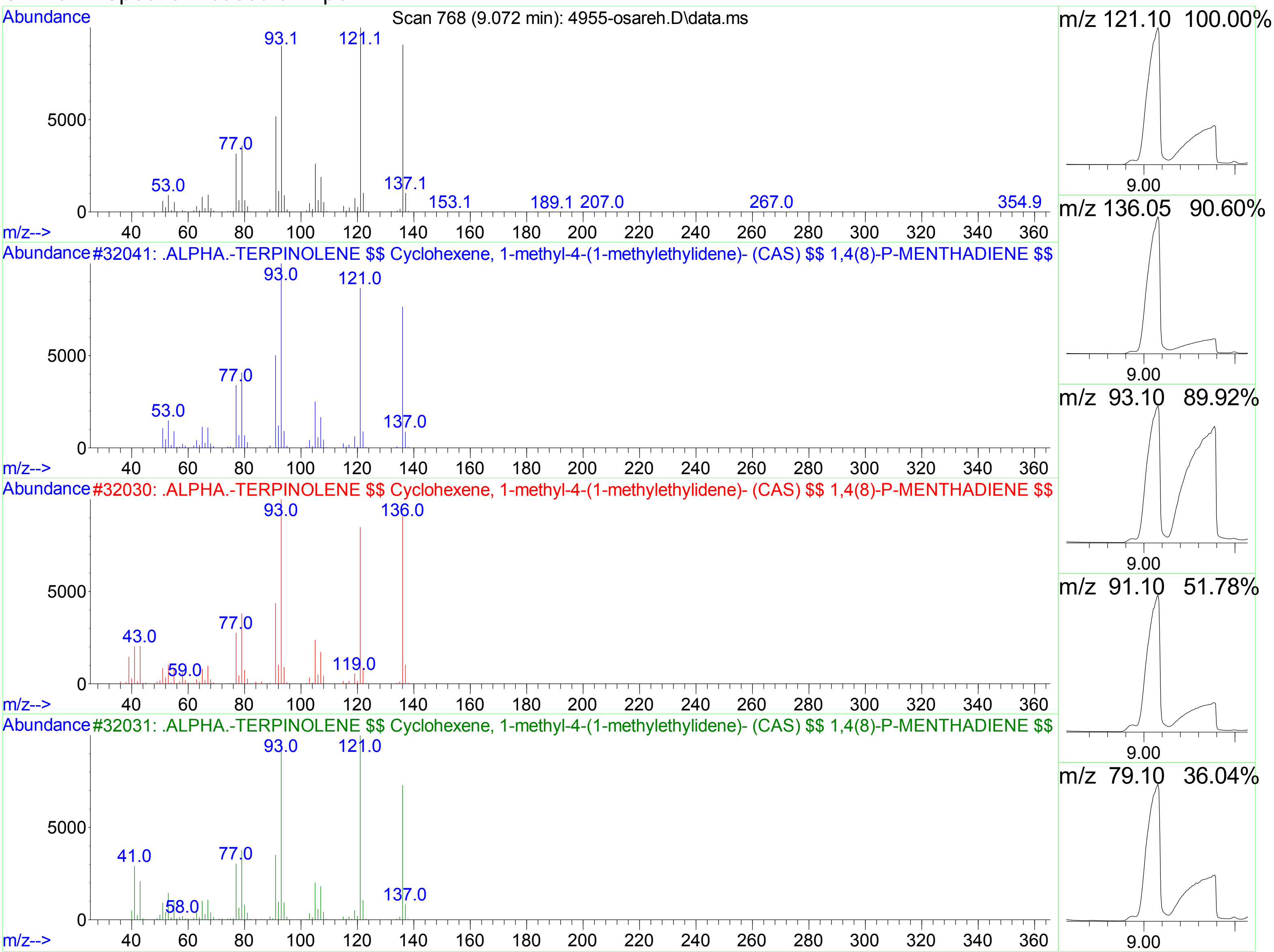

Data File: G:\ÌÒÇÑÔ 1399\99-11-18\4955-osareh.D
Sample : 4955-osareh

Peak Number: 19 at 9.072 min Area: 1320659110 Area % 8.61

The 3 best hits from each library. Ref\# CAS\# Qual

E:\Database\wiley7n.l

		Ref#	CAS#	Qual
1	.ALPHA.-TERPINOLENE $$ Cyclohexe...	32041	000586-62-9	98
2	.ALPHA.-TERPINOLENE $$ Cyclohexe...	32030	000586-62-9	98
3	.ALPHA.-TERPINOLENE $$ Cyclohexe...	32031	000586-62-9	98

Library Search Report - ChemStation Integrator

Unknown Spectrum based on Apex

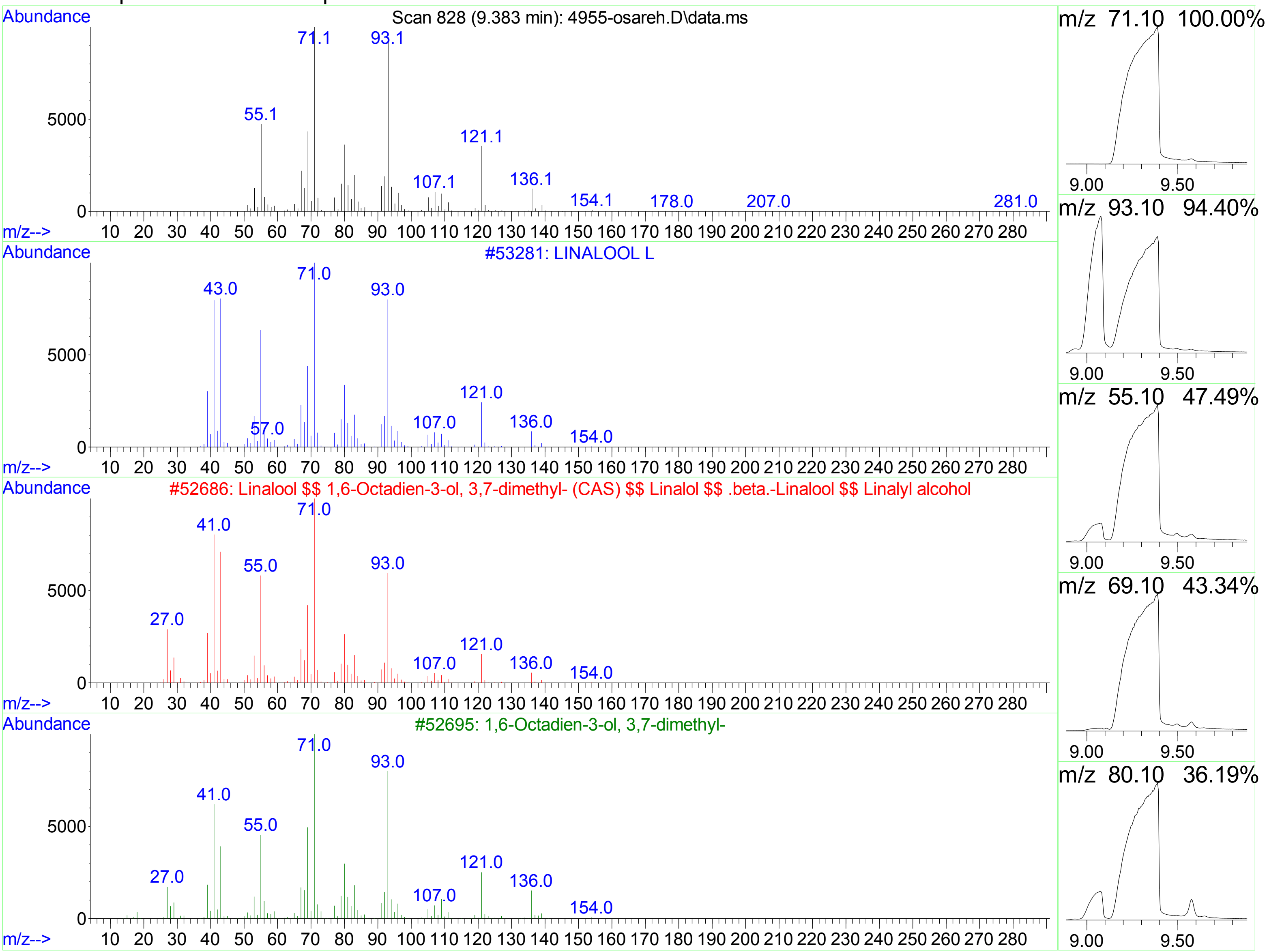

Data File: G:\\ÒÇÑÔ 1399\99-11-18\4955-osareh.D
Sample : 4955-osareh

Peak Number: 20 at 9.383 min Area: 2535851682 Area % 16.52

The 3 best hits from each library.	Ref#	CAS#	Qual
E:\Database\wiley7n.l			
1 LINALOOL L	53281	000078-70-6	96
2 Linalool $$ 1,6-Octadien-3-ol, 3...	52686	000078-70-6	95
3 1,6-Octadien-3-ol, 3,7-dimethyl-	52695	000078-70-6	94

Unknown Spectrum based on Apex

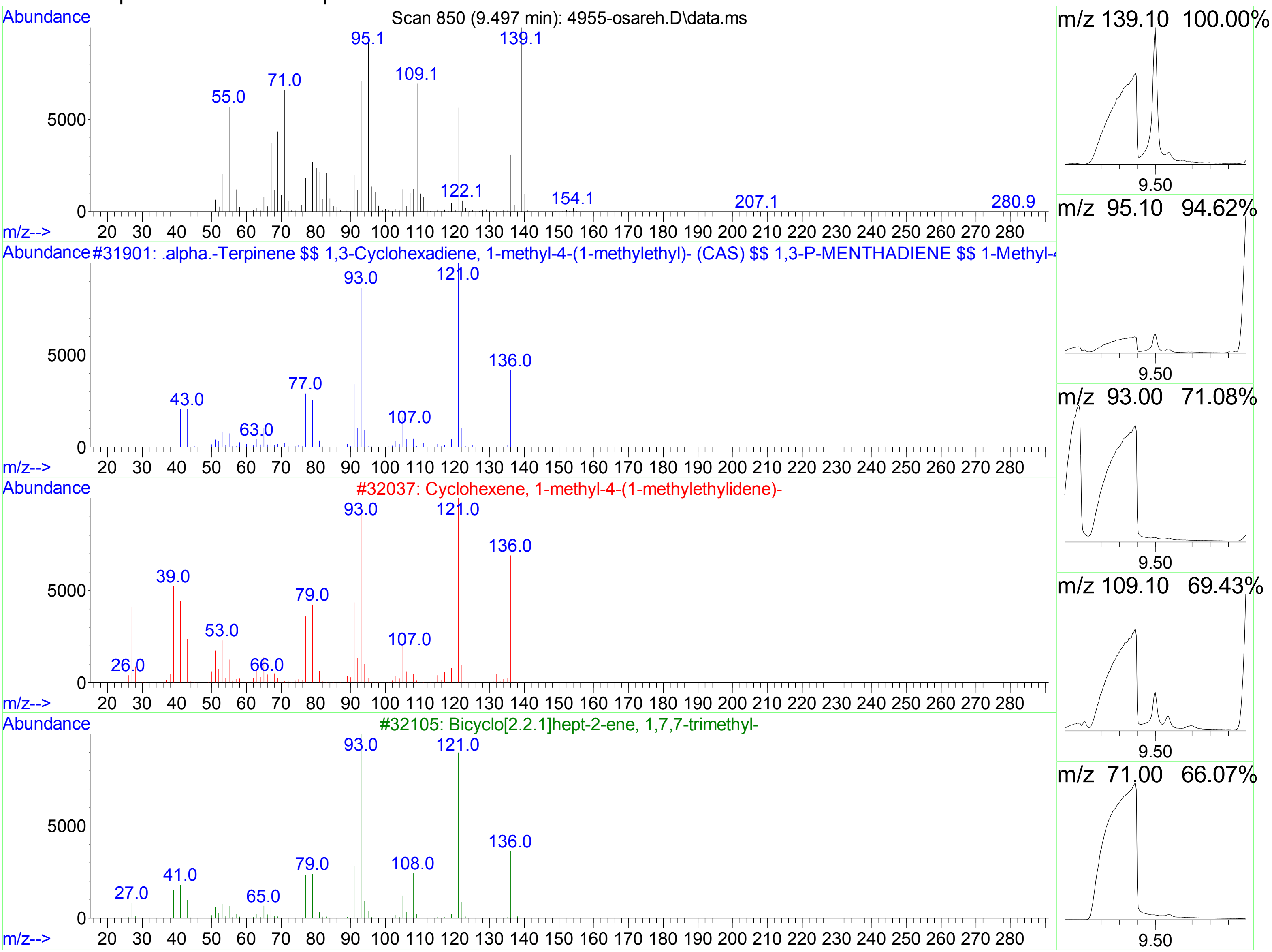

Data File: G:\ÌÒÇÑÔ 1399\99-11-18\4955-osareh.D
Sample : 4955-osareh

Peak Number: 21 at 9.497 min Area: 15489563 Area % 0.10

The 3 best hits from each library.	Ref#	CAS#	Qual
E:\Database\wiley7n.l			
1 .alpha.-Terpinene $$ 1,3-Cyclohe...	31901	000099-86-5	59
2 Cyclohexene, 1-methyl-4-(1-methy...	32037	000586-62-9	55
3 Bicyclo[2.2.1]hept-2-ene, 1,7,7-...	32105	000464-17-5	53

Unknown Spectrum based on Apex

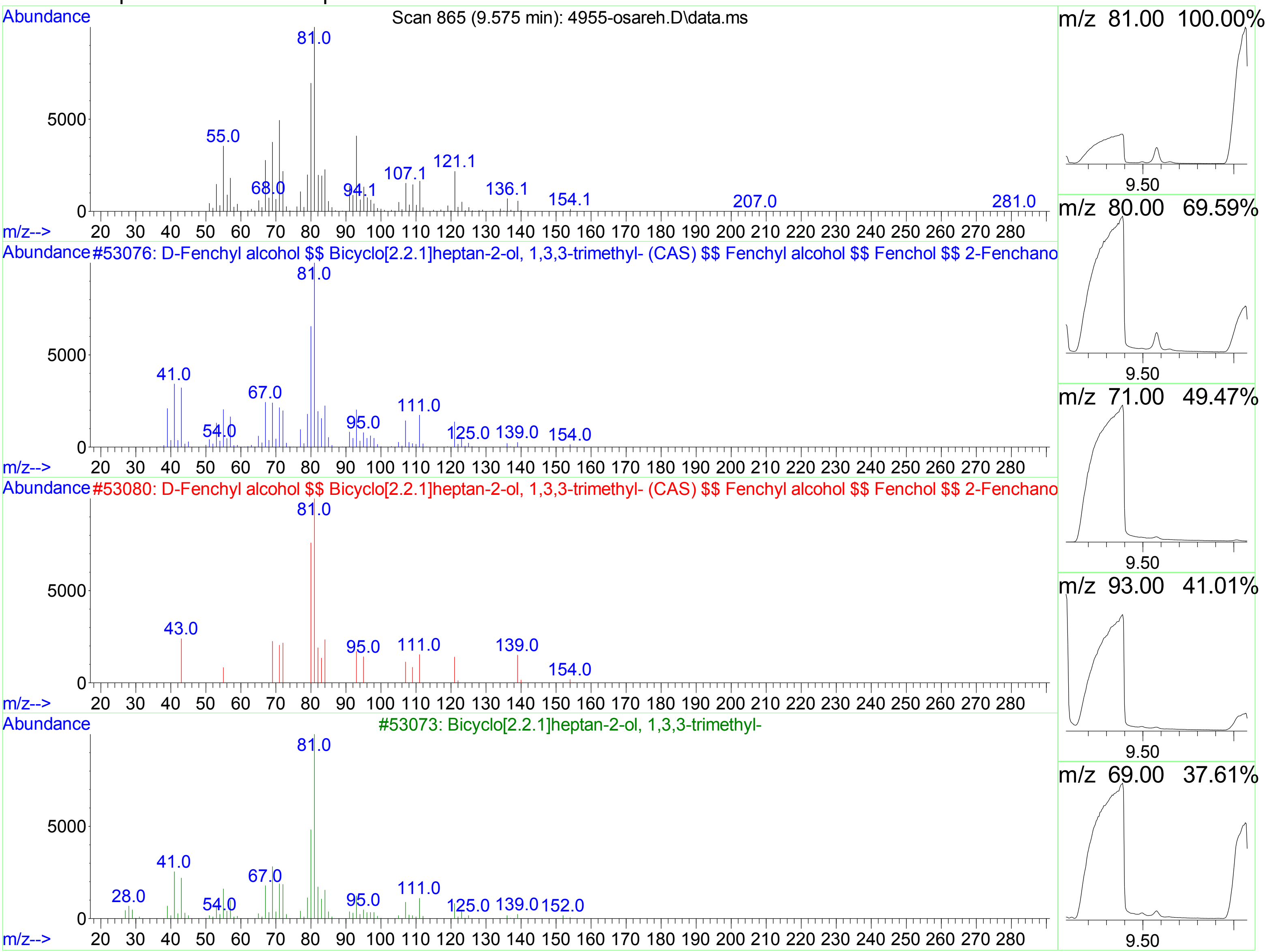

Data File: G:\ÌÒÇÑÔ 1399\99-11-18\4955-osareh.D
Sample : 4955-osareh

Peak Number: 22 at 9.575 min Area: 27489745 Area % 0.18

The 3 best hits from each library. Ref\# CAS\# Qual

E:\Database\wiley7n.l

1	D-Fenchyl alcohol $$ Bicyclo[2.2...	53076	001632-73-1	95
2	D-Fenchyl alcohol $$ Bicyclo[2.2...	53080	001632-73-1	76
3	Bicyclo[2.2.1]heptan-2-ol, 1,3,3...	53073	001632-73-1	70

Unknown Spectrum based on Apex

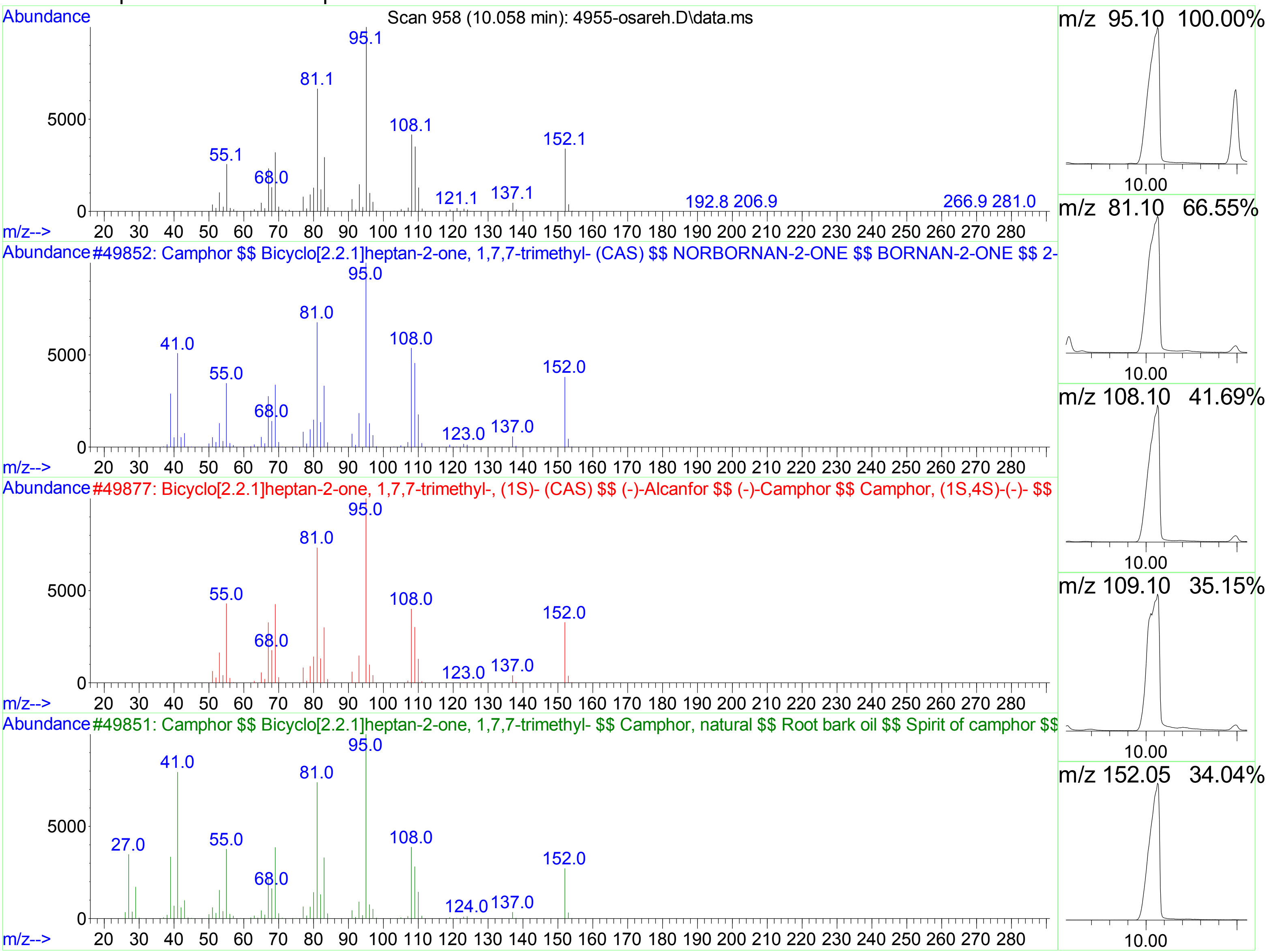

Data File: G:\ÌÒÇÑÔ 1399\99-11-18\4955-osareh.D
Sample : 4955-osareh

Peak Number: 23 at 10.058 min Area: 899002928 Area % 5.86

The 3 best hits from each library.	Ref#	CAS#	Qual
E:\Database\wiley7n.l			
1 Camphor $$ Bicyclo[2.2.1]heptan-...	49852	000076-22-2	98
2 Bicyclo[2.2.1]heptan-2-one, 1,7,...	49877	000464-48-2	98
3 Camphor $$ Bicyclo[2.2.1]heptan-...	49851	000076-22-2	98

Unknown Spectrum based on Apex

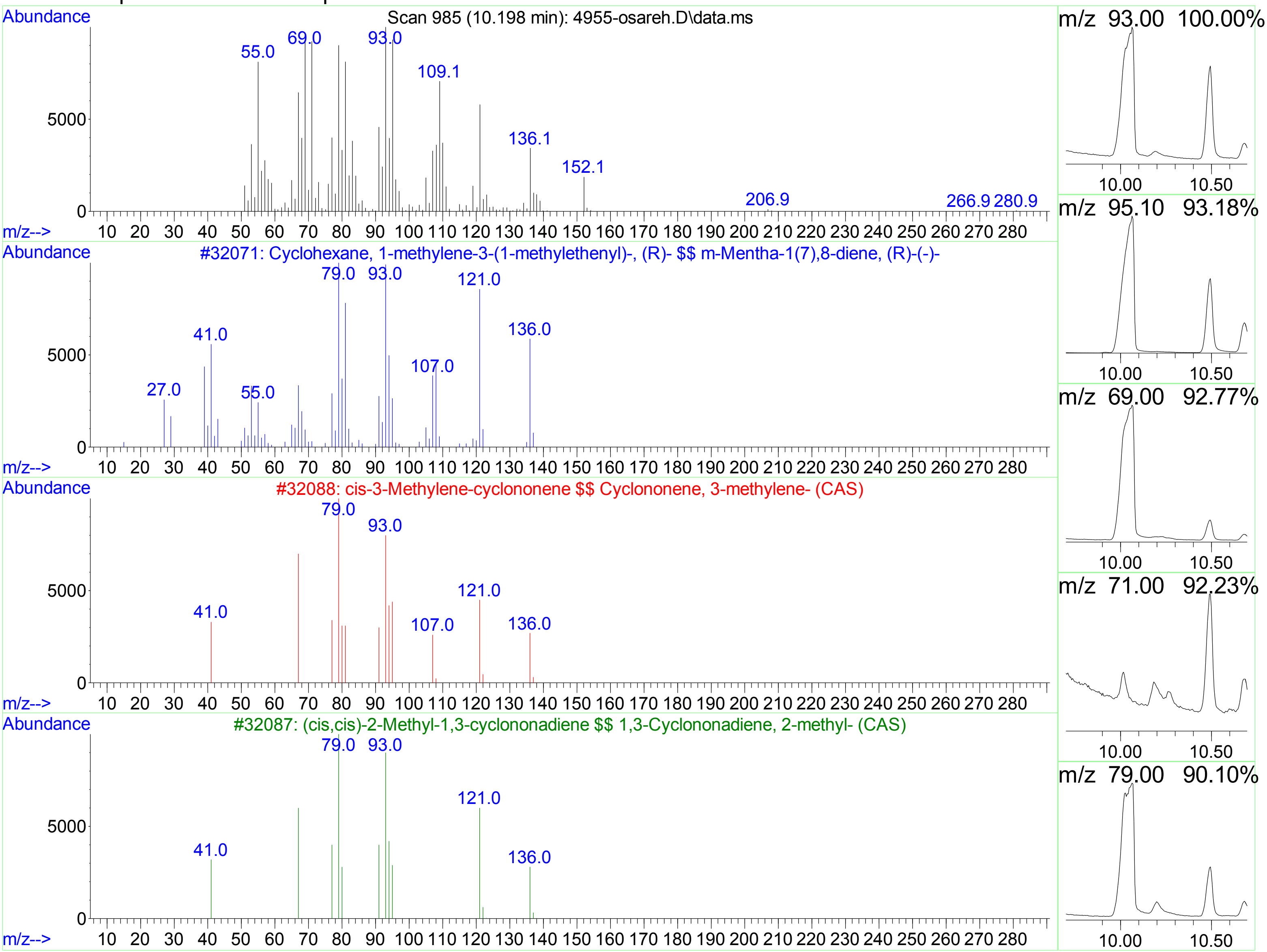

Data File: G:\ÌÒÇÑÔ 1399\99-11-18\4955-osareh.D
Sample : 4955-osareh

Peak Number: 24 at 10.198 min Area: 8822129 Area % 0.06

The 3 best hits from each library. Ref\# CAS\# Qual

E:\Database\wiley7n.l

1 Cyclohexane, 1-methylene-3-(1-me...	32071	013837-95-1	87
2 cis-3-Methylene-cyclononene $$ C...	32088	066135-92-0	86
3 (cis,cis)-2-Methyl-1,3-cyclonona...	32087	066135-93-1	83

Library Search Report - ChemStation Integrator

Unknown Spectrum based on Apex

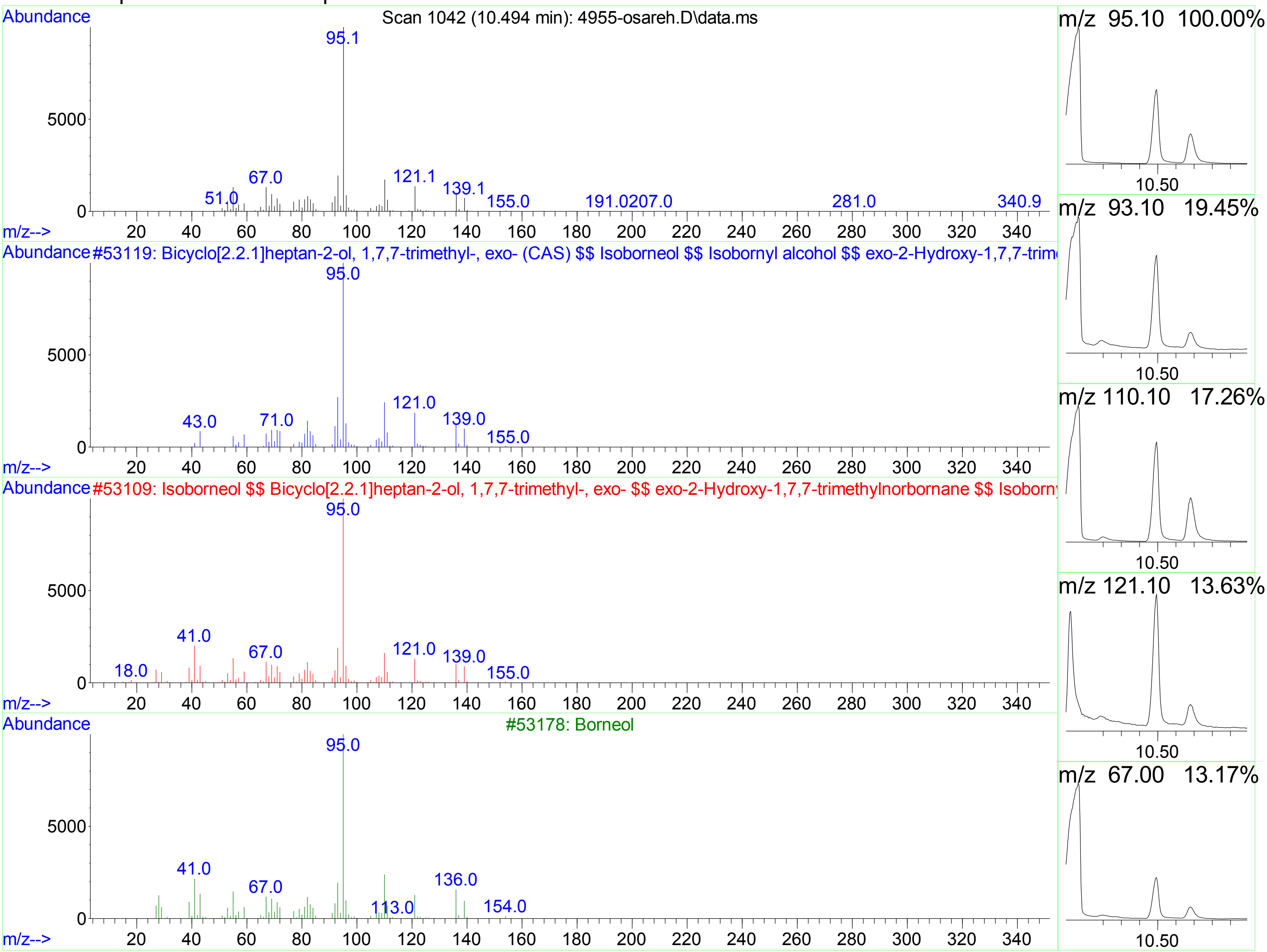

Data File: G:\ÌÒÇÑÔ 1399\99-11-18\4955-osareh.D
Sample : 4955-osareh

Peak Number: 25 at 10.494 min Area: 163913175 Area % 1.07

The 3 best hits from each library.	Ref#	CAS#	Qual
E:\Database\wiley7n.l			
1 Bicyclo[2.2.1]heptan-2-ol, 1,7,7...	53119	000124-76-5	95
2 Isoborneol $$ Bicyclo[2.2.1]hept...	53109	000124-76-5	95
3 Borneol	53178	010385-78-1	94

Unknown Spectrum based on Apex

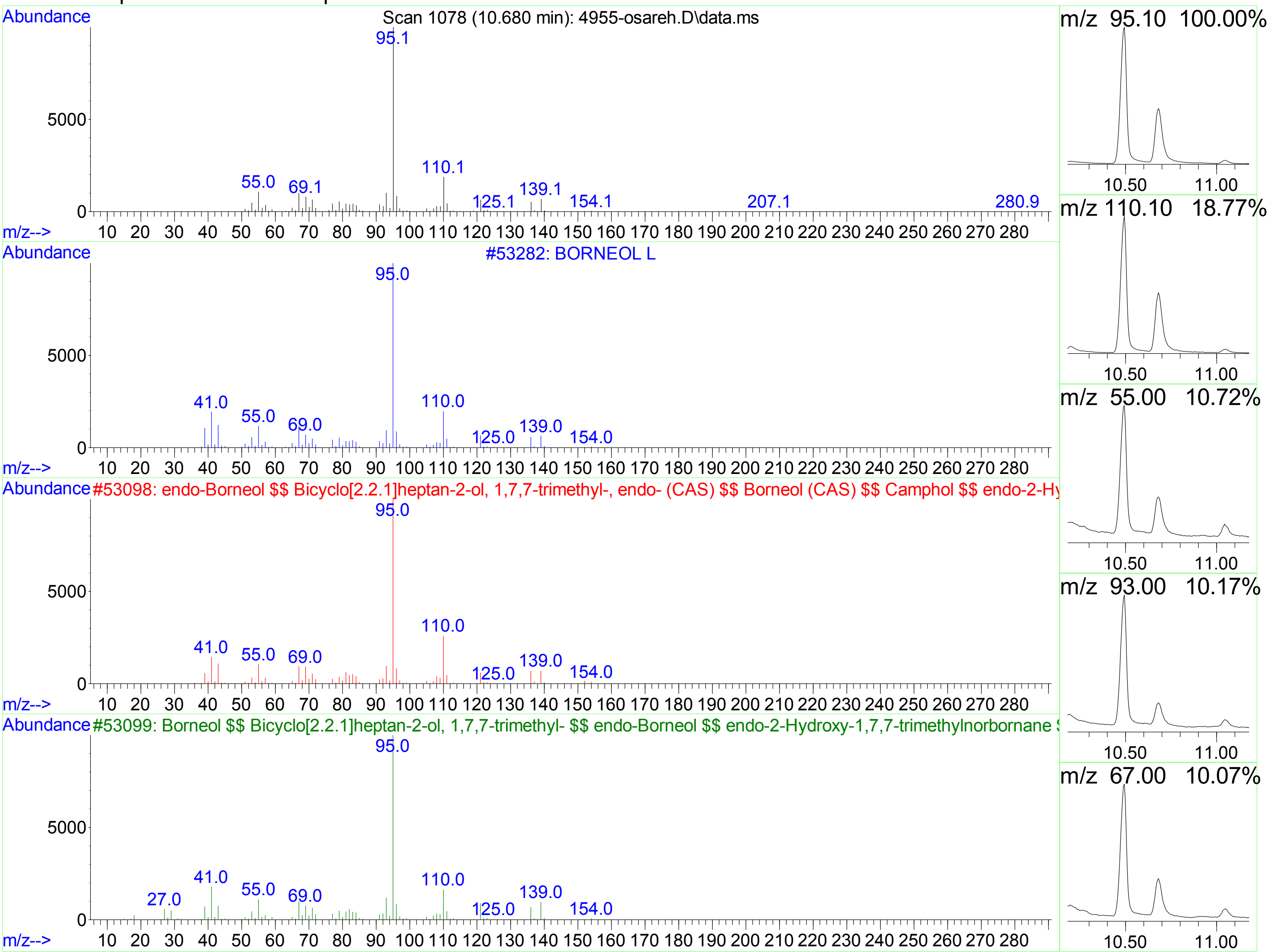

Data File: G:\ÌÒÇÑÔ 1399\99-11-18\4955-osareh.D
Sample : 4955-osareh

Peak Number: 26 at 10.680 min Area: 60496691 Area % 0.39

The 3 best hits from each library.	Ref#	CAS#	Qual
E:\Database\wiley7n.l			
1 BORNEOL L	53282	000464-45-9	97
2 endo-Borneol $$ Bicyclo[2.2.1]he...	53098	000507-70-0	94
3 Borneol $$ Bicyclo[2.2.1]heptan-...	53099	000507-70-0	91

Unknown Spectrum based on Apex

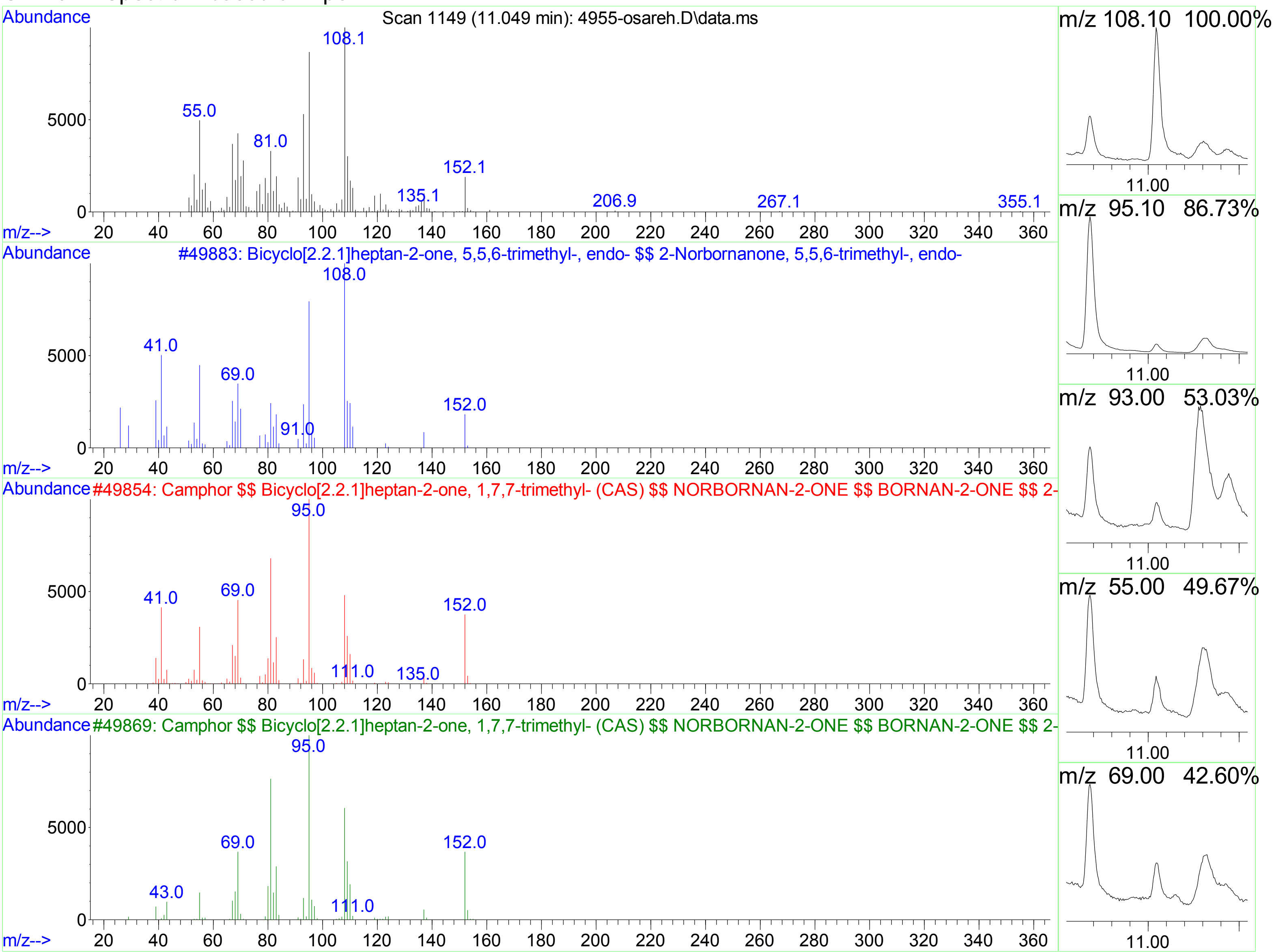

Data File: G:\ÌÒÇÑÔ 1399\99-11-18\4955-osareh.D
Sample : 4955-osareh

Peak Number: 27 at 11.049 min Area: 8699717 Area % 0.06

The 3 best hits from each library.	Ref#	CAS#	Qual
E:\Database\wiley7n.l			
1 Bicyclo[2.2.1]heptan-2-one, 5,5,...	49883	003767-44-0	91
2 Camphor $$ Bicyclo[2.2.1]heptan-...	49854	000076-22-2	64
3 Camphor $$ Bicyclo[2.2.1]heptan-...	49869	000076-22-2	64

Library Search Report - ChemStation Integrator

Unknown Spectrum based on Apex

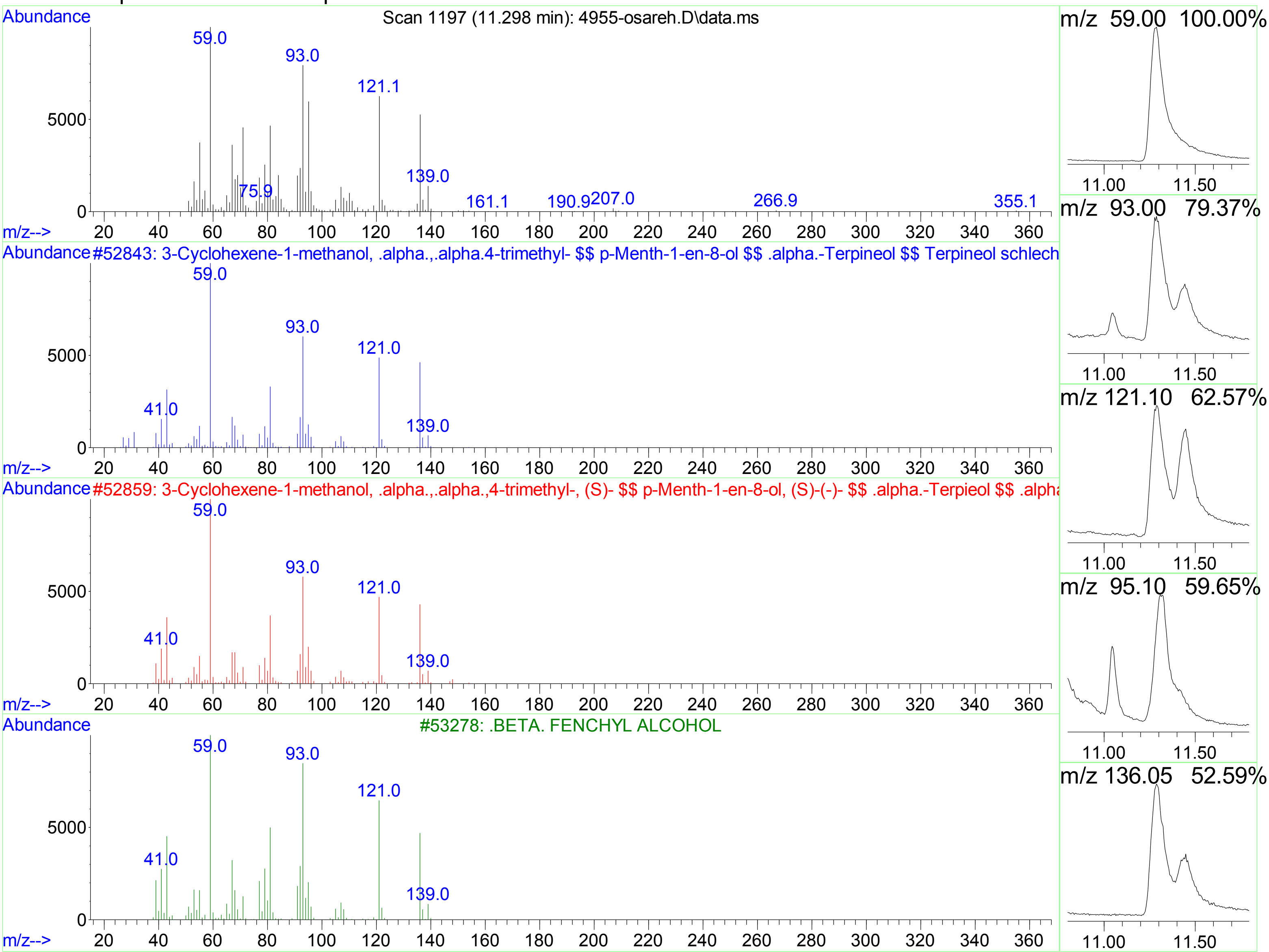

Data File: G:\ÌÒÇÑÔ 1399\99-11-18\4955-osareh.D
Sample : 4955-osareh

Peak Number: 28 at 11.298 min Area: 37017181 Area % 0.24

The 3 best hits from each library.	Ref#	CAS#	Qual
E:\Database\wiley7n.l			
1 3-Cyclohexene-1-methanol, .alpha...	52843	000098-55-5	95
2 3-Cyclohexene-1-methanol, .alpha...	52859	010482-56-1	93
3 .BETA. FENCHYL ALCOHOL	53278	000470-08-6	70

Unknown Spectrum based on Apex

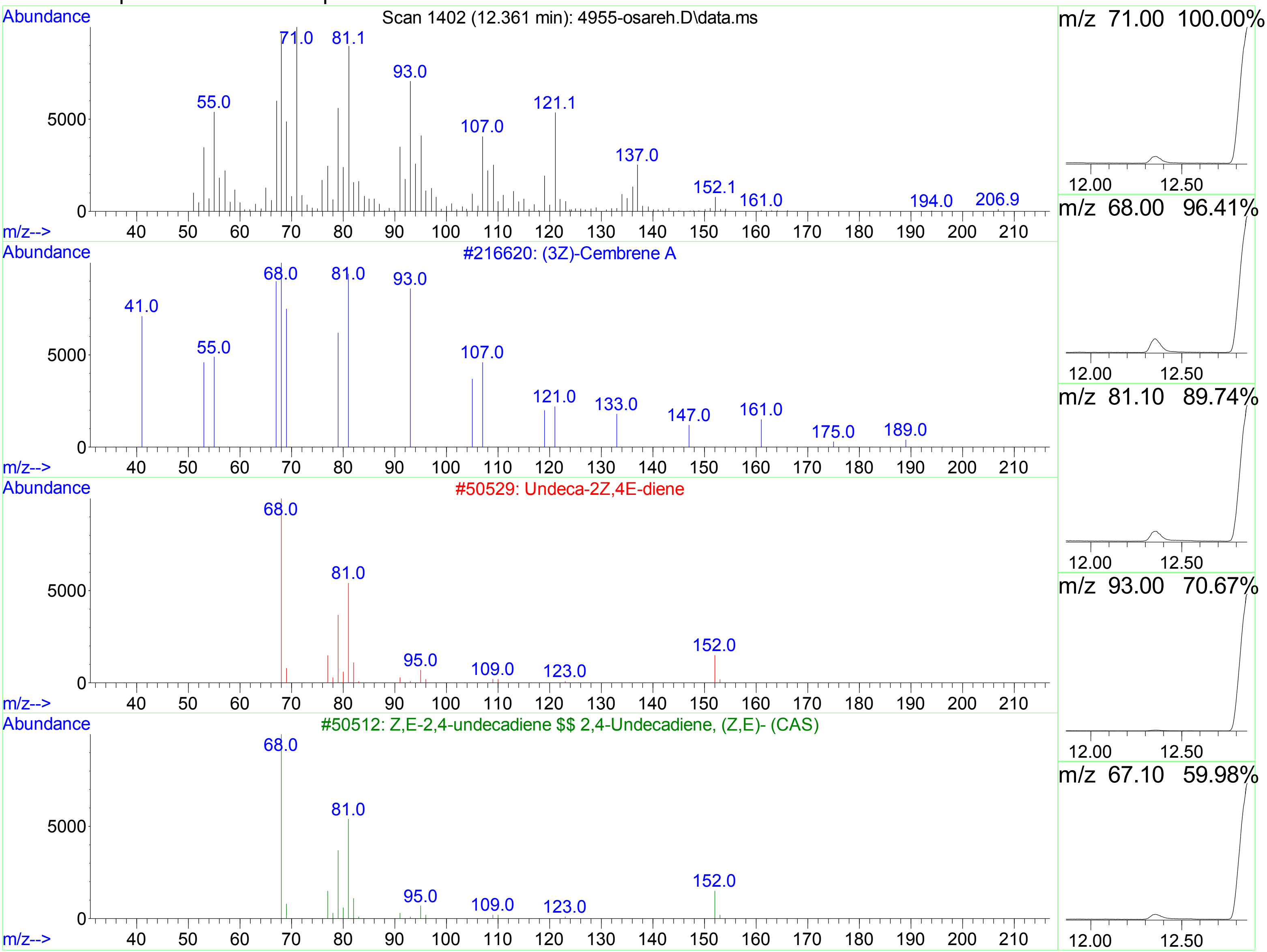

Data File: G:\ÌÒÇÑÔ 1399\99-11-18\4955-osareh.D
Sample : 4955-osareh

Peak Number: 29 at 12.361 min Area: 21897132 Area % 0.14

The 3 best hits from each library.	Ref#	CAS#	Qual
E:\Database\wiley7n.l			
1 (3Z)-Cembrene A	216620	071213-92-8	43
2 Undeca-2Z,4E-diene	50529	000000-00-0	38
3 Z,E-2,4-undecadiene $$ 2,4-Undec...	50512	066717-37-1	38

Unknown Spectrum based on Apex

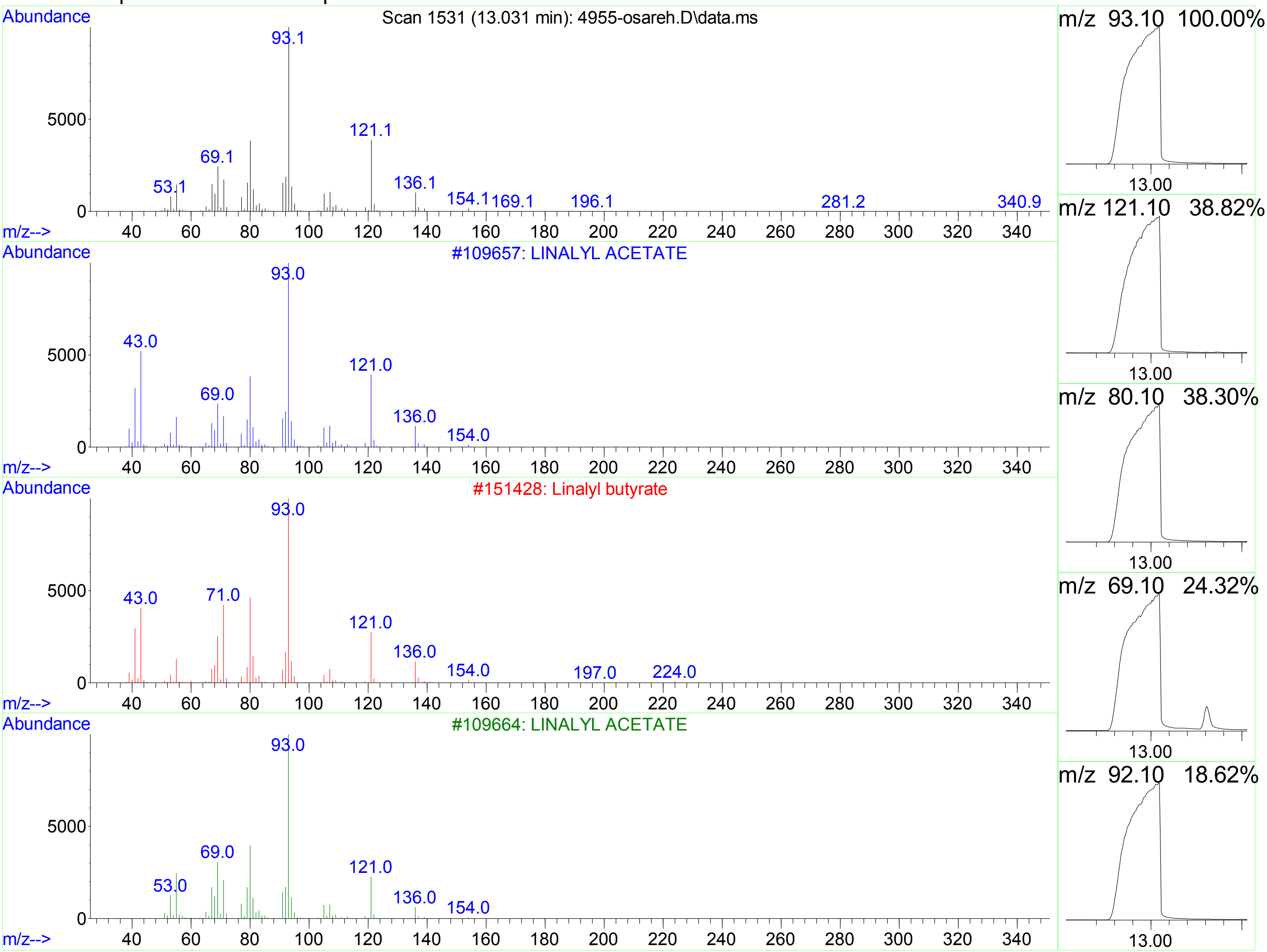

Data File: G:\ÌÒÇÑÔ 1399\99-11-18\4955-osareh.D
Sample : 4955-osareh

Peak Number: 30 at 13.031 min Area: 3696992825 Area % 24.09

The 3 best hits from each library.	Ref#	CAS#	Qual
E:\Database\wiley7n.l			
1 LINALYL ACETATE	109657	000115-95-7	91
2 Linalyl butyrate	151428	000078-36-4	90
3 LINALYL ACETATE	109664	000115-95-7	87

Unknown Spectrum based on Apex

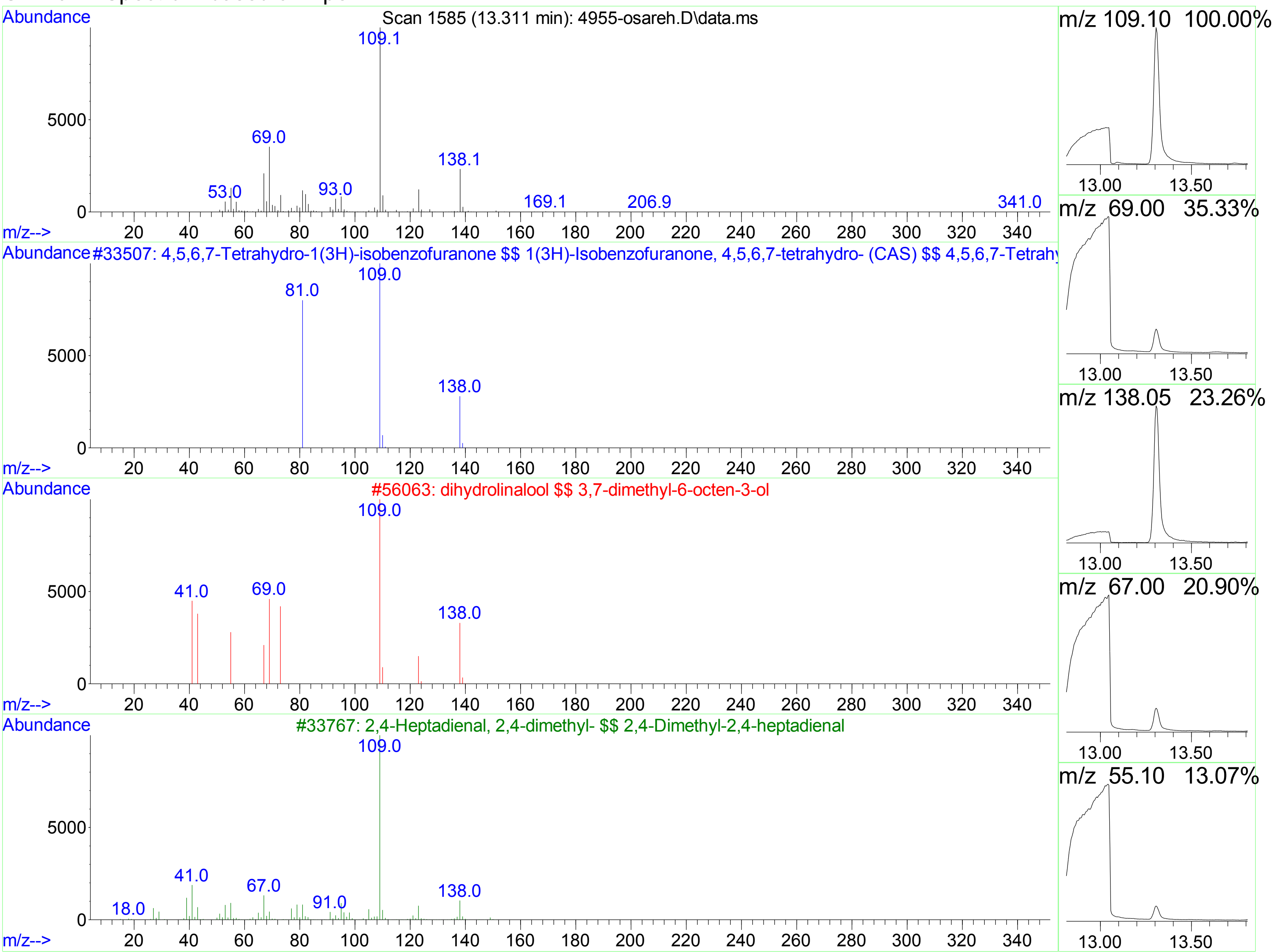

Data File: G:\ÌÒÇÑÔ 1399\99-11-18\4955-osareh.D
Sample : 4955-osareh

Peak Number: 31 at 13.311 min Area: 61127925 Area % 0.40

The 3 best hits from each library. Ref\# CAS\# Qual

E:\Database\wiley7n.l

1	4,5,6,7-Tetrahydro-1(3H)-isobenz...	33507	066309-76-0	64
2	dihydrolinalool $$ 3,7-dimethyl-...	56063	000000-00-0	64
3	2,4-Heptadienal, 2,4-dimethyl- $...	33767	042452-48-2	50

Library Search Report - ChemStation Integrator

Unknown Spectrum based on Apex

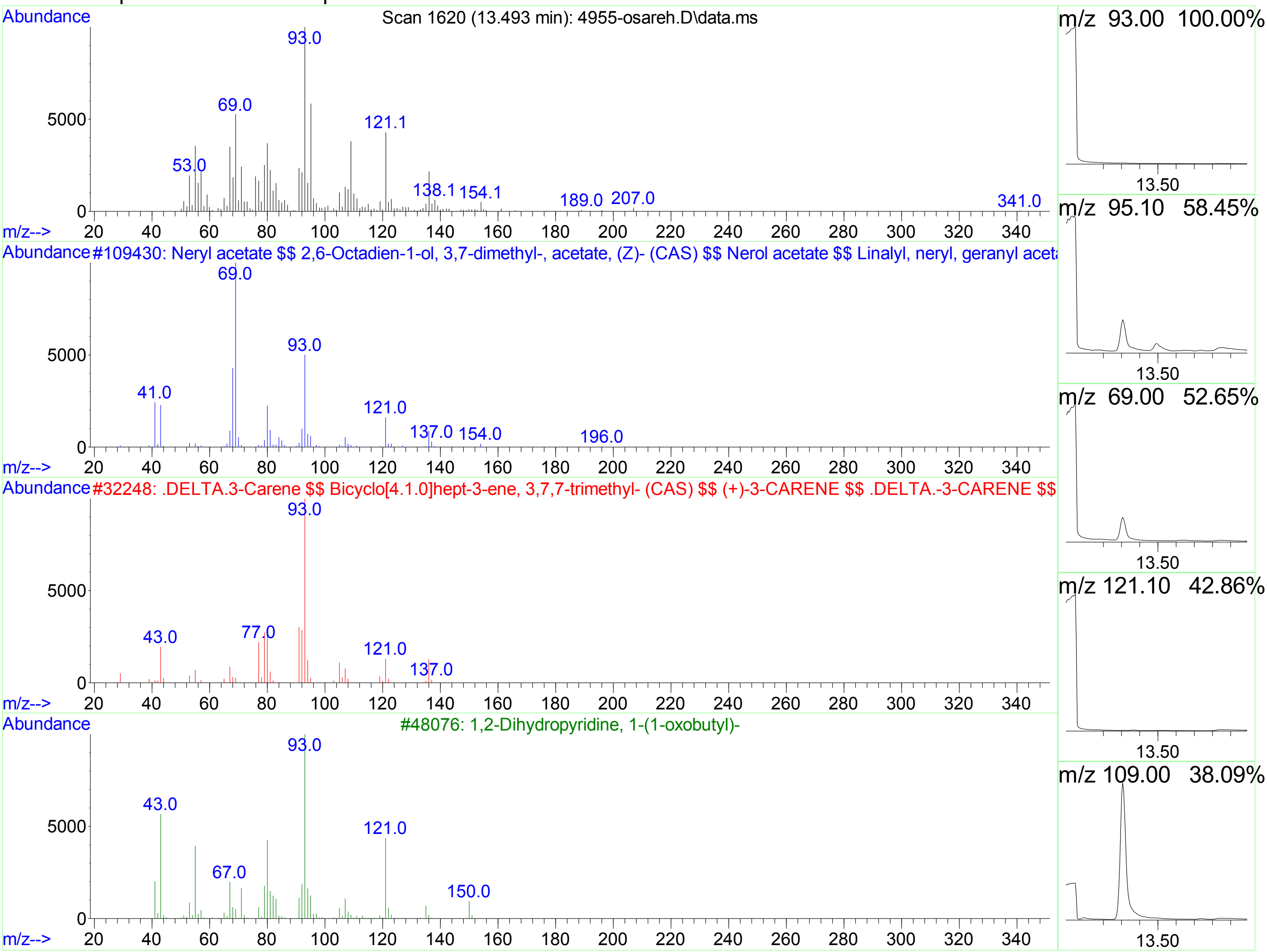

Data File: G:\ÌÒÇÑÔ 1399\99-11-18\4955-osareh.D
Sample : 4955-osareh

Peak Number: 32 at 13.493 min Area: 2193185 Area % 0.01

The 3 best hits from each library.	Ref#	CAS#	Qual
E:\Database\wiley7n.l			
1 Neryl acetate $$ 2,6-Octadien-1-...	109430	000141-12-8	60
2 .DELTA.3-Carene $$ Bicyclo[4.1.0...	32248	013466-78-9	55
3 1,2-Dihydropyridine, 1-(1-oxobut...	48076	000000-00-0	55

Library Search Report - ChemStation Integrator

Unknown Spectrum based on Apex

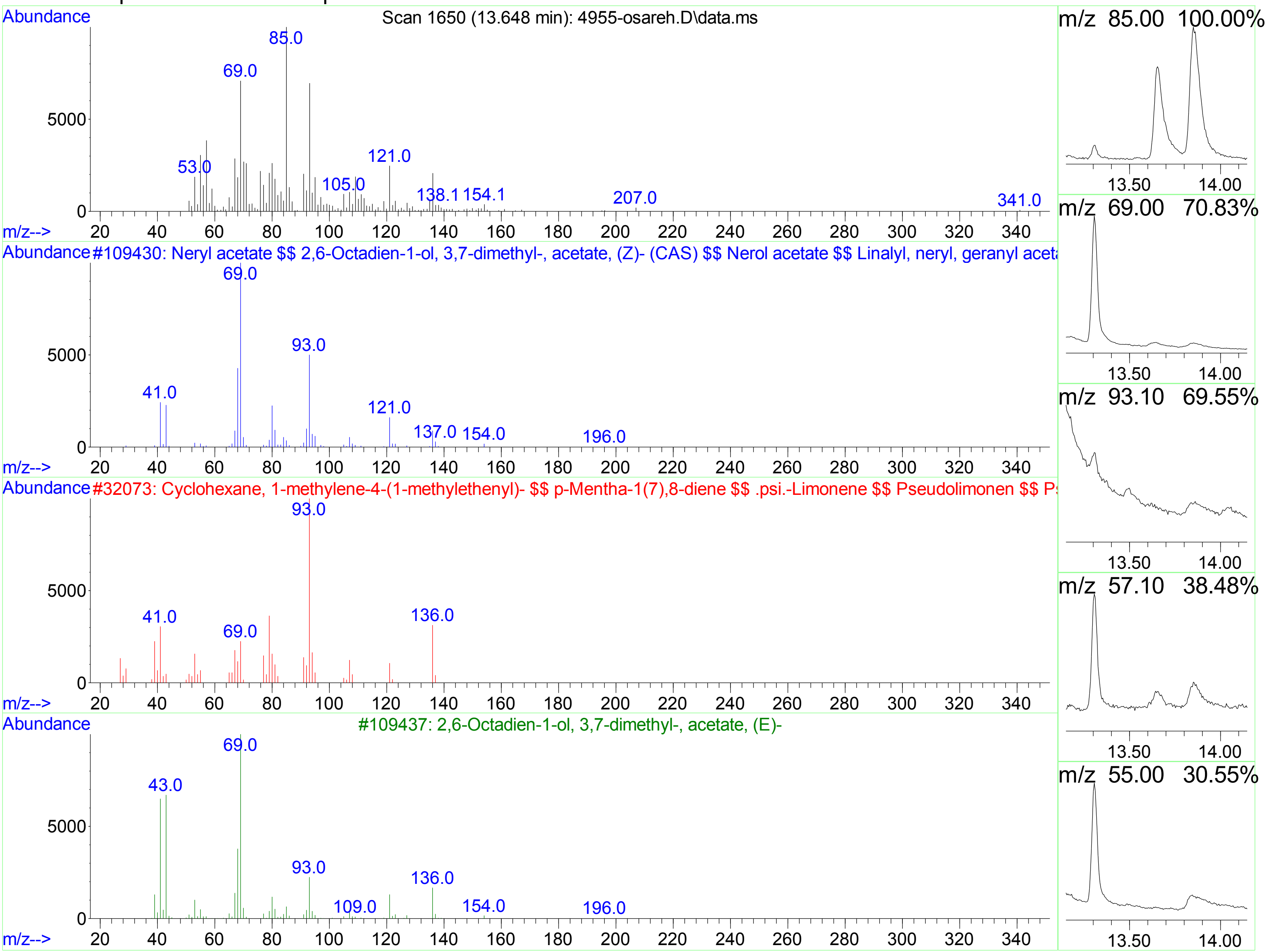

Data File: G:\ÌÒÇÑÔ 1399\99-11-18\4955-osareh.D
Sample : 4955-osareh

Peak Number: 33 at 13.648 min Area: 2342096 Area % 0.02

The 3 best hits from each library. Ref# CAS# Qual

E:\Database\wiley7n.l

1	Neryl acetate $$ 2,6-Octadien-1-...	109430	000141-12-8	42
2	Cyclohexane, 1-methylene-4-(1-me...	32073	000499-97-8	42
3	2,6-Octadien-1-ol, 3,7-dimethyl-...	109437	000105-87-3	42

Library Search Report - ChemStation Integrator

Unknown Spectrum based on Apex

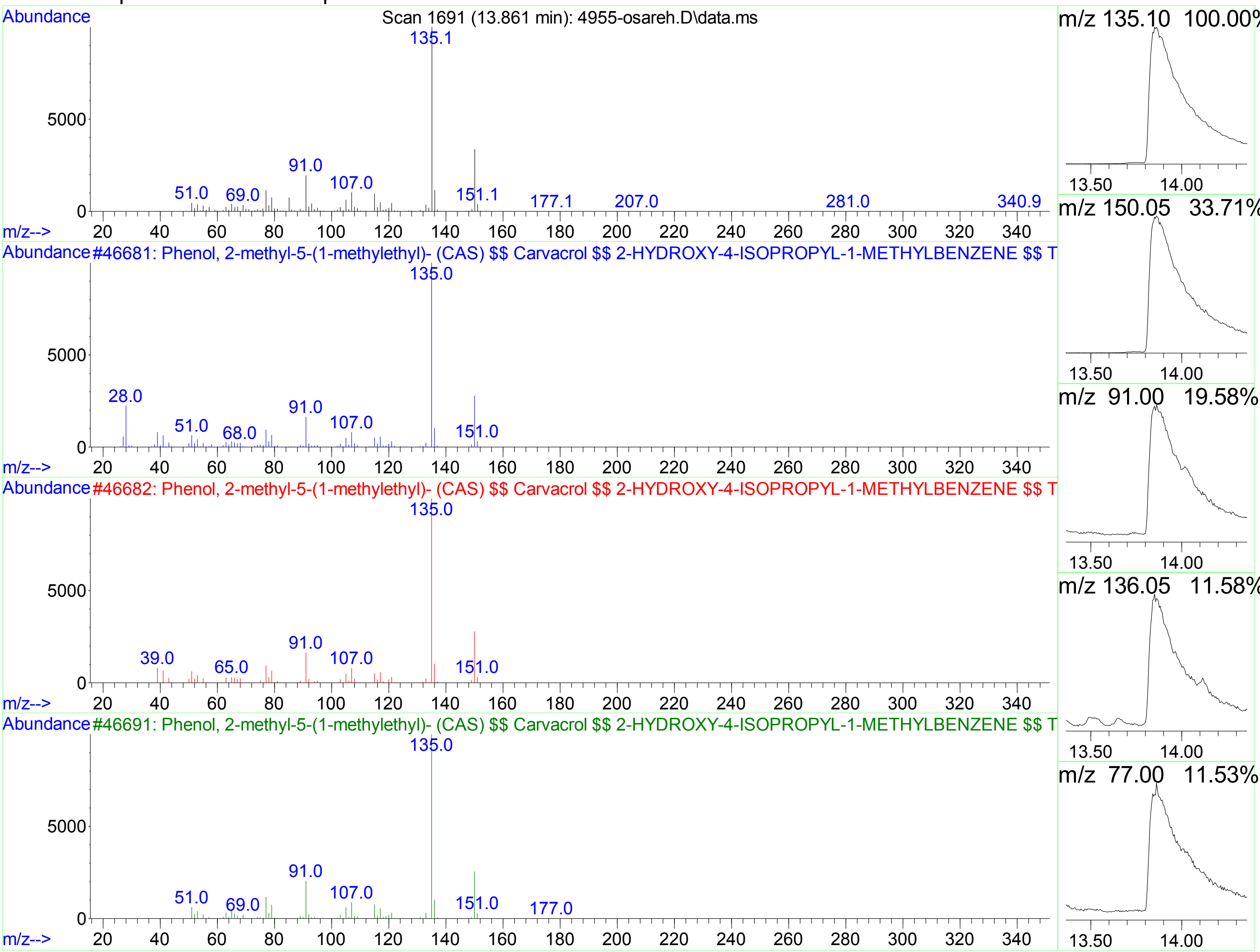

Data File: G:\ÌÒÇÑÔ 1399\99-11-18\4955-osareh.D
Sample : 4955-osareh

Peak Number: 34 at 13.861 min Area: 236808892 Area % 1.54

The 3 best hits from each library.	Ref#	CAS#	Qual
E:\Database\wiley7n.l			
1 Phenol, 2-methyl-5-(1-methylethy...	46681	000499-75-2	95
2 Phenol, 2-methyl-5-(1-methylethy...	46682	000499-75-2	95
3 Phenol, 2-methyl-5-(1-methylethy...	46691	000499-75-2	95

Unknown Spectrum based on Apex

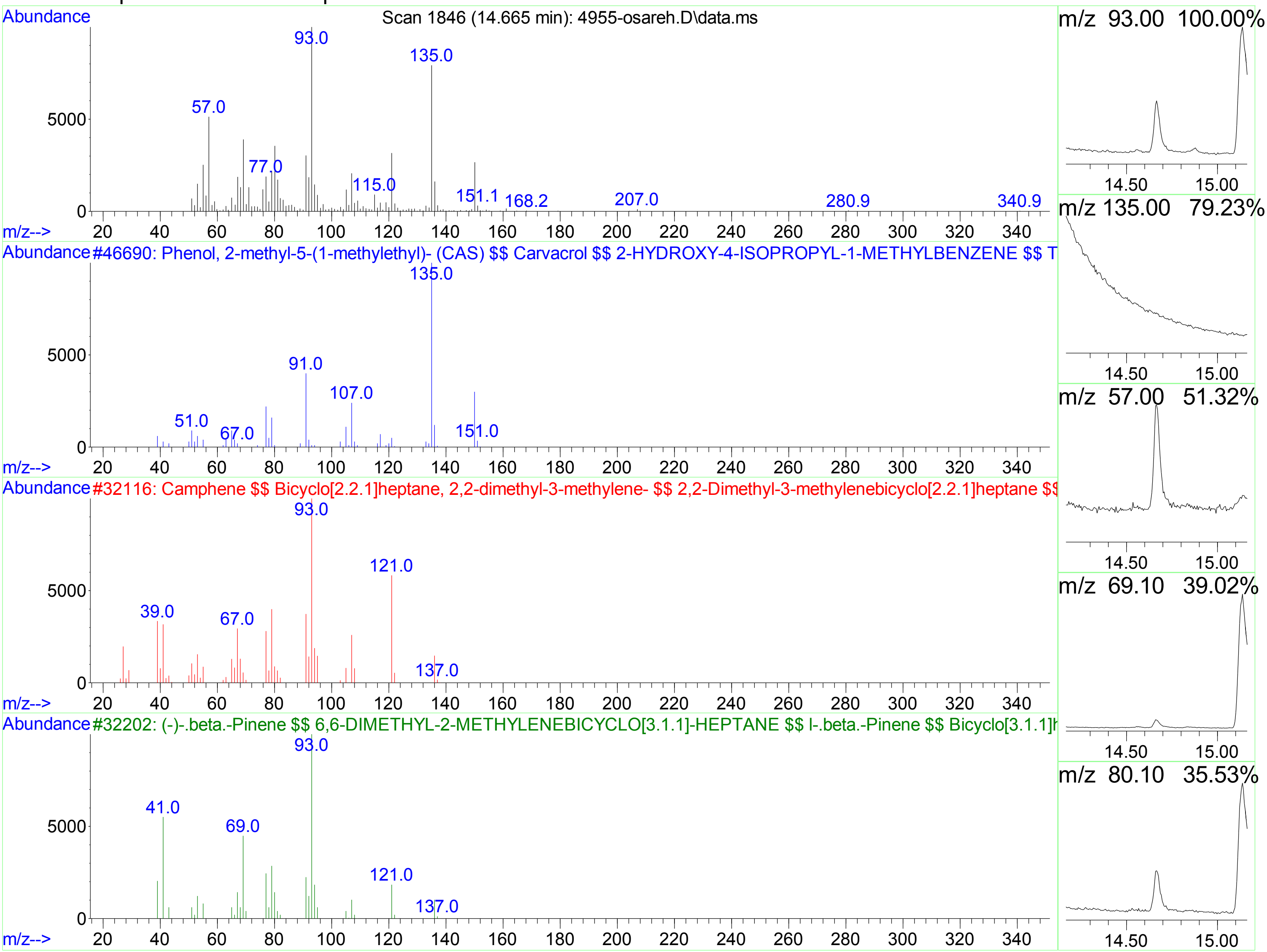

Data File: G:\ÌÒÇÑÔ 1399\99-11-18\4955-osareh.D
Sample : 4955-osareh

Peak Number: 35 at 14.665 min Area: 5650427 Area % 0.04

The 3 best hits from each library.	Ref#	CAS#	Qual
E:\Database\wiley7n.l			
1 Phenol, 2-methyl-5-(1-methylethy...	46690	000499-75-2	86
2 Camphene $$ Bicyclo[2.2.1]heptan...	32116	000079-92-5	80
3 (-)-.beta.-Pinene $$ 6,6-DIMETHY...	32202	018172-67-3	50

Library Search Report - ChemStation Integrator

Unknown Spectrum based on Apex

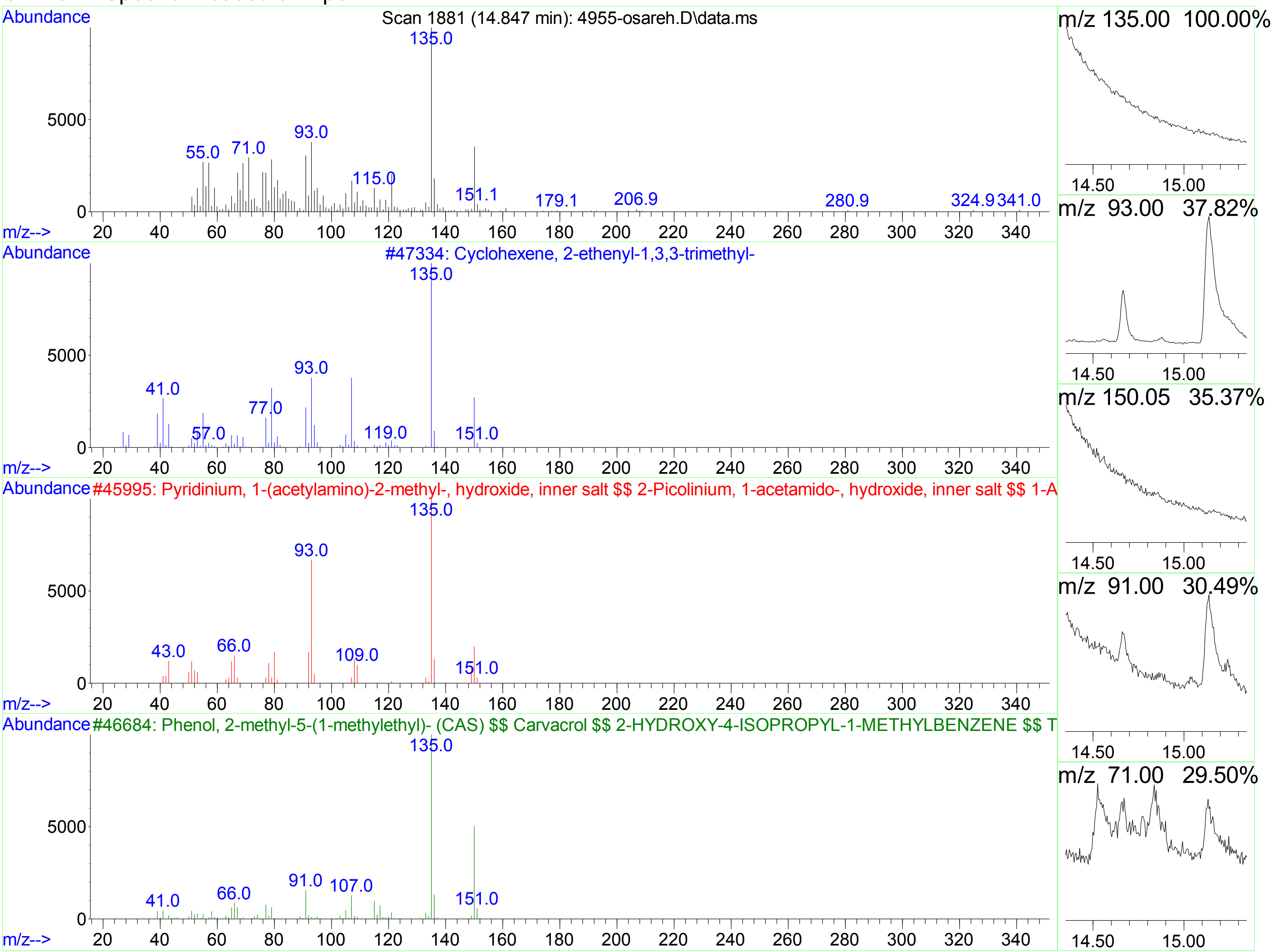

Data File: G:\ÌÒÇÑÔ 1399\99-11-18\4955-osareh.D
Sample : 4955-osareh

Peak Number: 36 at 14.847 min Area: 2104968 Area % 0.01

The 3 best hits from each library. Ref\# CAS\# Qual

E:\Database\wiley7n.l

1 Cyclohexene, 2-ethenyl-1,3,3-tri... 47334 005293-90-3 64
2 Pyridinium, 1-(acetylamino)-2-me... 45995 007584-27-2 62
3 Phenol, 2-methyl-5-(1-methylethy... 46684 000499-75-2 60

Unknown Spectrum based on Apex

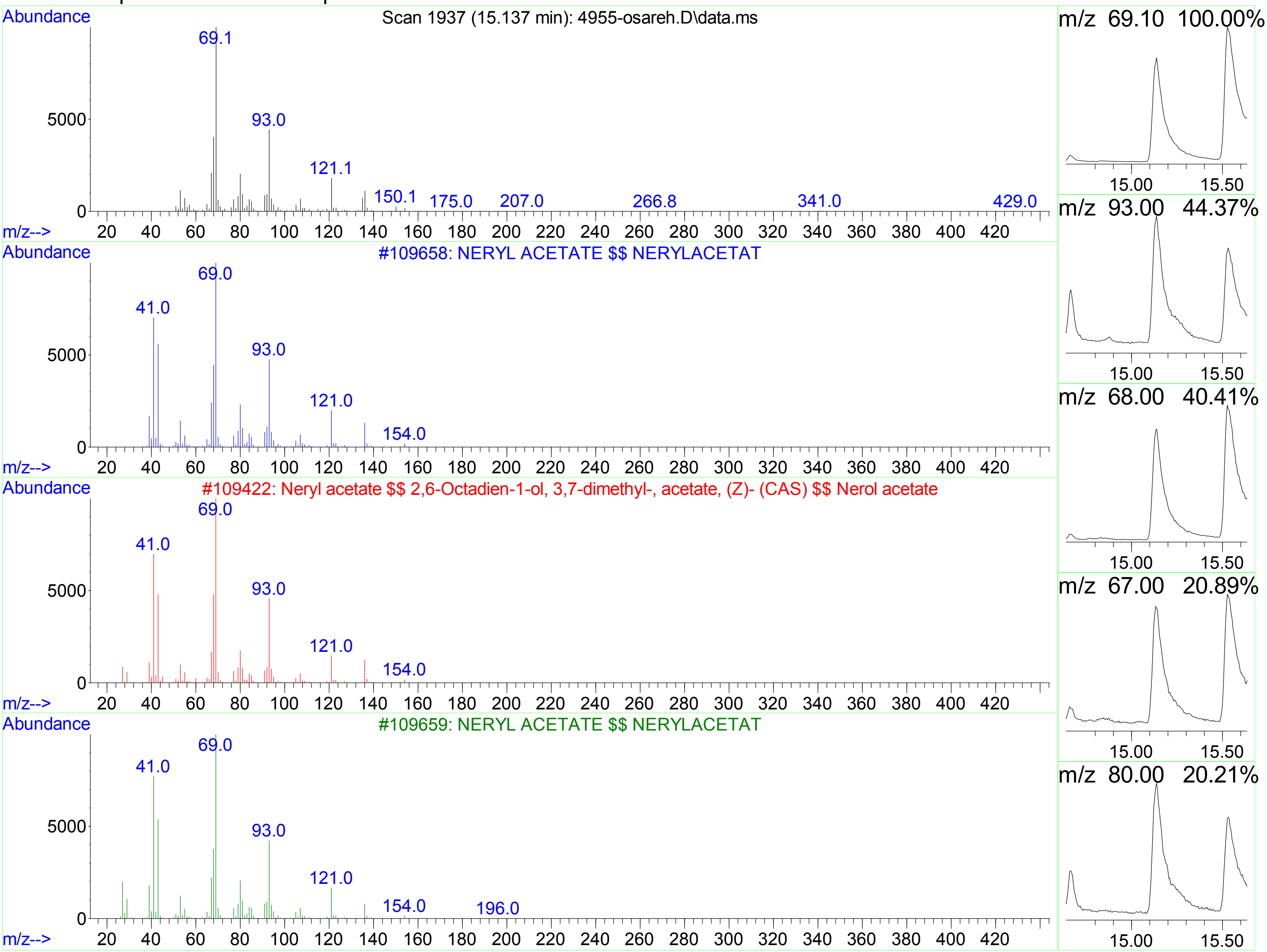

Data File: G:\ÌÒÇÑÔ 1399\99-11-18\4955-osareh.D
Sample : 4955-osareh

Peak Number: 37 at 15.137 min Area: 54682545 Area % 0.36

The 3 best hits from each library.	Ref#	CAS#	Qual
E:\Database\wiley7n.l			
1 NERYL ACETATE $$ NERYLACETAT	109658	000141-12-8	91
2 Neryl acetate $$ 2,6-Octadien-1-...	109422	000141-12-8	91
3 NERYL ACETATE $$ NERYLACETAT	109659	000141-12-8	91

Unknown Spectrum based on Apex

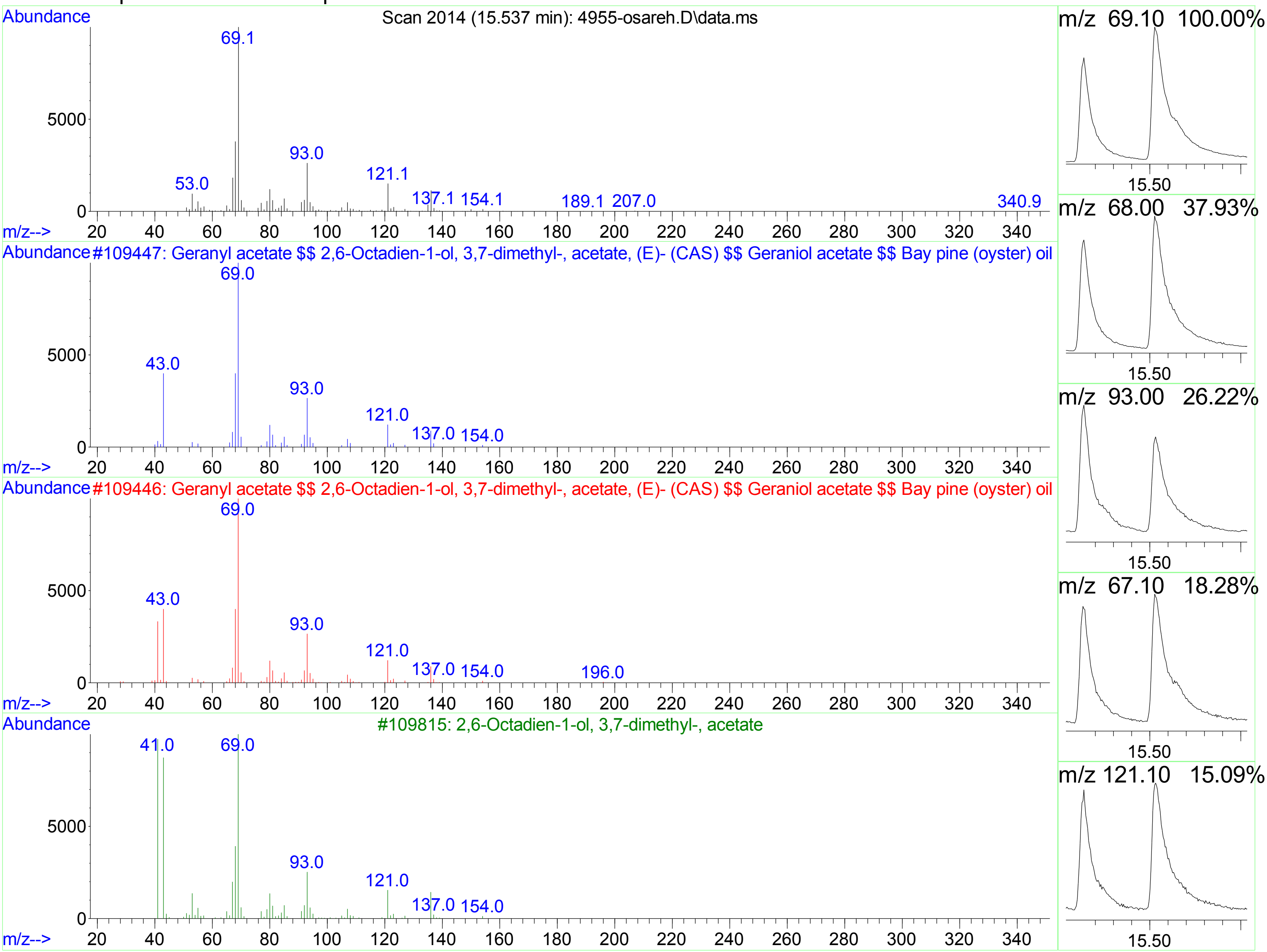

Data File: G:\ÌÒÇÑÔ 1399\99-11-18\4955-osareh.D
Sample : 4955-osareh

Peak Number: 38 at 15.537 min Area: 36188201 Area % 0.24

The 3 best hits from each library.	Ref#	CAS#	Qual
E:\Database\wiley7n.l			
1 Geranyl acetate $$ 2,6-Octadien-...	109447	000105-87-3	91
2 Geranyl acetate $$ 2,6-Octadien-...	109446	000105-87-3	91
3 2,6-Octadien-1-ol, 3,7-dimethyl-...	109815	016409-44-2	91

Unknown Spectrum based on Apex

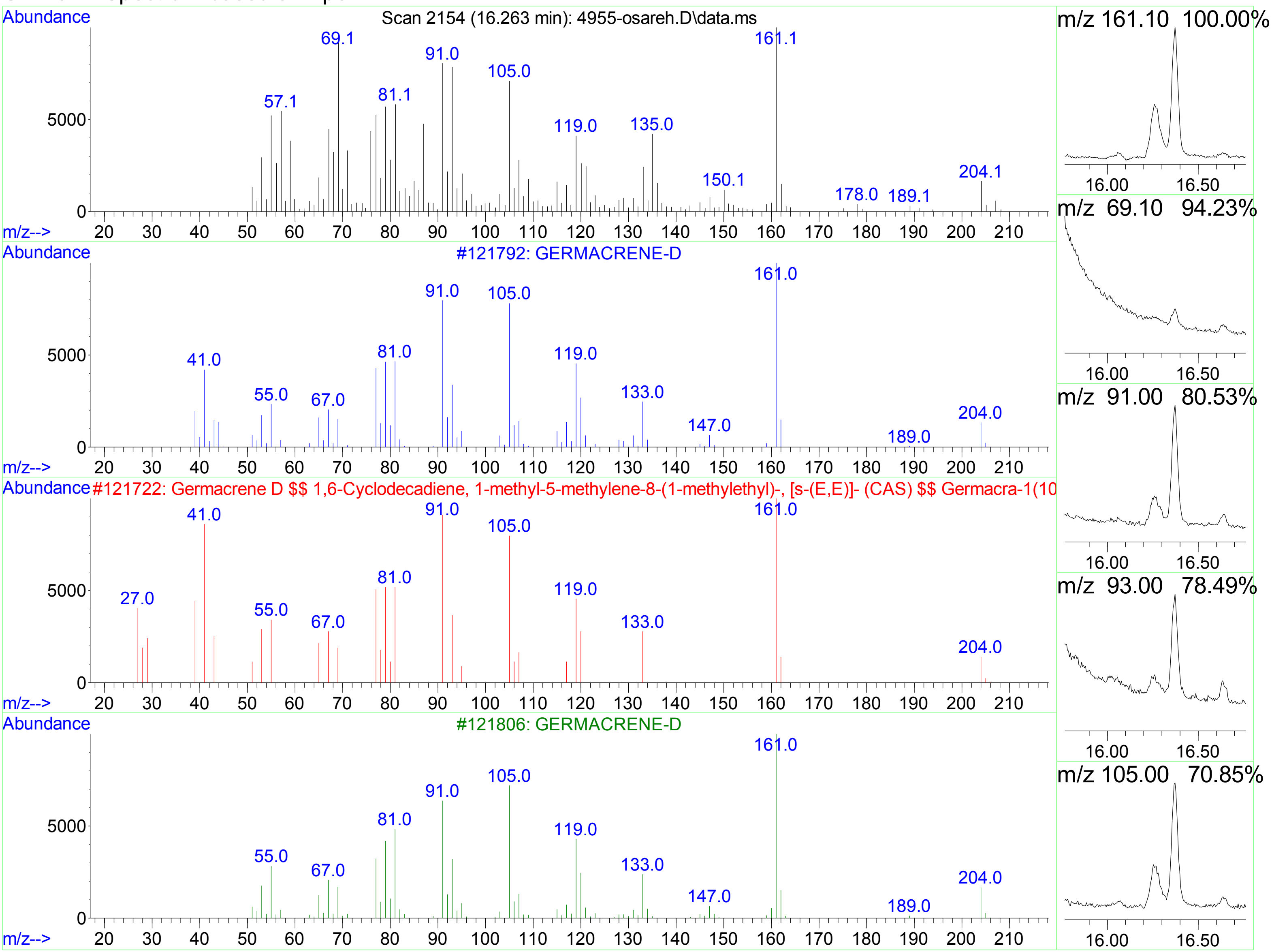

Data File: G:\ÌÒÇÑÔ 1399\99-11-18\4955-osareh.D
Sample : 4955-osareh

Peak Number: 39 at 16.263 min Area: 2278671 Area % 0.01

The 3 best hits from each library.	Ref\#	CAS\#	Qual
E:\Database\wiley7n.l			
1 GERMACRENE-D	121792	023986-74-5	93
2 Germacrene D $$ 1,6-Cyclodecadie...	121722	023986-74-5	93
3 GERMACRENE-D	121806	023986-74-5	93

Library Search Report - ChemStation Integrator

Unknown Spectrum based on Apex

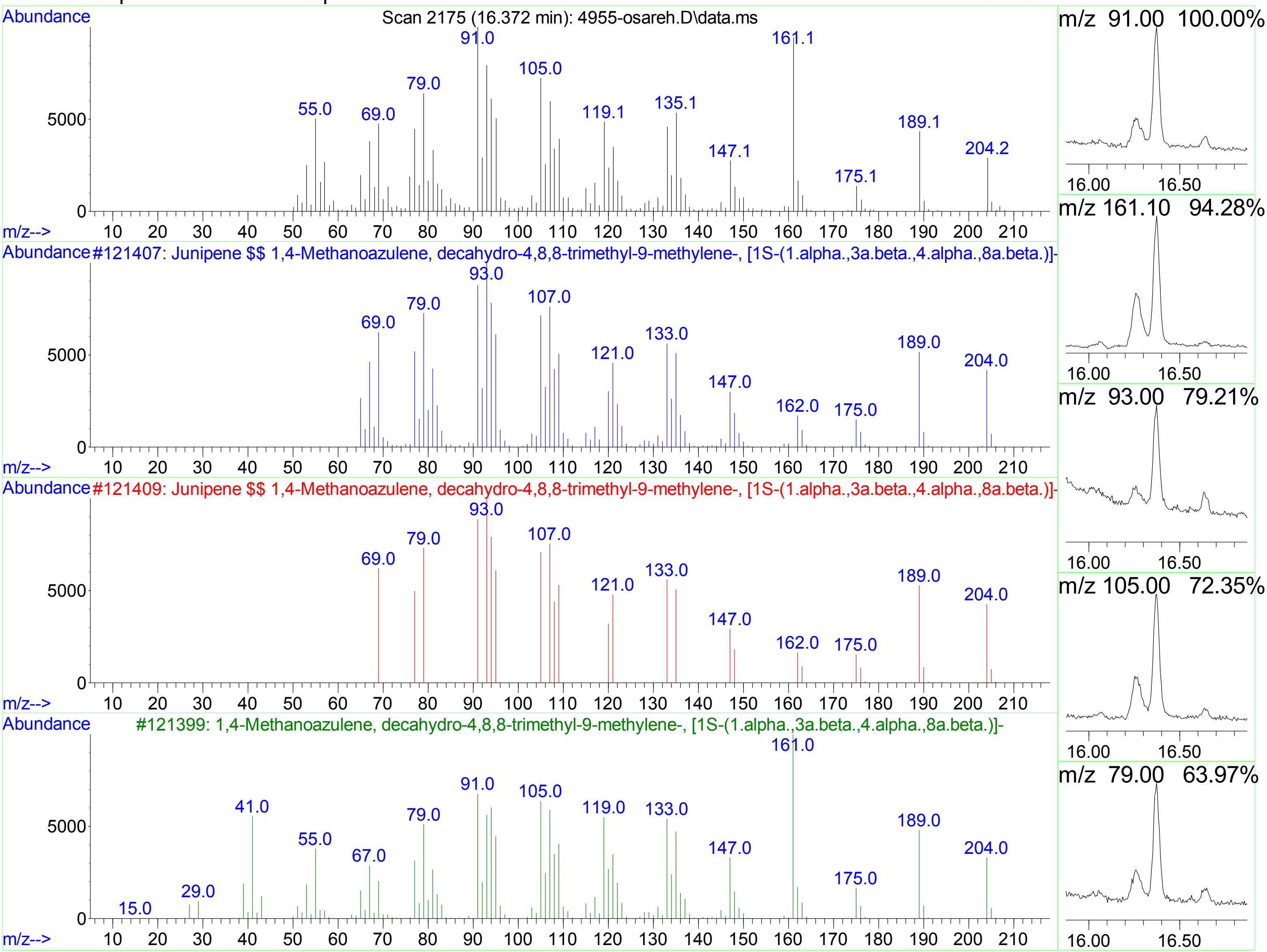

Data File: G:\ÌÒÇÑÔ 1399\99-11-18\4955-osareh.D
Sample : 4955-osareh

Peak Number: 40 at 16.372 min Area: 11372392 Area % 0.07

The 3 best hits from each library.	Ref#	CAS#	Qual
E:\Database\wiley7n.l			
1 Junipene $$ 1,4-Methanoazulene, ...	121407	000475-20-7	99
2 Junipene $$ 1,4-Methanoazulene, ...	121409	000475-20-7	99
3 1,4-Methanoazulene, decahydro-4,...	121399	000475-20-7	99

Unknown Spectrum based on Apex

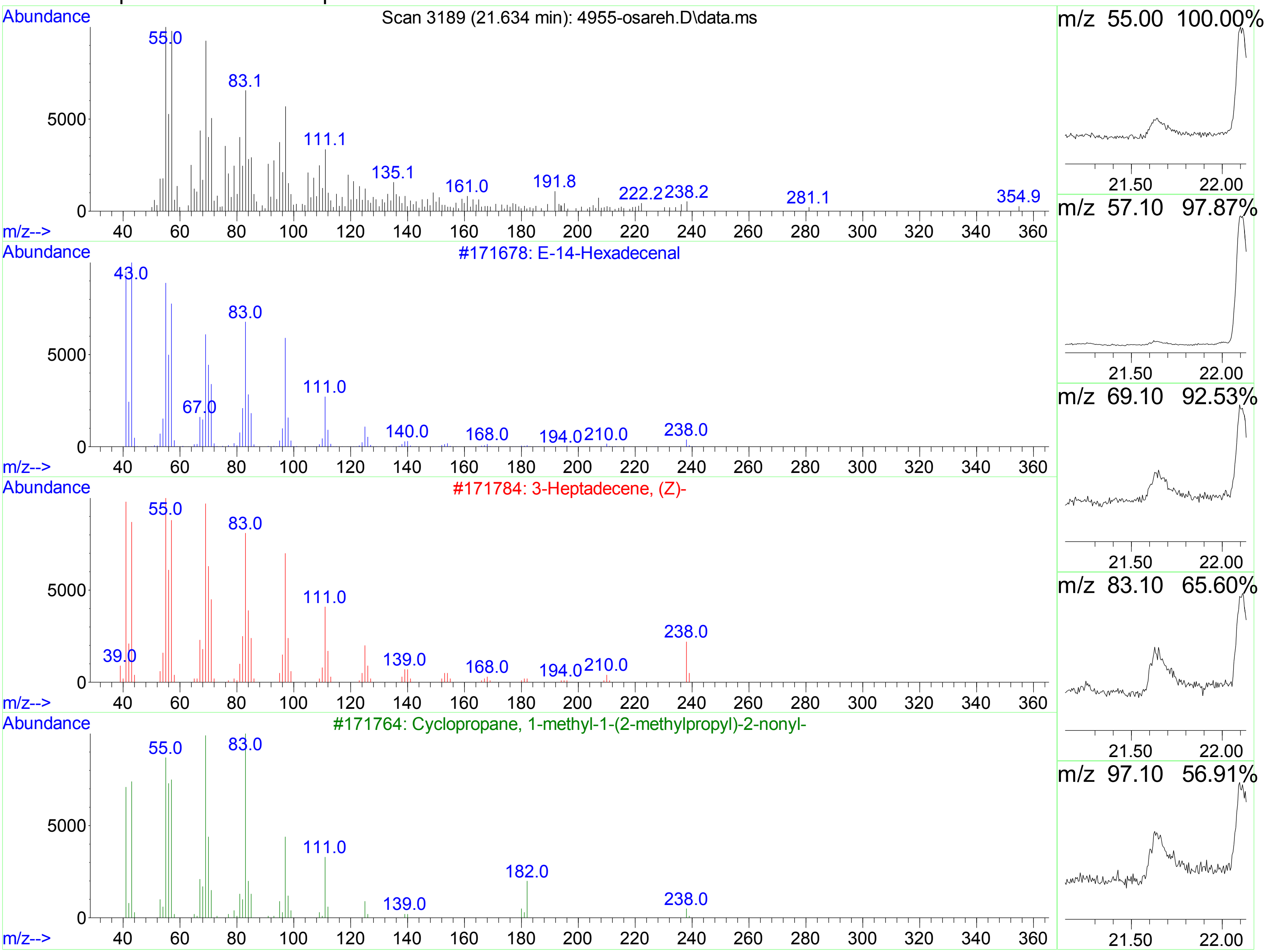

Data File: G:\ÌÒÇÑÔ 1399\99-11-18\4955-osareh.D
Sample : 4955-osareh

Peak Number: 41 at 21.634 min Area: 3201747 Area % 0.02

The 3 best hits from each library.	Ref#	CAS#	Qual
E:\Database\wiley7n.l			
1 E-14-Hexadecenal	171678	000000-00-0	93
2 3-Heptadecene, (Z)-	171784	000000-00-0	83
3 Cyclopropane, 1-methyl-1-(2-meth...	171764	041977-41-7	78

Unknown Spectrum based on Apex

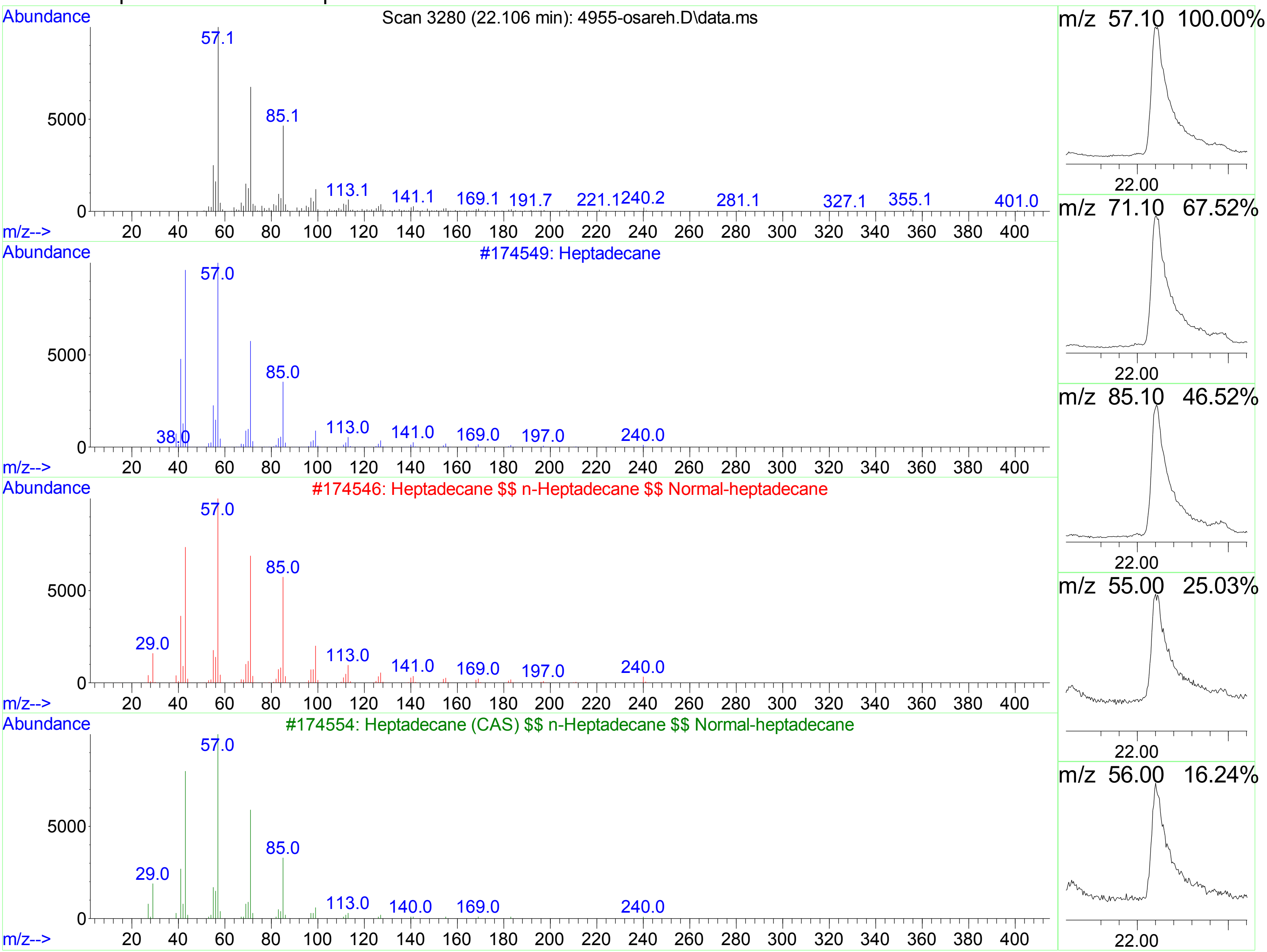

Data File: G:\ÌÒÇÑÔ 1399\99-11-18\4955-osareh.D
Sample : 4955-osareh

Peak Number: 42 at 22.106 min Area: 33636139 Area % 0.22

The 3 best hits from each library.	Ref#	CAS#	Qual
E:\Database\wiley7n.l			
1 Heptadecane	174549	000629-78-7	96
2 Heptadecane $$ n-Heptadecane $$ ...	174546	000629-78-7	96
3 Heptadecane (CAS) $$ n-Heptadeca...	174554	000629-78-7	96

Library Search Report - ChemStation Integrator

Unknown Spectrum based on Apex

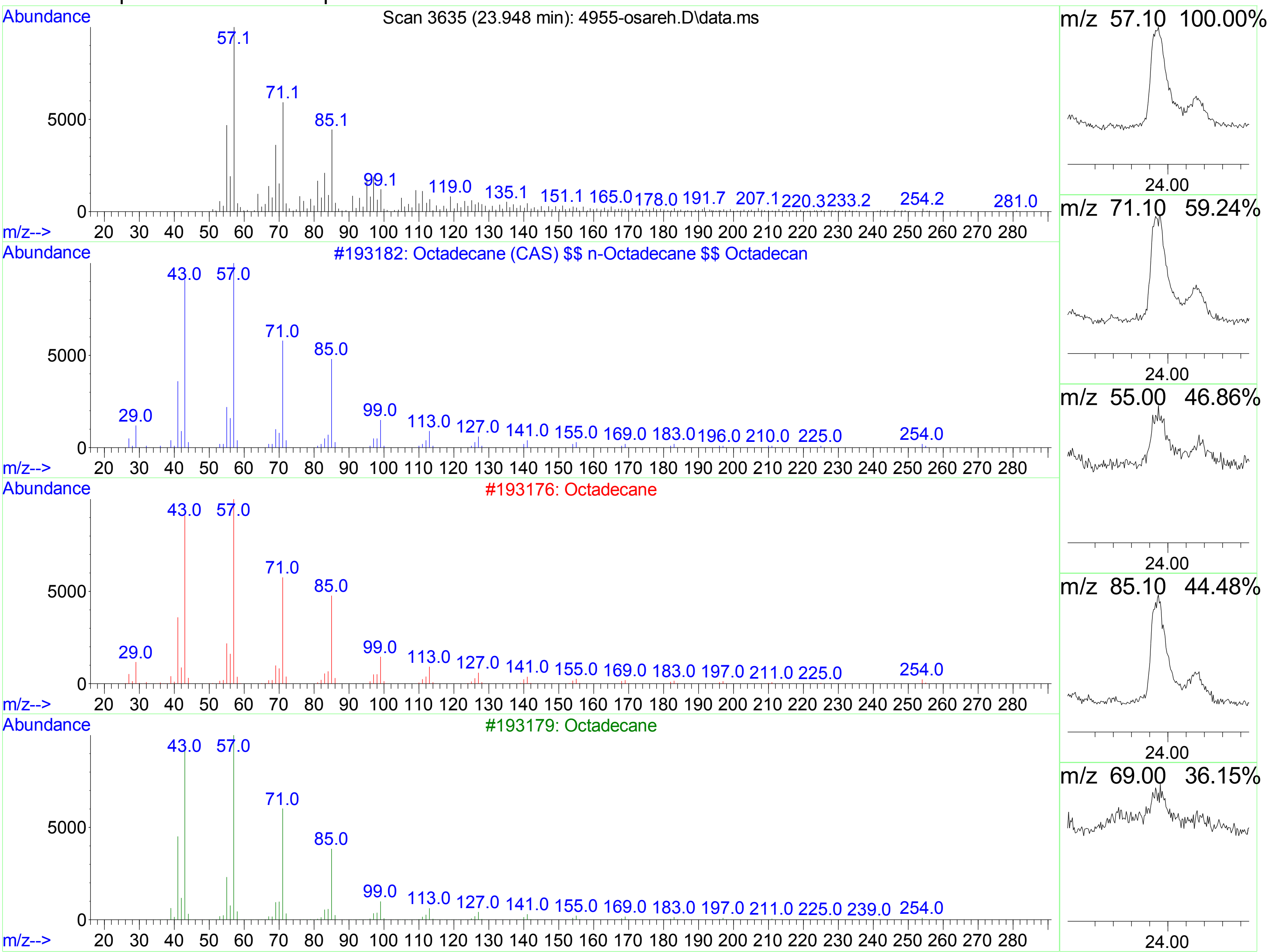

Data File: G:\ÌÒÇÑÔ 1399\99-11-18\4955-osareh.D
Sample : 4955-osareh

Peak Number: 43 at 23.948 min Area: 5961596 Area % 0.04

The 3 best hits from each library.	Ref#	CAS#	Qual
E:\Database\wiley7n.l			
1 Octadecane (CAS) $$ n-Octadecane...	193182	000593-45-3	95
2 Octadecane	193176	000593-45-3	95
3 Octadecane	193179	000593-45-3	92

Unknown Spectrum based on Apex

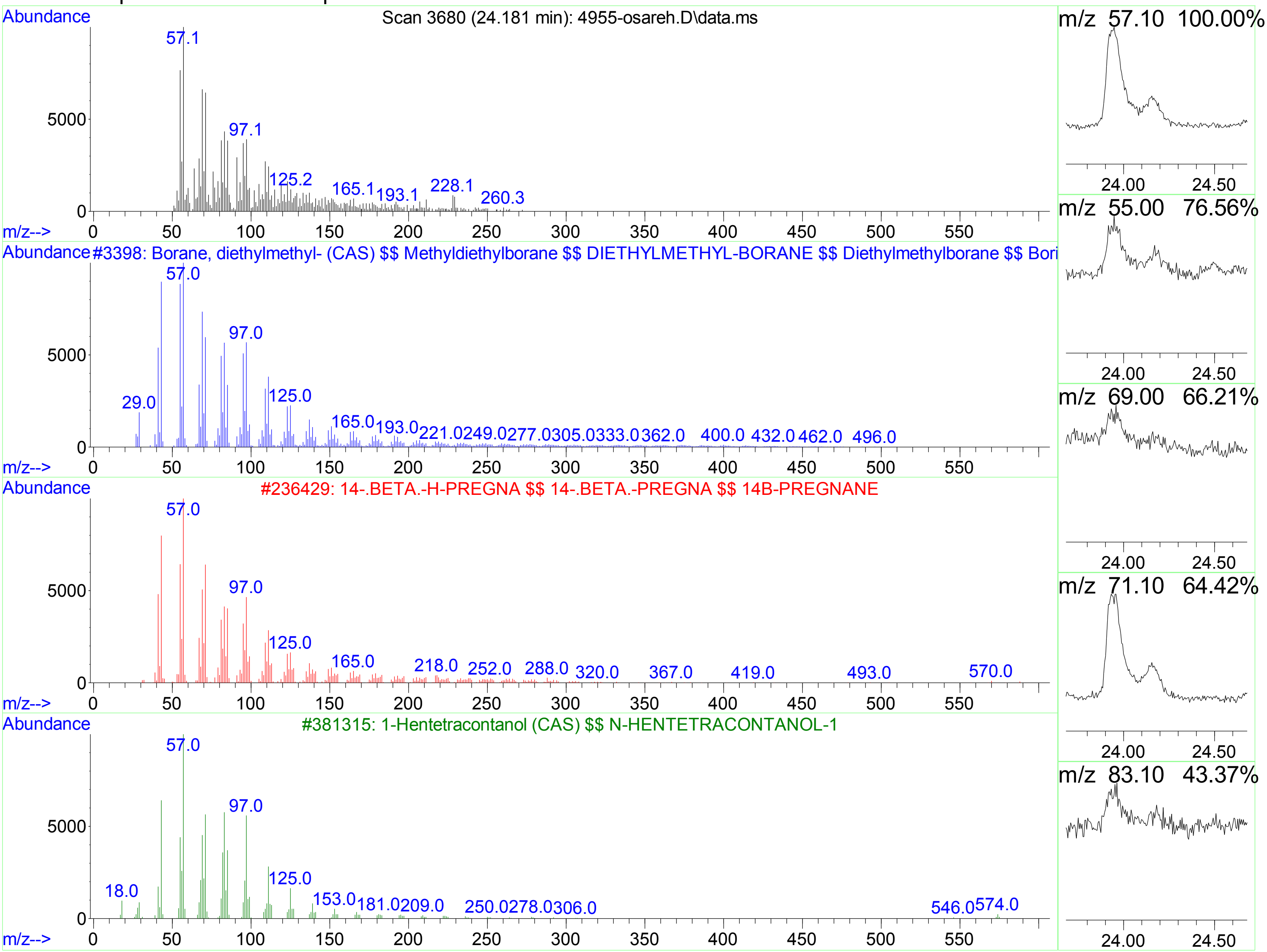

Data File: G:\ÌÒÇÑÔ 1399\99-11-18\4955-osareh.D
Sample : 4955-osareh

Peak Number: 44 at 24.181 min Area: 2070380 Area % 0.01

The 3 best hits from each library. Ref\# CAS\# Qual

E:\Database\wiley7n.l

1	Borane, diethylmethyl- (CAS) $$ ...	3398	001115-07-7	93
2	14-.BETA.-H-PREGNA $$ 14-.BETA.-...	236429	000000-00-0	81
3	1-Hentetracontanol (CAS) $$ N-HE...	381315	040710-42-7	70

Unknown Spectrum based on Apex

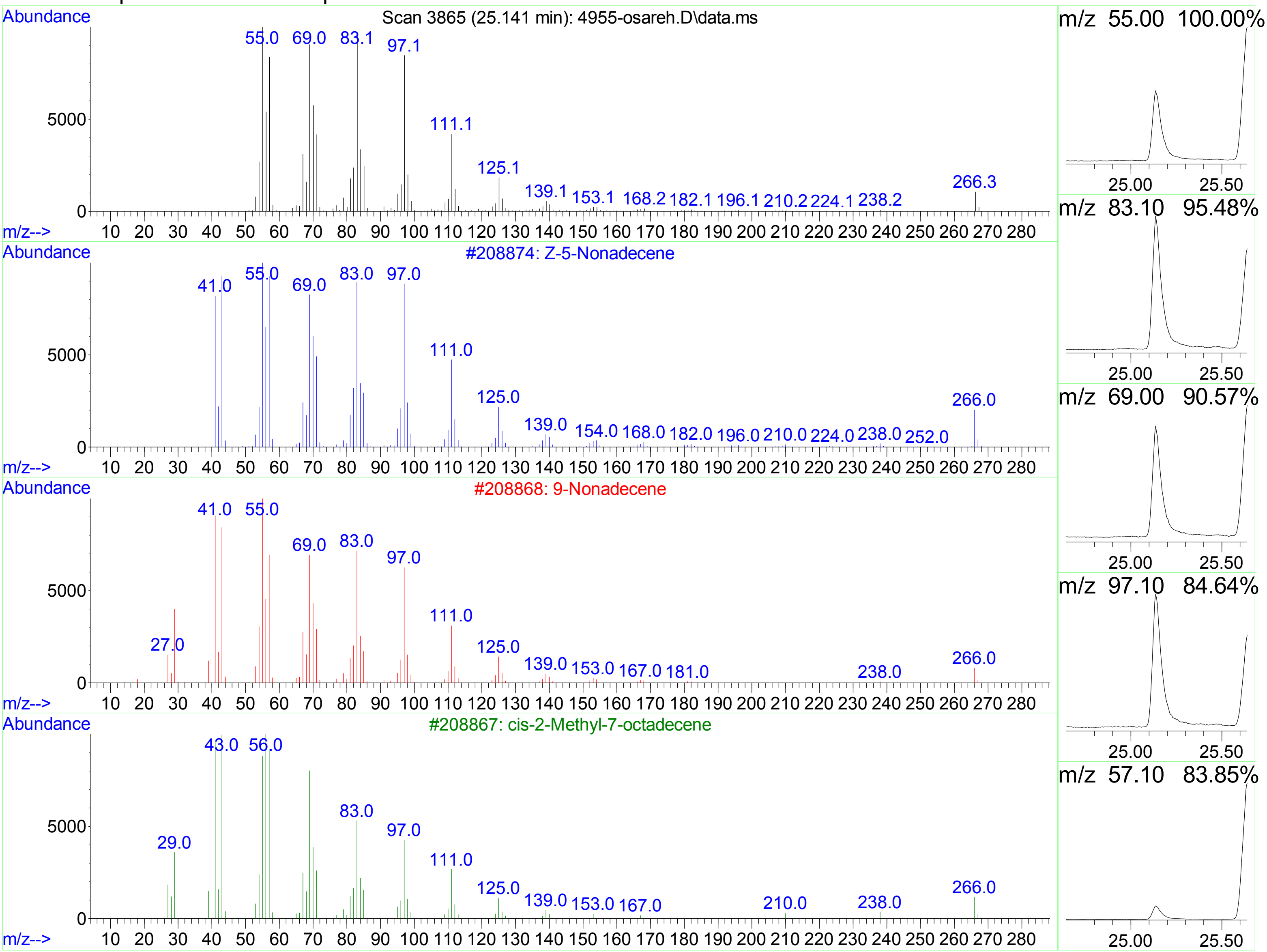

Data File: G:\ÌÒÇÑÔ 1399\99-11-18\4955-osareh.D
Sample : 4955-osareh

Peak Number: 45 at 25.141 min Area: 90517020 Area % 0.59

The 3 best hits from each library.	Ref#	CAS#	Qual
E:\Database\wiley7n.l			
1 Z-5-Nonadecene	208874	000000-00-0	99
2 9-Nonadecene	208868	031035-07-1	97
3 cis-2-Methyl-7-octadecene	208867	035354-39-3	93

Unknown Spectrum based on Apex

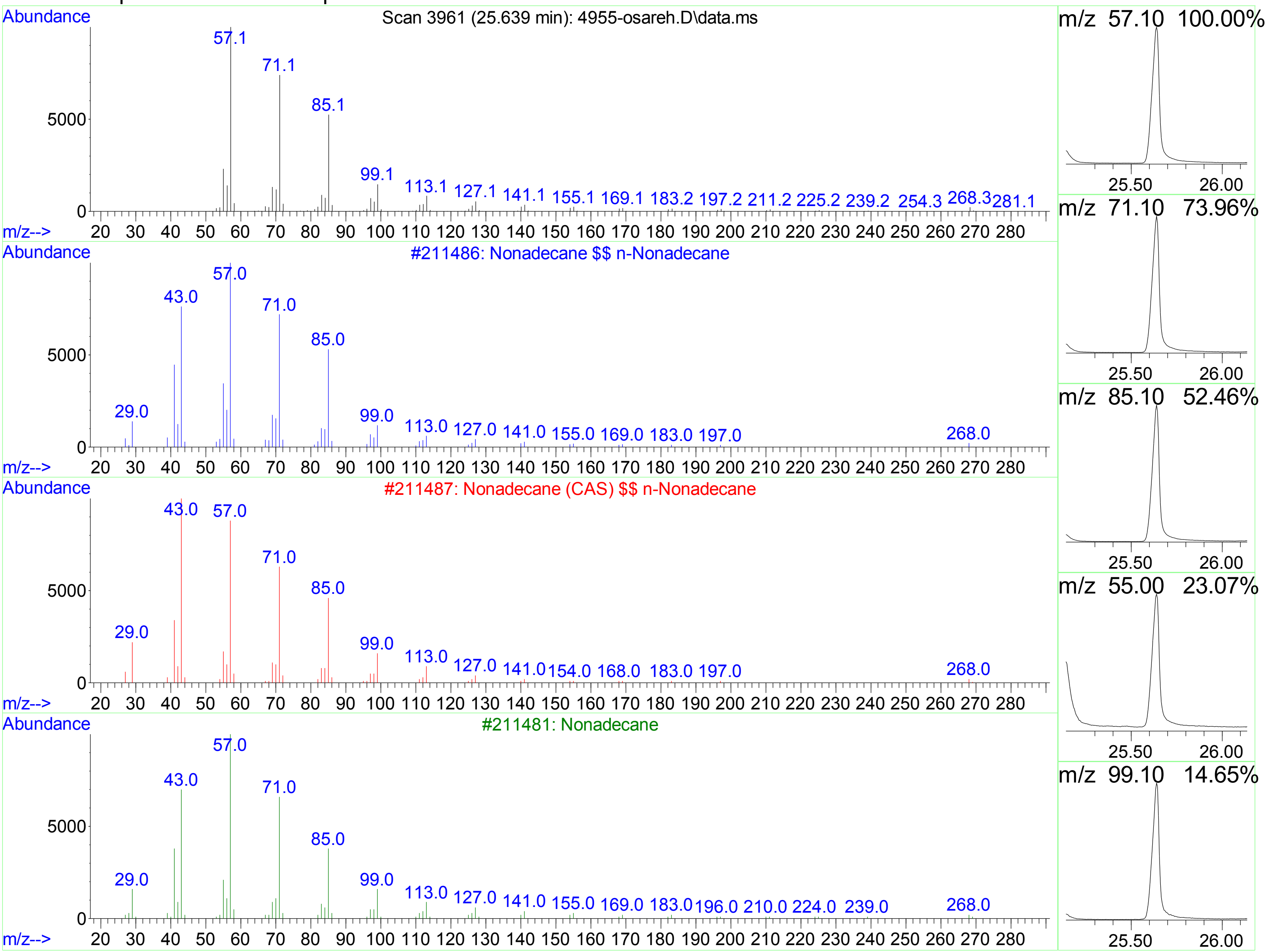

Data File: G:\ÌÒÇÑÔ 1399\99-11-18\4955-osareh.D
Sample : 4955-osareh

Peak Number: 46 at 25.639 min Area: 252670422 Area % 1.65

The 3 best hits from each library.	Ref\#	CAS\#	Qual
E:\Database\wiley7n.l			
1 Nonadecane $$ n-Nonadecane	211486	000629-92-5	98
2 Nonadecane (CAS) $$ n-Nonadecane	211487	000629-92-5	98
3 Nonadecane	211481	000629-92-5	95

Unknown Spectrum based on Apex

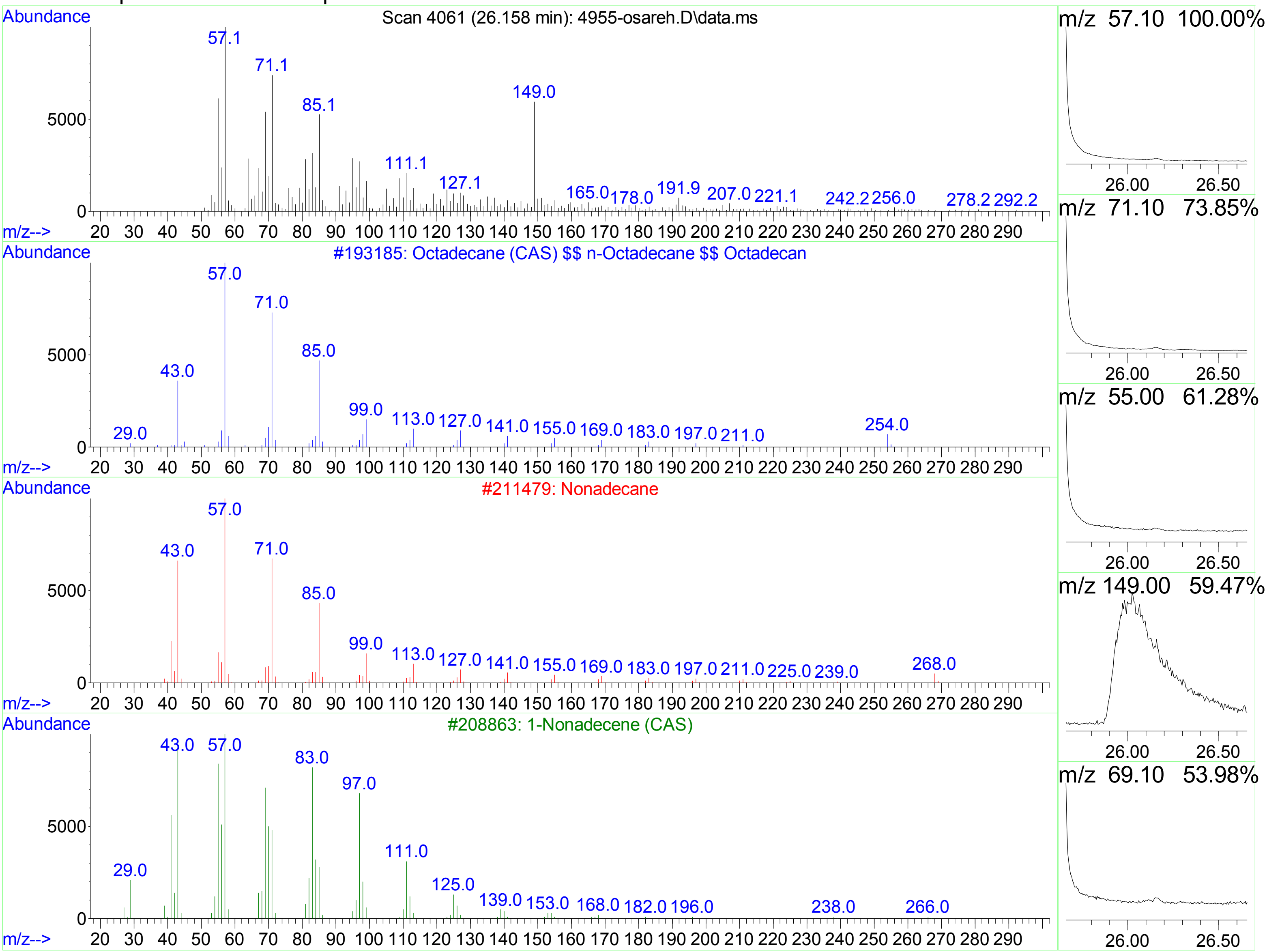

Data File: G:\ÌÒÇÑÔ 1399\99-11-18\4955-osareh.D
Sample : 4955-osareh

Peak Number: 47 at 26.158 min Area: 2150202 Area % 0.01

The 3 best hits from each library. Ref# CAS# Qual

E:\Database\wiley7n.l

	Name	Ref#	CAS#	Qual
1	Octadecane (CAS) $$ n-Octadecane...	193185	000593-45-3	90
2	Nonadecane	211479	000629-92-5	87
3	1-Nonadecene (CAS)	208863	018435-45-5	56

Library Search Report - ChemStation Integrator

Unknown Spectrum based on Apex

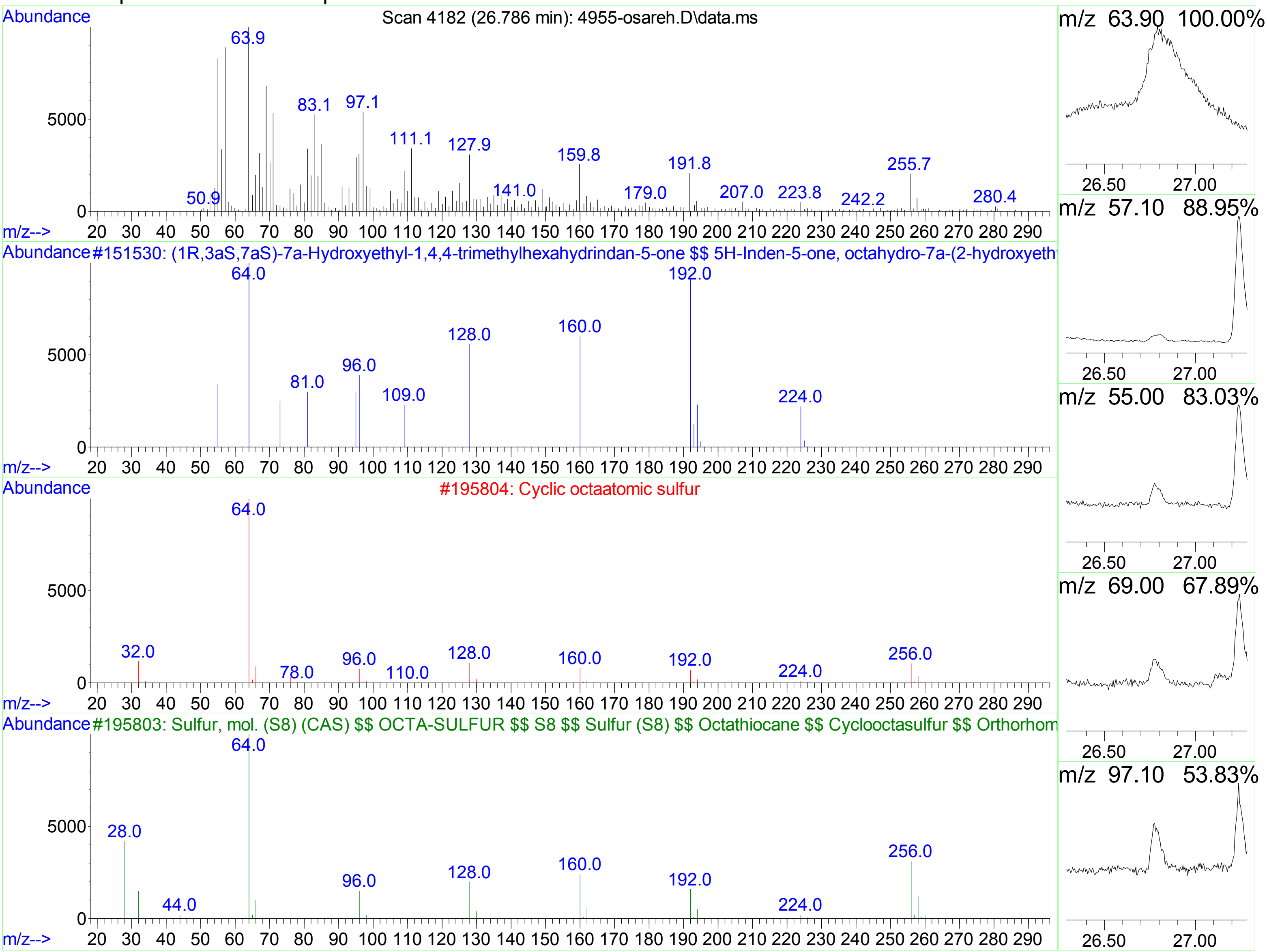

Data File: G:\ÌÒÇÑÔ 1399\99-11-18\4955-osareh.D
Sample : 4955-osareh

Peak Number: 48 at 26.786 min Area: 4765579 Area % 0.03

The 3 best hits from each library.	Ref\#	CAS\#	Qual
E:\Database\wiley7n.l			
1 (1R,3aS,7aS)-7a-Hydroxyethyl-1,4...	151530	128790-34-1	70
2 Cyclic octaatomic sulfur	195804	010544-50-0	70
3 Sulfur, mol. (S8) (CAS) $$ OCTA-...	195803	010544-50-0	70

Library Search Report - ChemStation Integrator

Unknown Spectrum based on Apex

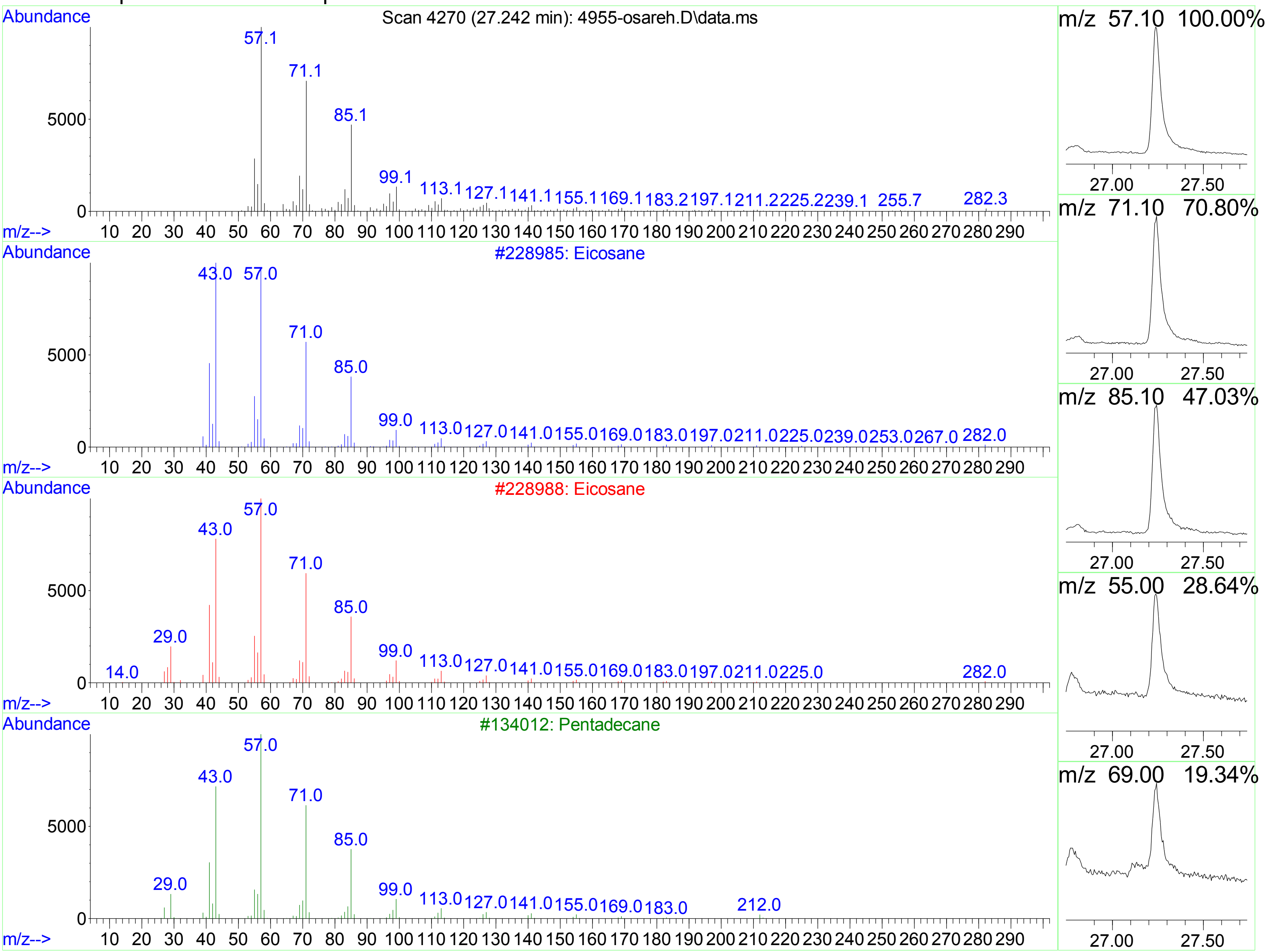

Data File: G:\ÌÒÇÑÔ 1399\99-11-18\4955-osareh.D
Sample : 4955-osareh

Peak Number: 49 at 27.242 min Area: 20570476 Area % 0.13

The 3 best hits from each library. Ref\# CAS\# Qual

E:\Database\wiley7n.l

		Ref#	CAS#	Qual
1	Eicosane	228985	000112-95-8	96
2	Eicosane	228988	000112-95-8	96
3	Pentadecane	134012	000629-62-9	95

Unknown Spectrum based on Apex

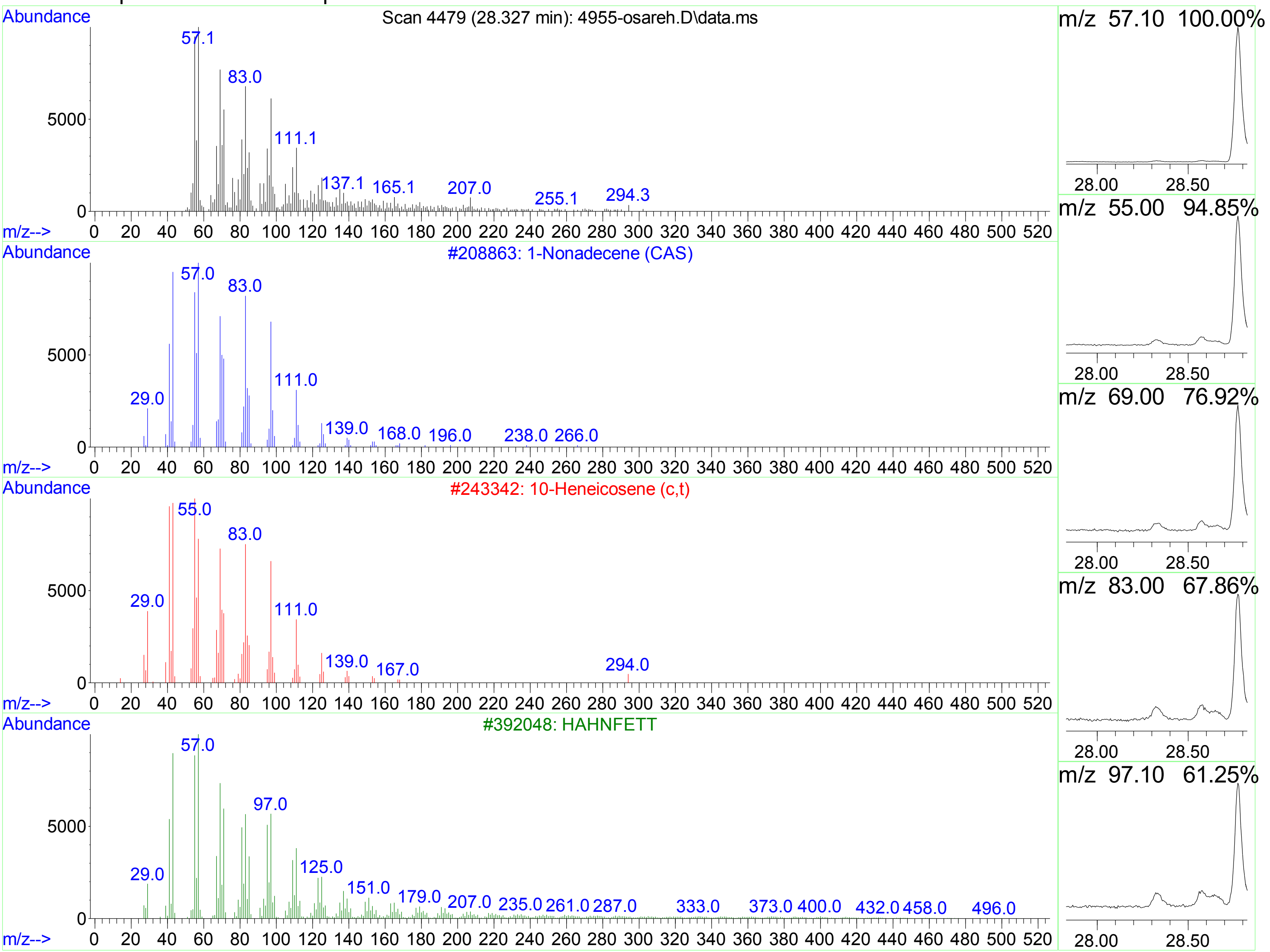

Data File: G:\ÌÒÇÑÔ 1399\99-11-18\4955-osareh.D
Sample : 4955-osareh

Peak Number: 50 at 28.327 min Area: 2932767 Area % 0.02

The 3 best hits from each library.	Ref#	CAS#	Qual
E:\Database\wiley7n.l			
1 1-Nonadecene (CAS)	208863	018435-45-5	95
2 10-Heneicosene (c,t)	243342	095008-11-0	87
3 HAHNFETT	392048	000000-00-0	87

Unknown Spectrum based on Apex

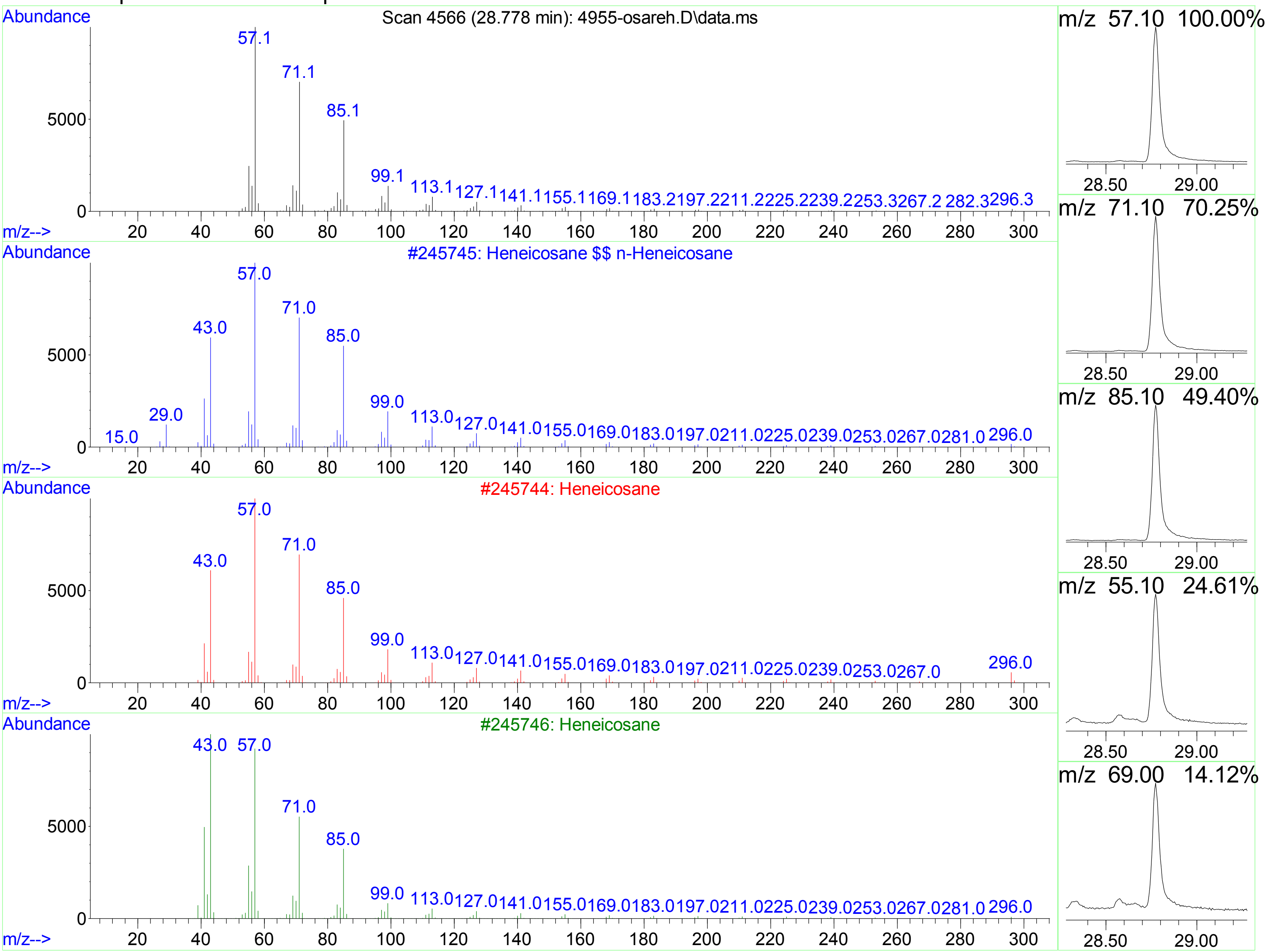

Data File: G:\ÌÒÇÑÔ 1399\99-11-18\4955-osareh.D
Sample : 4955-osareh

Peak Number: 51 at 28.778 min Area: 92159232 Area % 0.60

The 3 best hits from each library.	Ref#	CAS#	Qual
E:\Database\wiley7n.l			
1 Heneicosane $$ n-Heneicosane	245745	000629-94-7	98
2 Heneicosane	245744	000629-94-7	95
3 Heneicosane	245746	000629-94-7	94

Unknown Spectrum based on Apex

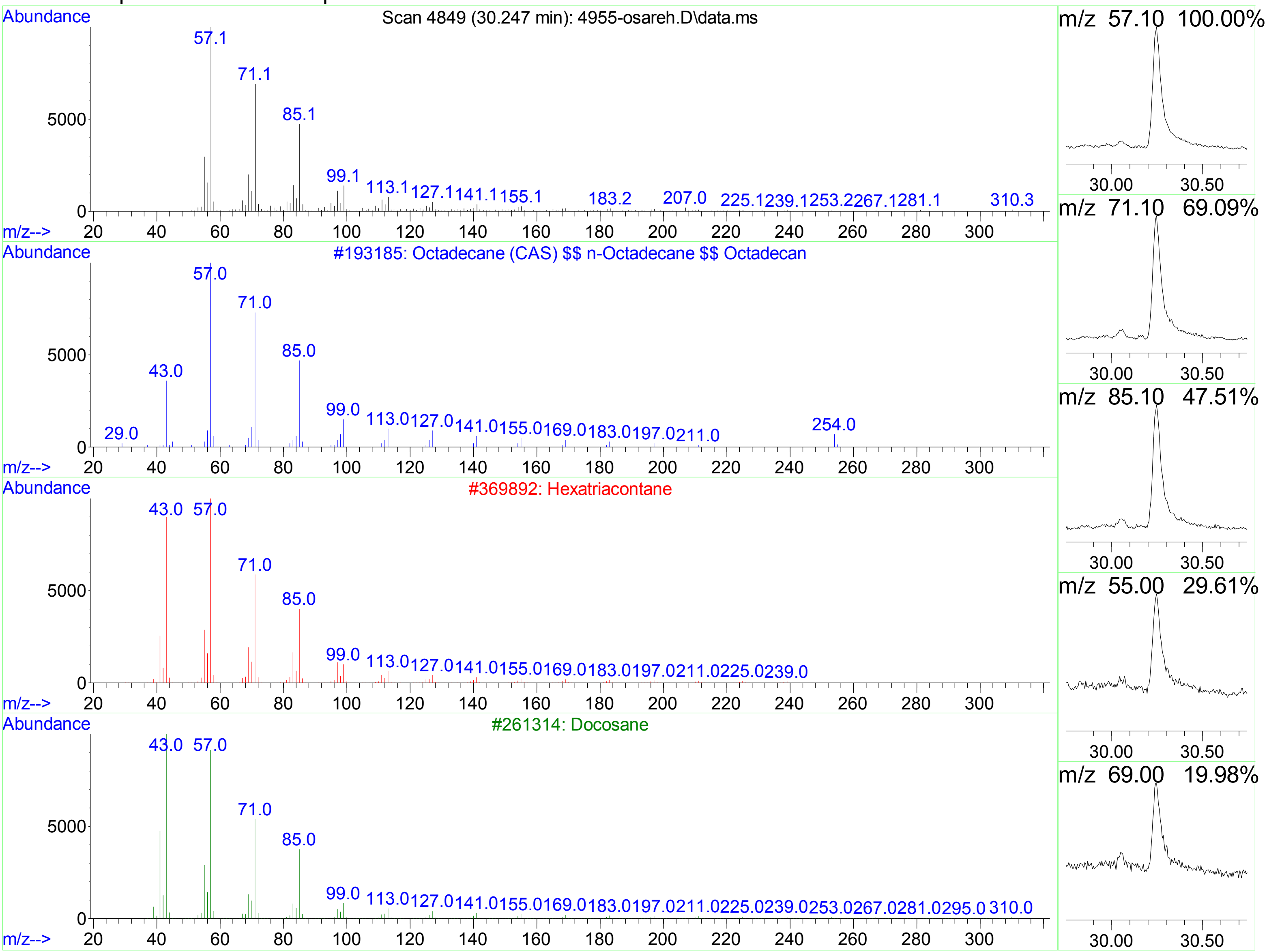

Data File: G:\ÌÒÇÑÔ 1399\99-11-18\4955-osareh.D
Sample : 4955-osareh

Peak Number: 52 at 30.247 min Area: 12036662 Area % 0.08

The 3 best hits from each library. Ref\# CAS\# Qual

E:\Database\wiley7n.l

	Name	Ref#	CAS#	Qual
1	Octadecane (CAS) $$ n-Octadecane...	193185	000593-45-3	95
2	Hexatriacontane	369892	000630-06-8	94
3	Docosane	261314	000629-97-0	92

Unknown Spectrum based on Apex

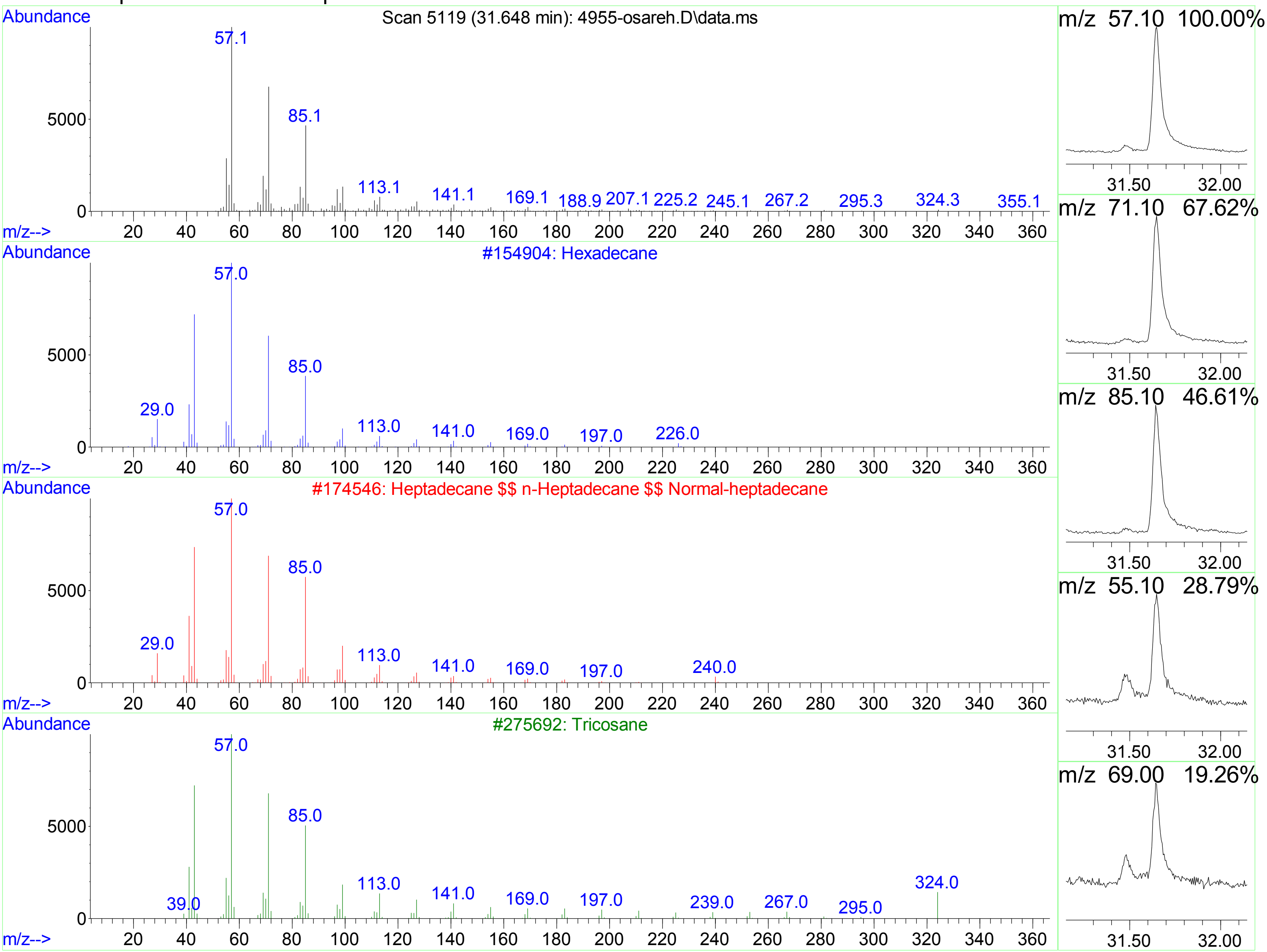

Data File: G:\ÌÒÇÑÔ 1399\99-11-18\4955-osareh.D
Sample : 4955-osareh

Peak Number: 53 at 31.648 min Area: 13595897 Area % 0.09

The 3 best hits from each library.	Ref#	CAS#	Qual
E:\Database\wiley7n.l			
1 Hexadecane	154904	000544-76-3	96
2 Heptadecane $$ n-Heptadecane $$ ...	174546	000629-78-7	93
3 Tricosane	275692	000638-67-5	93

Unknown Spectrum based on Apex

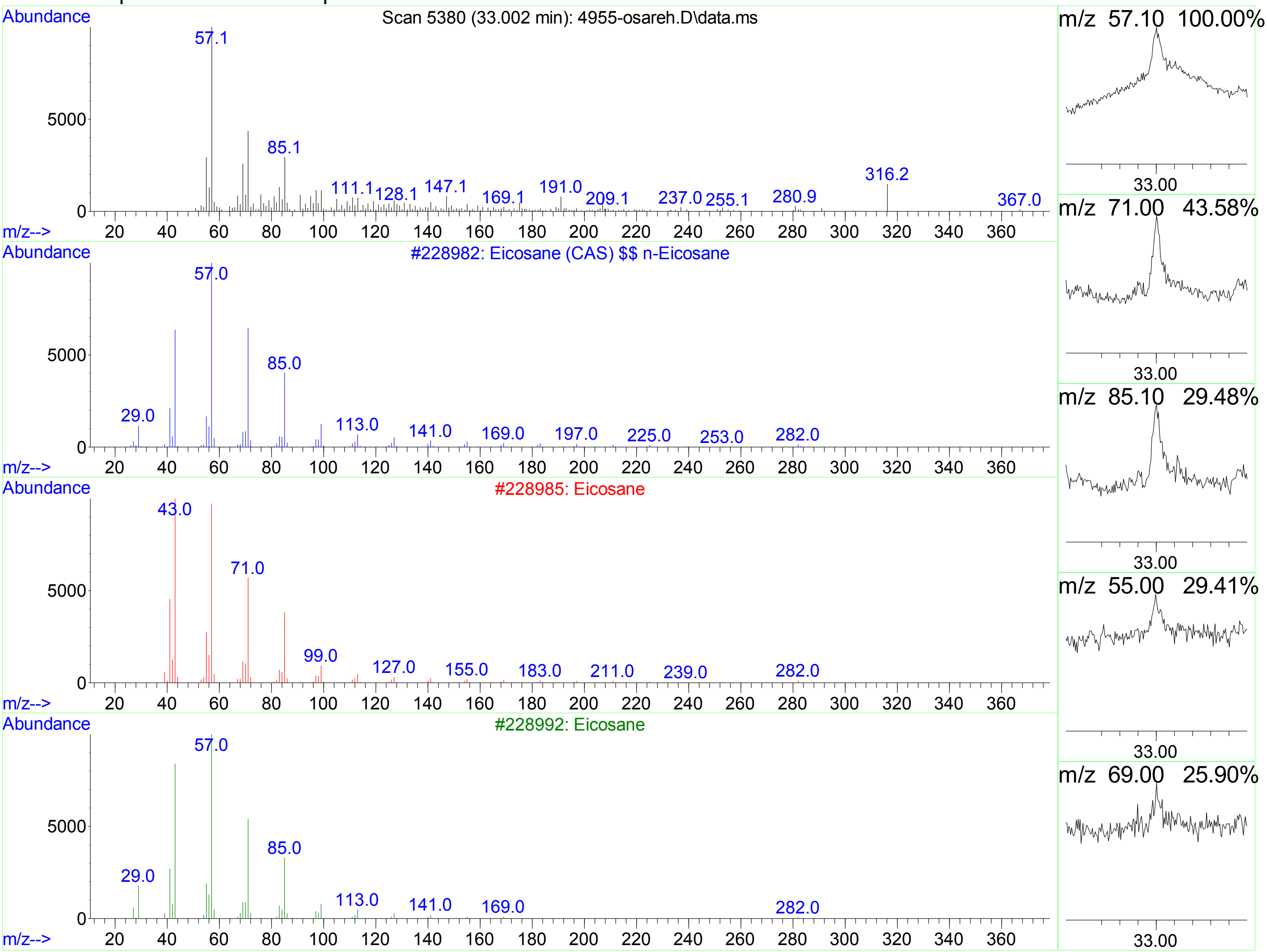

Data File: G:\ÌÒÇÑÔ 1399\99-11-18\4955-osareh.D
Sample : 4955-osareh

Peak Number: 54 at 33.002 min Area: 8036804 Area % 0.05

The 3 best hits from each library.	Ref#	CAS#	Qual
E:\Database\wiley7n.l			
1 Eicosane (CAS) $$ n-Eicosane	228982	000112-95-8	98
2 Eicosane	228985	000112-95-8	94
3 Eicosane	228992	000112-95-8	94

Unknown Spectrum based on Apex

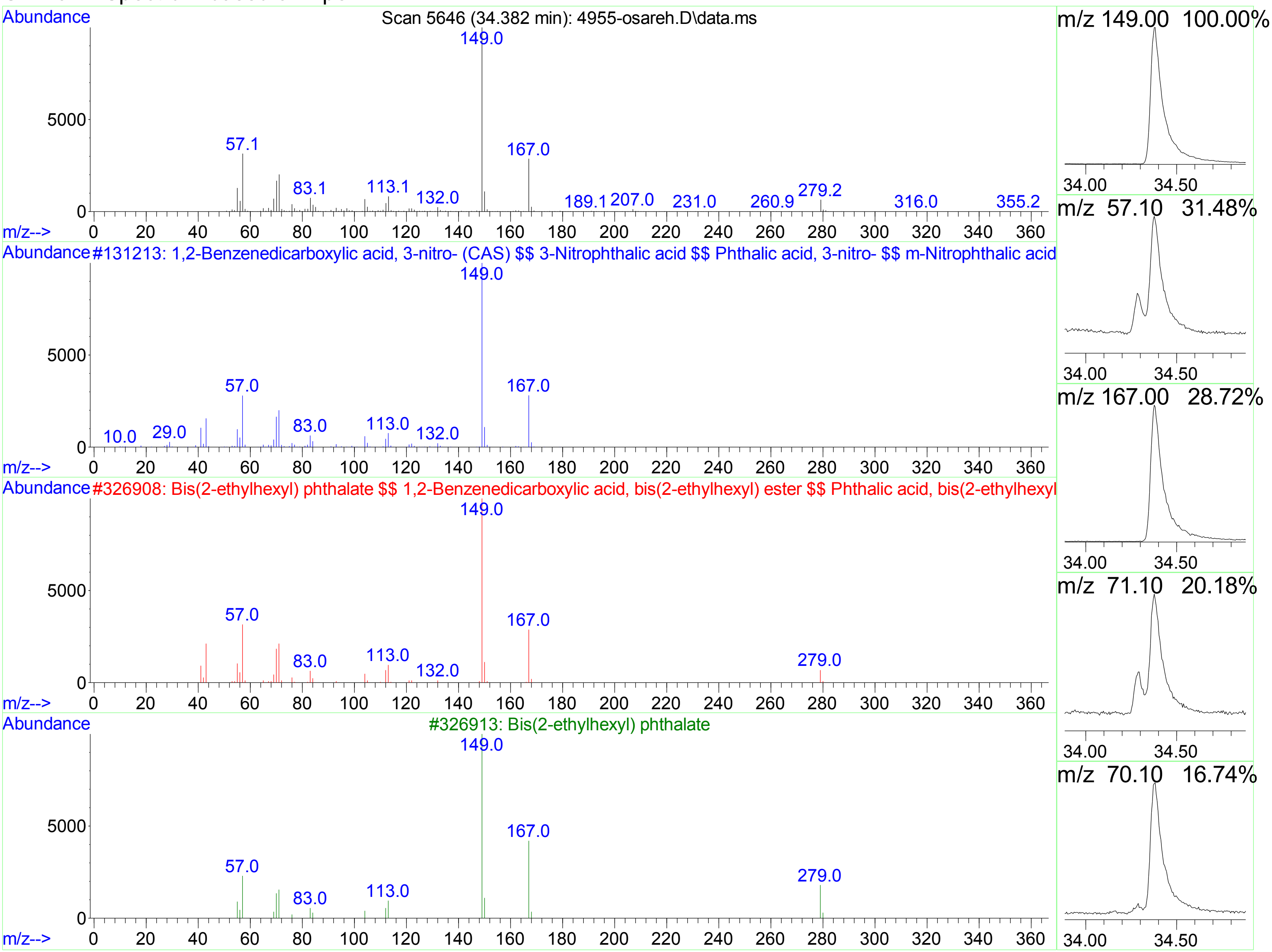

Data File: G:\ÌÒÇÑÔ 1399\99-11-18\4955-osareh.D
Sample : 4955-osareh

Peak Number: 55 at 34.382 min Area: 30104178 Area % 0.20

The 3 best hits from each library. Ref\# CAS\# Qual

E:\Database\wiley7n.l

		Ref#	CAS#	Qual
1	1,2-Benzenedicarboxylic acid, 3-...	131213	000603-11-2	91
2	Bis(2-ethylhexyl) phthalate $$ 1...	326908	000117-81-7	91
3	Bis(2-ethylhexyl) phthalate	326913	000117-81-7	91

Unknown Spectrum based on Apex

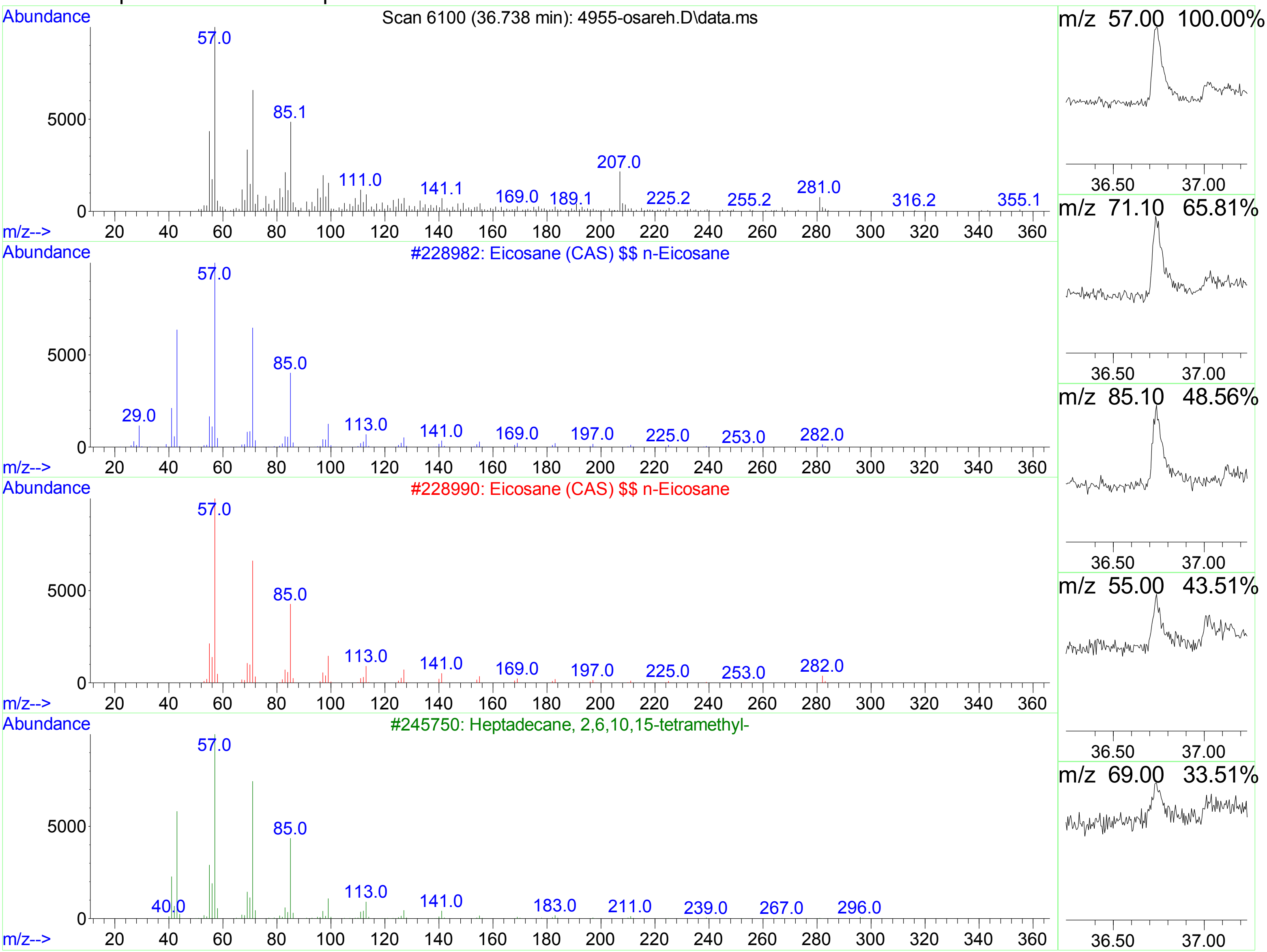

Data File: G:\ÌÒÇÑÔ 1399\99-11-18\4955-osareh.D
Sample : 4955-osareh

Peak Number: 56 at 36.738 min Area: 2878625 Area % 0.02

The 3 best hits from each library. Ref\# CAS\# Qual

E:\Database\wiley7n.l

1 Eicosane (CAS) $$ n-Eicosane	228982	000112-95-8	95
2 Eicosane (CAS) $$ n-Eicosane	228990	000112-95-8	95
3 Heptadecane, 2,6,10,15-tetramethyl-	245750	054833-48-6	91

Library Search Report - ChemStation Integrator

Unknown Spectrum based on Apex

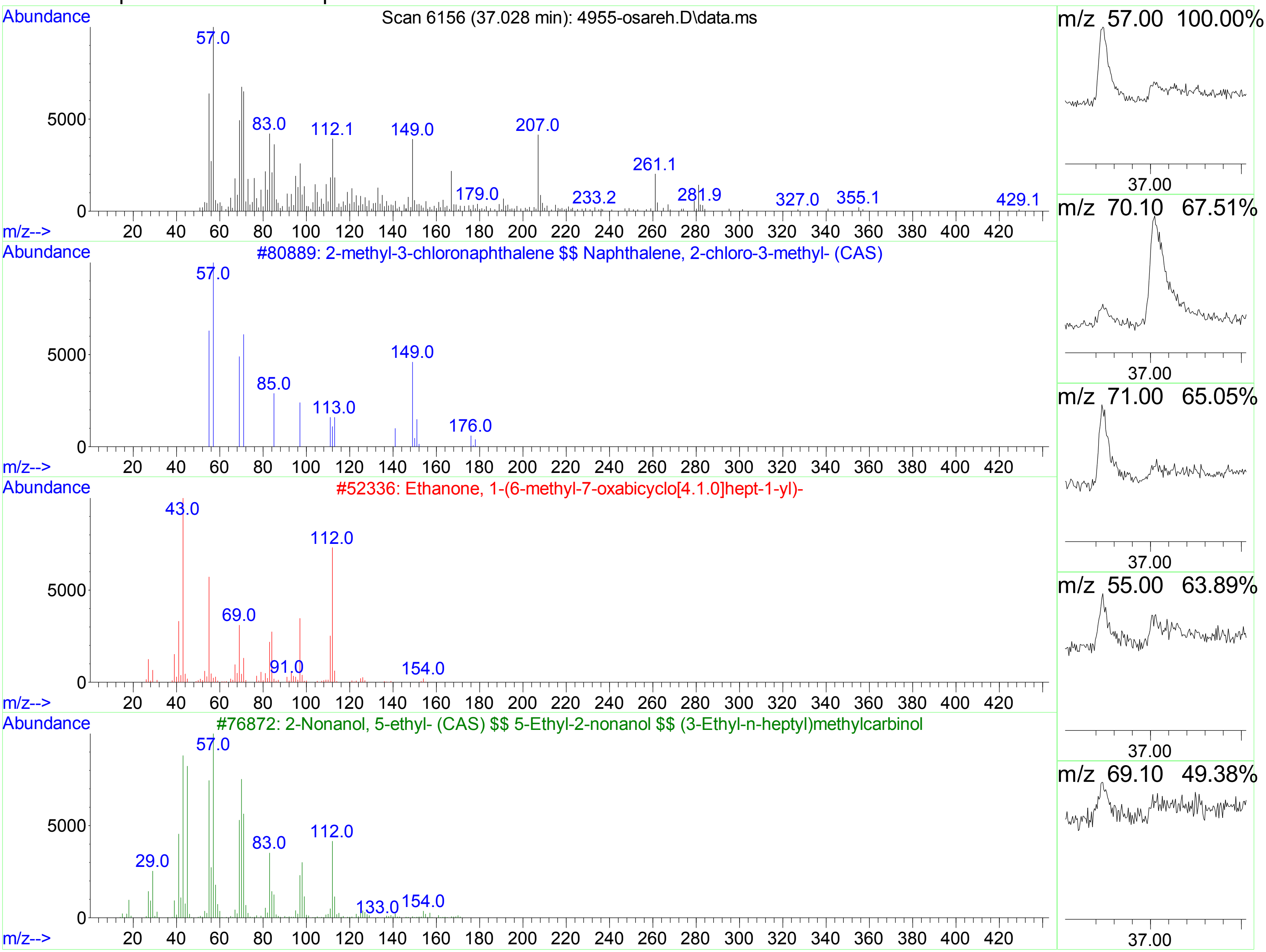

Data File: G:\ÌÒÇÑÔ 1399\99-11-18\4955-osareh.D
Sample : 4955-osareh

Peak Number: 57 at 37.028 min Area: 3444195 Area % 0.02

The 3 best hits from each library.	Ref#	CAS#	Qual
E:\Database\wiley7n.l			
1 2-methyl-3-chloronaphthalene $$ ...	80889	062956-38-1	22
2 Ethanone, 1-(6-methyl-7-oxabicyc...	52336	015120-94-2	18
3 2-Nonanol, 5-ethyl- (CAS) $$ 5-E...	76872	000103-08-2	14

Library Search Report - ChemStation Integrator

Unknown Spectrum based on Apex

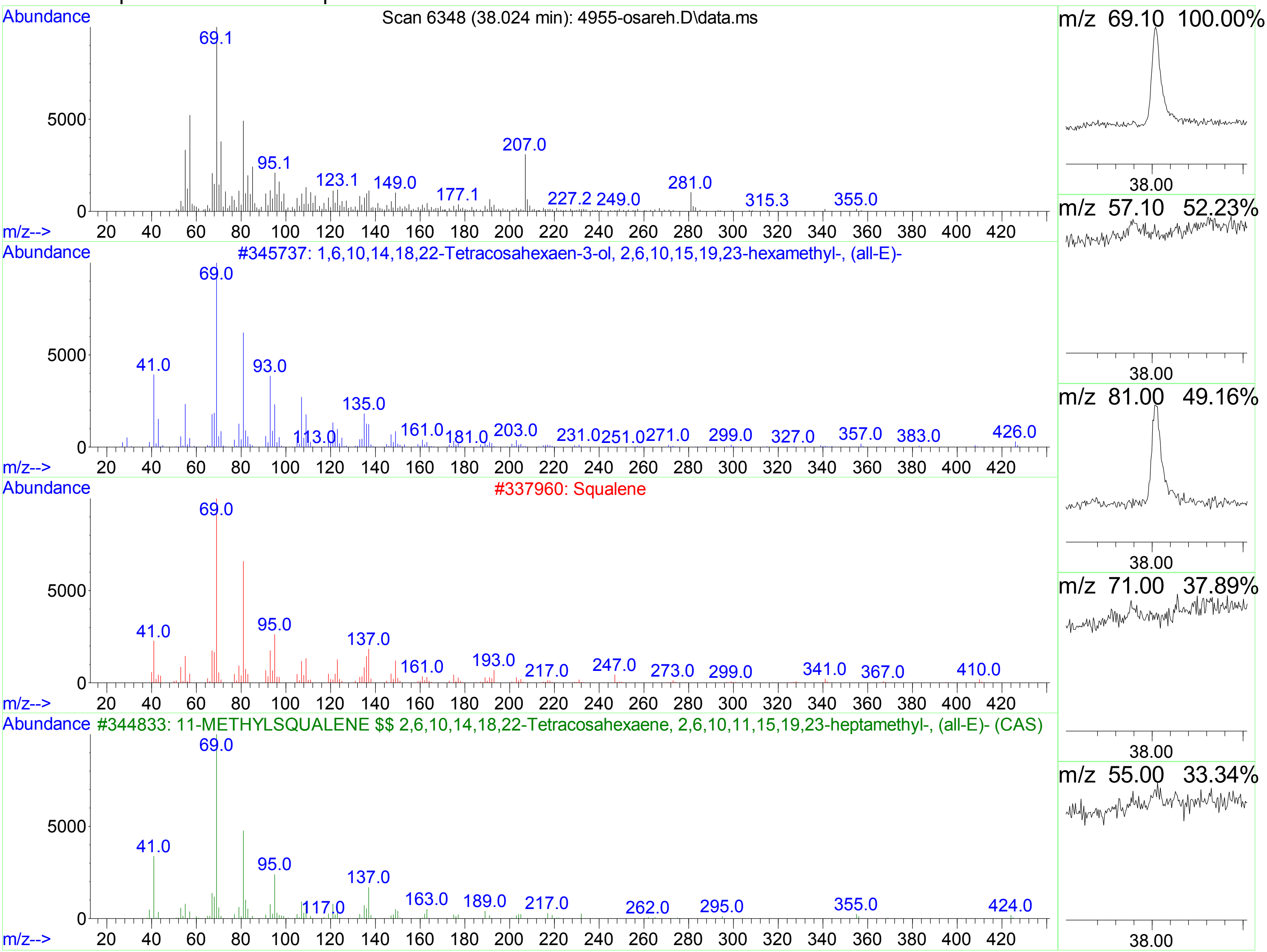

Data File: G:\ÌÒÇÑÔ 1399\99-11-18\4955-osareh.D
Sample : 4955-osareh

Peak Number: 58 at 38.024 min Area: 2350031 Area % 0.02

The 3 best hits from each library. Ref\# CAS\# Qual

E:\Database\wiley7n.l

1 1,6,10,14,18,22-Tetracosahexaen-... 345737 054159-46-5 62
2 Squalene 337960 007683-64-9 62
3 11-METHYLSQUALENE $$ 2,6,10,14,1... 344833 063424-36-2 50

Unknown Spectrum based on Apex

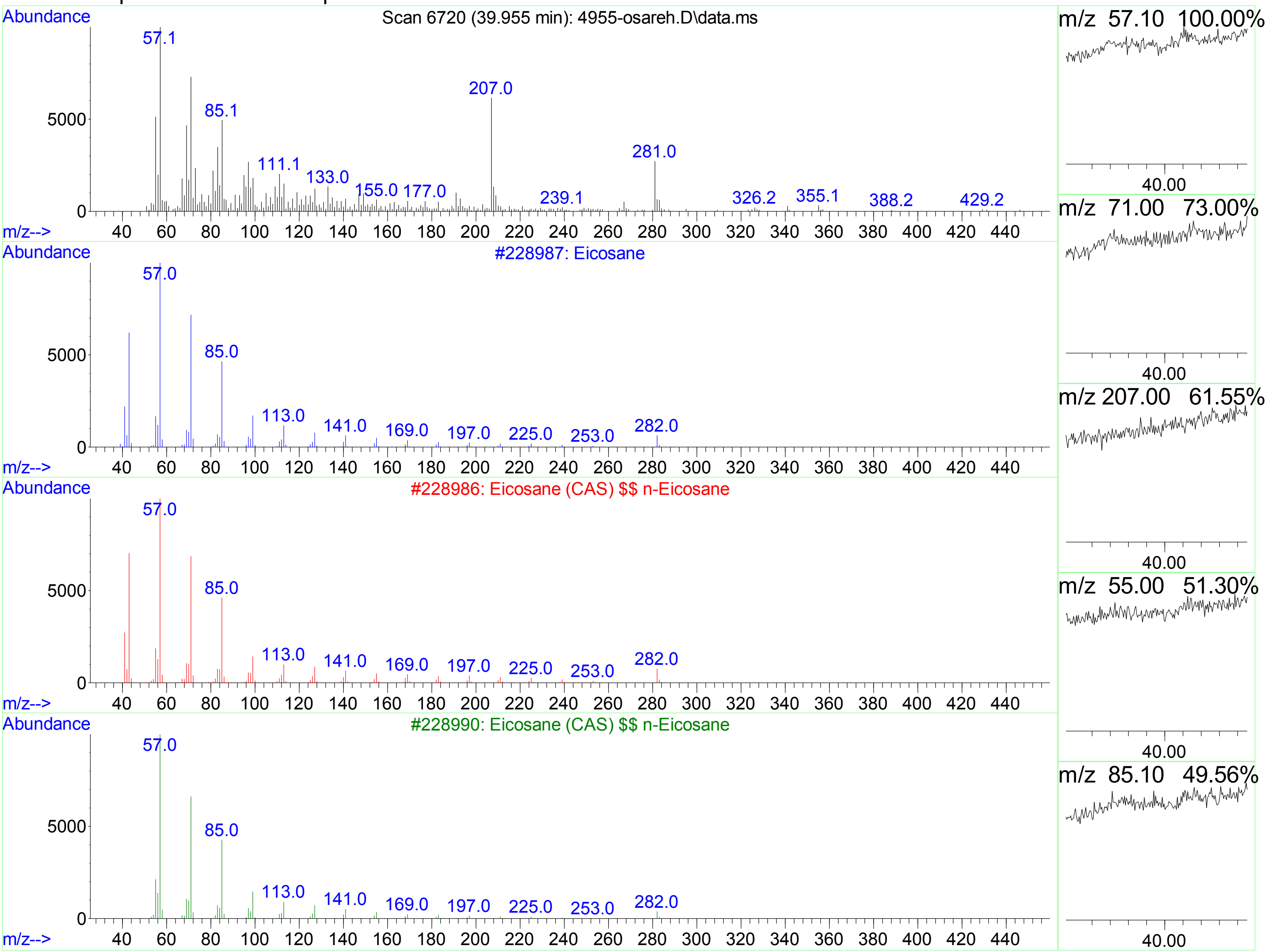

Data File: G:\ÌÒÇÑÔ 1399\99-11-18\4955-osareh.D
Sample : 4955-osareh

Peak Number: 59 at 39.955 min Area: 2423136 Area % 0.02

The 3 best hits from each library.	Ref\#	CAS\#	Qual
E:\Database\wiley7n.l			
1 Eicosane	228987	000112-95-8	92
2 Eicosane (CAS) $$ n-Eicosane	228986	000112-95-8	74
3 Eicosane (CAS) $$ n-Eicosane	228990	000112-95-8	72

Library Search Report - ChemStation Integrator

Unknown Spectrum based on Apex

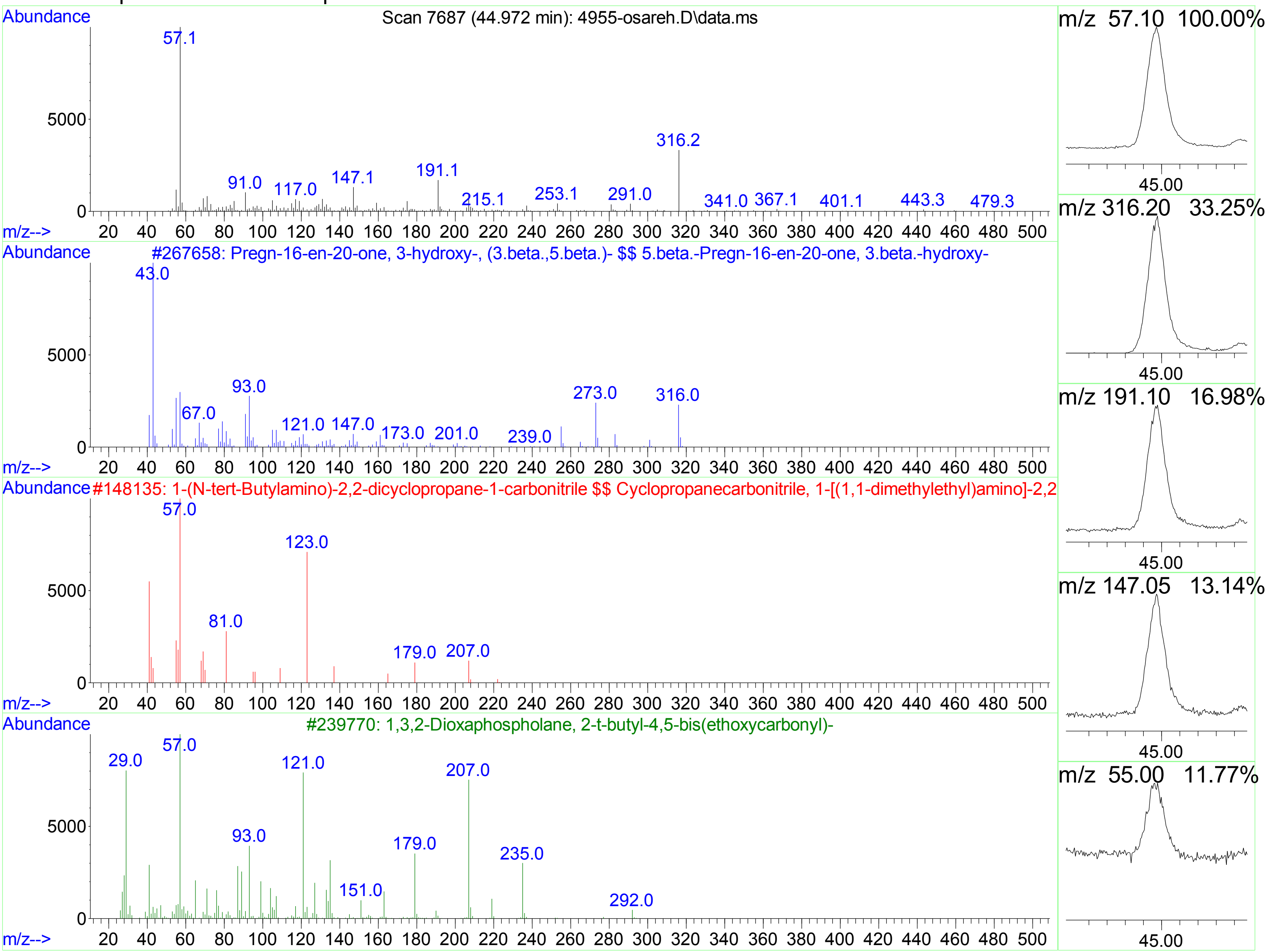

Data File: G:\ÌÒÇÑÔ 1399\99-11-18\4955-osareh.D
Sample : 4955-osareh

Peak Number: 60 at 44.972 min Area: 38207846 Area % 0.25

The 3 best hits from each library. Ref\# CAS\# Qual

E:\Database\wiley7n.l

1	Pregn-16-en-20-one, 3-hydroxy-, ...	267658	000566-59-6	10
2	1-(N-tert-Butylamino)-2,2-dicycl...	148135	123445-52-3	9
3	1,3,2-Dioxaphospholane, 2-t-buty...	239770	080043-97-6	9

Unknown Spectrum based on Apex

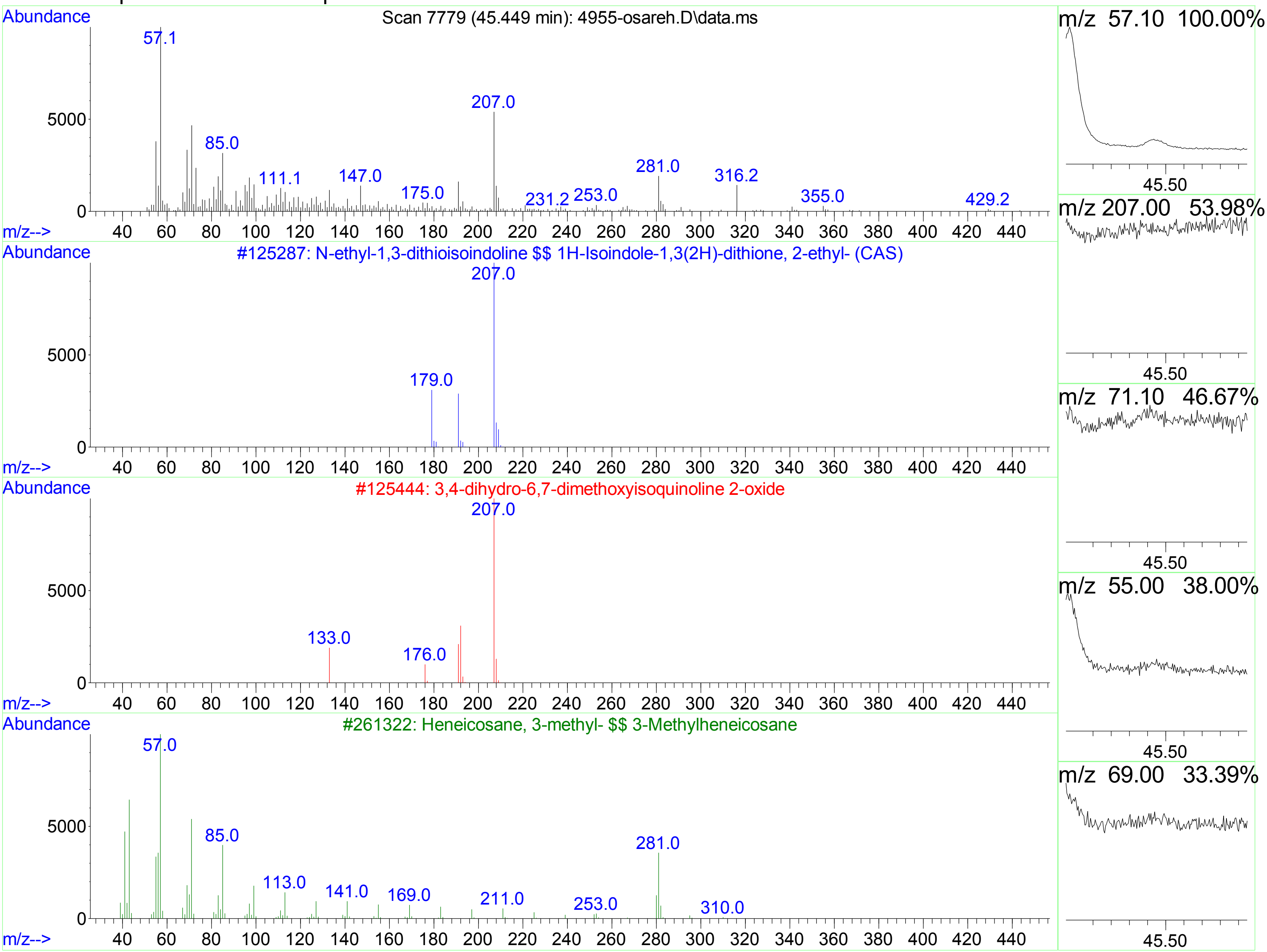

Data File: G:\ÌÒÇÑÔ 1399\99-11-18\4955-osareh.D
Sample : 4955-osareh

Peak Number: 61 at 45.449 min Area: 2621446 Area % 0.02

The 3 best hits from each library. Ref# CAS# Qual

E:\Database\wiley7n.l

	Name	Ref#	CAS#	Qual
1	N-ethyl-1,3-dithioisoindoline $$...	125287	035373-06-9	49
2	3,4-dihydro-6,7-dimethoxyisoquin...	125444	084122-10-1	47
3	Heneicosane, 3-methyl- $$ 3-Meth...	261322	006418-47-9	38

Library Search Report - ChemStation Integrator

Unknown Spectrum based on Apex

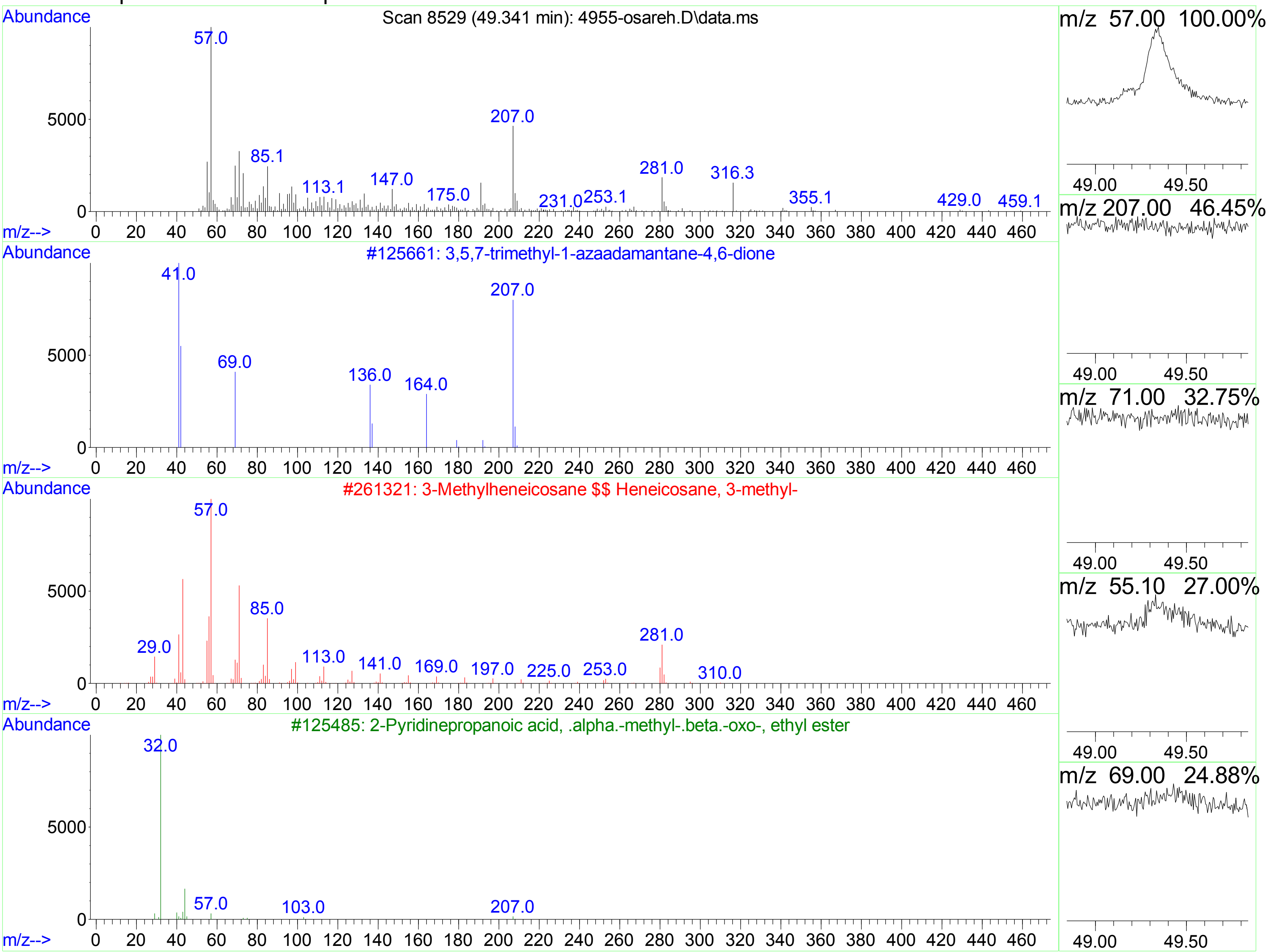

Data File: G:\ÌÒÇÑÔ 1399\99-11-18\4955-osareh.D
Sample : 4955-osareh

Peak Number: 62 at 49.341 min Area: 7120929 Area % 0.05

The 3 best hits from each library.	Ref#	CAS#	Qual
E:\Database\wiley7n.l			
1 3,5,7-trimethyl-1-azaadamantane-...	125661	136947-01-8	27
2 3-Methylheneicosane $$ Heneicosa...	261321	006418-47-9	10
3 2-Pyridinepropanoic acid, .alpha...	125485	000000-00-0	9
