# Supplementary figures and images for: Comparative de novo transcriptome analysis of flower and root of Oliveria decumbens Vent. to identify putative genes in terpenes biosynthesis pathway
Source: Front Genet. 2022 Aug 4;13:916183. doi: 10.3389/fgene.2022.916183 (PMC9386285; doi:10.3389/fgene.2022.916183)

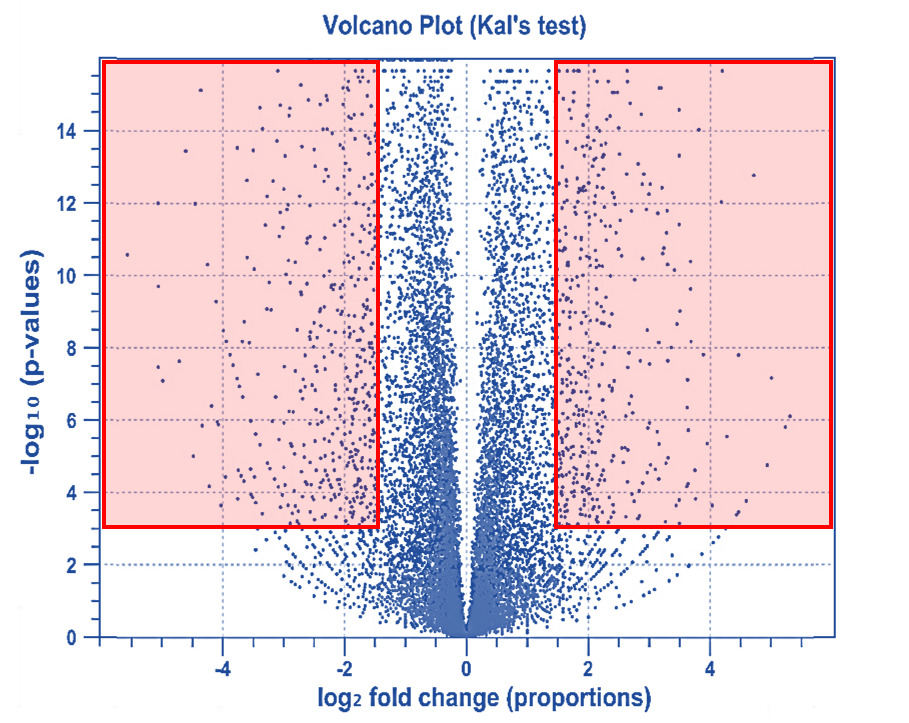

Supplement: Supplementary file 2 [file Image1.JPEG]
