# Supplementary material for: Comparative de novo transcriptome analysis of flower and root of Oliveria decumbens Vent. to identify putative genes in terpenes biosynthesis pathway
Source: Front Genet. 2022 Aug 4;13:916183. doi: 10.3389/fgene.2022.916183 (PMC9386285; doi:10.3389/fgene.2022.916183)
Supplement: Supplementary file 3 [file DataSheet1.PDF]

Library Search Report

Data Path : G:\ÌÒÇÑÔ 1399\99-09-27\
Data File : 4054-osareh.D
Acq On : 21 Dec 2020 2:51
Operator :
Sample : 4054-osareh
Misc :
ALS Vial : 89 Sample Multiplier: 1

Search Libraries: E:\Database\wiley7n.l Minimum Quality: 0

Unknown Spectrum: Apex
Integration Events: ChemStation Integrator - events.e

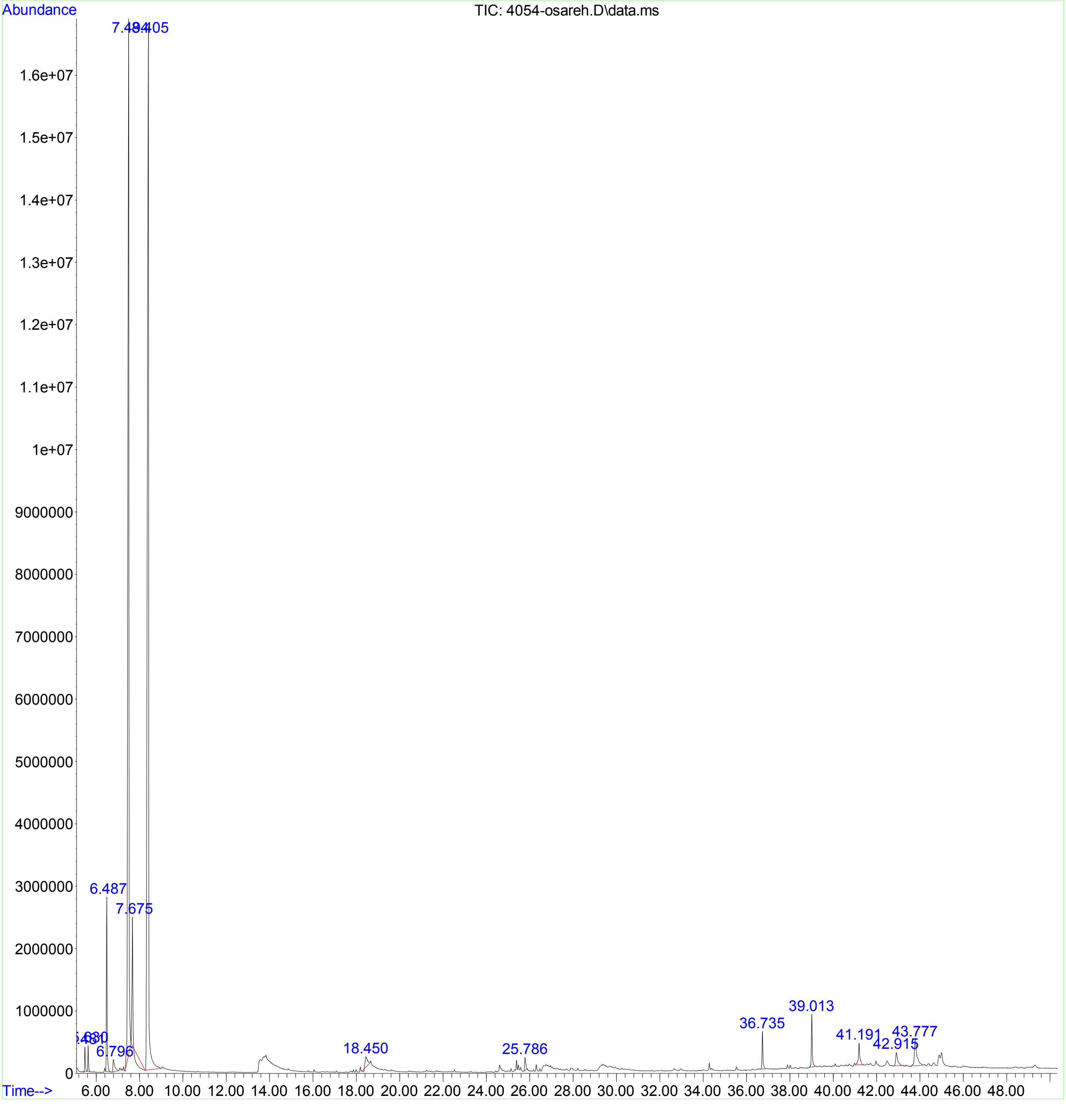

Library Search Report - ChemStation Integrator

Unknown Spectrum based on Apex

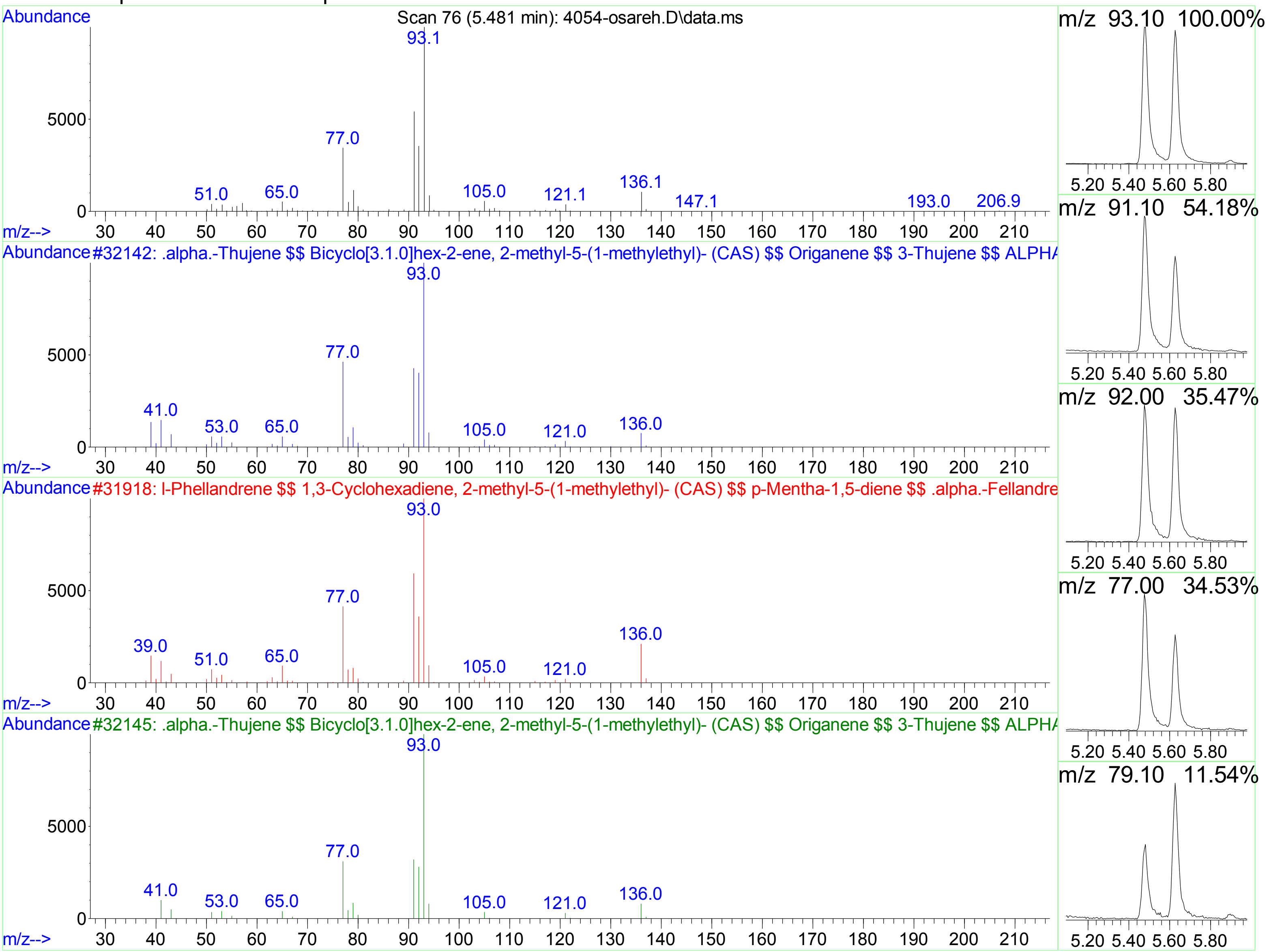

Data File: G:\ÌÒÇÑÔ 1399\99-09-27\4054-osareh.D
Sample : 4054-osareh

Peak Number: 1 at 5.481 min Area: 8088980 Area % 0.52

The 3 best hits from each library. Ref\# CAS\# Qual

E:\Database\wiley7n.l

	Name	Ref#	CAS#	Qual
1	.alpha.-Thujene $$ Bicyclo[3.1.0...	32142	002867-05-2	94
2	l-Phellandrene $$ 1,3-Cyclohexad...	31918	000099-83-2	94
3	.alpha.-Thujene $$ Bicyclo[3.1.0...	32145	002867-05-2	93

Unknown Spectrum based on Apex

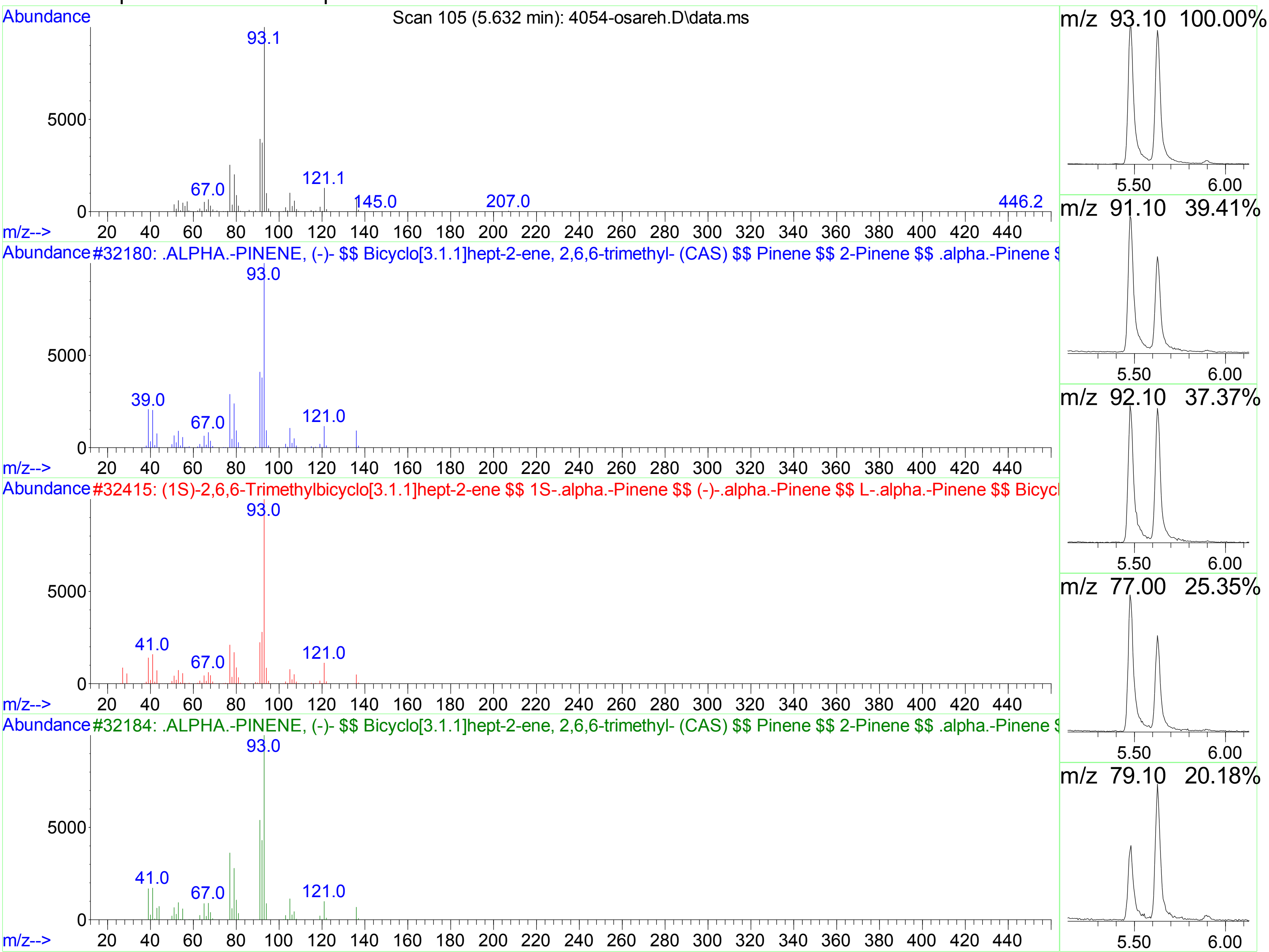

Data File: G:\ÌÒÇÑÔ 1399\99-09-27\4054-osareh.D
Sample : 4054-osareh

Peak Number: 2 at 5.632 min Area: 8636519 Area % 0.56

The 3 best hits from each library.	Ref#	CAS#	Qual
E:\Database\wiley7n.l			
1 .ALPHA.-PINENE, (-)- $$ Bicyclo[...	32180	000080-56-8	96
2 (1S)-2,6,6-Trimethylbicyclo[3.1....	32415	007785-26-4	96
3 .ALPHA.-PINENE, (-)- $$ Bicyclo[...	32184	000080-56-8	95

Library Search Report - ChemStation Integrator

Unknown Spectrum based on Apex

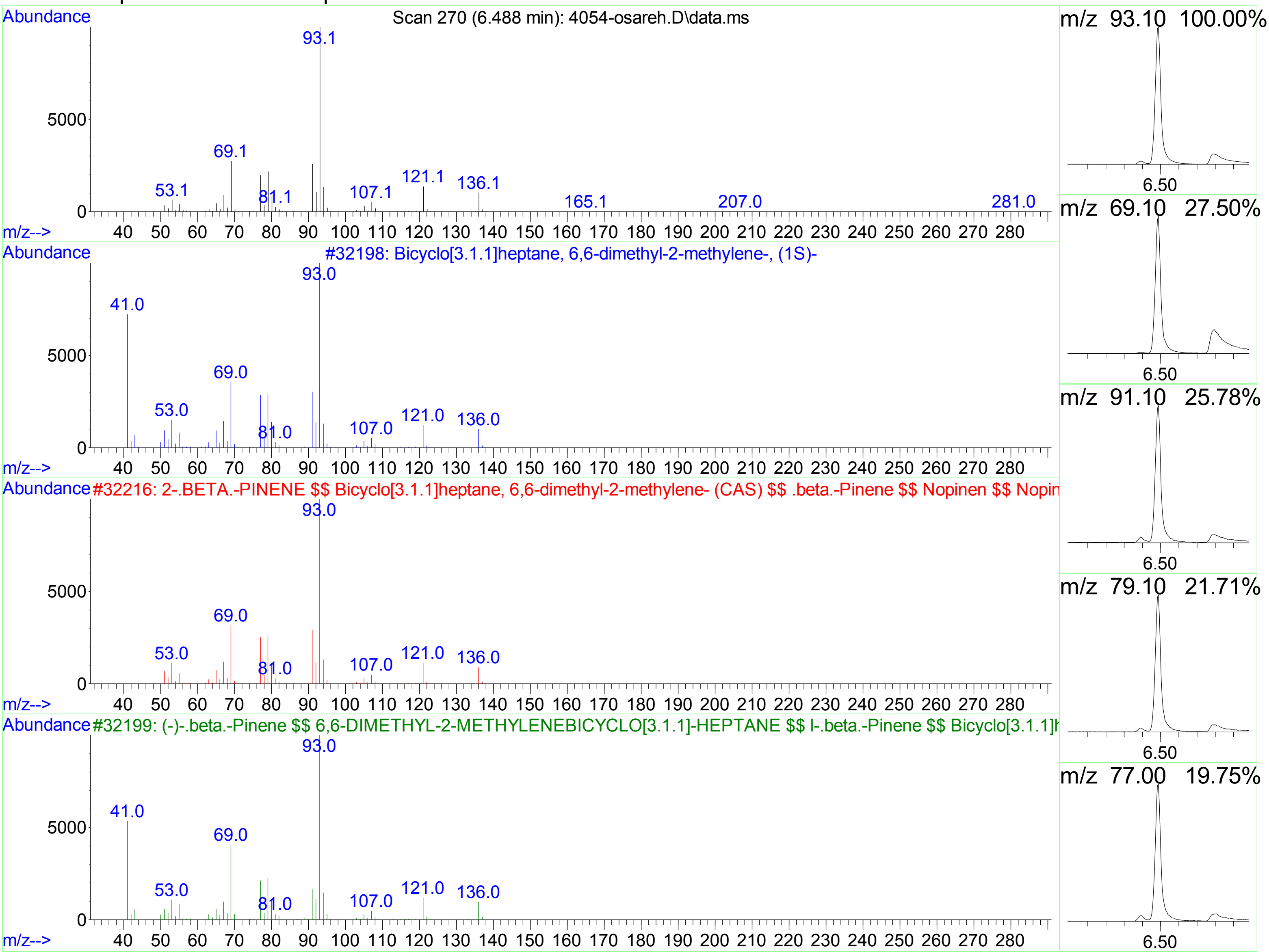

Data File: G:\ÌÒÇÑÔ 1399\99-09-27\4054-osareh.D
Sample : 4054-osareh

Peak Number: 3 at 6.488 min Area: 62613095 Area % 4.06

The 3 best hits from each library. Ref\# CAS\# Qual

E:\Database\wiley7n.l

1	Bicyclo[3.1.1]heptane, 6,6-dimet...	32198	018172-67-3	94
2	2-.BETA.-PINENE $$ Bicyclo[3.1.1...	32216	000127-91-3	94
3	(-)-.beta.-Pinene $$ 6,6-DIMETHY...	32199	018172-67-3	94

Unknown Spectrum based on Apex

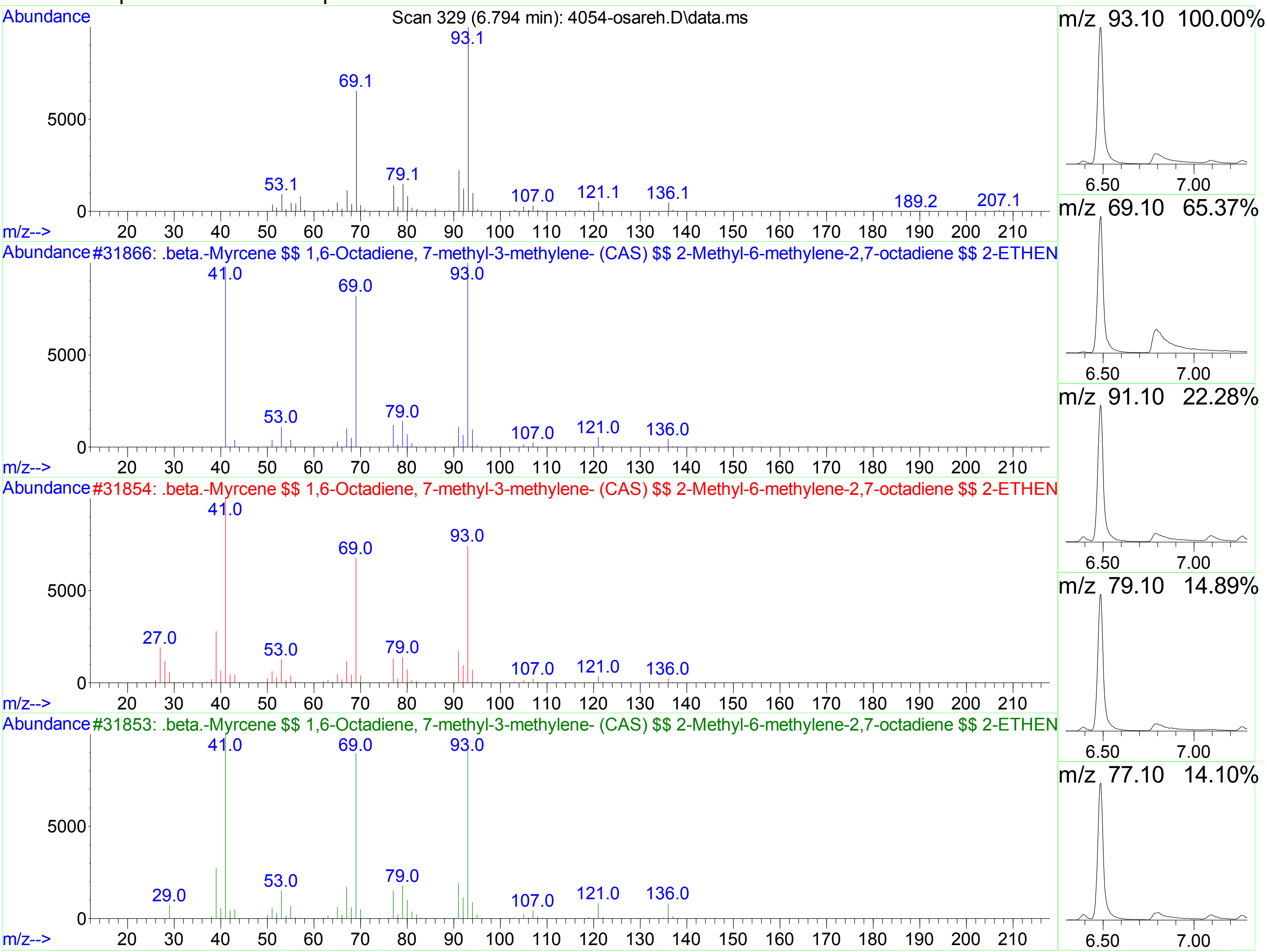

Data File: G:\ÌÒÇÑÔ 1399\99-09-27\4054-osareh.D
Sample : 4054-osareh

Peak Number: 4 at 6.794 min Area: 11427800 Area % 0.74

The 3 best hits from each library.	Ref#	CAS#	Qual
E:\Database\wiley7n.l			
1 .beta.-Myrcene $$ 1,6-Octadiene,...	31866	000123-35-3	94
2 .beta.-Myrcene $$ 1,6-Octadiene,...	31854	000123-35-3	94
3 .beta.-Myrcene $$ 1,6-Octadiene,...	31853	000123-35-3	93

Unknown Spectrum based on Apex

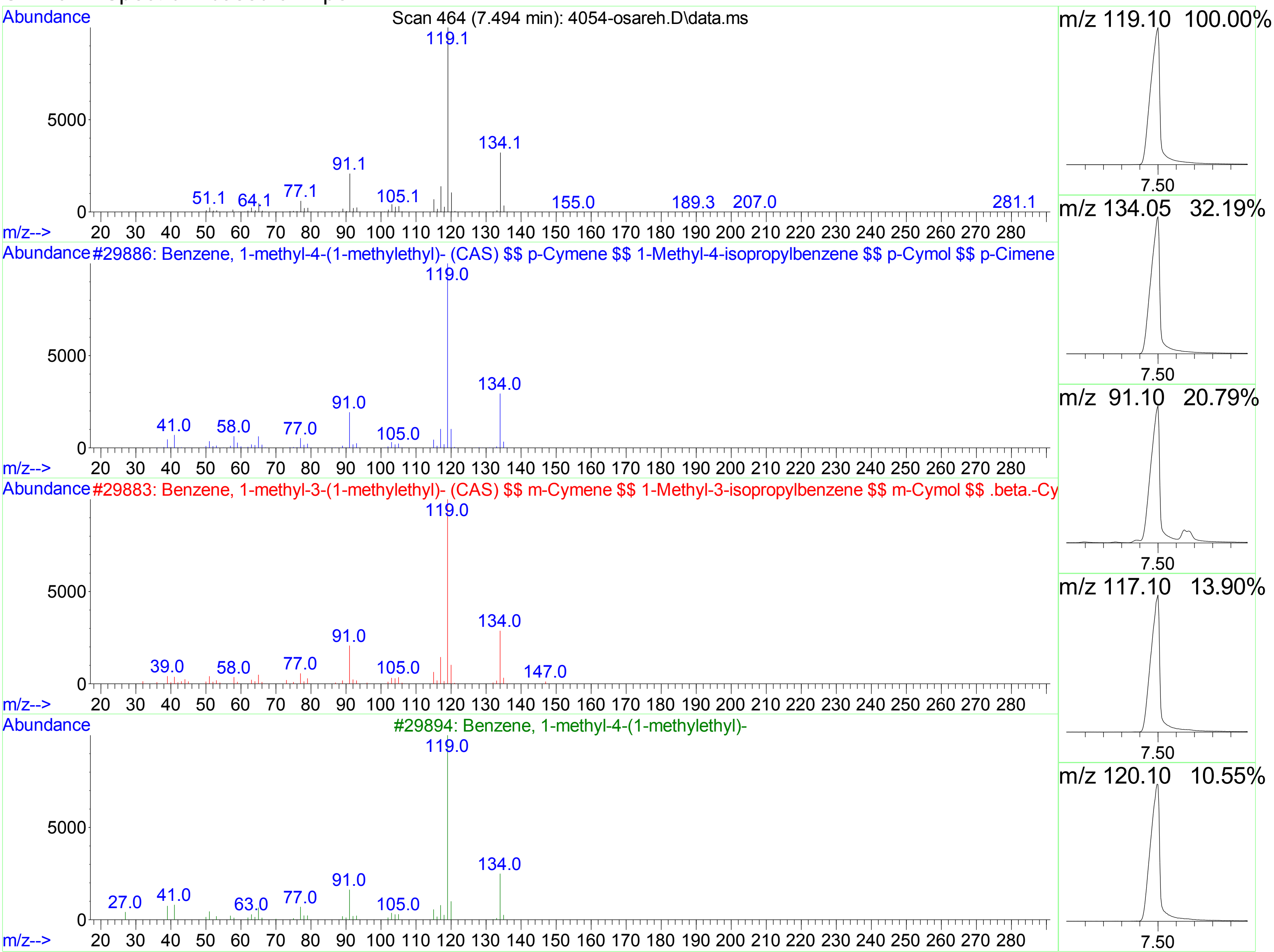

Data File: G:\ÌÒÇÑÔ 1399\99-09-27\4054-osareh.D
Sample : 4054-osareh

Peak Number: 5 at 7.494 min Area: 572751971 Area % 37.14

The 3 best hits from each library. Ref\# CAS\# Qual

E:\Database\wiley7n.l

		Ref#	CAS#	Qual
1	Benzene, 1-methyl-4-(1-methyleth...	29886	000099-87-6	95
2	Benzene, 1-methyl-3-(1-methyleth...	29883	000535-77-3	95
3	Benzene, 1-methyl-4-(1-methyleth...	29894	000099-87-6	95

Library Search Report - ChemStation Integrator

Unknown Spectrum based on Apex

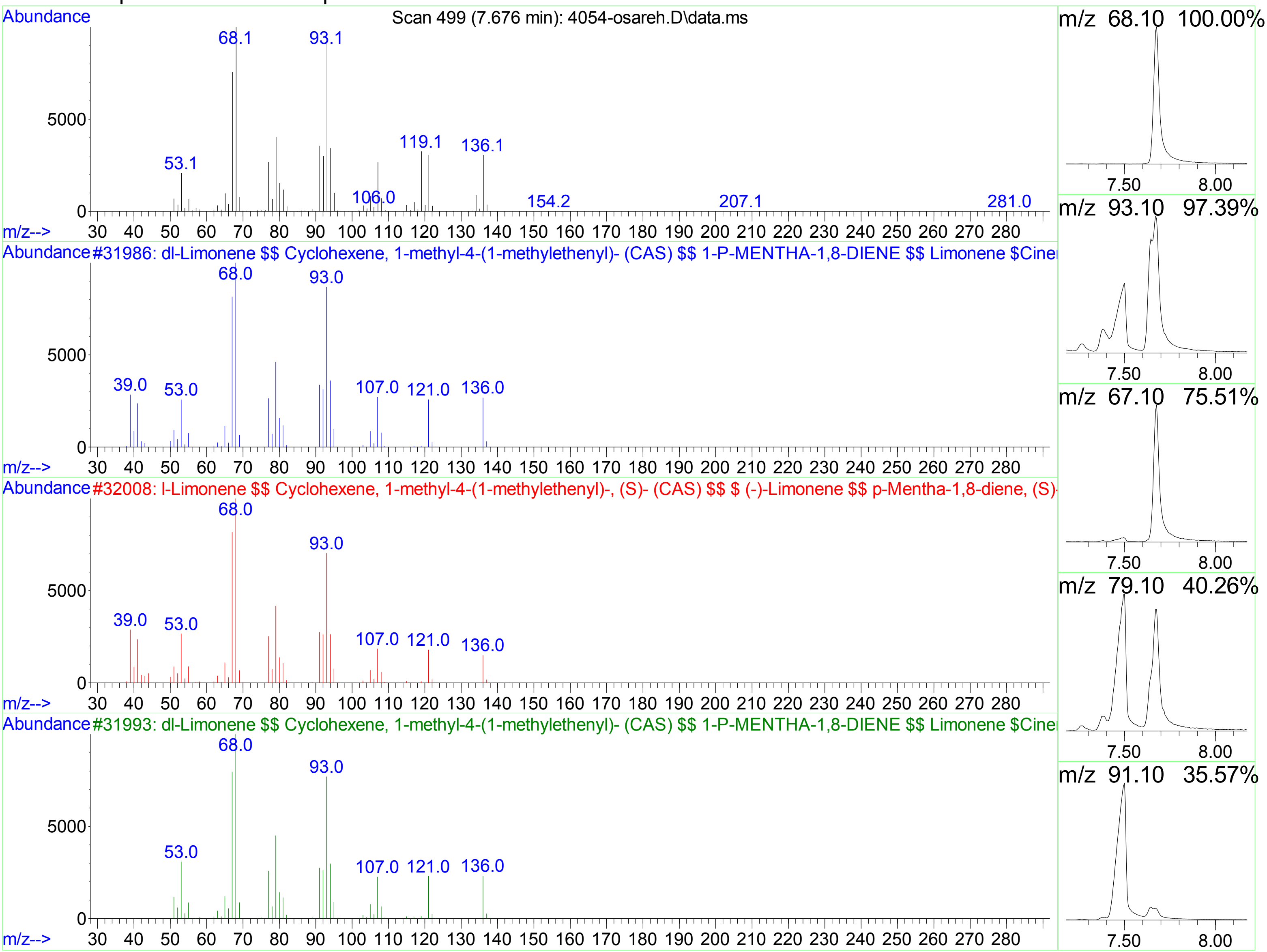

Data File: G:\ÌÒÇÑÔ 1399\99-09-27\4054-osareh.D
Sample : 4054-osareh

Peak Number: 6 at 7.676 min Area: 24816957 Area % 1.61

The 3 best hits from each library.	Ref#	CAS#	Qual
E:\Database\wiley7n.l			
1 dl-Limonene $$ Cyclohexene, 1-me...	31986	000138-86-3	99
2 l-Limonene $$ Cyclohexene, 1-met...	32008	005989-54-8	98
3 dl-Limonene $$ Cyclohexene, 1-me...	31993	000138-86-3	97

Unknown Spectrum based on Apex

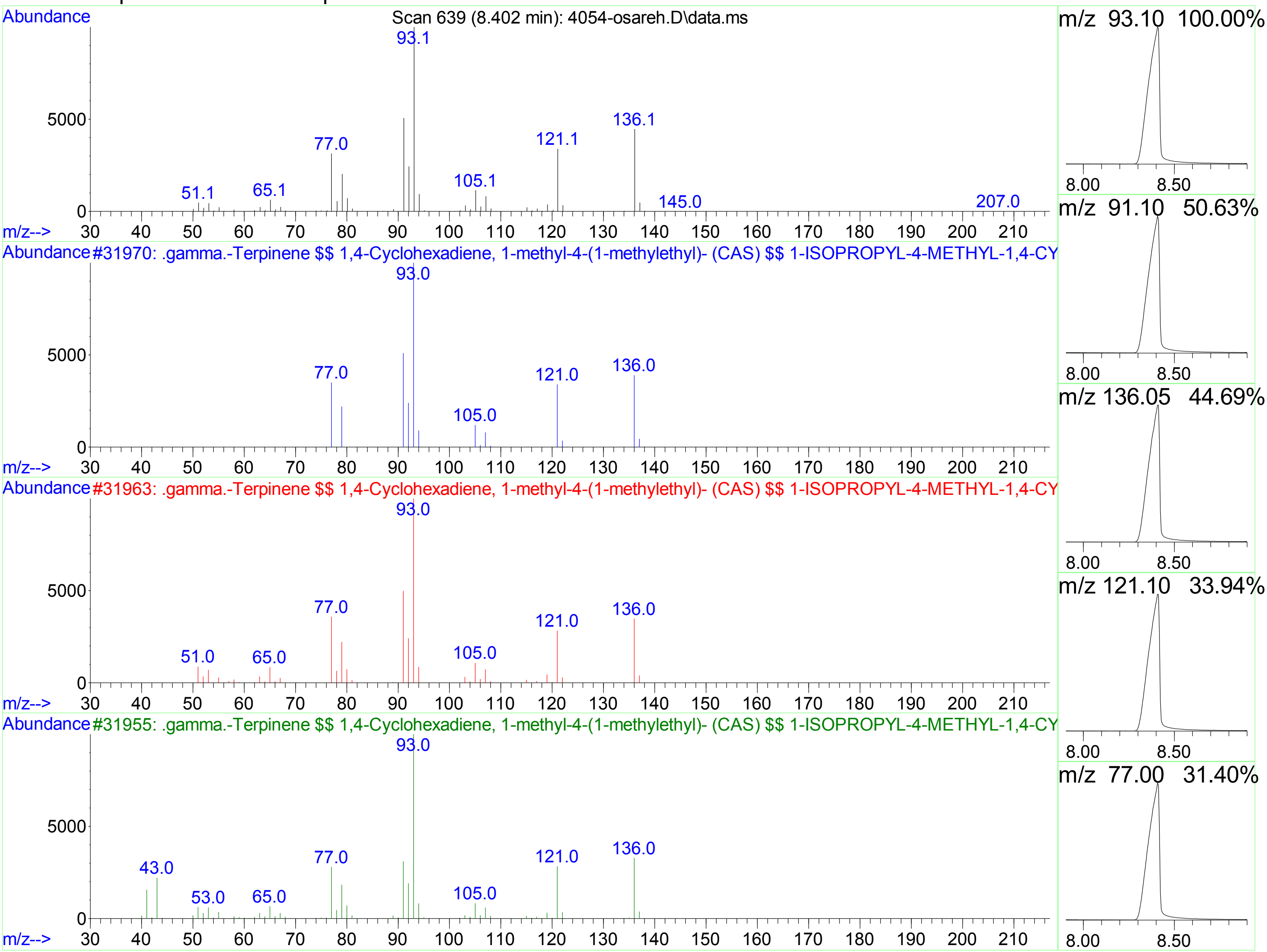

Data File: G:\ÌÒÇÑÔ 1399\99-09-27\4054-osareh.D
Sample : 4054-osareh

Peak Number: 7 at 8.402 min Area: 731111571 Area % 47.40

The 3 best hits from each library.	Ref\#	CAS\#	Qual
E:\Database\wiley7n.l			
1 .gamma.-Terpinene $$ 1,4-Cyclohe...	31970	000099-85-4	97
2 .gamma.-Terpinene $$ 1,4-Cyclohe...	31963	000099-85-4	96
3 .gamma.-Terpinene $$ 1,4-Cyclohe...	31955	000099-85-4	96

Unknown Spectrum based on Apex

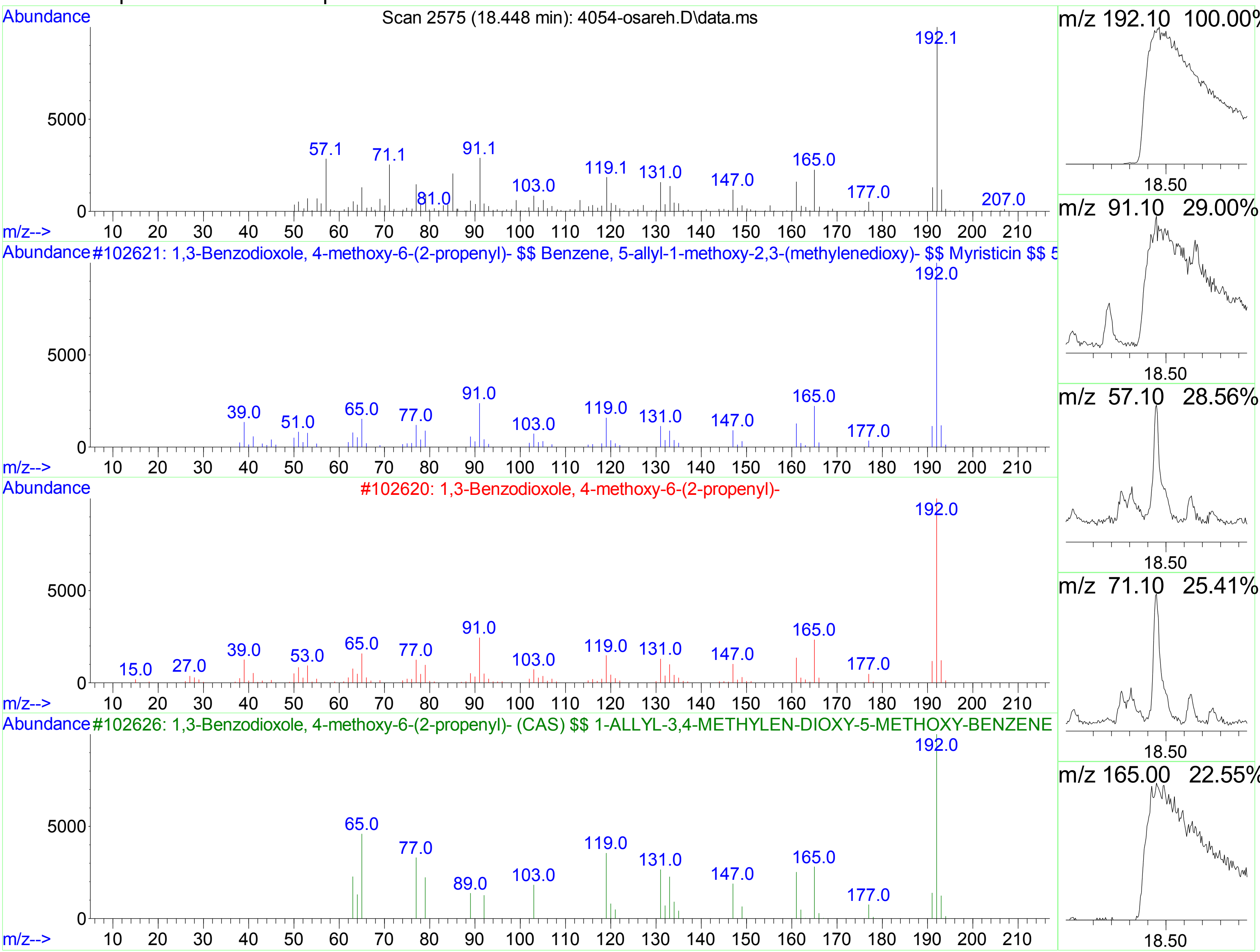

Data File: G:\ÌÒÇÑÔ 1399\99-09-27\4054-osareh.D
Sample : 4054-osareh

Peak Number: 8 at 18.448 min Area: 10645933 Area % 0.69

The 3 best hits from each library.	Ref#	CAS#	Qual
E:\Database\wiley7n.l			
1 1,3-Benzodioxole, 4-methoxy-6-(2...	102621	000607-91-0	98
2 1,3-Benzodioxole, 4-methoxy-6-(2...	102620	000607-91-0	98
3 1,3-Benzodioxole, 4-methoxy-6-(2...	102626	000607-91-0	97

Unknown Spectrum based on Apex

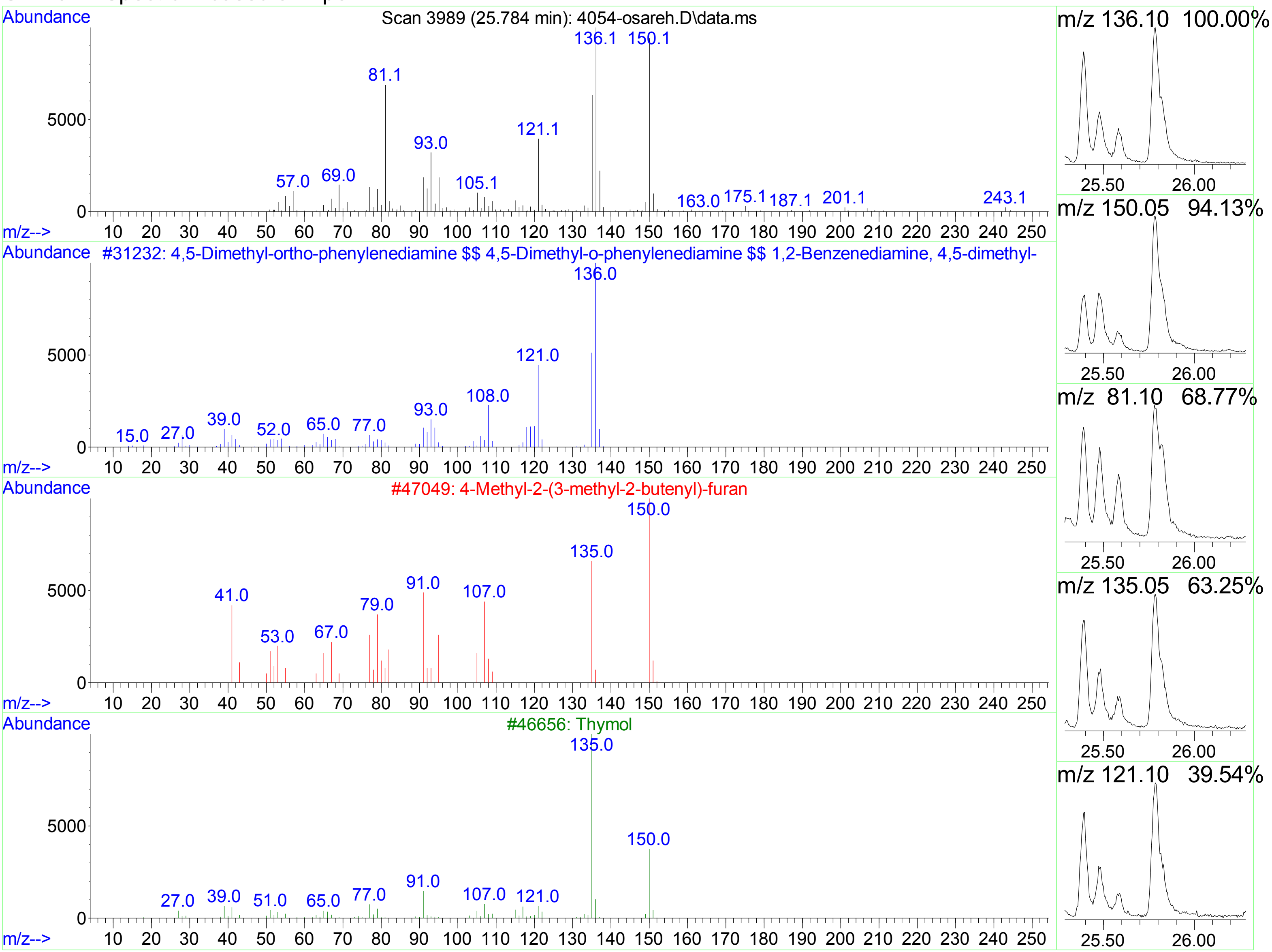

Data File: G:\ÌÒÇÑÔ 1399\99-09-27\4054-osareh.D
Sample : 4054-osareh

Peak Number: 9 at 25.784 min Area: 6887616 Area % 0.45

The 3 best hits from each library.	Ref#	CAS#	Qual
E:\Database\wiley7n.l			
1 4,5-Dimethyl-ortho-phenylenediam...	31232	003171-45-7	43
2 4-Methyl-2-(3-methyl-2-butenyl)-...	47049	000000-00-0	43
3 Thymol	46656	000089-83-8	38

Unknown Spectrum based on Apex

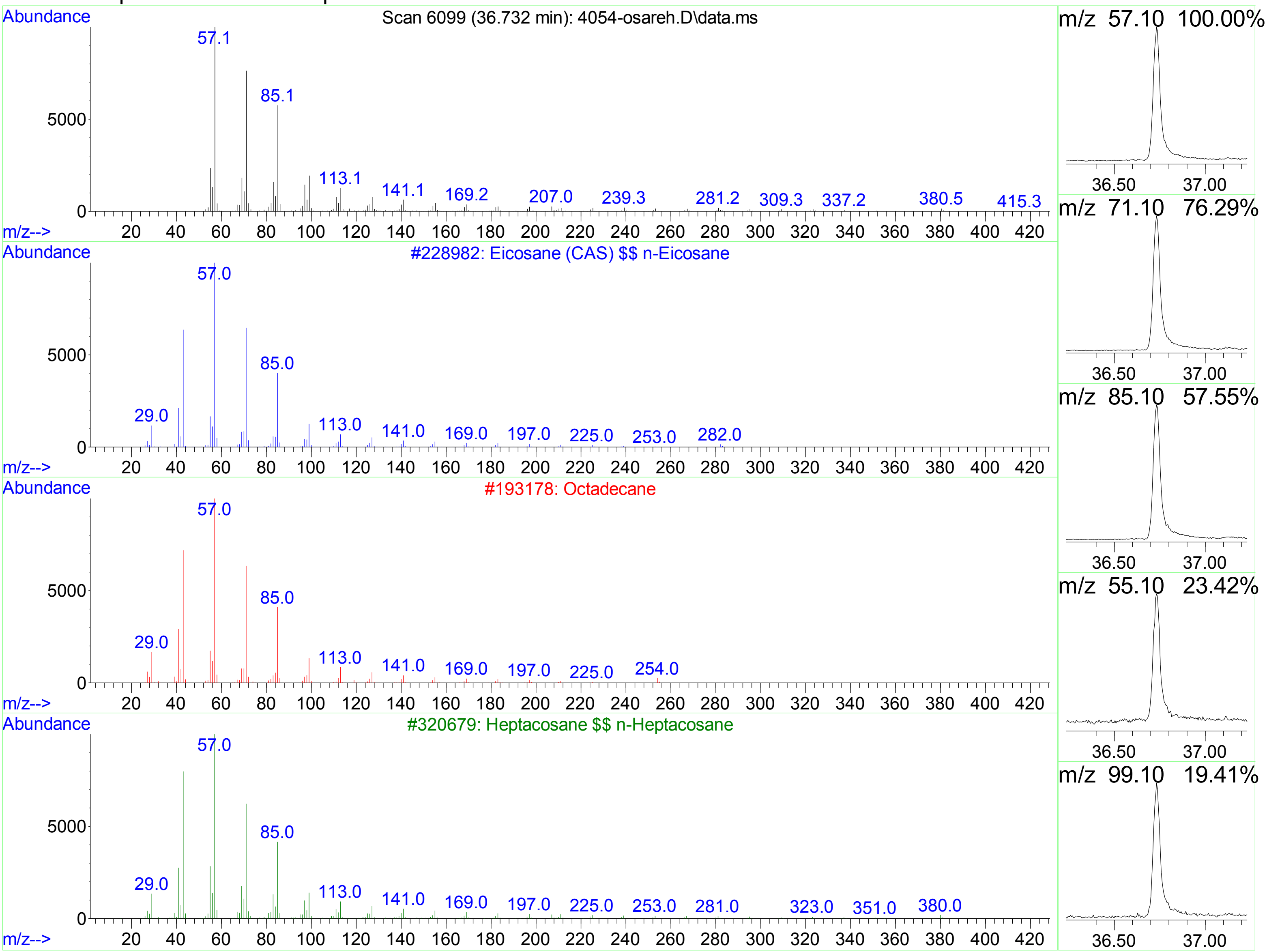

Data File: G:\ÌÒÇÑÔ 1399\99-09-27\4054-osareh.D
Sample : 4054-osareh

Peak Number: 10 at 36.732 min Area: 17261463 Area % 1.12

The 3 best hits from each library. Ref\# CAS\# Qual

E:\Database\wiley7n.l

1 Eicosane (CAS) $$ n-Eicosane 228982 000112-95-8 97
2 Octadecane 193178 000593-45-3 96
3 Heptacosane $$ n-Heptacosane 320679 000593-49-7 96

Unknown Spectrum based on Apex

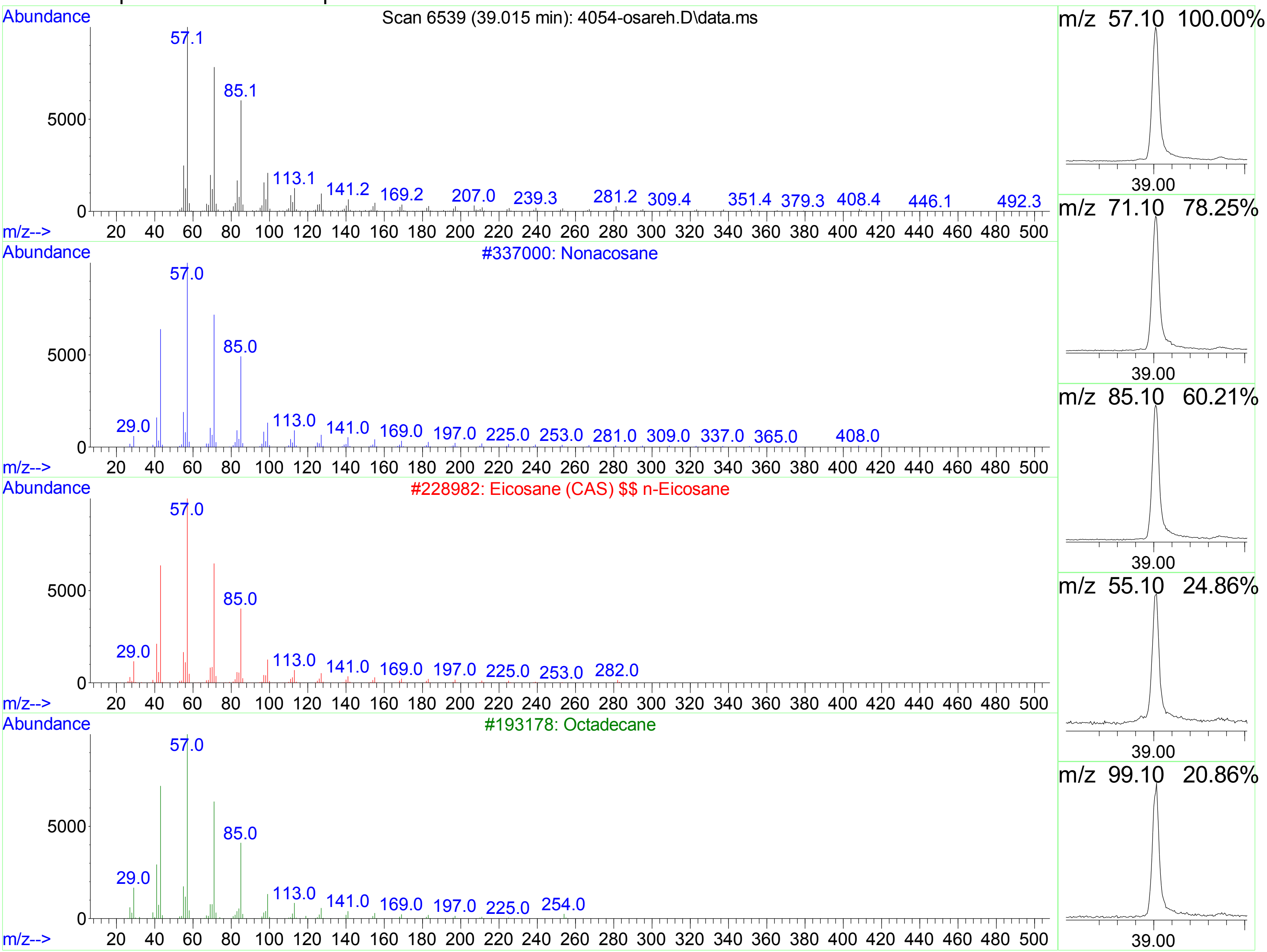

Data File: G:\ÌÒÇÑÔ 1399\99-09-27\4054-osareh.D
Sample : 4054-osareh

Peak Number: 11 at 39.015 min Area: 25007756 Area % 1.62

The 3 best hits from each library.	Ref#	CAS#	Qual
E:\Database\wiley7n.l			
1 Nonacosane	337000	000630-03-5	98
2 Eicosane (CAS) $$ n-Eicosane	228982	000112-95-8	97
3 Octadecane	193178	000593-45-3	96

Unknown Spectrum based on Apex

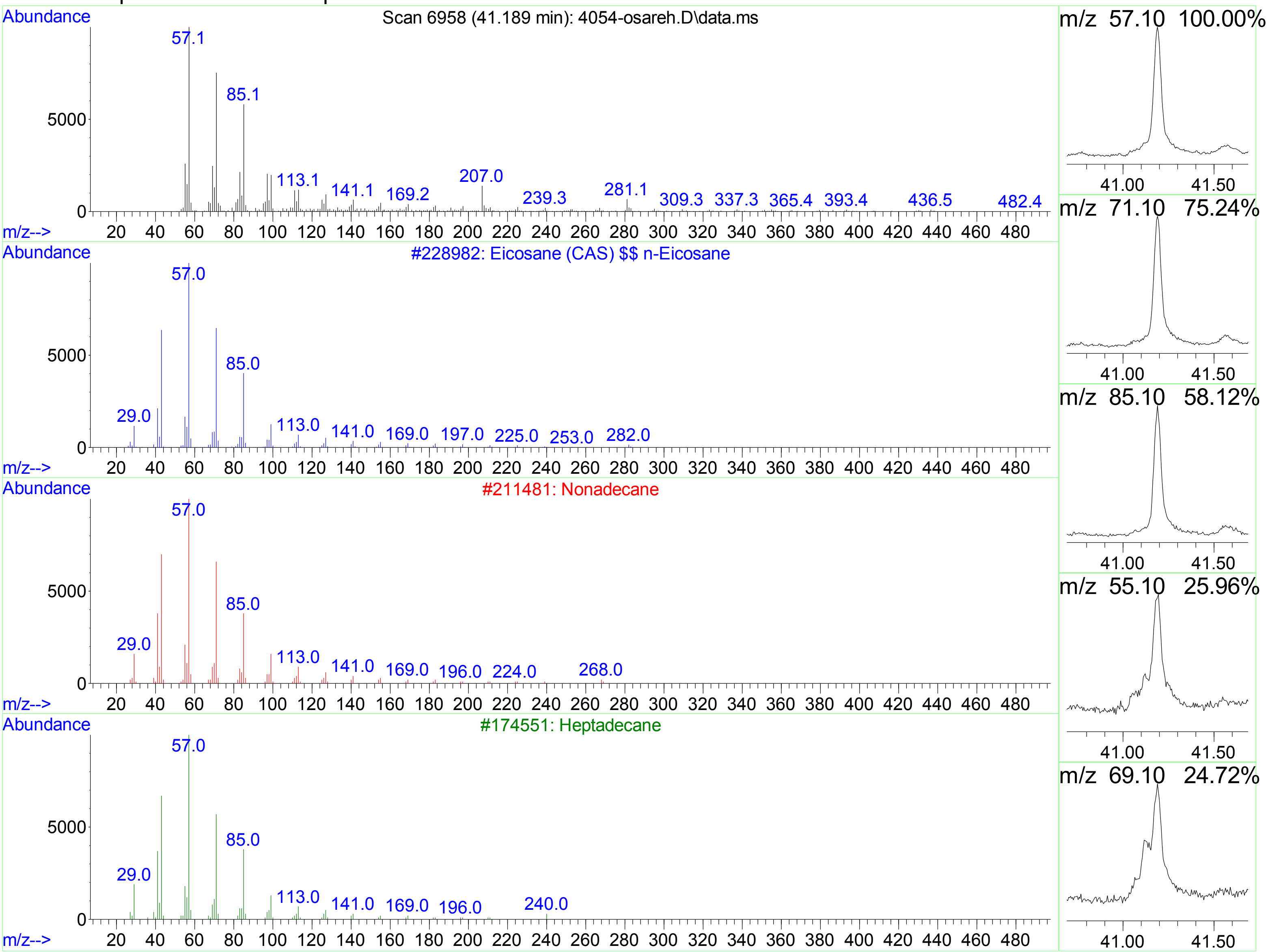

Data File: G:\ÌÒÇÑÔ 1399\99-09-27\4054-osareh.D
Sample : 4054-osareh

Peak Number: 12 at 41.189 min Area: 16361038 Area % 1.06

The 3 best hits from each library.	Ref\#	CAS\#	Qual
E:\Database\wiley7n.l			
1 Eicosane (CAS) $$ n-Eicosane	228982	000112-95-8	98
2 Nonadecane	211481	000629-92-5	97
3 Heptadecane	174551	000629-78-7	97

Unknown Spectrum based on Apex

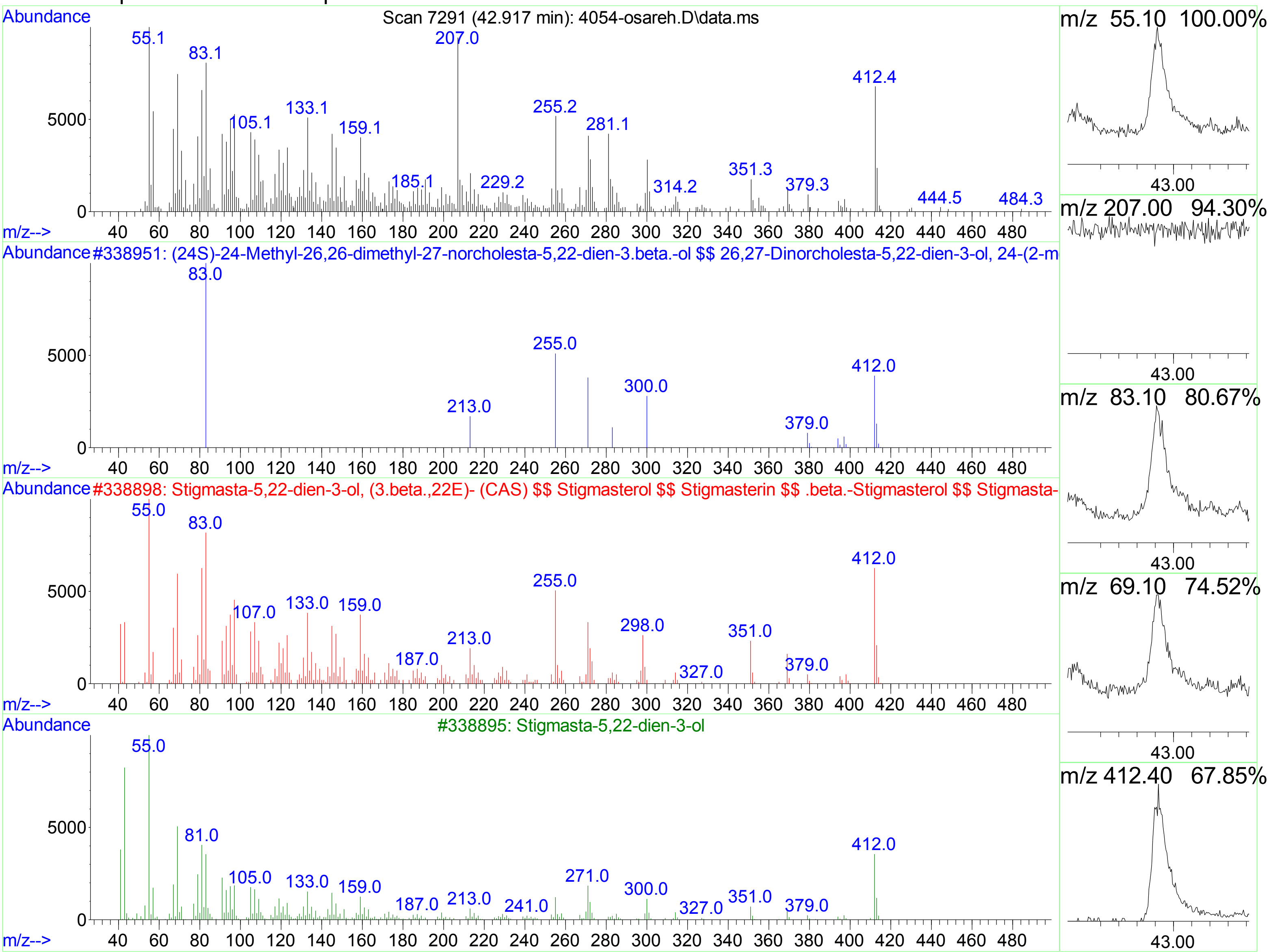

Data File: G:\ÌÒÇÑÔ 1399\99-09-27\4054-osareh.D
Sample : 4054-osareh

Peak Number: 13 at 42.917 min Area: 13442558 Area % 0.87

The 3 best hits from each library. Ref\# CAS\# Qual

E:\Database\wiley7n.l

1	(24S)-24-Methyl-26,26-dimethyl-2...	338951	091229-07-1	89
2	Stigmasta-5,22-dien-3-ol, (3.bet...	338898	000083-48-7	89
3	Stigmasta-5,22-dien-3-ol	338895	000000-00-0	89

Library Search Report - ChemStation Integrator

Unknown Spectrum based on Apex

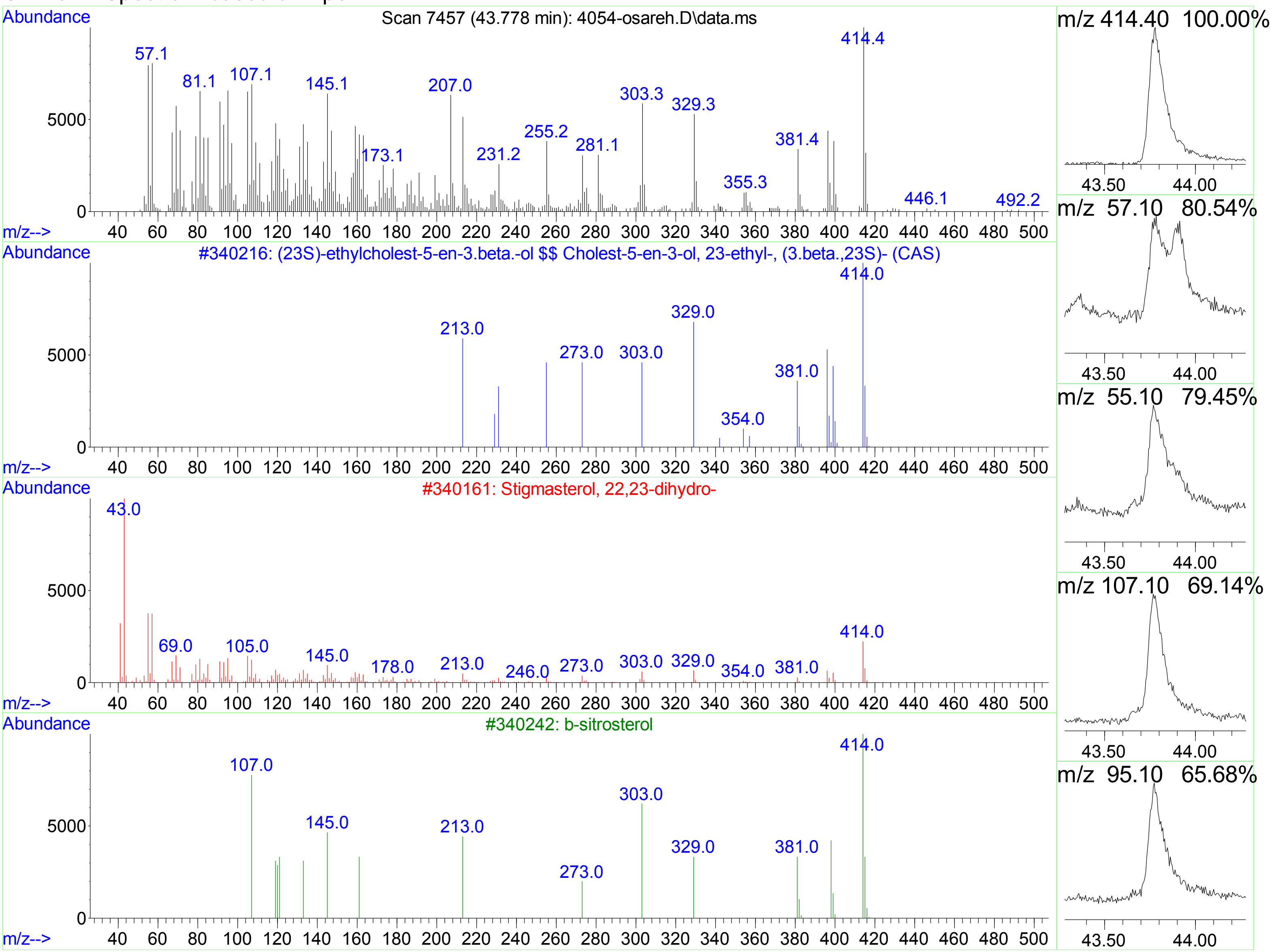

Data File: G:\lÒÇÑÔ 1399\99-09-27\4054-osareh.D
Sample : 4054-osareh

Peak Number: 14 at 43.778 min Area: 33272627 Area % 2.16

The 3 best hits from each library.	Ref#	CAS#	Qual
E:\Database\wiley7n.l			
1 (23S)-ethylcholest-5-en-3.beta.-...	340216	113845-28-6	99
2 Stigmasterol, 22,23-dihydro-	340161	000000-00-0	99
3 b-sitrosterol	340242	000000-00-0	95
